# Effect of empagliflozin on ventricular arrhythmias in patients with type 2 diabetes treated with an implantable cardioverter-defibrillator: the EMPA-ICD trial

**DOI:** 10.1186/s12933-024-02309-9

**Published:** 2024-06-28

**Authors:** Shinya Fujiki, Kenichi Iijima, Yoshihisa Nakagawa, Kazuyoshi Takahashi, Masaaki Okabe, Kengo Kusano, Shingen Owada, Yusuke Kondo, Kenichi Tsujita, Wataru Shimizu, Hirofumi Tomita, Masaya Watanabe, Morio Shoda, Masafumi Watanabe, Takashi Tokano, Toyoaki Murohara, Takashi Kaneshiro, Takeshi Kato, Hidemori Hayashi, Koji Maemura, Shinichi Niwano, Tomio Umemoto, Hisako Yoshida, Keiko Ota, Takahiro Tanaka, Nobutaka Kitamura, Koichi Node, Tohru Minamino, for the EMPA ICD investigators

**Affiliations:** 1 Department of Cardiovascular Medicine, Niigata University Graduate School of Medical and Dental Sciences, Niigata, Japan; 2 Department of Cardiovascular Biology and Medicine, Juntendo University Graduate School of Medicine, 2-1-1 Hongo, Bunkyo-ku, Tokyo, 113-8421 Japan; 3 Department of Cardiovascular Medicine, Shiga University of Medical Science, Otsu, Shiga Japan; 4 Department of Cardiology, Niigata City General Hospital, Niigata, Japan; 5 Department of Cardiology, Tachikawa General Hospital, Niigata, Japan; 6 Department of Cardiovascular Medicine, National Cerebral and Cardiovascular Center, Osaka, Japan; 7 Department of Internal Medicine, Division of Cardiology, Iwate Medical University, Iwate, Japan; 8 Department of Cardiovascular Medicine, Chiba University Graduate School of Medicine, Chiba, Japan; 9 Department of Cardiovascular Medicine, Graduate School of Medical Sciences, Kumamoto University, Kumamoto, Japan; 10 Department of Cardiovascular Medicine, Nippon Medical School, Tokyo, Japan; 11 Department of Cardiology and Nephrology, Hirosaki University Graduate School of Medicine, Aomori, Japan; 12 Department of Cardiovascular Medicine, Hokkaido University Graduate School of Medicine, Sapporo, Hokkaido Japan; 13 Department of Cardiology, Tokyo Women’s Medical University Hospital, Tokyo, Japan; 14 Department of Cardiology, Pulmonology, and Nephrology, Yamagata University Faculty of Medicine, Yamagata, Japan; 15 Department of Cardiology, Juntendo University Urayasu Hospital, Chiba, Japan; 16 Department of Cardiology, Nagoya University Graduate School of Medicine, Nagoya, Aichi Japan; 17 Department of Cardiovascular Medicine, Fukushima Medical University, Fukushima, Japan; 18 Department of Cardiology, Graduate School of Medical Science, Kanazawa University, Kanazawa, Japan; 19 Department of Cardiovascular Medicine, Nagasaki University Graduate School of Biomedical Sciences, Nagasaki, Japan; 20 Department of Cardiovascular Medicine, Kitasato University, Kanagawa, Japan; 21 Department of Cardiology, Jichi Medical University Saitama Medical Center, Saitama, Japan; 22 Department of Medial Statistics, Osaka Metropolitan University Graduate School of Medicine, Osaka, Japan; 23 Data Management Group, Department of Clinical Research Support, Center for Clinical Research and Innovation, Osaka Metropolitan University Hospital, Osaka, Japan; 24 Clinical and Translational Research Center, Niigata University Medical and Dental Hospital, Niigata, Japan; 25 Department of Cardiovascular Medicine, Saga University, Saga, Japan; 26 Japan Agency for Medical Research and Development-Core Research for Evolutionary Medical Science and Technology (AMED-CREST), Japan Agency for Medical Research and Development, Tokyo, Japan

**Keywords:** Ventricular arrhythmia, Sodium-glucose cotransporter 2, Type 2 diabetes, Empagliflozin

## Abstract

**Background:**

Sodium-glucose cotransporter 2 (SGLT2) inhibitors reduce the risk of hospitalization for heart failure and cardiovascular death with type 2 diabetes; however, their effect on arrhythmias is unclear. The purpose of this study was to investigate the effects of empagliflozin on ventricular arrhythmias in patients with type 2 diabetes.

**Methods:**

A total of 150 patients with type 2 diabetes who were treated with an implantable cardioverter-defibrillator or cardiac resynchronization therapy defibrillator (ICD/CRT-D) were randomized to once-daily empagliflozin or placebo for 24 weeks. The primary endpoint was the change in the number of ventricular arrhythmias from the 24 weeks before to the 24 weeks during treatment. Secondary endpoints included the change in the number of appropriate device discharges and other values.

**Results:**

In the empagliflozin group, the number of ventricular arrhythmias recorded by ICD/CRT-D decreased by 1.69 during treatment compared to before treatment, while in the placebo group, the number increased by 1.79. The coefficient for the between-group difference was − 1.07 (95% confidence interval [CI] − 1.29 to − 0.86; *P* < 0.001). The change in the number of appropriate device discharges during and before treatment was 0.06 in the empagliflozin group and 0.27 in the placebo group, with no significant difference between the groups (*P* = 0.204). Empagliflozin was associated with an increase in blood ketones and hematocrit and a decrease in blood brain natriuretic peptide and body weight.

**Conclusions:**

In patients with type 2 diabetes treated with ICD/CRT-D, empagliflozin reduces the number of ventricular arrhythmias compared with placebo.

*Trial registration* jRCTs031180120.

**Supplementary Information:**

The online version contains supplementary material available at 10.1186/s12933-024-02309-9.

## Introduction

Ventricular arrhythmias are one of the leading causes of cardiovascular death in patients with cardiovascular disease [1]. In particular, patients with type 2 diabetes are known to be at high risk of arrhythmias and sudden death [2–4]. Large clinical trials have demonstrated that in patients with type 2 diabetes, sodium-glucose cotransporter 2 (SGLT2) inhibitors reduce the risk of hospitalization for heart failure and cardiovascular death, including sudden death [5–7], and other clinical trials have shown that SGLT2 inhibitors have beneficial effects on the risk of heart failure hospitalization and cardiovascular death irrespective of type 2 diabetes [8–10]. Recently, a post hoc analysis of the DAPA-HF (Dapagliflozin and Prevention of Adverse Outcomes in Heart Failure) trial data revealed that dapagliflozin reduces the risk of ventricular arrhythmias in heart failure patients with reduced ejection fraction [11]. The results of a meta-analysis of randomized controlled trials also suggested that SGLT2 inhibitors have antiarrhythmic effects on not only ventricular arrhythmias but also atrial fibrillation and sudden cardiac death [12–14]. Various molecular mechanisms have been proposed for the antiarrhythmic effects of SGLT2 inhibitors [15, 16], but at present, no prospective studies have directly examined whether and how these drugs have antiarrhythmic effects. Therefore, we designed the EMPA-ICD (Empagliflozin in Patients with Type 2 Diabetes Treated with an Implantable Cardioverter-Defibrillator) trial to prospectively evaluate the antiarrhythmic effects of the SGLT-2 inhibitor empagliflozin on ventricular arrhythmias in patients with type 2 diabetes treated with an implantable cardioverter-defibrillator or cardiac resynchronization therapy-defibrillator (ICD/CRT-D) [17].

## Methods

### Trial design and oversight

The trial design is described elsewhere [17] and in the Supplementary Note 2. The EMPA-ICD trial was a prospective, multicenter, placebo-controlled, double-blind, randomized, investigator-initiated clinical trial in patients with cardiovascular disease and type 2 diabetes that compared the efficacy in treating arrythmia of empagliflozin 10 mg once daily and matching placebo; both study drugs were added to standard care. The trial was approved by the Certified Review Board of the Niigata University Graduate School of Medicine and was performed in compliance with the Declaration of Helsinki and the Clinical Trials Act. All enrolled patients provided written informed consent prior to eligibility screening. The sponsors were Nippon Boehringer Ingelheim Co. Ltd. and Eli Lilly and Company.

The Steering Committee developed the protocol and statistical analysis plan, oversaw patient recruitment, supervised data analysis, identified problems during the conduct of the study, discussed solutions, and coordinated any actions required for study operations; the Data and Safety Monitoring Board evaluated safety-related data, discussed the need to amend the protocol, considered the appropriateness of continuing the study, and made respective recommendations; and the Event Assessment Committee evaluated the data related to each arrhythmia event, considered the appropriateness of continuing the study, and made recommendations.

### Patients

Men and women aged 20 years or older with cardiovascular disease and type 2 diabetes were eligible if they were being treated with ICD/CRT-D. Initially, HbA1c levels (6.5–10.0) were included in the inclusion criteria, but following the results of the DAPA-HF trial [8], the Steering Committee recommended eliminating the HbA1c inclusion criteria after June 2020. Key exclusion criteria for pre-randomization eligibility included an estimated glomerular filtration rate (eGFR) of less than 30 mL/min/1.73 m^2^, type 1 diabetes, and the following events likely to affect the onset of clinically significant ventricular arrhythmia within 24 weeks before the eligibility tests: change of antiarrhythmic drug; catheter ablation for ventricular arrhythmia; coronary revascularization; open-heart surgery; development of coronary artery disease, stroke, or transient ischemic stroke; seizure; infection requiring hospitalization; and heart failure requiring hospitalization.

### Trial procedures

All participants who met the enrolment criteria were randomly assigned (1:1) to treatment with empagliflozin or placebo. Treatment was assigned with the stratified allocation method on the basis of left ventricular ejection fraction (LVEF; < 40% or ≥ 40%), age (< 65 or ≥ 65 years), and sex. After randomization, patients received empagliflozin (10 mg once daily) or placebo for 24 weeks in a blinded manner. Empagliflozin and placebo were provided by Boehringer Ingelheim in tablet form. The placebo was a tablet that was identical in smell, color, and size to the study drug and contained no drug component. They were prepacked in bottles and consecutively numbered according to a computer-generated stratified permuted randomization method by Specially Appointed Associate Professor Hisako Yoshida, who is responsible for allocating; the details of the series were unknown to any of the investigators. Numbered study drugs were stored in a central office and sent to each site by a blinded pharmacist, according to the drug number issued after the investigator enrolled the patient on the computer. Patients were evaluated at trial visits every 3 months to assess clinical status and adverse events. If an investigator judged that a patient’s blood glucose level was insufficiently controlled according to the Japanese treatment guidelines for diabetes [18], they were permitted to administer new diabetic drugs other than SGLT2 inhibitors or to increase the dose of such diabetic drugs. In patients receiving treatment for arrhythmias or underlying cardiac disease, changes of antiarrhythmic drugs were avoided as far as possible during the study. Recordings by ICD/CRT-D were evaluated at week 0 for the 24-week baseline period and at week 24 for the 24-week treatment period. Holter monitoring and hematological and echocardiographic tests were performed before (at week 0) and after treatment (at week 24). Additional details of the trial design are provided in the Supplementary Note 2.

### Endpoints

The primary endpoint was the change in the number of ventricular arrhythmias, including non-sustained ventricular tachycardia (NSVT), ventricular tachycardia (VT), and ventricular fibrillation (VF), recorded by ICD/CRT-D during the 24-week baseline period (assessed at week 0) and the 24-week treatment period (assessed at week 24). Secondary endpoints were the following parameters: the number of ventricular arrhythmias recorded by ICD/CRT-D during the 24-week treatment period (assessed at week 24), the change in the number of anti-tachycardia pacing events and shock therapies recorded by ICD/CRT-D from the baseline period (assessed at week 0) to the treatment period (assessed at week 24), and the change in the number of total, single, and double ventricular premature contractions (VPC) and of NSVTs, and VTs recorded by Holter monitoring before (week 0) and after treatment (week 24). Additional secondary endpoints were the changes from before (week 0) to after treatment (week 24) in serum concentrations of ketones (acetoacetic acid, 3-hydroxybutyric acid, and total ketone bodies) and plasma concentrations of catecholamines (adrenalin, noradrenalin, and dopamine); blood ketones and catecholamines were assessed in the fasting and bed-resting state and measured at a central laboratory (SRL, Inc., Tokyo, Japan).

### Statistical analysis

Statistical analyses were performed according to a predefined statistical analysis plan (Supplementary Note 2). The sample size was calculated using a generalized linear model (GLM) with the number of ventricular arrhythmias as the dependent variable, the time and treatment group as independent variables, and a significance level of 0.05. We applied the incidence rate ratio (IRR) from a previous study [19] and set the feasible number of patients to be enrolled within the enrollment period (April 2019 to April 2020) at 210, based on the intention that 20 medical institution sites would participate in the study. Assuming that 10 patients (5%) would drop out, we planned to include 200 patients in the present study, which would provide a power of 80%. In this study, an IRR of 1.44 was used in the sample size estimation [19]. We assumed a Poisson distribution, with a mean of 1 event per patient at baseline in both the treatment and placebo groups. After 24 weeks, the mean number of events was 0.8 in the treatment group and 1.152 in the placebo group, which was hypothesized to correspond to an IRR of 1.44 (1.152/0.8). However, the planned number of patients could not be recruited by April 2020 because of the COVID-19 pandemic. Therefore, the enrollment period was extended by 1 year, and 11 participating sites were added to the study during this extension of the enrollment period. A total of 150 patients were eventually enrolled. We confirmed that the final data set involving 150 randomized individuals included in the analysis was sufficient to ensure at least 70% power.

Numerical data are presented as means ± SD, and frequencies are presented as percentages for descriptive purposes. The primary outcome (ventricular arrhythmias) and secondary outcome (appropriate device discharges) were analyzed by GLM in the intention-to-treat population, which included all randomized patients. The models were used to evaluate between-group differences in changes over time. The models included the time (week 0 or week 24), the treatment group (empagliflozin or placebo), and the interaction between time and treatment group as independent variables as well as the number of events or laboratory values as dependent variables; no imputation of missing data was performed. For the analysis of differences between week 0 and week 24 in other outcome measures, Student’s t-test was applied when variances were assumed to be equal; Welch’s t-test was used in cases of unequal variances. The *P* value was 2-sided, and a *P* value less than 0.05 was considered statistically significant. All analyses were performed using the SAS statistical software package version 9.4 (SAS Institute Inc., Cary, NC, USA).

## Results

### Patients

**Fig. 1:**
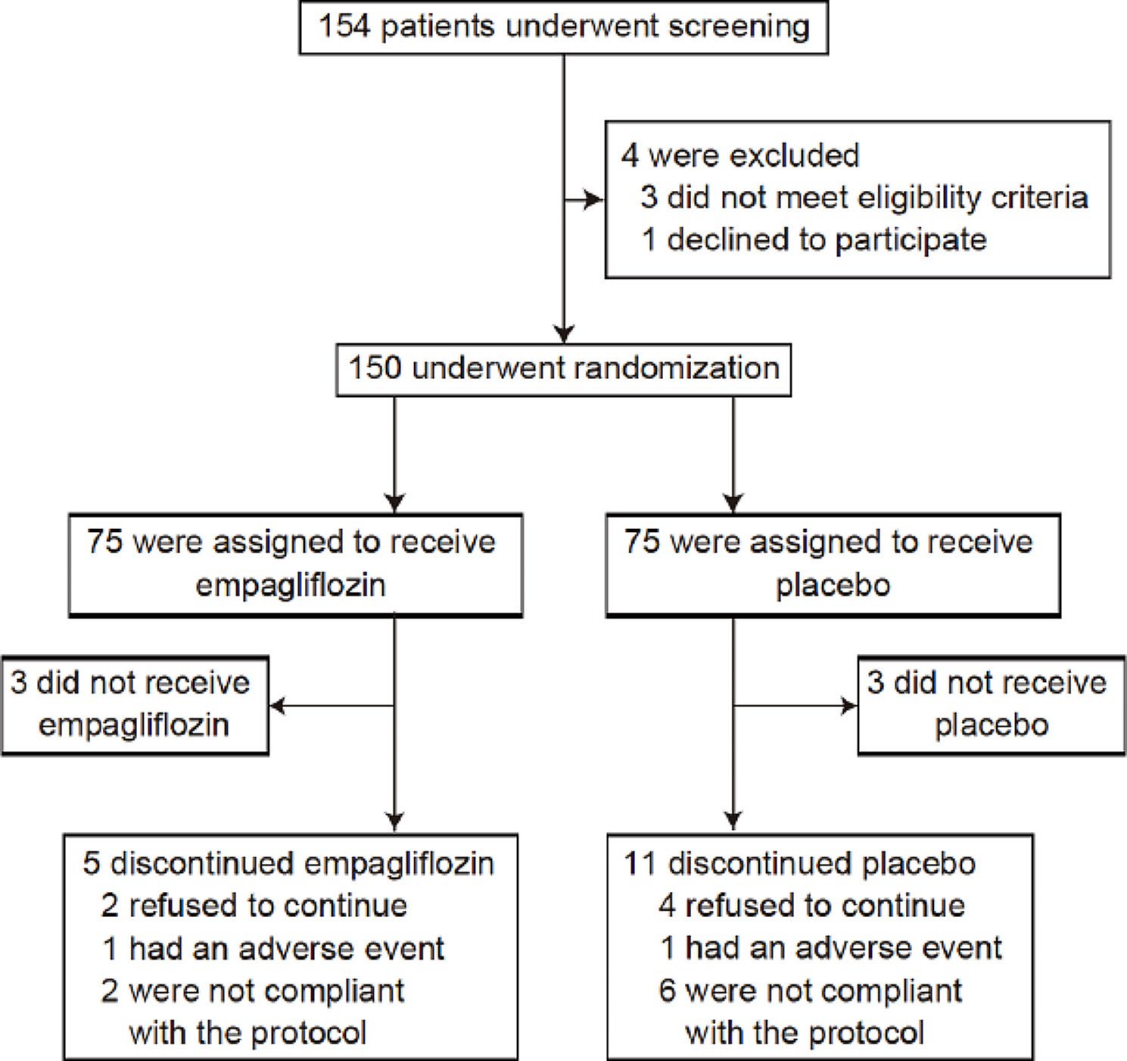
Enrollment and follow-up. All the patients who underwent randomization were included in the primary analysis

From April 19, 2019, through April 20, 2021, a total of 150 patients were randomly assigned to receive either empagliflozin or placebo at 31 centers in Japan (Fig. 1). All randomized patients were included in the primary analysis. At baseline (week 0), patient characteristics and treatments for cardiovascular disease were well balanced between the groups and no significant differences were found (Table 1). The trial population was predominately male (83.3%) with body mass index in the normal range (25.2 ± 4.3 kg/m^2^) and a median age of 71 (64–76) years. The mean glycated hemoglobin was 7.1% ± 0.8%. Underlying cardiac diseases included ischemic heart disease (44.0%), cardiomyopathy (32.7%), and hereditary arrhythmic disease (10.0%). The mean LVEF was 46.0%, and 38.0% of study participants had systolic dysfunction (LVEF < 40). The final date of follow-up for data collection was November 2, 2021. The trial medication was discontinued for reasons other than death in 5 patients receiving empagliflozin and 11 patients receiving placebo; 2 patients discontinued treatment because of adverse events (Fig. 1). No defibrillator settings were changed during the study period in any of the patients.

**Table 1:** Patient characteristics at baseline

Characteristic^a^	Empagliflozin		Placebo	
	n		n	
Male sex—no. (%)	75	63 (84.0)	75	62 (82.7)
Median age—yr (IQR)	75	72.0 (64.0–76.0)	75	70.0 (63.0–76.0)
Smoking history—no. (%)	75	47 (62.7)	73	50 (68.5)
Indication for ICD implantation—no. (%)	75		75	
Primary prevention		12 (16.0)		12 (16.0)
Ventricular fibrillation		22 (29.3)		17 (22.7)
Monomorphic ventricular tachycardia		23 (30.7)		23 (30.7)
Polymorphic ventricular tachycardia		3 (4.0)		6 (8.0)
Non-sustained ventricular tachycardia		11 (14.7)		15 (20.0)
Other		4 (5.3)		2 (2.7)
Body mass index^b^	73	25.1 ± 4.3	72	25.3 ± 4.3
Heart rate—bpm	72	69.6 ± 9.3	72	67.6 ± 9.7
Systolic blood pressure—mmHg	72	118.3 ± 19.4	71	124.1 ± 20.1
Left ventricular ejection fraction	74		72	
Mean left ventricular ejection fraction (%)		46.9 ± 16.8		45.2 ± 15.2
Left ventricular ejection fraction<40%—no. (%)		30 (40.5)		27 (37.5)
BNP (pg/mL)	66	116.1 ± 128.9	64	137.3 ± 162.9
Glycated hemoglobin—%	69	7.2 ± 0.9	64	7.0 ± 0.5
Hematocrit (%)	74	41.3 ± 5.1	72	40.1 ± 5.1
Underlying cardiac diseases—no. (%)	75		75	
Ischemic heart disease		33 (44.0)		33 (44.0)
Cardiomyopathy		24 (32.0)		25 (33.3)
Hereditary arrhythmic disease		7 (9.3)		8 (10.7)
Cardiovascular history—no. (%)	75		75	
Atrial fibrillation		24 (32.0)		20 (26.7)
Hypertension		47 (62.7)		49 (65.3)
Dyslipidemia		56 (74.7)		49 (65.3)
Cerebrovascular disease		10 (13.3)		4 (5.3)
eGFR—mL/min/1.73 m^2^	70	57.7 ± 16.0	66	54.0 ± 15.2
Pharmacological treatment—no. (%)	73		71	
Glucose-lowering therapy		45 (61.6)		41 (57.7)
Metformin		13 (17.8)		13 (18.3)
Sulfonylurea		10 (13.7)		7 (9.9)
Dipeptidyl peptidase-4 inhibitor		36 (49.3)		33 (46.5)
Glucagon-like peptide-1 agonist		1 (1.4)		1 (1.4)
Insulin		7 (9.6)		4 (5.6)
Other		14 (19.2)		11 (15.5)
Beta-blockers		62 (84.9)		59 (83.1)
Angiotensin-converting enzyme inhibitors or angiotensin receptor blockers		52 (71.2)		53 (74.6)
Mineralocorticoid receptor antagonists		25 (34.2)		20 (28.2)
Diuretics		32 (43.8)		33 (46.5)
Calcium channel blockers		16 (21.9)		23 (32.4)
Antiarrhythmic drug		35 (47.9)		37 (52.1)
Cardiotonic drug		4 (5.5)		3 (4.2)
Non-pharmacological treatment—no. (%)				
PCI	75	20 (26.7)	75	22 (29.3)
CABG	75	6 (8.0)	75	9 (12.0)
Cardiac valve surgery	75	3 (4.0)	75	5 (6.7)
Catheter ablation	75	18 (24.0)	75	13 (17.3)
CRT-D	75	22 (29.3)	73	20 (27.4)

^a^Data are shown as mean ± SD unless otherwise indicated. Percentages may not total 100 because of rounding. No variable was significantly different between the two groups

^b^Body mass index is calculated as the weight in kilograms divided by the square of the height in meters

BNP, brain natriuretic peptide; CABG coronary artery bypass graft; CRT-D, cardiac resynchronization therapy defibrillator; eGFR, estimated glomerular filtration rate; ICD, implantable cardioverter-defibrillator; IQR, interquartile range

### Primary outcome

**Fig. 2:**
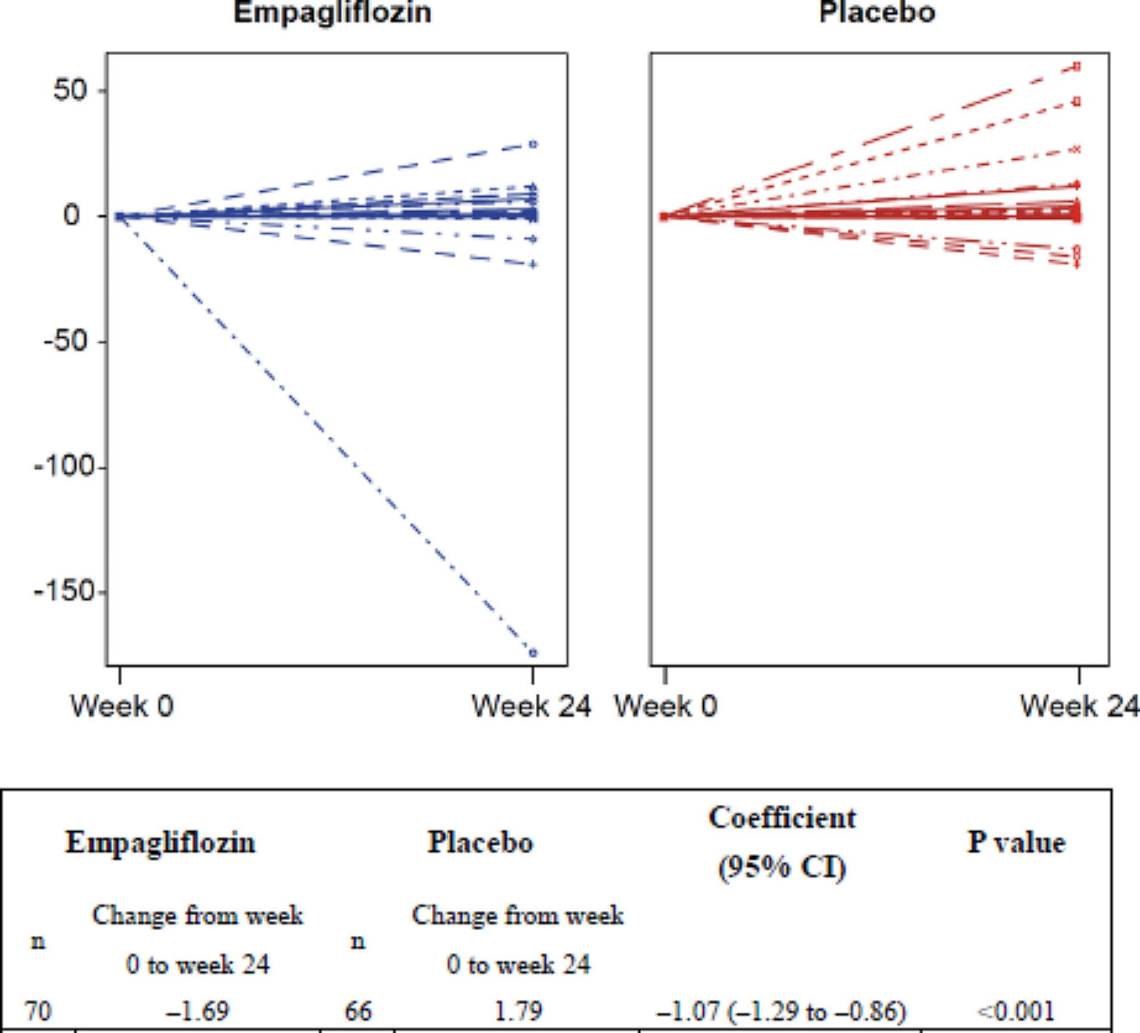
Changes in the number of ventricular arrhythmias before and after treatment. The figure shows the changes in the number of ventricular arrhythmias recorded by an implantable cardioverter-defibrillator or cardiac resynchronization therapy defibrillator from the 24 weeks before treatment (assessed at week 0) and to the 24 weeks during treatment (assessed at week 24) in the empagliflozin and placebo groups. *P* values were calculated by the generalized linear model to evaluate for significant differences in interactions between treatment group and period from baseline to week 24, which indicates a difference in the change of each variable over time between the empagliflozin and placebo groups

In the empagliflozin group, the total number of ventricular arrhythmias recorded by ICD/CRT-D decreased by 1.69 events during the 24-week treatment period compared with the 24-week baseline period (Fig. 2), but in the placebo group, the total number increased by 1.79 events from the baseline to the treatment period (Fig. 2). The coefficient for the between-group difference was − 1.07 (95% CI − 1.29 to − 0.86; *P* < 0.001; Fig. 2). Patient background characteristics that showed significant interactions and were therefore potential confounding factors for the effect of empagliflozin on ventricular arrhythmias were age; history of smoking; body mass index; LVEF; brain natriuretic peptide (BNP); glycated hemoglobin; hematocrit; history of ischemic heart disease; eGFR; use of angiotensin-converting enzyme inhibitors, angiotensin receptor blockers, diuretics, or antiarrhythmic drugs; and catheter ablation therapy (Supplementary Fig. 1). No significant differences were found in any of these factors between the empagliflozin and placebo groups (Table 1).

### Secondary outcomes

The change in the number of appropriate device discharges during and before treatment was 0.06 in the empagliflozin group and 0.27 in the placebo group, with no significant difference between the groups (*P* = 0.204; Table 2). The placebo group showed a trend towards an increase in the number of total VPCs, single VPCs, double VPCs, and VTs per day recorded by Holter monitoring at post-treatment compared to pre-treatment (Table 2).

**Table 2:** Secondary outcomes

Variable	Empagliflozin		Placebo		*P* value
	n	Change from week 0 to week 24	n	Change from week 0 to week 24	
Appropriate device discharge—events per 24 weeks	70	0.06	66	0.27	0.204^a^
Holter monitoring—events per day					
Total VPC	60	25.12	56	397.67	0.506^b^
Single VPC	59	− 28.92	55	126.19	0.741^c^
Double VPC	59	− 14.98	55	22.58	0.405^c^
VT	59	3.64	55	7.04	0.651^b^
Ketones, µmol/L					
Total ketone bodies	58	130.00	60	− 16.98	0.009^c^
Acetoacetic acid	58	35.22	60	− 5.42	0.005^c^
3-Hydroxybutyric acid	58	94.78	60	− 11.57	0.013^c^
Catecholamines—pg/mL					
Adrenaline	58	− 0.07	60	2.17	0.538^b^
Noradrenaline	58	− 5.95	60	36.00	0.343^b^
Dopamine	58	4.41	60	0.02	0.190^c^
Other measurements					
Glycated hemoglobin—%	62	− 0.29	57	0.05	< 0.001^c^
Hematocrit—%	69	1.86	64	− 0.38	< 0.001^b^
BNP—pg/ml	61	− 29.65	55	0.83	0.012^b^
Body weight—kg	68	− 2.40	64	− 0.01	< 0.001^b^
Systolic blood pressure—mmHg	68	− 1.46	63	− 5.97	0.090^b^

^a^*P* values were calculated using a generalized linear model to evaluate for significant differences in interactions between treatment group and period from baseline to week 24, indicating a between-group (empagliflozin group vs. placebo group) difference in the change of each variable over time

^b^*P* values were calculated using Student’s t-test to evaluate for significant differences in interactions between treatment group and period from baseline to week 24, indicating a between-group (empagliflozin group vs. placebo group) difference in the change of each variable over time

^c^*P* values were calculated using Welch’s t-test to evaluate for significant differences in interactions between treatment group and period from baseline to week 24, indicating a between-group (empagliflozin group vs. placebo group) difference in the change of each variable over time

BNP, brain natriuretic peptide; VPC, ventricular premature contraction; VT, ventricular tachycardia

### Other prespecified outcomes and safety

Treatment with empagliflozin was associated with an increase in blood ketones and hematocrit and a decrease in blood BNP and body weight (Table 2). Blood total ketone bodies, acetoacetic acid, and 3-hydroxybutyric acid were increased in the empagliflozin group compared with the placebo group, but blood adrenaline and noradrenaline tended to decrease in the empagliflozin group compared with the placebo group (Table 2). Changes from week 0 to week 24 in glycated hemoglobin, hematocrit, BNP, body weight, and systolic blood pressure in the two groups are shown in Table 2.

A total of 7 adverse events of special interest occurred in 7 patients. These events included 2 cases of liver dysfunction in the empagliflozin group and 1 case each of hypoglycemia, dehydration, genital tract infection, heart failure hospitalization, and inappropriate device discharge in the placebo group. Only the case of dehydration in the placebo group was deemed by the investigators to be related to a study drug.

## Discussion

The EMPA-ICD trial is the first prospective study to demonstrate the antiarrhythmic effects of empagliflozin. In patients with type 2 diabetes treated with ICD/CRT-D, empagliflozin significantly reduced the number of ventricular arrhythmias compared with placebo. Empagliflozin also had a favorable, but not statistically significant, effect on the number of appropriate device discharges and ventricular arrhythmias recorded by Holter monitoring.

Treatment for diabetes has advanced greatly, and the prognosis for patients with diabetes has improved; however, compared with non-diabetic patients, patients with diabetes still have significantly higher total mortality and cardiovascular death [4, 20, 21]. In particular, patients with diabetes are known to be at high risk of sudden death, which is often caused by ventricular arrhythmias [3, 4, 20, 21]. The EMPA-REG study showed that treatment with empagliflozin significantly reduced all-cause and cardiovascular death and also showed a decreasing trend in the risk of sudden death [5]. A retrospective analysis of the DAPA-HF trial showed that dapagliflozin reduced the number of events in the composite endpoint of sudden death, severe ventricular arrhythmia, and cardiac arrest [11]. A nationwide population-based longitudinal cohort study found that treatment with SGLT2 inhibitors reduced total mortality and new onset of supraventricular/ventricular arrhythmias; however, the reduction in the incidence of ventricular arrhythmias was not significant [22]. Some of the studies in the above-mentioned meta-analysis reported that treatment with SGLT2 inhibitors reduced the risk of total mortality and sudden death and/or the frequency of ventricular arrhythmias, whereas others reported no effect [12, 16, 23]. Common problems in these analyses included the lack of arrhythmic event monitoring by the device and the extremely low incidence of such events, so prospective clinical studies are needed to verify the antiarrhythmic effects of SGLT2 inhibitors.

The results of the post hoc analysis of the DAPA-HF trial data generally support the results of this study [11], but it should be noted that all patients in the DAPA-HF trial had systolic dysfunction (LVEF < 40), whereas only about 40% of patients in the EMPA-ICD trial did; however, all patients in the present study had type 2 diabetes and received ICD/CRT-D therapy. As a result, the EMPA-ICD trial differs from the DAPA-HF trial in that it targeted patients with more arrhythmic events (3–4 events per person-24 weeks vs. < 2 events per 100 person-years, respectively). Other differences are that the DAPA-HF trial analyzed data based on adverse event reports, whereas the EMPA-ICD trial compared the number of arrhythmic events before and during treatment in the same patients by evaluating ICD/CRT-D records. Recently, the Ertugliflozin to Reduce Arrhythmic Burden in ICD/CRT Patients (ERASe) study group has initiated a larger and longer randomized controlled trial in patients experiencing more arrhythmic events, with primary endpoints similar to those in the EMPA-ICD study [24]. Results from the ERASe study will clarify whether SGLT2 inhibitors have antiarrhythmic effects.

Several preclinical studies have examined the mechanisms of the antiarrhythmic action of SGLT2 inhibitors [15, 16]. One potential mechanism is that the diuretic and reno-protective effects of SGLT2 inhibitors may improve hemodynamics and thus suppress arrhythmias, and several clinical studies have confirmed the beneficial effect of SGLT2 inhibitors on hemodynamic status [25, 26]. In the present study, the empagliflozin group showed an improvement in BNP and a decrease in body weight compared with the placebo group. A second potential mechanism is that the increase in ketones induced by SGLT2 inhibitors may suppress arrhythmias by improving myocardial energy metabolism and decreasing sympathetic nervous system activity through signaling pathways mediated by plasma membrane receptors [27]. In the present study, a significant increase in ketone bodies was observed in the empagliflozin group, as well as a trend for a decrease in catecholamine concentrations. SGLT2 inhibitors are also thought to increase hematocrit through their protective effects on the proximal tubules and diuretic effects, thereby increasing the oxygen-carrying capacity of myocardial tissue and reducing arrhythmias [28]. Significant increases in hematocrit were also observed in the empagliflozin group in this study. In addition to these hemodynamic, autonomic, and metabolic effects, several direct and indirect antiarrhythmic effects have been suggested for SGLT2 inhibitors. For example, inhibition of late sodium currents [29] and Ca^2+^-dependent activation of Ca^2+^/calmodulin-dependent kinase II and sarcoplasmic reticulum calcium leakage [30] may reduce the incidence of ventricular arrhythmias. Suppression of remodeling, such as ventricular fibrosis and enlargement, may also indirectly help reduce ventricular arrhythmias [31, 32].

In the EMPA-ICD trial, factors associated with the favorable effect of empagliflozin on ventricular arrhythmias included age above 65 years, history of smoking, LVEF of less than 40%, BNP greater than 100 pg/mL, history of ischemic heart disease, eGFR less than 45 mL/min/1.73 m^2^, use of loop diuretics or anti-arrhythmic drugs, and catheter ablation therapy, suggesting an increased benefit of empagliflozin in patients at higher arrhythmic risk (Supplementary Fig. 1). Interestingly, the DAPA-HF trial showed that the beneficial effect of dapagliflozin was further increased in patients with *N*-terminal pro-brain natriuretic peptide levels below the median when compared with patients with levels above the median [11]. Further analysis of such cofounding factors may provide novel insights into mechanisms of the antiarrhythmic effects of SGLT2 inhibitors.

### Limitations

Our trial has some limitations. First, it was affected by the COVID-19 pandemic and was terminated before the planned sample size had been reached, so the study enrolled a relatively small number of patients. Second, the clinically small differences and the lack of clear benefit for more meaningful arrhythmias also are considered as limitations. Although the differences were statistically significant, the absolute value of empagliflozin’s effect in suppressing ventricular arrhythmias was relatively low (1.69 events per 6 months). One reason for this relatively low absolute value may be that a number of the enrolled patients had few or no arrhythmic events at baseline. Therefore, a similar study in patients with a high number of arrhythmic events before treatment may be warranted. Third, because all enrolled patients were Japanese and had type 2 diabetes, the study does not provide information on the antiarrhythmic effect of empagliflozin in other ethnic groups and in non-diabetic patients, which limits the generalizability of the results. Fourth, 60% of the study population had an LVEF greater than 40, and patients enrolled in our trial had different indications for defibrillators and various underlying diseases. Although there was no significant difference between the treatment and placebo groups in terms of patient background, we cannot rule out the possibility that a slight bias may have influenced the study results. Last, this study did not collect detailed records of ventricular arrhythmic events, particularly NSVT duration and heart rate. These factors may affect the pathological significance of NSVT, which was difficult to verify with the results of this study.

## Conclusion

In patients with type 2 diabetes who are treated with ICD/CRT-D, empagliflozin reduces the number of ventricular arrhythmias compared with placebo. The results of the EMPA-ICD trial suggest that empagliflozin may have a beneficial effect on ventricular arrhythmias in patients with type 2 diabetes who receive treatment with ICD/CRT-D.

### Electronic supplementary material

Below is the link to the electronic supplementary material.

Supplementary Material 1.

Supplementary Material 2.

Supplementary Material 3.

Supplementary Material 4.

## Data Availability

Deidentified participant data will be made available on reasonable request 2 years after the date of publication. Requests should be directed to the corresponding author (t.minamino@juntendo.ac.jp). Requestors will be required to sign a data access agreement to ensure the appropriate use of the study data.

## Abbreviations

BNP: Brain natriuretic peptide
CABG: Coronary artery bypass graft
CRT-D: Cardiac resynchronization therapy defibrillator
DAPA-HF: Dapagliflozin and Prevention of Adverse Outcomes in Heart Failure
eGFR: Estimated glomerular filtration rate
EMPA-ICD: Empagliflozin in patients with type 2 diabetes treated with an implantable cardioverter-defibrillator
ERASe: Ertugliflozin to Reduce Arrhythmic Burden in ICD/CRT Patients
GLM: Generalized linear model
ICD: Implantable cardioverter-defibrillator
IQR: Interquartile range
LVEF: Left ventricular ejection fraction
NSVT: Non-sustained ventricular tachycardia
PCI: Percutaneous coronary intervention
SGLT2: Sodium-glucose cotransporter 2
VF: Ventricular fibrillation
VPC: Ventricular premature contractions
VT: Ventricular tachycardia
