# Supplementary material for: Effect of empagliflozin on ventricular arrhythmias in patients with type 2 diabetes treated with an implantable cardioverter-defibrillator: the EMPA-ICD trial
Source: Cardiovasc Diabetol. 2024 Jun 28;23:224. doi: 10.1186/s12933-024-02309-9 (PMC11214255; doi:10.1186/s12933-024-02309-9)
Supplement: Supplementary file 1 — Supplementary Material 1. [file 12933_2024_2309_MOESM1_ESM.pdf]

Supplementary Figure 1. Patient background characteristics that show significant interactions to be potential confounding factors for the effect of empagliflozin on ventricular arrhythmias

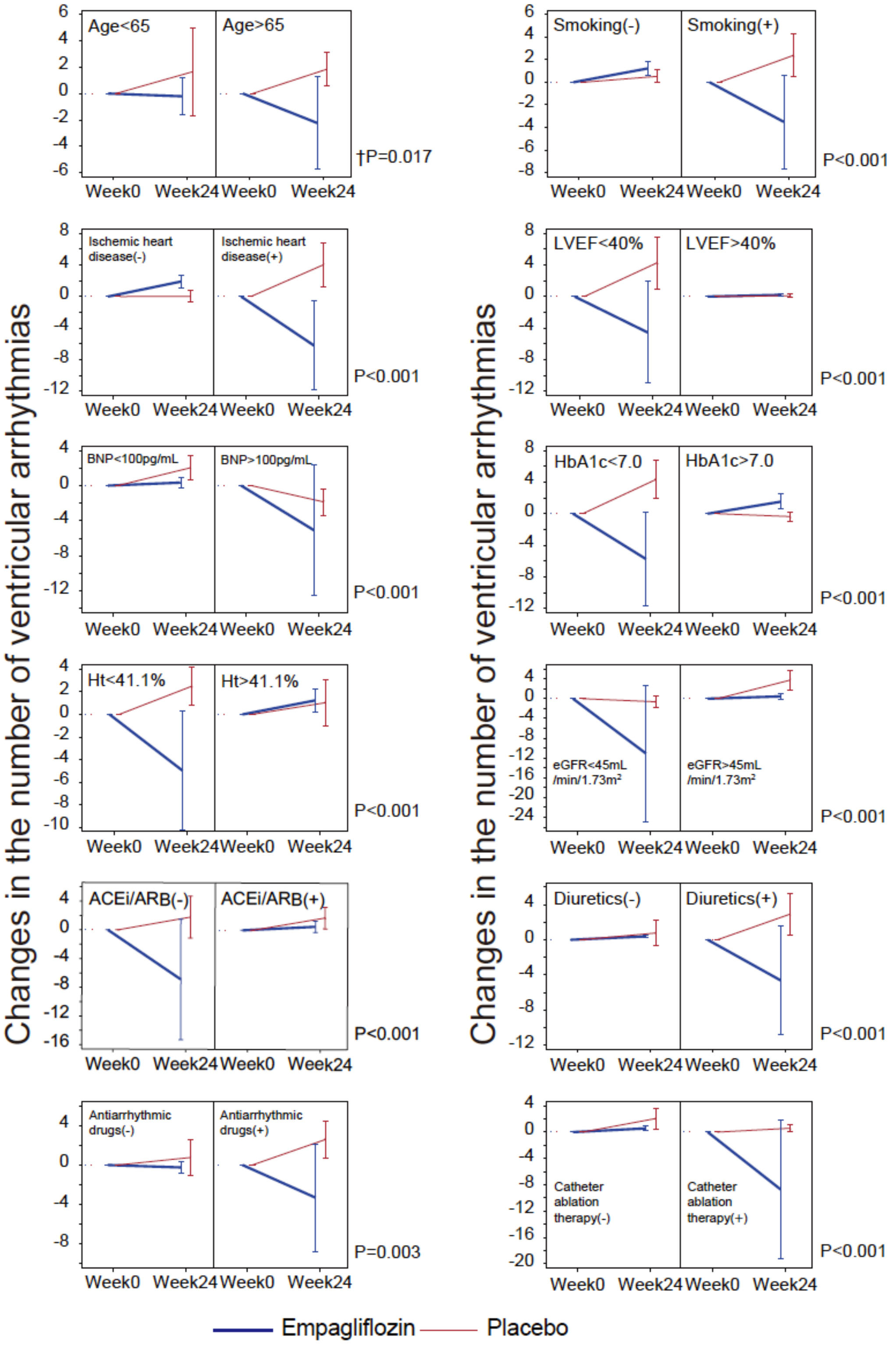

Each line graph with a standard error bar represents the changes in the number of ventricular arrhythmias recorded by an implantable cardioverter-defibrillator or cardiac resynchronization therapy defibrillator from the 24 weeks before treatment (assessed at week 0) and week 24 during treatment (assessed at week 24) in the empagliflozin relative to the placebo group, stratified by background factors. Background factors were dichotomous variables at baseline, and continuous variables were dichotomous at the clinical cutoff or median. ACEi/ARB, angiotensin-converting enzyme inhibitors/angiotensin receptor blockers; BNP, brain natriuretic peptide; eGFR, estimated glomerular filtration rate; Ht, hematocrit; HbA1c, hemoglobin A1c; LVEF, left ventricular ejection fraction.

P values were calculated by the generalized linear model to assess significant differences in interactions between dichotomous background factors and period in the empagliflozin group.
