# Supplementary material for: Effect of empagliflozin on ventricular arrhythmias in patients with type 2 diabetes treated with an implantable cardioverter-defibrillator: the EMPA-ICD trial
Source: Cardiovasc Diabetol. 2024 Jun 28;23:224. doi: 10.1186/s12933-024-02309-9 (PMC11214255; doi:10.1186/s12933-024-02309-9)
Supplement: Supplementary file 2 — Supplementary Material 2. [file 12933_2024_2309_MOESM2_ESM.pdf]

Supplementary Note 1

Table of Contents

EMPA-ICD Trial Committee and Investigators..2

List of the EMPA-ICD investigators...7

EMPA-ICD Trial Committee and Investigators

(1) Principle Investigator

Tohru Minamino, Professor and Chairman, Department of Cardiovascular Biology and Medicine, Juntendo University Graduate School of Medicine, Tokyo , Japan
Part-time Lecturer, Department of Cardiovascular Medicine, Niigata University Graduate School of Medical and Dental Sciences, Niigata, Japan
Research leader, Japan Agency for Medical Research and Development-Core Research for Evolutionary Medical Science and Technology (AMED-CREST), Japan Agency for Medical Research and Development

(2) Study adviser

Yoshifusa Aizawa, Department of Research and Development, Tachikawa General Hospital, Tachikawa Medical Center

(3) Person in charge of supporting research and development programs

Koichi Node, Professor, Department of Cardiovascular Medicine, Saga University

(4) Person who coordinates and manages research

Shinya Fujiki, Department of Cardiovascular Medicine, Niigata University Graduate School of Medical and Dental Sciences

(5) Steering committee

Toyoaki Murohara (Chairperson), Professor, Department of Cardiology, Nagoya University Graduate School of Medicine
Toshihisa Anzai, Professor, Department of Cardiovascular Medicine, Hokkaido University Graduate School of Medicine
Kenji Ando, Medical Director, Cardiovascular Medicine, Kokura Kinen Hospital
Junichi Nitta, Senior Director, Cardiovascular Medicine, Sakakibara Heart Institute
Masaaki Okabe, Director, Tachikawa General Hospital
Ritsushi Kato, Professor, Department of Cardiology, Saitama Medical University International Medical Center
Kazuomi Kario, Professor, Division of Cardiovascular Medicine, Department of Medicine, Jichi Medical University School of Medicine
Kengo Kusano, Director, Department of Cardiovascular Medicine, National Cerebral and Cardiovascular Center

Yusuke Kondo, Associate Professor, Department of Cardiovascular Medicine, Chiba University Graduate School of Medicine
Shingo Sasaki, Associate Professor, Department of Cardiology and Nephrology, Hirosaki University Graduate School of Medicine
Yoshiaki Kubota, Assistant Professor, Department of Cardiovascular Medicine, Nippon Medical School
Morio Shoda, Professor, Department of Cardiology, Tokyo Women's Medical University Hospital
Masaki Ieda, Professor, Department of Cardiology, Faculty of Medicine, University of Tsukuba
Kazuyoshi Takahashi, Director, Department of Cardiovascular Medicine, Niigata City General Hospital
Takashi Kaneshiro, Associate Professor, Department of Cardiovascular Medicine, Fukushima Medical University
Kenichi Tsujita, Professor, Department of Cardiovascular Medicine, Graduate School of Medical Sciences, Kumamoto University
Hirofumi Tomita, Professor, Department of Cardiology and Nephrology, Hirosaki University Graduate School of Medicine
Shigeto Naito, Director, Cardiovascular Medicine, Gunma Prefectural Cardiovascular Center
Shinichi Niwano, Professor, Department of Cardiovascular Medicine, Kitasato University
Tetsuji Miura, Professor, Department of Cardiovascular, Renal and Metabolic Medicine, Sapporo Medical University
Tomio Umemoto, Department of Cardiology, Jichi Medical University Saitama Medical Center
Takeshi Kato, Assistant Professor, Department of Cardiology, Graduate School of Medical Science, Kanazawa University
Yoshihisa Nakagawa, Professor, Department of Cardiovascular Medicine, Shiga University of Medical Science
Koji Maemura, Professor, Department of Cardiovascular Medicine, Nagasaki University Graduate School of Biomedical Sciences
Hiroshi Tada, Professor, Department of Cardiovascular Medicine, Faculty of Medical Sciences, University of Fukui
Masafumi Watanabe, Professor, Department of Cardiology, Pulmonology, and Nephrology, Yamagata University Faculty of Medicine

Masafumi Yano, Professor, Division of Cardiology, Department of Medicine and Clinical Science, Yamaguchi University Graduate School of Medicine
Shingen Owada, Lecturer, Department of Cardiology, Iwate Medical University Hospital
Yugo Yamashita, Assistant Professor, Department of Cardiovascular Medicine, Kyoto University Graduate School of Medicine
Takashi Saigawa, Director, Department of Cardiovascular Medicine, Niigata Prefectural Central Hospital
Hidemori Hayashi, Associate Professor, Department of Cardiovascular Biology and Medicine, Juntendo University Graduate School of Medicine
Takashi Tokano, Associate Professor, Department of Cardiovascular Biology and Medicine, Juntendo University Graduate School of Medicine

(6) Data and safety monitoring board

Naohiko Takahashi (Chairperson), Professor, Department of Cardiovascular Medicine, Oita University
Kojiro Ueki, Director, Diabetes Research Center, Research Institute, National Center for Global Health and Medicine
Yohei Ohno, Associate Professor, Cardiovascular Medicine, Tokai University
Koichiro Kuwahara, Professor, Department of Cardiovascular Medicine, Shinshu University
Motoaki Sano, Associate Professor, Department of Cardiovascular Medicine, Keio University

(7) Event assessment committee

Hiroshi Furushima (Chairperson), Director, Furushima Clinic
Hirotaka Sugiura, Director, Cardiovascular Medicine, Niigata Medical Center
Shinsuke Okada, Director, Cardiovascular Medicine, Niigata Medical Center

(8) Statistical analysis body

Takahiro Tanaka, Specially Appointed Assistant Professor, Clinical Research Quality Control division, Clinical and Translational Research Center, Niigata University Medical and Dental Hospital

(9) Research office

Shinya Fujiki, Department of Cardiovascular Medicine, Niigata University Graduate School of Medical and Dental Sciences

Noriko Kawaharada, Micron, Inc.

(10) Project management body

Kenichi Iijima, Associate Professor, Department of Cardiovascular Biology and Medicine, Juntendo University Graduate School of Medicine

(11) Data management body

Kanae Takahashi, Department of Biostatistics, Hyogo Medical University Hospital

Keiko Ota, Center for Clinical Research and Innovation (CCRI), Osaka Metropolitan University Hospital

(12) Monitoring body

Noriko Kawarada, Micron, Inc.

(13) Audit body

Akiko Shibuya, Micron, Inc.

(14) Central measurement bodies

Measurement of blood ketone body fraction, blood catecholamine concentration, erythropoietin, and reticulocytes:

SRL, Inc.

Measurement of telomere length and P53, P21, and P16:

Goro Katsuumi, Department of Cardiovascular Biology and Medicine, Juntendo University Graduate School of Medicine

Metabolome analysis:

Institute for Advanced Biosciences, Keio University

(15) People responsible for tests

Holter monitoring:

Kenichi Iijima, Associate Professor, Department of Cardiovascular Biology and Medicine, Juntendo University Graduate School of Medicine

Echocardiography:

Takeshi Okubo, Department of Cardiovascular Medicine, Niigata University Graduate School of Medical and Dental Sciences

^{123}I-MIBG myocardial scintigraphy:

Yosuke Horii, Assistant Professor, Department of Radiology and Radiation Oncology,

Niigata University Graduate School of Medical and Dental Sciences

(16) Study drug management body

Hiroyasu Sasahara, Implementation Management division, Clinical and Translational Research Center, Niigata University Medical and Dental Hospital

(17) Study drug allocation body

Hiroyasu Sasahara, Implementation Management division, Clinical and Translational Research Center, Niigata University Medical and Dental Hospital

Hisako Yoshida, Department of Medical Statistics, Osaka Metropolitan University Graduate School of Medicine and Faculty of Medicine

(18) Study contact person

Noriko Kawaharada, Project Management, Micron, Inc.

(19) Research Funder

Nippon Boehringer Ingelheim Co., Ltd

(Some of the research expenses will be borne by Eli Lilly and Company [US].)

List of the EMPA-ICD investigators

Department of Cardiovascular Biology and Medicine, Niigata University Graduate School of Medical and Dental Sciences
Shinya Fujiki, Daisuke Izumi, Kazuyuki Ozaki

Department of Cardiovascular Medicine, Shiga University of Medical Science
Yoshihisa Nakagawa, Tomoya Ozawa

Niigata City General Hospital
Kazuyoshi Takahashi, Yukio Hosaka

Tachikawa General Hospital, Tachikawa Medical Center
Masaaki Okabe, Koichi Fuse

Department of Cardiovascular Biology and Medicine, Juntendo University Graduate School of Medicine
Tohru Minamino, Kenichi Iijima, Hidemori Hayashi, Takashi Tokano, Fuminori Odagiri, Goro Katsu-umi

Department of Cardiovascular Medicine, National Cerebral and Cardiovascular Center
Kengo Kusano, Kenzaburo Nakajima

Department of Cardiology, Iwate Medical University Hospital
Shingen Owada, Yohei Sawa

Department of Cardiovascular Medicine, Chiba University Graduate School of Medicine
Yusuke Kondo, Yoshio Kobayashi

Department of Cardiovascular Medicine, Graduate School of Medical Sciences, Kumamoto University
Kenichi Tsujita, Hisanori Kanazawa

Department of Cardiovascular Medicine, Nippon Medical School
Yoshiaki Kubota, Wataru Shimizu

Department of Cardiology and Nephrology, Hirosaki University Graduate School of Medicine
Hirofumi Tomita, Sasaki Shingo

Department of Cardiovascular Medicine, Hokkaido University Graduate School of Medicine
Toshihisa Anzai, Masaya Watanabe

Department of Cardiology, Tokyo Women's Medical University Hospital
Morio Shoda, Atsushi Suzuki

Department of Cardiology, Pulmonology, and Nephrology, Yamagata University Faculty of Medicine
Masafumi Watanabe, Takanori Arimoto

Department of Cardiology, Nagoya University Graduate School of Medicine
Toyoaki Murohara, Yasuya Inden

Department of Cardiovascular Medicine, Fukushima Medical University
Takashi Kaneshiro, Shinya Yamada

Department of Cardiology, Graduate School of Medical Science, Kanazawa University
Takeshi Kato, Masayuki Takamura

Department of Cardiovascular Medicine, Nagasaki University Graduate School of Biomedical Sciences
Koji Maemura, Shuji Arakawa

Department of Cardiovascular Medicine, Kitasato University
Shinichi Niwano, Naruya Ishizue

Department of Cardiology, Jichi Medical University Saitama Medical Center
Tomio Umemoto, Takeshi Mitsuhashi

Department of Cardiology, Saitama Medical University International Medical Center
Ritsushi Katou, Kenta Tsutsui

Department of Cardiology, Niigata Prefectural Central Hospital
Takashi Saigawa, Tohru Watanabe

Division of Cardiology, Gunma Prefectural Cardiovascular Center
Shigeto Naito, Koki Nakamura

Department of Cardiology, Kokura Memorial Hospital
Kenji Ando, Jun Hirokami

Division of Cardiology, Department of Medicine and Clinical Science, Yamaguchi University Graduate School of Medicine
Masafumi Yano, Masakazu Fukuda

Department of Cardiology, Sakakibara Heart Institute

Junichi Nitta, Jun Umemura

Department of Cardiovascular, Renal and Metabolic Medicine, Sapporo Medical University School of Medicine
Tetsuji Miura, Daigo Nagahara

Department of Cardiovascular Medicine, Kyoto University Graduate School of Medicine
Yugo Yamashita

Division of Cardiovascular Medicine, Department of Medicine, Jichi Medical University School of Medicine
Kazuomi Kario, Tomoyuki Kabutoya

Department of Cardiology, Faculty of Medicine, University of Tsukuba
Masaki Ieda, Akira Kimata

Clinical and Translational Research Center, Niigata University Medical and Dental Hospital,
Takahiro Tanaka, Nobutaka Kitamura

Department of Medial Statistics, Osaka Metropolitan University Graduate, School of Medicine
Hisako Yoshida

Data Management Group, Department of Clinical Research Support, Center for Clinical Research and Innovation, Osaka Metropolitan University Hospital
Keiko Ota

Department of Cardiovascular Medicine, Saga University
Koichi Node
