# Supplementary material for: Effect of empagliflozin on ventricular arrhythmias in patients with type 2 diabetes treated with an implantable cardioverter-defibrillator: the EMPA-ICD trial
Source: Cardiovasc Diabetol. 2024 Jun 28;23:224. doi: 10.1186/s12933-024-02309-9 (PMC11214255; doi:10.1186/s12933-024-02309-9)
Supplement: Supplementary file 3 — Supplementary Material 3. [file 12933_2024_2309_MOESM3_ESM.pdf]

Supplementary Note 2

This supplement contains the following items:

1. Original protocol, final protocol, summary of changes
2. Original statistical analysis plan, final statistical analysis plan with revision history

Placebo-controlled, double-blind study in type 2 diabetes-complicated patients with arrhythmia device implanted to evaluate change in severe arrhythmia after empagliflozin intervention

Comparison of empagliflozin or placebo for prevention of lethal ventricular arrhythmia in type 2 diabetic patients with implantable cardioverter defibrillators

[EMPA-ICD]

Protocol

Ver. 1 .3

Study duration planned: From publication date in jRCT till October 2022

Issued: Oct. 17, 2018 Ver. 1.3

Table of contents

1 : Overview 8
1-1. Study design 8
1-2. Objectives 8
1-3. Patients 8
1-3-1. Inclusion criteria 8
1-4. Treatment 10
1-5. Evaluation endpoints 10
1-5-1. Primary endpoint 10
1-5-2. Secondary endpoints 11
1-5-3. Exploratory evaluation endpoints 11
1-5-4. Safety evaluation items 12
1-6. Study schedule 13
1-7. Target sample size and study duration planned 13
2 : Study background 14
3 : Objective 15
4 : Summary of study drug 15
4-1. Active drug 15
4-2. Placebo 16
5 : Patients 16
5-1. Patients 16
5-2. Inclusion criteria 16
5-3. Exclusion criteria 17
5-4. Discontinuation criteria 18
6 : Informed consent 19
6-1. Procedure to obtain informed consent 19
6-2. In case where information likely to affect participation in the study was obtained 20
7 : Study methods 20
7-1. Study design 20
7-2. Study outline 20
7-3. Target sample size and study duration planned 21
7-4. Patient enrollment and allocation 21
7-5. Blinding 22
7-6. Treatment method 22
7-7. Actions taken for additional treatment 22

７-８. Provisions for handling of study drug (Procedure for management and delivery) 25
７-９. Combination treatment 25
８: Observation and test items 28
８-１. Schedule for observation and tests (Refer to Table 1) 28
８-２. Observation and test items 29
９:　Evaluation endpoints 33
９-１. Primary endpoint 33
９-２. Secondary endpoints 34
９-３. Exploratory evaluation endpoints 35
９-４. Safety evaluation items 36
１０: Measures to be taken for AE onset 36
１０-１. About adverse reaction (AE) 36
１０-２. Duration to collect information on adverse events 37
１０-３. Investigation items of adverse events 37
１０-４. Report of adverse events 39
１１: Data collection 40
１１-１. Completion of case report form (CRF) and report 40
１１-２. Data management 41
１２: Statistical analysis 41
１２-１. Definition of analysis populations 41
１２-１-１. Full analysis set (FAS） 41
１２-１-２. Per protocol set (PPS) 41
１２-１-３. Safety analysis set (SAS) 41
１２-２. Analysis of primary endpoint 41
１２-３. Analysis of secondary, exploratory, and safety endpoints 42
１２-４. Significance level 42
１２-５. Handling of missing data 42
１３: Target sample size and rationales for setting 42
１４: Ethics 44
１４-１. Ethical review 44
１４-２. Protection of personal information 44
１４-３. Compensation for health impairment 44
１４-４. Patient’s benefit and disadvantage 45
１４-５. Patient’s expenses 45
１５: Quality control and assurance 45

１５-１. Source data 45
１５-２. Handling of data and samples 46
１５-３. Record storage 46
１５-４. Monitoring and audit 46
１５-５. Inspection by CRB or regulatory authority 47
１５-６. Control of incompatibility 47
１５-６-１. About incompatibility and serious incompatibility 47
１５-６-２. Duration to collect incompatibility information 47
１５-６-３. Report of incompatibility 47
１５-７. Report to administrator of participating medical institution 49
１５-８. Regular report to CRB 49
１５-９. Regular report to MHLW 49
１５-１０. Management for implementation of double-blind study 49
１６: Study fund and conflict of interest (COI) 50
１６-１. Study fund 50
１６-２. Conflict of interest (COI) 50
１７: Publication of study results and attribution of right 51
１７-１. Registration of clinical study 51
１７-２. Publication and attribution of study results 51
１８: Revision of study protocol 52
１９: Completion, discontinuation, or suspension of study 52
１９-１. Completion of study 52
１９-２. Discontinuation or suspension of study 52
２０: About Certified Review Board (CRB) 52
２１: Study system 53
２２: References 53

Appendices

A. Package insert for empagliflozin
B. Patient information, consent form, consent withdrawal
C. Procedure for special blood tests
D. Procedure for Holter monitoring
E. Procedure for echocardiographic tests
F. Procedure of ^{123}I-MIBG myocardial scintigraphy
G. Procedure to report adverse events
H. SOP at occurrence of incompatibility

I. Procedure for monitoring

J. Procedure for audit

K. Study system

[Abbreviations and definition of terms]

Abbreviation	Spelled-out terms
ADL	activities of daily living
ALT	alanine aminotransferase
AST	aspartate aminotransferase
BMI	body mass index
BNP	brain natriuretic peptide
BUN	blood urea nitrogen
CABG	coronary artery bypass grafting
Cl	chloride
COI	conflict of interest
Cr	creatinine
CRC	clinical research coordinator
CRT-D	cardiac resynchronization therapy-defibrillator
DNA	deoxyribonucleic acid
DPP-4	dipeptidyl peptidase-4
e’	early diastolic mitral annular velocity by tissue doppler echocardiography
EDC	electronic data capture
eGFR	estimated glomerular filtration rate
FAS	full analysis set
FDA	Food and drug administration
GLP-1	Glucagon-like peptide-1
HDL-C	high density lipoprotein cholesterol
HbA1c	hemoglobin A1c
ICD	implantable cardioverter defibrillator
IRR	Incident rate ratio
jRCT	Japan Registry of Clinical Trials
K	Potassium
LDH	lactate dehydrogenase
LDL-C	low density lipoprotein cholesterol
LVEF	left ventricular ejection fraction
^{123}I-MIBG	^{123}I-Meta-iodobenzylguanidine
Na	natrium

Abbreviation	Spelled-out terms
NSVT	non-sustained ventricular tachycardia
NYHA	New York Heart Association
OGTT	oral glucose tolerance test
PCI	percutaneous coronary intervention
PPS	per protocol set
RNA	ribonucleic acid
SAS	safety analysis set
SGLT2	sodium-dependent glucose transporter 2
SU	sulfonylurea
T-Bil	total bilirubin
TC	total cholesterol
TG	triglyceride
peak TRV	Peak Tricuspid regurgitation velocity
UA	uric acid
VF	ventricular fibrillation
VPC	ventricular premature complex
VT	ventricular tachycardia

1 : Overview

1 - 1 . Study design

Investigator-initiated multicenter, prospective, placebo-controlled, randomized, double-blind, parallel group comparison study

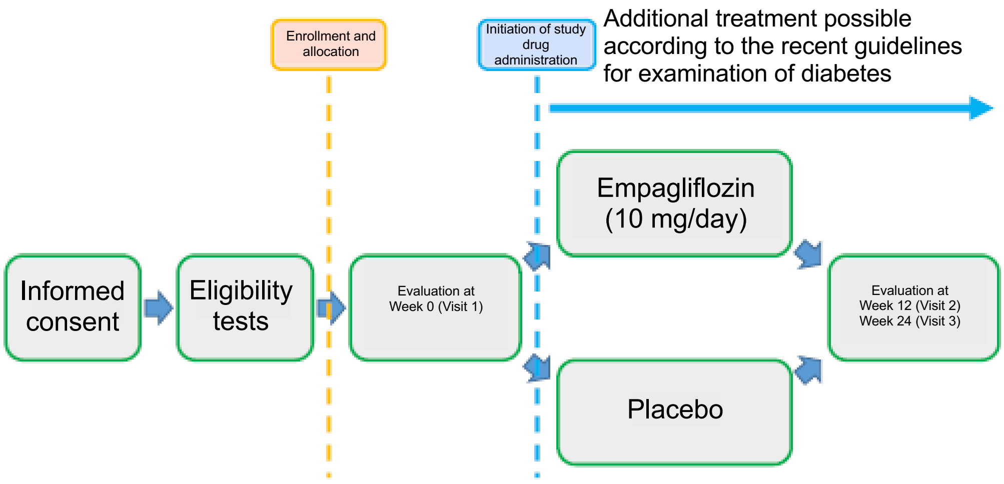

1 - 2 . Objectives

To evaluate whether empagliflozin decreases the number of events attributable to severe arrhythmia in type 2 diabetes-complicated patients with arrhythmia device implanted.

1 - 3 . Patients

Type 2 diabetes-complicated patients（HbA1c ≥ 6.5%）with arrhythmia treatment device (ICD/CRT-D) implanted

1 - 3 - 1 . Inclusion criteria

1) Patients aged 20 years or more when written consent was obtained (regardless of gender).
2) Patients who received ICD or CRT-D implantation surgery more than 24 weeks before the eligibility tests (regardless of implantation purpose: primary or secondary prevention).
3) Patients diagnosed with type 2 diabetes whom Investigator or Subinvestigator judged as possible to be administered empagliflozin (HbA1c should be 6.5 to 10% at the time of eligibility tests, regardless whether drugs other than SGLT2 inhibitor are used to treat diabetes).
4) Patients who themselves granted consent to participation in the study in writing.

1-3-2. Exclusion criteria

1) Patients using SGLT2 inhibitor at the time of eligibility tests.
2) Patients who were administered SGLT2 inhibitor within 24 weeks before the eligibility tests.
3) Patients who have past history of hypersensitivity to empagliflozin (Jardiance® Tablets).
4) Patients who have past history of diabetic ketoacidosis, diabetic coma, or hypoglycemic attack (hypoglycemia or protracted hypoglycemia requiring intervention of a third person) within 24 weeks before the eligibility tests.
5) Patients severely infected, before surgery, or seriously injured at the time of eligibility tests (excluding surgical operation to replace battery cell).
6) Patients receiving examination for type 1 diabetes.
7) Patients with cardiac failure of NYHA Class Ⅳ.
8) Patients with severe renal impairment (eGFR less than 30 mL/min/1.73 m^2 within 3 months after the consent was obtained or receiving renal dialysis).
9) Patients with serious hepatic function disorder (AST or ALT more than 3 times as high as the institutional standards within 3 months after the consent was obtained).
10) Patients with pituitary or adrenal insufficiency.
11) Patients who are under malnutritional or fasting condition, irregularly taking foods, with poor dietary intake or debilitated.
12) Patients with history of excessive alcohol intake.
13) Patients who have gastrointestinal disorder like diarrhea or vomiting at the time of eligibility tests and are likely to be dehydrated.
14) Patients with urinary tract or genital infection at the time of eligibility tests.
15) Patients who are pregnant, likely to be pregnant, or breastfeeding at the time of eligibility tests or want to be pregnant while participating in this study.
16) Patients whose BMI is less than 18.5 kg/m^2 at the time of eligibility tests.
17) Patients in whom the following events were observed to be likely to affect onset of severe arrhythmia within 24 weeks before the eligibility tests: alteration of antiarrhythmic drug, catheter ablation for ventricular arrhythmia, coronary revascularization, open-heart surgery, development of coronary artery disease, stroke or transient ischemic stroke seizure, infection requiring hospitalization, and cardiac failure requiring hospitalization.
18) Patients who are using an arrhythmia device unable to record nonsustained ventricular tachycardia (NSVT).

19) Patients who are complicated by non-remitted malignant tumor at the time of eligibility tests.

20) Patients whom Investigator or Subinvestigator judges as inappropriate to participate in this clinical study.

*See Sections 5-2 and 5-3 for rationales for setting the inclusion and exclusion criteria.

1-4. Treatment

Patients receive either of the following treatments after the eligibility tests, according to case enrollment No. assigned on the Web site for enrollment.

1) Study drug

Empagliflozin 10 mg or placebo

2) Dosage and administration

To orally administer once daily before or after breakfast for 24 weeks

1-5. Evaluation endpoints

1-5-1. Primary endpoint

The number of severe arrhythmia events (NSVT/VT/VF) recorded in the arrhythmia device is evaluated at Week 0 and 24 (or study discontinuation), to calculate the following: Difference in No. of severe arrhythmia events between the empagliflozin and placebo groups.

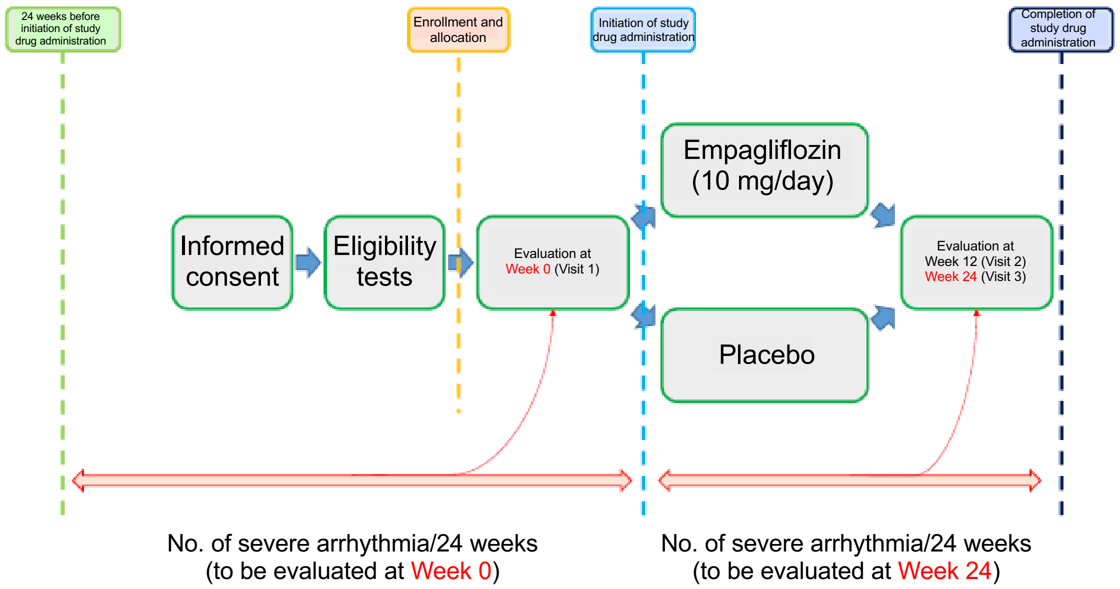

*The retrospective 24-week records are evaluated at Week 0 or 24, as shown above in Figure:

1-5-2. Secondary endpoints

1) The number of severe arrhythmia events (NSVT/VT/VF) and appropriate device operations (anti-tachycardia pacing/shock operation) recorded in the arrhythmia device is evaluated at Week 0 and 24 (or study discontinuation), to calculate the following.

(1-1) Change in the number of severe arrhythmia events

(1-2) Incidence of severe arrhythmia events before and after treatment

(1-3) No. of severe arrhythmia events at Week 24

(1-4) Presence or absence of severe arrhythmia event at Week 24

(1-5) Change rate of No. of appropriate device operations

(1-6) Change in No. of appropriate device operation

(1-7) Incidence of appropriate device operation before and after treatment

(1-8) No. of appropriate device operations at Week 24

(1-9) Presence or absence of appropriate device operation at Week 24

These variables are compared between the empagliflozin and placebo groups.

In addition, the following are evaluated at Week 0 and 24 (or study discontinuation), to compare the change rate, change, and values at Week 24 between the empagliflozin and placebo groups.

2) Holter electrocardiographic tests: Total recording time, total No. of VPC, No. of single VPC, No. of two-consecutive VPC, and No. of ventricular tachycardia.

3) Blood ketone body fraction (acetoacetic acid, 3-hydroxybutyric acid, and total ketone bodies): blood concentration of each ketone body.

4) Blood catecholamine fraction (adrenalin, noradrenalin, and dopamine): blood concentration of catecholamine.

Correlation between difference in No. of severe arrhythmia events and blood ketone body or catecholamine is comparatively evaluated between the empagliflozin and placebo groups.

1-5-3. Exploratory evaluation endpoints

1) No. of atrial fibrillation events recorded in the arrhythmia device and biological monitoring indices are evaluated at Week 0 and 24, to calculate the following.

(1-1) Change rate of No. of atrial fibrillation event

(1-2) Change in No. of atrial fibrillation events

(1-3) Incidence of atrial fibrillation event before and after treatment

(1-4) No. of atrial fibrillation events at Week 24

(1-5) Presence or absence of atrial fibrillation event at Week 24
(1-6) Change rate of biological monitoring index
(1-7) Change in biological monitoring index
(1-8) Biological monitoring index at Week 24

These are compared between the empagliflozin and placebo groups.

In addition, the following are evaluated at Week 0 and 24 (or study discontinuation), to compare the change rate, change, and value at Week 24 between the empagliflozin and placebo groups.

2) NYHA classification of cardiac function
3) Body weight
4) Blood pressure, pulse rate, and body temperature
5) Blood glucose: HbA1c and fasting blood glucose
6) Serum lipid, serum uric acid, and cardiac failure marker: TC, HDL-C, LDL-C, TG, UA, and BNP
7) Renal function: Serum Cr, eGFR (estimated), BUN, Na, K, and Cl
8) Hepatic function: Total protein, albumin, AST, ALT, ALP, T-Bil, and LDH
9) Hematological value: RBC count, WBC count, Hb, Ht, and Plt
10) Erythropoietin
11) Reticulocytes
12) DNA tests: Telomere length and G-tail length
13) RNA tests: P53, P21, and P16
14) Metabolome analysis
15) 12-lead ECG: Pulse rate, PQ interval, QRS interval, and QT interval
16) Systolic and diastolic capacity measured by echocardiography test: LVEF, E wave, A wave, E/A, sep-e', lat-e', E/e', LAVI, peak TRV, and HR
17) ^{123}I-MIBG myocardial scintigraphy-obtained sympathetic activity index: Heart-to-mediastinum ratio (H/M), and washout rate

1-5-4. Safety evaluation items

1) Adverse events and reactions (ARs) which developed during a period from initiation of administration until Week 24 (or study discontinuation).

1-6. Study schedule

Enrollment and allocation → (before visit 1) | Initiation of study drug administration → (after visit 1)

	Before treatment		Treatment period		
	Eligibility test	visit 1	visit 2	visit 3	Study discontinuation*11
	Week -12 to 0	Week 0<br>–	Week 12<br>± 4 weeks	Week 24*10<br>± 4 weeks	
Patient information/Obtaining of consent	○				
Patient background	○				
Interview/Physical examination	○	○	○	○	△
Confirmation of drug compliance			○	○	△
Confirmation of study procedure compliance		○	○	○	△
Confirmation of drugs in combination use	○	○	○	○	△
Height	○				
Body weight/Body temperature	○	○	△	○	△
Blood pressure/Pulse rate*1		○	△	○	△
Hematological test	○*4, 5	○*4	△	○	△
Hematological test (blood glucose/lipids)*2	○*4, 5	○*4	△	○	△
Hematological test (special)*2		○*6, 7, 8		○*7, 8	△*7, 8
12-lead ECG		○*4		○	△
Holter monitoring		○*4		○	△
Echocardiography	○*4	○*4		○	△
^{123}I-MIBG myocardial scintigraphy		△*4, 8		△*8	△*8
Adverse events*3	○	○	○	○	△
Evaluation of arrhythmia device		○*9		○	△

*1 to 11: See Section 8-1 for detail.

1-7. Target sample size and study duration planned

1) Sample size of patients: 210 (105 each for active drug and placebo)

2) Treatment observation: From the disclosure date in jRCT till October 2020 (Deadline of enrollment: April 2020)

3) Study duration: From the disclosure date in jRCT till October 2022

２: Study background

The mortality rate of diabetes-complicated patients with cardiac disorder and the rate of rehospitalization for cardiac failure aggravation are both high, and diabetes is known to worsen prognosis of patients with cardiac disorder.[1] This status similarly occurs in patients with implantable cardioverter defibrillator (ICD) implanted or with cardiac resynchronization therapy-defibrillator (CRT-D) implanted for treatment of arrhythmia, indicating that risky defibrillating operation and ICD-related death risk are increased.[2]

Thus, treatment of diabetes is considered important in cardiac disorder patients implanted with such arrhythmia treatment devices, but the guidelines of the Japanese Circulation Society do not specifically recommend target level of blood glucose and diabetes drugs.[3,4] In current status, the dosing regimen is decided at physician’s discretion.

Sodium-dependent glucose transporter 2 (SGLT2） inhibitor is a hypoglycemic drug with new action mechanism, which selectively and reversibly inhibits glucose resorption by SGLT (expressed at proximal tubular site S1 to 2) at the proximal renal tubules to excrete excessive blood glucose into urine, independently of insulin secretion. In a recent EMPA-REG OUTCOME Study, it was shown that administration of SGLT2 inhibitor empagliflozin significantly suppressed cardiovascular death or vascular events (cerebral infarction and myocardial infarction, etc.).[5] Although the study showed the pleiotropic favorable effects of SGLT2, including hypotensive effects and body weight reduction, the mechanism involved in outcome is not known, yet. We authors focus on increase in blood concentration of ketone body after administration of SGLT2 inhibitor.[6] Ketone body produced mostly in the liver is expected to alleviate oxidative stress or suppress sympathetic nerves in the tissues,[7] and is thus presumed to suppressively control arrhythmia in the heart (Refer to Fig. 1).

The primary objective in the present study is to demonstrate whether SGLT2 inhibitor improves or not the number of arrhythmia events, by evaluating the arrhythmia events in patients implanted with an arrhythmia device before and after intervention. In addition, ketone body and catecholamine are assayed to evaluate their relationship to arrhythmia. Taking it into consideration the EMPA-REG OUTCOME study results suggesting that Ht increase after administration of SGLT2 inhibitor was likely to be related to prognosis improvement, change in reticulocytes and erythropoietin related to those results is also evaluated. The authors also pay attention to change in aging-related substances and evaluate them together.

The present study is expected to contribute to establishment of better therapy for diabetes not only in patients with arrhythmia device implanted but also in diabetes-complicated patients with cardiac disorder.

Figure 1.

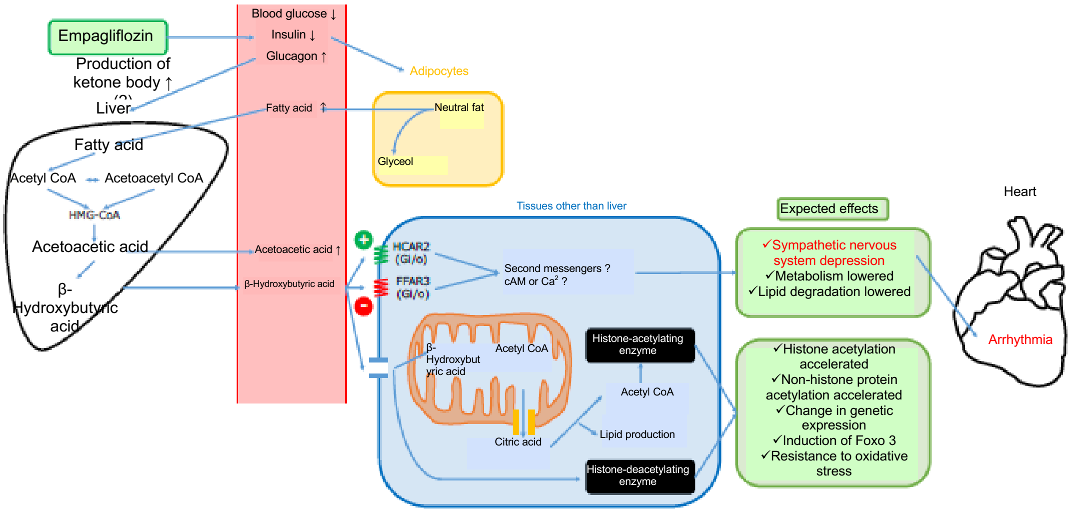

3 : Objective

The principal objective is to demonstrate whether or not empagliflozin improves the number of severe arrhythmia events in type 2 diabetes-complicated patients with arrhythmia device implanted.

Study hypothesis:

Administration of empagliflozin improves the number of severe arrhythmia events in type 2 diabetes-complicated patients with ICD or CRT-D implanted.

4 : Summary of study drug

The study drug shown below, supplied from Boehringer Ingelheim Japan, is used in this study.

4 - 1 . Active drug

Non-proprietary name: Empagliflozin

Chemical name: (1S)-1, 5-Anhydro-1-C-{4-chloro-3-[(4-{[(3S)-oxolan-3-yl]oxy} phenyl)methyl]phenyl}-D-glucitol

Brand name: Jardiance® Tablets 10 mg

Refer to the package insert (Appendix A) for detail.

Investigator or Subinvestigator treats patients according to the updated package insert, keeping in mind the revised revision and referring to Recommendation for Proper Use of

SGLT2 Inhibitors (See Section 7-8).

4-2. Placebo

Placebo is tablet apparently identical to that of active drug, not containing the effective ingredient.

5: Patients

5-1. Patients

Type 2 diabetes-complicated patients (HbA1c ≥ 6.5%) with arrhythmia device (ICD/CRT-D) implanted.

5-2. Inclusion criteria

1) Patients aged 20 years or more when the written consent was obtained (regardless of gender).
2) Patients who received ICD or CRT-D implantation surgery more than 24 weeks before the eligibility tests (regardless of implantation purpose: primary or secondary prevention)
3) Patients diagnosed with type 2 diabetes whom Investigator or Subinvestigator judged as possible to be administered empagliflozin (HbA1c should be between 6.5 and 10% at the time of eligibility tests, regardless whether drugs other than SGLT2 inhibitor are used to treat diabetes).
4) Patients who themselves granted consent to participation in the study in writing.

[Rationales for setting]

1) Adult patients able to grant consent to participation in the study by themselves, without setting the upper limit of age in order to evaluate in the same patient population as in routine clinical practice. Elderly patients should be carefully judged to be included in this study.
2) Patients with arrhythmia device implanted are selected, to be sure to record occurrence of severe arrhythmia.
3) When to newly diagnose with type 2 diabetes, Treatment Guide for Diabetes (2016-2017), ed. The Japan Diabetes Society[8)] is used. In the case where type 2 diabetes was previously diagnosed, the disease is what was diagnosed according to the diagnosis criteria in those days.
4) The setting complies with Clinical Trial Act in Japan.

[Treatment Guide for Diabetes[8)]]

1) Diabetes type is diagnosed when any one of 1 to 4 glycemic state below is confirmed by the initial tests.
   ① Early morning fasting blood glucose level: ≥ 126 mg/dL
   ② Two-hour blood glucose level after 75 g OGTT: ≥ 200 mg/dL
   ③ Casual blood glucose level: ≥ 200 mg/dL
   ④ HbA1c: ≥ 6.5%
2) Diagnosis with diabetes is possible, if diabetes type could be reconfirmed by the tests on another day. However, the blood glucose level is essential to meet the diabetes criteria in either of the initial or repeated tests.
3) If simultaneous measurement of blood glucose and HbA1c is both sure to indicate diabetic type, diabetes can be diagnosed only by the initial tests.
4) Diagnosis with diabetes is possible only in the initial tests, if blood glucose level indicates diabetes type and one of the following symptoms is recognized.
   ① Typical diabetic symptoms such as thirst, polydipsia, polyuria, and weight loss.
   ② Definite diabetic retinopathy

5-3. Exclusion criteria

1) Patients using SGLT2 inhibitor at the time of eligibility tests.
2) Patients who were administered SGLT2 inhibitor within 24 weeks before the eligibility tests.
3) Patients with past history of hypersensitivity to empagliflozin (Jardiance® Tablets).
4) Patients with past history of diabetic ketoacidosis, diabetic coma, or hypoglycemic attack (hypoglycemia or protracted hypoglycemia requiring intervention by a third person) within 24 weeks before the eligibility tests.
5) Patients severely infected at the time of eligibility tests, or seriously injured before surgery (excluding surgical operation to replace battery cell).
6) Patients diagnosed with type 1 diabetes.
7) Patients with cardiac failure of NYHA Class Ⅳ.
8) Patients with severe renal impairment (eGFR less than 30 mL/min/1.73 m^2 within 3 months after consent was obtained or receiving renal dialysis).
9) Patients with serious hepatic function disorder (AST or ALT more than 3 times as high as the institutional standards within 3 months after consent was obtained).
10) Patients with pituitary or adrenal insufficiency.
11) Patients under malnutritional or fasting condition, irregularly taking foods, with poor

dietary intake or debilitated.

12) Patients with history of excessive alcohol intake.

13) Patients who have gastrointestinal disorder like diarrhea or vomiting at the time of eligibility tests and are likely to be dehydrated.

14) Patients with urinary tract or genital infection at the time of eligibility tests.

15) Patients who are pregnant, likely to be pregnant, or breastfeeding at the time of eligibility tests or want to be pregnant while participating in this study.

16) Patients whose BMI is less than 18.5 kg/m^2 at the time of eligibility tests.

17) Patients in whom the following events likely to affect onset of severe arrhythmia were observed within 24 weeks before the eligibility tests: alteration of antiarrhythmic drug, catheter ablation for ventricular arrhythmia, coronary revascularization, open-heart surgery, development of coronary artery disease, stroke or transient ischemic stroke seizure, infection requiring hospitalization, or cardiac failure requiring hospitalization.

18) Patients who are using an arrhythmia device unable to record NSVT.

19) Patients who are complicated by non-remitted malignant tumor at the time of eligibility tests.

20) Patients whom Investigator or Subinvestigator judges as inappropriate to participate in this clinical study.

[Rationales for setting]

1) and 2) Likely to affect the evaluation endpoints.

3) to 15) The package insert requires this drug to be contraindicated or carefully administered. Previous studies[9,10] show the safety in an eGFR range from 30 to 45 mL/min/1.73 m^2.

16) Administration of SGLT2 inhibitor is reported to cause weight loss.

17) and 18) Likely to affect the evaluation endpoints.

19) Patient’s safety is taken into consideration. If remitted at enrollment, the patient is not applicable to the exclusion criteria.

20) Likely to affect the study results, if compliance with the study protocol is concerned because the patient is usually poor in drug adherence or highly likely to miss visit to the hospital, eventually to drop out of the study. Investigator or Subinvestigator makes final decision whether to exclude or not.

5-4. Discontinuation criteria

Should one of the following events occur after enrollment, the study is discontinued. The

observation, test, and evaluation are carried out as soon as possible after study discontinuation.

1) When Investigator or Subinvestigator judges that the study is hard to be continued because the primary disease or complication is aggravated.
2) When Investigator or Subinvestigator judges that the study is hard to be continued because of adverse event onset.
3) When Investigator or Subinvestigator judges that the study is hard to be continued because the patient moved.
4) When Investigator or Subinvestigator judges that the study is hard to be continued because the patient moved to another hospital.
5) Patient requires discontinuation of participation in the study or withdrawal of consent.
6) SGLT2 inhibitor was taken after study drug was administered.
7) When the following events likely to influence onset of severe arrhythmia are observed: alteration of antiarrhythmic drug, catheter ablation for ventricular arrhythmia, coronary revascularization, open-heart surgery, development of coronary artery disease, stroke or transient ischemic stroke seizure, infection requiring hospitalization, or cardiac failure requiring hospitalization.
8) When Investigator or Subinvestigator judges that the study is hard to be continued for reasons other than above.

When to discontinue the study for reasons cited above, the reasons why it was judged hard to continue the study are clearly stated in medical record. In addition, when a patient requires discontinuation of participation in the study or withdrawal of consent, withdrawal of consent in writing is obtained from the patient him-or herself, using “Consent Withdrawal Form” (Appendix B).

6 : Informed consent

When to initiate the study, “Informed Consent” is obtained in advance according to the updated “Clinical Trial Act.”

6-1. Procedure to obtain informed consent

Investigator or Subinvestigator verbally and carefully explains to patients about the study content using the Certified Review Board (CRB)-approved “Patient Information” (Appendix B) prior to confirmation of the eligibility. Upon confirming that patients understood enough the explanation, Investigator or Subinvestigator asks them to participate in the study, and obtains Consent Form (Appendix B) voluntarily completed by patients themselves.

If consented, patients date and sign the completed Consent Form. Investigator or

Subinvestigator who explained also date and sign the form. Even when a clinical research coordinator (CRC) complements the explanation, CRC also dates and signs the form. Patients themselves receive a duplicate of “Patient Information” and “Consent Form.” Investigator retains the original Consent Form at the participating medical institution for 5 years after the study was reported to be discontinued or completed.

6-2. In case where information likely to affect participation in the study was obtained

If information likely to affect patient’s volition to continuously participate in the study was obtained during the study period, Investigator or Subinvestigator immediately explains the information to patients, again to confirm whether patients are willing to continuously participate in the study. Then, patient information is simultaneously revised, reviewed at CRB, and approved at the participating medical institution. Finally, the consent to continuous participation in the study is again obtained from patients in writing.

7: Study methods

7-1. Study design

Investigator-initiated multicenter, prospective, placebo-controlled, randomized, double-blind, parallel group comparison study

7-2. Study outline

Investigator or Subinvestigator obtains the consent from patients, confirms their eligibility, enrolls them, and initiates treatment according to the group allocation. Patients are randomly allocated to the empagliflozin and placebo groups, and administered study drug for 24 weeks under double-blinded condition. Patients participate in the study during a period from obtaining of consent until completion of the treatment observation period (Refer to Fig. 2).

Figure 2.

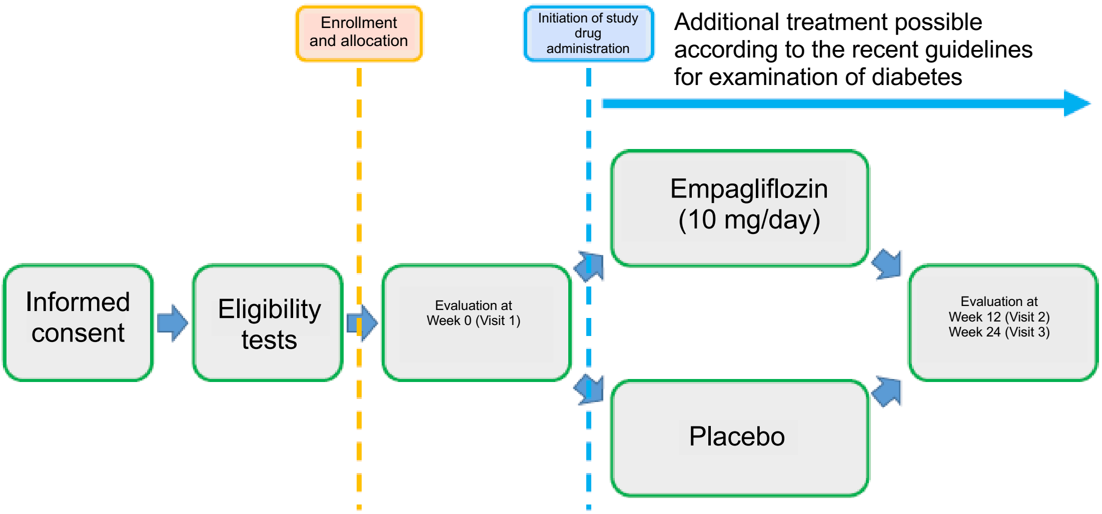

7-3. Target sample size and study duration planned

1) Sample size of patients: 210 (105 each for active drug and placebo)

2) Treatment observation: From the disclosure date in jRCT till October 2020 (Deadline of enrollment: April 2020)

3) Study duration: From the disclosure date in jRCT till October 2022

7-4. Patient enrollment and allocation

Patient enrollment and allocation follows the centralized method. After confirming the patient's eligibility, Investigator or Subinvestigator soon accesses Web site for patient enrollment, and inputs and transmits information necessary for enrollment. The transmitted eligibility is immediately confirmed on the Web site and registered in the database, if eligible, thereafter giving patient enrollment No. and treatment allocation. If ineligible, it is notified to that effect.

Patients are allocated to the treatment groups at a ratio of 1:1 by the stratified allocation method using the following factors. A person responsible for allocation of study drug decides the allocation scheme.

Stratification factors

1) LVEF

2) Age

3) Gender

7-5. Blinding

This study is designed as double-blind. After randomization, patients, Investigator, Subinvestigator, and study staffs involved in this study all can not know the allocation results until completion of data fixation. After completion of the study, the person responsible for allocation of study drug opens the key (unblind) after fixation of the database.

The blinding codes are opened, only under an emergency situation where an appropriate medical treatment is necessary for serious adverse events or the patient’s safety is necessary to be secured. If the key was opened, it is immediately reported to Study-Representing Physician, when participation in the study is discontinued.

7-6. Treatment method

Following allocation of patient enrollment No., study drug is delivered from Niigata University. Investigator or Subinvestigator at each participating medical institution begins treatment after receiving the study drug. Administration of the study drug is continued for 24 weeks.

7-7. Actions taken for additional treatment

When Investigator or Subinvestigator judged that the blood glucose level is insufficiently controlled, it is allowed to use or increase diabetes drugs other than SGLT2 inhibitor, although attention should be carefully paid to adverse reactions such as hypoglycemia or dehydration. The therapeutic purpose follows Treatment Guide for Diabetes (2016-2017)[8)] in Japan. Special attention is necessary to be paid to elderly patients, because the target blood glucose level may be different. After completion of the treatment period, treatment is continued with an optional diabetes drug (Refer to Fig. 3).

Figure 3.

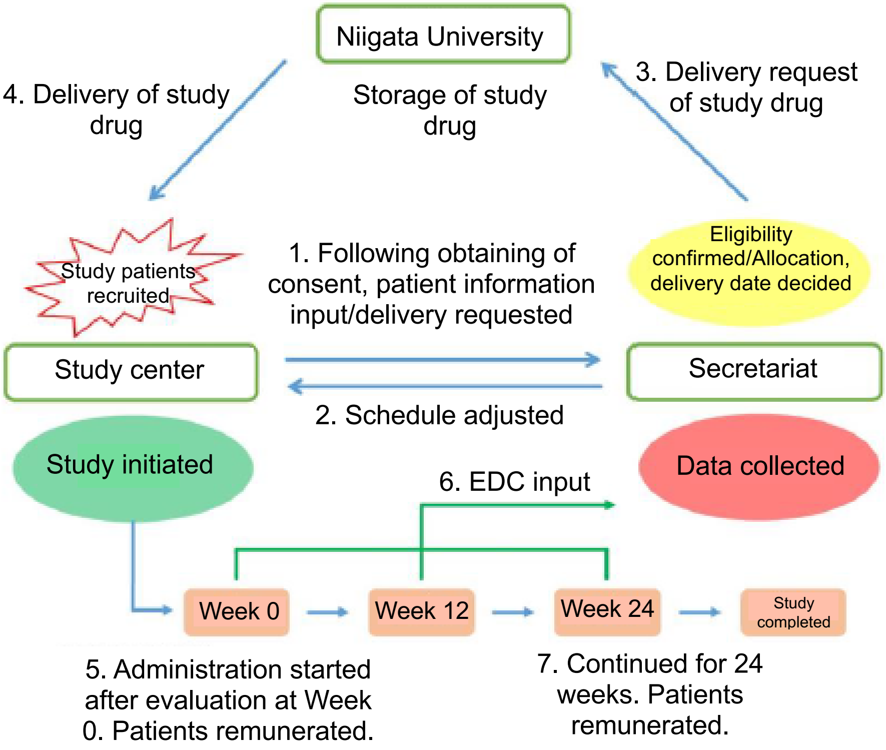

1) Empagliflozin group:

Empagliflozin 10 mg is once daily orally administered before or after breakfast.

2) Placebo group:

Placebo is once daily orally administered before or after breakfast.

In both groups, treatment with empagliflozin is combined with only or some of SU drug, rapid-acting insulin secretagogue, α-glucosidase inhibitor, biguanides, thiazolidine drug, DPP-4 inhibitor, GLP-1 receptor agonist, insulin preparation, or diet/exercise therapy. Selection of treatment is at Investigator or Subinvestigator discretion.

[Therapeutic target for diabetes[8]]

To normalize blood glucose level[Note 1)]: HbA1c less than 6.0%

To prevent complication[Note 2)]: HbA1c less than 7.0%

When hard to intensify treatment[Note 3)]: HbA1c less than 8.0%

The treatment target is individually set up, taking into consideration age, disease duration, organ damage, risky hypoglycemia, or support system.

Note 1): If possible to achieve only by an appropriate diet/exercise therapy, or by drug therapy without causing adverse reactions like hypoglycemia.

Note 2): The target level of HbA1c is less than 7.0% from a viewpoint of prevention of complication. The applicable blood glucose level is roughly less than 130 mg/dL at fasting and less than 180 mg/dL 2 hrs after a meal.

Note 3): When hard to intensify treatment because of adverse reaction like hypoglycemia or other reasons.

[Target level of blood glucose in elderly diabetes patients[8]]

The therapeutic target in elderly patients is individually set up, even taking into consideration cognitive function, basic ADL, instrumental ADL, and comorbidities, in addition to age, disease duration, risky hypoglycemia, and support system. However, attention should be enough paid to severe hypoglycemia which becomes more risky in association of aging.

■ Patient characteristics and health condition.[Note 1)]

Category I: ① Normal cognitive function and ② independence of ADL

Category II: ① Mild cognitive impairment to mild dementia or ② instrumental ADL lowered and independence of basic ADL

Category III: ① Moderate or worse dementia, or ② basic ADL lowered, or ③ many comorbidities and functional disorder

■ When drugs are concerned about induction of severe hypoglycemia (e.g., insulin preparations, SU drugs, glinides) are not used.[Note 2)]

Category I: HbA1c less than 7.0%

Category II: HbA1c less than 7.0%

Category III: HbA1c less than 8.0%

■ When drugs are concerned about induction of severe hypoglycemia (e.g., insulin preparations, SU drugs, glinides) are used.[Note 3)]

Category I: HbA1c less than 7.5% (lower limit: 6.5%) in patients aged 65 years or more and less 75 years, and less than 8.0% (lower limit: 7.0%) in patients aged 75 years or more

Category II: HbA1c less than 8.0% (lower limit: 7.0%)

Category III: HbA1c less than 8.5% (lower limit: 7.5%)

Note 1: Refer to Homepage of The Japan Geriatrics Society (http://www.jpn-geriat-soc.or.jp/) for evaluation of cognitive function and basic ADL (for example, dressing, walking, bathing, and using toilet) and instrumental ADL (for example, shopping, preparing meals, medication management, and monetary control). Under the end-of-life condition, treatments to prevent

marked hyperglycemia and subsequent dehydration and acute complications are prioritized.
Note 2: Even in the case of elderly patients with diabetes, the target level of HbA1c to prevent complications is less than 7.0%. However, if the target can be fulfilled by an appropriate dietary or exercise therapy alone or without adverse reactions of drug therapy, the target HbA1c level is less than 6.0%, and if hard to intensify the treatment, the target is less than 8.0%. In these cases, no lower limit is set up. Under a condition applicable to Category III, if combination use of multiple drugs is concerned about adverse events or social support is not enough for serious comorbidity, the target is allowed to be less than 8.5%.
Note 3: When prevention from onset or progression of complication is preferential in consideration of the morbidity duration of diabetes, the target level or lower limit can be set up for individual elderly patients, while taking measures to prevent severe hypoglycemia. When these drugs have been used since before reaching age of 65 years and the blood glucose is below the target level or lower limit shown in Figure, the current situation is maintained, basically, paying attention to occurrence of severe hypoglycemia. Glinides may be classified into a drug not concerned about severe hypoglycemia, in consideration of type, dose, and blood glucose level

[Important precautions]

Before using diabetes drugs, refer to "Guidelines for medical treatment and its safety in the elderly" ed. The Japan Geriatrics Society. When to use these drugs, be careful with onset of adverse reactions, avoiding use of multiple drugs.

７-８. Provisions for handling of study drug (Procedure for management and delivery)

１) Management of study drug: Study drug (active drug and placebo) is delivered all at once to Clinical and Translational Research Center (CTRC), Niigata University Medical & Dental Hospital, from the study drug supplier, and temporarily stored there. A table of vials numbered for active drug and placebo is together delivered. After the study drug vials were delivered, a person responsible for study drug management packs them by 7 vials/patient (for use in 6 months + extra 1 month), cross-checking with the table.

２) Distribution of study drug: The study drug vials are distributed to each participating medical institution by an independent person responsible for study drug management of CTRC, Niigata University Medical & Dental Hospital.

* Refer to SOP for study drug management for detail.

７-９. Combination treatment

Attention should be paid to use of drugs below in both of the treatment groups during the

study period.

1) Combination use-prohibited drugs: all SGLT2 inhibitors.

2) Precautions for coadministration: Drugs whose interaction with empagliflozin is concerned can be used in combination, but attention should be paid to onset of adverse reactions/complications due to interaction.

- Diabetes drugs: e.g., SU drug, rapid-acting insulin secretagogue, α-glucosidase inhibitor, biguanides, thiazolidine drug, pioglitazone, DPP-4 inhibitor, GLP-1 receptor agonist, and insulin preparations.
- Drugs intensifying action to lower the blood glucose level: e.g., β-blockers, salicylates, and monoamine oxidase inhibitor
- Drugs attenuating action to lower the blood glucose level: e.g., adrenalin, corticosteroid, and thyroid hormone.
- Drugs with diuretic action: e.g., loop diuretics and thiazide diuretics

Should a combination use-prohibited drug be used, a measure is taken according to the procedure for “Control of incompatibility (Section 15-6).” To fulfill the study, refer to the updated “Recommendation for Proper Use of SGLT2 Inhibitors.”

Recommendation for Proper Use of SGLT2 Inhibitors:

１. To use in combination with insulin secretagogues like insulin and SU drug, their dose is reduced, paying full attention to hypoglycemia. Patients should be of course educated enough about hypoglycemia.

２. Administration should be careful, if patients are aged 75 years or more or aged 65 to 74 years with geriatric syndrome (sarcopenia, cognitive function lowered, or ADL decreased).

３. An enough measure should be taken for prevention of dehydration, even including explanation to patients. If a diuretic is used in combination, attention should be paid specifically to dehydration.

４. Drugs should be absolutely withdrawn when food intake is not enough because of pyrexia, diarrhea, or vomiting or because of inappetence (sick day).

５. If accompanied by systemic malaise, nausea, vomiting, or weight loss, blood ketone body should be checked: ketoacidosis is likely even though the blood glucose level looks normal.

６. Should cutaneous symptoms like erythema suspected of drug eruption appear, administration should be immediately discontinued, to consult with Department of Dermatology. Adverse reactions should be sure to be reported.

7. To discover urinary tract infection/genital infection, patients should be appropriately interviewed. It is recommended to use questionnaire while interviewing. If discovered, consult with Department of Urology or Gynecology.

SGLT2 Inhibitor Proper Use Committee, The Japan Diabetes Society, revised May 12, 2016.
*Refer to the updated package insert or recommendation by the society, when to follow up.

8 : Observation and test items

8 - 1 . Schedule for observation and tests (Refer to Table 1)

Table 1:

	Enrollment and allocation	Initiation of study drug administration			
	Before treatment		Treatment period		
	Eligibility test	visit 1	visit 2	visit 3	Study discontinuation*11
	Week -12 to 0	Week 0	Week 12	Week 24*10	
		–	± 4 weeks	± 4 weeks	
Patient information/Obtaining of consent	○				
Patient background	○				
Interview/Physical examination	○	○	○	○	△
Confirmation of drug compliance			○	○	△
Confirmation of study procedure compliance		○	○	○	△
Confirmation of drugs in combination use	○	○	○	○	△
Height	○				
Body weight/Body temperature	○	○	△	○	△
Blood pressure/Pulse rate*1		○	△	○	△
Hematological test	○*4, 5	○*4	△	○	△
Hematological test (blood glucose/lipids)*2	○*4, 5	○*4	△	○	△
Hematological test (special)*2		○*6, 7, 8		○*7, 8	△*7, 8
12-lead ECG		○*4		○	△
Holter monitoring		○*4		○	△
Echocardiography	○*4	○*4		○	△
^{123}I-MIBG myocardial scintigraphy		△*4, 8		△*8	△*8
Adverse events*3	○	○	○	○	△
Evaluation of arrhythmia device		○*9		○	△

○, Essential; △, Optional

*1: Blood pressure/pulse rate is measured in sitting position after resting for more than 5 min as a general rule.

*2: Fasting blood is sampled after resting in supine position on the bed for 30 min as much as possible. In this study, fasting blood sampling is defined as below. If not sampled while fasting and resting, it should be recorded to that effect.

[When blood is sampled before noon]
Blood sampling more than 10 hrs after the last meal: Blood is sampled without taking meal after dinner the day before (no breakfast on the day).
[When blood is sampled after noon]
Blood sampling more than 5 hrs after the last meal: Blood is sampled without taking meal after breakfast on the day (no lunch on the day).

*3: Should adverse event develop, the rationales for judgment (e.g., test value) should be reported.

*4: The test results obtained within 12 weeks before the tests at Week 40 are can be utilized, even before the consent was obtained. The echocardiographic results at the time of eligibility tests are used for allocation to treatment group.

*5: AST, ALT, eGFR, and HbA1c are included in the eligibility tests.

*6: The test results obtained after obtaining consent to Week 0 can be utilized.

*7: Special blood sample volume is as follows: 5 mL for ketone body fraction and erythropoietin test; 7 mL for fraction test for catecholamine; 2 mL for reticulocyte sampling; 10 mL for DNA tests (telomere length and G-tail length): 5 mL for RNA tests (P53, P21, and P16); and 5 mL for metabolome analysis. In the DNA or RNA tests, genes categorized into personal information are not analyzed.

*8: Some of blood samplings for DNA tests (telomere length and G-tail length), RNA tests (P53, P21, and P16), and metabolome analysis, and ^{123}I-MIBG myocardial scintigraphy are optional.

*9: If arrhythmia device was evaluated during a period from consent obtaining until Week 0, the results can be utilized, including even the evaluation results obtained by using the remote monitoring system.

*10: The test results evaluated within 4 weeks before and after Week 24 can be utilized. However, the arrhythmia device is checked at Week 24, and correctly recorded, including even the evaluation results obtained by using the remote monitoring system.

*11: When the study was discontinued, the tests are carried promptly out as much as possible.

8-2. Observation and test items

Investigator or Subinvestigator records the following observation and test results in Case Report Form (CRF). In this study, electronic data capture (EDC) is used to report to Data Center. The transmission data do not include information able to identify the patient (Refer to Section 11. Data collection).

1) Patient background at the time of eligibility test.

The following are investigated.

Gender, age, smoking history, alcohol-drinking history, lethal arrhythmia which caused implantation with arrhythmia device (ventricular fibrillation, monomorphic ventricular tachycardia, polymorphic ventricular tachycardia, and non-sustained ventricular tachycardia) and underlying diseases (ischemic heart disease, cardiac valve disease, dilated cardiomyopathy, hypertrophic cardiomyopathy, restrictive cardiomyopathy, arrhythmogenic right ventricular cardiomyopathy, left ventricular non-compaction, mitochondrial cardiomyopathy, other cardiomyopathy, Brugada syndrome, long QT syndrome, and idiopathic ventricular fibrillation, etc.), maker and type of arrhythmia treatment device, comorbidity and past history (atrial fibrillation, hypertension, dyslipidemia, cerebral infarction or cerebral hemorrhage), oral drugs (all).

Past history of non-drug treatment: PCI, CABG, cardiac valvulopathy surgery, and catheter ablation, etc.

2) Interview and physical examination: at the time of eligibility test, and Week 0, 12, and 24 (or study discontinuation).

Interviewed about to judge NYHA class.

3) Confirmation of drug compliance: at Week 12 and 24 (or study discontinuation).

If forgotten to take study drug, input EDC to that effect.

4) Confirmation of study procedure compliance in the protocol: at Week 0, 12, and 24 (or study discontinuation).

To check whether the tests and treatment adhere to the study protocol.

5) Confirmation of drugs in combination use: at Week 0, 12, and 24 (or study discontinuation).

To confirm whether the drug is prohibited to be used in combination or cautioned to be coadministered. In addition, all drugs orally taken at every time point are inspected for the type and dose (for study compliance and safety evaluation).

6) Height, body weight, and body temperature: at the time of eligibility test and Week 0 and 24 (or study discontinuation) (*Optional at Week 12).

Height, body weight, and body temperature in the examination room are measured at visit to the hospital (at fasting). At Week 0, 12, and 24 (or study discontinuation), only body weight is measured. Height is measured only at the time of eligibility tests.

7) Blood pressure and pulse rate: at Week 0 and 24 (or study discontinuation) (*Optional at Week 12).

Blood pressure (SBP/DBP) and pulse rate are measured in sitting position after

resting for more than 5 min, as a general rule.

8) Hematological tests: at the time of eligibility tests and Week 0 and 24 (or study discontinuation) (*Optional at Week 12).

Hematological (RBC count, WBC count, Hb, Ht, and Plt), blood biochemical (total protein, albumin, AST, ALT, ALP, T-Bil, LDH, BUN, Na, K, Cl, uric acid, Cr, and estimated eGFR), and BNP.

The eligibility tests include AST, ALT, and eGFR.

9) Hematological tests (blood sugar and lipid): at the time of the eligibility tests and Week 0 and 24 (or study discontinuation) (*Optional at Week 12)

Blood glucose tests: fasting blood glucose and HbA1c

Lipid tests: TG, TC, HDL-C, and LDL-C

HbA1c is tested for the eligibility.

10) Hematological tests (special): at Week 0 and 24 (or study discontinuation) （*Blood sampling for DNA tests (telomere length and G-tail length), RNA tests (P53, P21, and P16), and metabolome analysis are optional）

Blood ketone body fraction: acetoacetic acid, 3-hydroxybutyric acid, and total ketone bodies

Blood catecholamine fraction: adrenalin, noradrenalin, and dopamine

Erythropoietin and reticulocytes

DNA tests: Telomere length and G-tail length

RNA tests: P53, P21, and P16

Metabolome analysis

Refer to Appendix C (Procedure for special blood tests) for test procedure in detail.

*Blood sample volume: 8) + 9) = 17 mL and 10) = 34 mL.

11) 12-lead ECG tests: at Week 0 and 24 (or study discontinuation)

Heart rate, rhythm, PQ interval, QRS interval and QT interval

12) Holter monitoring: at Week 0 and 24 (or study discontinuation)

Total recoding time, total No. of VPC, No. of single VPC, No. of two-consecutive VPC, and No. of ventricular tachycardia

Electrocardiogram is analyzed by cardiologists. Refer to Appendix D (Procedure for Holter monitoring) for the test procedure in detail.

13) Echocardiographic tests: at the time of eligibility tests and Week 0 and 24 (or study discontinuation)

Systolic capacity (LVEF) and diastolic capacity (LVEF, E wave, A wave, E/A, sep-e’, lat-e’, E/e’, LAVI, and peak TRV) are measured: Biplane disk summation method (modified Simpson method) is recommended. For e’, both sep-e’ and lat-e’ are

measured. For LAVI, LAV is first measured and corrected by body surface area at each time point. HR at measurement time is also reported. Refer to Appendix E (Procedure for echocardiographic tests) for the test procedure.

14) ^{123}I-MIBG myocardial scintigraphy tests: at Week 0 and 24 (or study discontinuation) (*Optional).

Front planar image is used to calculate the H/M ratio and washout rate. For the test methods, refer to Appendix F (Procedure of ^{123}I-MIBG myocardial scintigraphy).

15) Adverse events: at the time of eligibility tests and Week 0, 12, and 24 (or study discontinuation).

The presence or absence of symptoms applicable to adverse events and findings are confirmed at the time of examinations. For definition of these and reporting procedure, refer to Appendix G (Procedure to report adverse events).

16) Evaluation of arrhythmia device: at Week 0 and 24 (or study discontinuation).

Week 0: During a period of 24 weeks before Week 0, severe arrhythmia (NSVT/VT/VF), No. of events to appropriately treat severe arrhythmia (anti-tachycardia pacing/shock operation), and biological monitoring indicators are confirmed. In the case where atrial lead is inserted, the number of atrial fibrillations events is also confirmed.

Week 24: Severe arrhythmia (NSVT/VT/VF), No. of events to appropriately treat severe arrhythmia (anti-tachycardia pacing/shock operation), and biological monitoring indicators are confirmed during a period of 24 weeks after intervention. In the case where atrial lead is inserted, the number of atrial fibrillations is also confirmed.

If the setting was changed, what was changed is input in EDC, including the reasons.

9 : Evaluation endpoints

9 - 1 . Primary endpoint

The number of severe arrhythmia events (NSVT/VT/VF) recorded in the arrhythmia device is evaluated at Week 0 and 24 (or discontinuation), to calculate the following: Difference in No. of severe arrhythmia events between the empagliflozin and placebo groups.

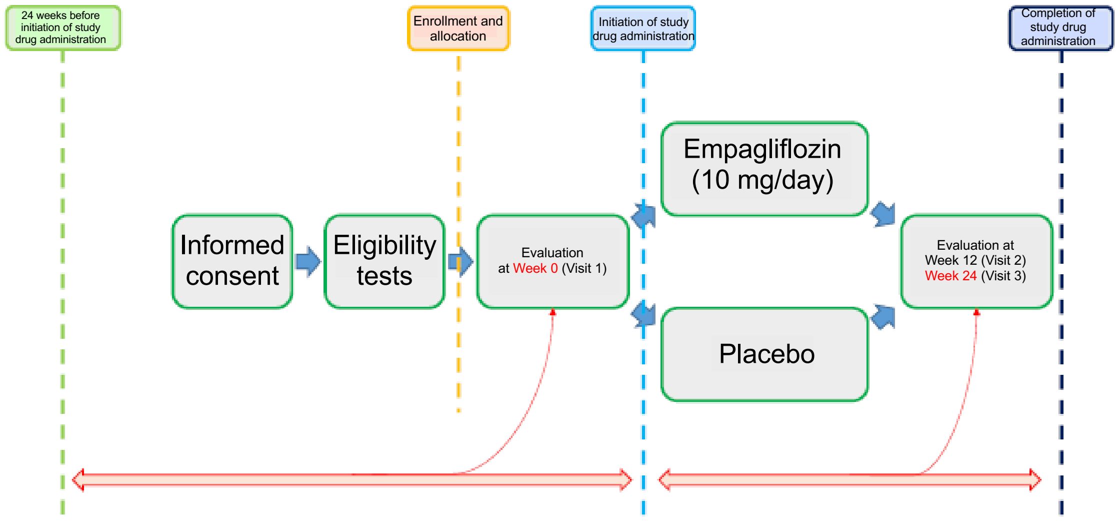

[Severe arrhythmia]

Severe arrhythmia and its appropriate treatment are confirmed by probing information recorded in the arrhythmia device through the remote monitoring system or by programmer's directly retrieving. Actually, when intracardiac ECG is checked to differentially diagnose the type of severe arrhythmia and to treat, type of treatment and appropriate or inappropriate device operation are judged. Each type of arrhythmia is defined as bloew.[7)]

✓Nonsustained ventricular tachycardia (NSVT): Among arrhythmia cases auto diagnosed as NSVT according to the auto diagnosis standards of the device used for individual patients, what cardiologists judges the auto diagnosis as correct. In general, the cases where arrhythmia was sustained more than 5-consecutive VPC and disappeared by ten-consecutive VPC, without being treated by ICD, are auto diagnosed as NSVT in many cases.

✓Ventricular tachycardia (VT)/ventricular fibrillation (VF): Among arrythmia cases auto diagnosed as VT/VF according to the auto diagnosis standards of the device used for individual patients, what cardiologists judges the auto diagnosis as correct.

[Appropriate treatment]

Only when above lethal arrhythmia was treated, meeting the respectively set treatment standards, the treatment is judged as appropriate. When non-lethal arrhythmia (atrial fibrillation or supraventricular tachycardia) was mistakenly treated, the treatment is judged as inappropriate treatment.

[Setting of arrhythmia device]

It is highly likely to disadvantage patients to make identical the setting of arrhythmia monitoring and treatment, the setting is adjusted to individual cases and decided by cardiologists. During the study period, the device setting is not immoderately changed, but can be changed only when a cardiologist in charge of the treatment judges as necessary to change.

[Evaluation of arrhythmia event]

Event Assessment Committee is externally set up to individually evaluate the arrhythmia events data, whether to be appropriate or not, and the results are reported to Study-Representing Physician.

9-2. Secondary endpoints

1) No. of severe arrhythmia events (NSVT/VT/VF) and appropriate device operations (anti-tachycardia pacing/shock operation) recorded in the device are evaluated at Week 0 and 24 (or study discontinuation), to calculate the following.

(1-1) Change in the number of severe arrhythmia events

(1-2) Incidence of severe arrhythmia event before and after treatment

(1-3) No. of severe arrhythmia events at Week 24

(1-4) Presence or absence of severe arrhythmia events at Week 24

(1-5) Change rate of No. of appropriate device operations

(1-6) Change in No. of appropriate device operation

(1-7) Incidence of appropriate device operation before and after treatment

(1-8) No. of appropriate device operations at Week 24

(1-9) Presence or absence of appropriate device operation at Week 24

These variables are compared between the empagliflozin and placebo groups.

In addition, the following are evaluated at Week 0 and 24 (or study discontinuation), to compare the change rate, change, and values at Week 24 between the empagliflozin and placebo groups.

2) Holter monitoring: Total recording time, total No. of VPC, No. of single VPC, No. of two-consecutive VPC, and No. of ventricular tachycardia.

3) Blood ketone body fraction (acetoacetic acid, 3-hydroxybutyric acid, and total ketone bodies): Blood concentration of each ketone body.

4) Blood catecholamine fraction (adrenalin, noradrenalin, and dopamine): Blood concentration of each catecholamine.

The correlation between difference in No. of severe arrhythmia events and blood ketone body or catecholamine is evaluated in comparison between the empagliflozin and placebo groups.

9-3. Exploratory evaluation endpoints

1) The number of atrial fibrillation events and biological monitoring index recorded in the arrhythmia device is evaluated at Week 0 and 24, to calculate the following.

(1-1) Change rate of No. of atrial fibrillation event

(1-2) Change in No. of atrial fibrillation events

(1-3) Incidence of atrial fibrillation event before and after treatment

(1-4) No. of atrial fibrillation events at Week 24

(1-5) Presence or absence of atrial fibrillation event at Week 24

(1-6) Change rate of biological monitoring index

(1-7) Change in biological monitoring index

(1-8) Biological monitoring index at Week 24

These are compared between the empagliflozin and placebo groups.

In addition, the following items are evaluated at Week 0 and 24 (or study discontinuation), to compare the change rate, change, and value at Week 24 between the empagliflozin and placebo groups.

2) NYHA classification of cardiac function

3) Body weight

4) Blood pressure, pulse rate, and body temperature
5) Blood glucose: HbA1c and fasting blood glucose
6) Serum lipid, serum uric acid, and cardiac failure marker: TC, HDL-C, LDL-C, TG, UA, and BNP
7) Renal function: Serum Cr, eGFR (estimated), BUN, Na, K, and Cl
8) Hepatic function: Total protein, albumin, AST, ALT, ALP, T-Bil, and LDH
9) Hematological value: RBC count, WBC count, Hb, Ht, and Plt
10) Erythropoietin
11) Reticulocytes
12) DNA tests: Telomere length and G-tail length
13) RNA tests: P53, P21, and P16
14) Metabolome analysis
15) 12-lead ECG: Pulse rate, PQ interval, QRS interval, and QT interval
16) Systolic and diastolic capacity measured by echocardiographic test: LVEF, E wave, A wave, E/A, sep-e’, lat-e’, E/e’, LAVI, peak TRV, and HR
17) ^{123}I-MIBG myocardial scintigraphy-obtained sympathetic activity index: Heart-to-mediastinum ratio (H/M), and washout rate

９-４. Safety evaluation items

1) Adverse events and adverse reactions which developed during a period from the day of consent obtained until Week 24 (or study discontinuation).

１０: Measures to be taken for AE onset

１０-１. About adverse reaction (AE)

An adverse event is defined to be any untoward medical occurrence in patients administered a study drug (including laboratory abnormalities), which does not necessarily have to have a causal relationship with this study. Of such adverse events, those whose causal relationship to the present study cannot be ruled out are called "disease or the like." For onset of any adverse event, Investigator or Subinvestigator promptly takes appropriate measures to treat them, to secure the patient’s safety. In addition, when administration of study drug was discontinued or treatment was essential for adverse events, Investigator or Subinvestigator tells patients to that effect. For measures to be taken adverse events, refer to Appendix G (Procedure to report adverse events).

[Adverse reactions of empagliflozin expected from the package insert] (Refer to Appendix A)
Severe adverse reaction: hypoglycemia, dehydration, ketoacidosis, pyelonephritis, and

sepsis
Other adverse reactions: Infections (cystitis, urinary tract infection, asymptomatic bacteriuria, vulvovaginal candidiasis, trichomoniasis, bacterial vaginitis, vulvovaginitis, and Vaginal moniliasis); Reproductive disorders (balanitis, genital pruritus, balanoposthitis, vulvovaginal discomfort, and vulvovaginal pruritus); Metabolism and nutrition disorders (hyperlipidemia and fluid volume decreased); Blood and lymphatic system disorders (hemoconcentration); Nerve disorders (dizziness and dysgeusia); Gastrointestinal disorders (constipation and abdominal distention); Skin and subcutaneous tissue disorders (rash, pruritus, and urticaria); Renal and urinary disorders (pollakiuria, polyuria, urine output increased, and dysuria); General disorders (thirst and feeling hungry), and Investigations (weight decreased, urine ketone body present, and blood ketone body present)

According to the guidance of US Food and Drug Administration (FDA) (dated August 29, 2018), attention is paid even to onset of perineal necrotizing fasciitis (Fournier's gangrene) as a severe adverse reaction.

１０-２. Duration to collect information on adverse events

Adverse events are collected, which developed during a period from the day of consent obtained until Week 24 (or study discontinuation). Any onset of disease or the like is followed up until the outcome is established

１０-３. Investigation items of adverse events

Investigator or Subinvestigator investigates development of adverse events for the following items.

1) Name of adverse event and onset date
2) Seriousness
3) Specifically noteworthy adverse events
4) Treatment and outcome, and confirmation date of outcome
5) Causal relationship to the study
6) Administration status of study drug (to be continued or discontinued)

[Definition of serious adverse event]

① that results in death,
② that is life threatening,
③ that requires hospitalization for treatment or prolongation of existing hospitalization,
④ that results in persistent or significant disability or incapacity,
⑤ that results in a congenital anomaly, and

⑥ Other events or reactions considered medically significant

A wording of “Considered medically significant” in No. 6 above refers to an event which is appropriately medically judged as endangering the patient, requiring medical or surgical treatment so as not to lead to serious adverse events defined in No.1 to 5 above. Patients may be hospitalized during the study period for study management or social reasons (ex. because of the day to implement fluid infusion, or not day trip treatment but hospitalization necessary: the hospital is far away to visit). This case of hospitalization or other cases where hospitalization was scheduled at initiation of the study are already reported by the source data at the time of eligibility tests. Therefore, these cases are not necessary to be reported as serious adverse event, if conduced as scheduled.

[Definition of specifically noteworthy adverse event]

① Liver disorder: AST, ALT, and T-Bil increased over the standards set up in this study.
② Kidney function decreased: Cr increased over the standard set up in this study.
③ Acidosis: Metabolic acidosis, ketoacidosis, and diabetic acidosis.
④ Leg amputation-associated events: Amputation, disarticulation, and autoamputation.
⑤ Perineal necrotizing fasciitis (Fournier's gangrene)

[Outcome]

Outcome is judged by 6 categories: “Recovered, Recovering, Not recovered, With sequelae, Death, and Unknown.” In the cases of Recovered, Recovering, or Death, the confirmation day of outcome is set as the day when the outcome was actually recognized. In the case where the follow-up is terminated because the outcome is Not recovered or Unknown, the confirmation day of outcome refers to the day when Investigator or Subinvestigator last confirmed the outcome, and the reasons for termination of follow-up or Unknown are filled out in the comment space.

[Causal relationship to study]

The causal relationship to study is judged by the following two categories. When judged as “Able to be ruled out,” the reason is filled out in the comment space.

① Causal relationship can be ruled out: In the cases where time relationship between adverse event and study is unreasonable, or where the cause of adverse event onset can be explained even by medical relevance other than the study.
② Causal relationship cannot be ruled out: In the cases not applicable to the above

cases, adverse events whose causal relationship to the study cannot be ruled out are set as "diseases or the likes" pursuant to the Clinical Trials Act.

１０-４. Report of adverse events

(Refer to Appendix G (Procedure to report adverse events) for detail)

When onset of serious adverse event was recognized, Investigator or Subinvestigator promptly reports to Study-Representing Physician in writing. In addition, adverse events whose causal relationship to the study cannot be ruled out (diseases or the likes) are reported even to administrator of the participating medical institution according to the Clinical Trials Act. Of these, serious cases (serious diseases or the likes) are reported even to CRB, according to the provisions. In addition, communication to cooperate with the reports based on “Drug and Medical Device Safety Information Reporting System” based on “Act on Securing Quality, Efficacy and Safety of Products Including Pharmaceuticals and Medical Devices” and the spontaneous report by the manufacturing/marketing business license holder should be appropriately executed according to the provision of each participating medical institution. The methods for reporting are summarized as below.

1) Expedited (alert) report

In this study, serious adverse events and specifically noteworthy adverse events are subjected to expedited report. When onset of relevant adverse event was recognized, Investigator or Subinvestigator immediately fills out Adverse Event Report Form as predetermined, transmits to Study Secretariat by FAX at 03-6262-2815, to input through EDC.

2) Routine report

In the cases where nonserious adverse events shown in “Section 10-3. Investigation items of adverse events” were recognized, Investigator or Subinvestigator inputs through EDC. During the study period, the outcome of relevant events is followed up as much as possible to report.

3) Report to administrator of the participating medical institution

Investigator and Subinvestigator share information on adverse events, and report the adverse events whose causal relationship to the study cannot be ruled out (diseases or the likes) even to administrator of the participating medical institution.

4) Report to Independent Data Monitoring Committee (IDMC)

Upon receiving an expedited report on adverse event, Study Secretariat promptly reports to IDMC in writing, simultaneously asking to review the appropriateness of measures taken for the relevant adverse event. The IDMC reviews the report, and reports the future responses including case handling or possible study continuation

to Study-Representing Physician in writing

5) Report to CRB

When considered an adverse event to be serious and unable to be ruled out of the causal relationship to the study (serious disease or the like), Study-Representing Physician reports to CRB. In addition, Study-Representing Physician reports a nonserious adverse event to CRB, if it is specially noteworthy as compared to the cases in common clinical practice, even though unable to rule out the causal relationship to the study (nonserious disease or the like). Other nonserious diseases or the likes are periodically reported in writing.

6) Report to Ministry of Health, Labour and Welfare

Study-Representing Physician periodically reports to Ministry of Health, Labour and Welfare in writing, about the items shown in Article 59, Item 1-No.1 of Enforcement Regulations, according to Article 60, Enforcement Regulations of Clinical Trial Act, within one month after the day when CRB commented.

7) Report to Boehringer Ingelheim Japan

Upon receiving an expedited report on adverse event from Study Secretariat, Study-Representing Physician reports to Department of Pharmacovigilance, Boehringer Ingelheim Japan in writing within 24 hrs, including the Study-Representing Physician's comments.

8) Duty of Study Secretariat

Study Secretariat who received a report from Investigator or Subinvestigator asks Study-Representing Physician about urgency, importance, and degree of influence, and also asks IDMC for judgment, as needed. In addition, if necessary, action is taken to temporarily discontinue patient enrollment or to inform the participating medical institution.

9) To inform the participating medical institutions

Study-Representing Physician communicates the relevant information on adverse event to all the participating medical institutions in response to the IDMC recommendation as needed, including decision to continue, change, or discontinue the study.

１１: Data collection

１１-１. Completion of case report form (CRF) and report

Investigator or Subinvestigator appropriately records the investigation items predetermined by CRF in examination records or the likes, and stores. In this study, the data are input in EDC to report to Data Center, including no information allowing to identify patients.

１１-２. Data management

Data Center demands the data, scrutinize and inquire the data, and control, according to the data management plan, to provide the dataset for statistical analysis.

At the Data Center, a person responsible for data management confirms the data, and theoretically checks using computers according to the data management plan. Should inconsistency or missing data be detected, the responsible person asks Investigator or other study staff. In consequence, Investigator or other study staff corrects EDC, if necessary, according to the predetermined method.

The stage were all the data were cleaned is set as tentative data fixation and Case Review Committee is held, as needed. At the Data Center, the Case Review Committee results are reflected on EDC, to finally fix the data.

１２: Statistical analysis

Statistical analysis plain is otherwise provided in detail.

１２-１. Definition of analysis populations

１２-１-１. Full analysis set (FAS)

FAS means a population of all enrolled patients, excluding cases where the study protocol is seriously violated (consent not obtained or seriously deviating from the study procedure).

１２-１-２. Per protocol set (PPS)

PPS means a patient population from which such patients significantly violating study protocol-provided treatment or combination therapy as below are excluded from all enrolled patients.

・ Violating the inclusion criteria
・ Violating the exclusion criteria
・ Violating combination use-prohibited drugs or therapies
・ Violating drug compliance rate

１２-１-３. Safety analysis set (SAS)

SAS means a population of all enrolled patients.

１２-２. Analysis of primary endpoint

The number of severe arrhythmia events (NSVT/VT/VF) recorded in the arrhythmia device is evaluated at Week 0 and 24 (or study discontinuation), and subjected to Poisson regression analysis by the generalized linear model, assigning difference in No. of severe arrhythmia events between the empagliflozin and placebo groups as an objective variable.

１２-３. Analysis of secondary, exploratory, and safety endpoints

Descriptive summary statistics are calculated for each endpoint. In continuous variables, difference is estimated based on the least-squares method. In discrete variables, difference in the ratio is estimated based on chi-square distribution. Other analyses in detail are shown in the statistical plan otherwise provided.

１２-４. Significance level

The significance level is set to 5% on both sides.

１２-５. Handling of missing data

The missing data are not imputed as a general rule. Handling of each case is deliberated at Case Review Committee.

１３: Target sample size and rationales for setting

Sample size of patients: 210 (105 each for active drug and placebo)

[Rationales for setting]

The number of patients practically feasible to be enrolled is set to 210, calculating from the number of feasible medical institutions (in total of 20 institutions: 10 to 15 patients/institution). Assuming that 10 patients (5%) be dropped out of this population of patients, 200 patients are estimated to be included in the present study. Assuming the incidence ratio of severe arrhythmia in the placebo to empagliflozin group (IRR=1.44) based on the previous report[9)] and using the generalized linear model for difference in the number of arrhythmia, 99 patients/group are necessary to detect the IRR efficacy 1.44, when the significance level and power are set to 0.05 and 80%, respectively. In this case, the number of patients is within the actually possible number to recruit patients. Thus, the target number of patients was set to 210.

No. of patients necessary by power.

Power	No. of patient necessary
60%	124 (62+62)
70%	156 (78+78)
80%	198 (99+99)
90%	266 (133+133)

[No. of patients at each institution]

Name of institution	No. of patients prospected to be enrolled
Tachikawa General Hospital	10
Kitasato University Hospital	20
Kumamoto University Hospital	5
Gunma Cardiovascular Center	10
Kokura Kinen Hospital	10
National Cerebral and Cardiovascular Center	30
Saitama Medical University International Medical Center	10-20
Sakakibara Heart Institute	3-5
Sapporo Medical University Hospital	10
Jichi Medical University Saitama Medical Center	3
Jichi Medical University Hospital	10
Chiba University Hospital	10
University of Tsukuba Hospital	2
Tokyo Women's Medical University Hospital	10
Nagoya University Hospital	3-4
Niigata City General Hospital	10
Niigata University Medical & Dental Hospital	30
Hirosaki University Hospital	10
Fukushima Medical University Hospital	12
Hokkaido University Hospital	5

１４: Ethics

In this clinical study, the human rights and welfare are protected in accordance with the ethical principles in the latest Helsinki Declaration and in compliance with the updated “Clinical Trial Act.”

１４-１. Ethical review

This clinical study is applicable to “Specified Clinical Research.” Thus, prior to initiation of the study, the study protocol is reviewed at CRB, where making an effort to hear the opinions and to take necessary measures, respecting the opinions, if commented. After the review, the approval application is made for implementation of this study at all the participating medical institutions. When the medical institutions all approved, the study is registered in jRCT, notified to MLHW, and then started.

１４-２. Protection of personal information

According to Article 27, Enforcement Regulation of Clinical Trial Act, any personnel involved in this study should carefully consider the patients’ privacy and personal information, and appropriately handle them. Specifically, patients enrolled at the time of eligibility tests are anonymized to protect personal information by giving enrollment No. in the study. In addition, Patient No. Table is made. EDC does not include information such as name and medical card No. able to identify patients. To publish the study results, no information able to identify patients is not included. The patients’ data obtained from the present study are not used for other than the objectives of this study. When used for other than the objectives of this study, consent is otherwise obtained from patients, as needed. If patients themselves require their own personal information to be disclosed, the relevant personal information is disclosed without any delay. In addition, if required to stop the data usage, utilization is discontinued without any delay.

１４-３. Compensation for health impairment

When this study caused some health impairment, the participating medial institution treats the health impairment or takes other necessary measures. The cost necessary for treatment is covered by public medical insurance which the patient joins.

In addition, study drug (active drug and placebo) used in this study is not a domestically available drug itself which Boehringer Ingelheim Japan imports and markets according to the Manufacturing and Marketing Approval, but what Study-Representing Physician personally imports from Boehringer Ingelheim Germany as a study drug. Therefore, any of health impairment caused by adverse reaction is not applicable to Relief System for Sufferers from

Adverse Drug Reactions of the PMDA. The active drug used is as effective as Jardiance® Tablets 10 mg distributed in Japan.

In this study, patients join the clinical study insurance to be prepared for study-related health impairment, and compensated for death and residual disability, if any.

１４-４. Patient’s benefit and disadvantage

This clinical study is carried out in common clinical practice for patients with type 2 diabetes. Because the active drug is administered according to the dosing regimen approved for this drug, patients are unlikely to be at risk over the routine clinical examination. However, adverse reaction may occur. In the placebo group, blood glucose is concerned to be poorly controlled, but additional administration is possible in that case (Refer to Sections 7-6 and 7-8).

In this study, blood volume to be sampled for the hematological tests is 17 mL for the routine tests and 34 mL for the special tests (e.g., blood ketone body fraction), increasing the necessary sampling volume by 17 to 51 mL/sampling.

In addition, patients are exposed to radioactivity in the ^{123}I-MIBG myocardial scintigraphy tests to evaluate the activity of cardiac sympathetic nerves. However, exposure to radiation in patients is too low, being unlikely to cause a certain influence. Furthermore, patients receive periodic detailed examinations during the study period, to check health condition.

１４-５. Patient’s expenses

Examination cost other than study drug during the study period is paid by patients themselves or covered by the health insurance which the patient joins. The cost of “Empagliflozin 10 mg Tablets” and “Placebo Tables” supplied as study drug is paid from the funds for this study. The cost of Hematological tests (special) is also paid from the funds for this study. In addition, QUO Card equivalent to ¥20,000 is paid to each patient at Week 0 and at completion of the study (Week 24 or study discontinuation).

１５: Quality control and assurance

１５-１. Source data

Source data provided by Clinical Trial Act is defined as below in this study.

１) Reference data to input EDC: Electronic/paper medical cards, nursing records, and test data, etc.

２) Some of EDC inputs: Matters of judgement and findings associated with causal relationship and seriousness of adverse events, which are directly input in EDC because the data applicable to 1) above are not available.

１５-２. Handling of data and samples

The clinical data recorded in source data and EDC or residual samples are retained at the participating medical institutions and Department of Cardiovascular Biology and Medicine, Niigata University Graduate School of Medical and Dental Sciences, for 5 years after the report date of study discontinuation or completion. The remaining samples are limitedly utilized, if new information was obtained and it was decide that additional analysis is necessary. The purposes to utilize the remaining samples and the storage duration should be agreed with patients in advance. To exchange samples and information among the participating medical institutions, Sample and Information Exchange Record is provided. After completion of the storage duration, they are appropriately disposed at the respective medical institutions.

Blood ketone body fraction (acetoacetic acid, 3-hydroxybutyric acid, and total ketone bodies) and blood catecholamine fraction (adrenalin, noradrenalin, and dopamine), erythropoietin, and reticulocytes are retrieved by SRL Inc. from the respective participating medical institutions for analysis. The remaining test samples are retrieved and stored at Department of Cardiovascular Biology and Medicine, Niigata University Graduate School of Medical and Dental Sciences.

In addition, the samples for metabolome analysis is once retrieved to Department of Cardiovascular Biology and Medicine, Niigata University Graduate School of Medical and Dental Sciences, and then forwarded to Institute for Advanced Biosciences, Keio University.

１５-３. Record storage

The following documents essential for the specified clinical study are stored for 5 years by Investigator or Study-Representing Physician after the report data of study discontinuation or completion: Matters/data identifying patients or related to examinations and tests (source data, clinical data recorded in EDC, and related documents such as Patient No. Table), study protocol, study plan, explanation to patients, and consent-related documents (patient information), Clinical Study Report, review-related documents received from CRB, monitoring and audit-related documents, study contract, and summary of drugs to be used.

１５-４. Monitoring and audit

This clinical study is applicable to “Specified Clinical Research,” and monitored and audited by a third party, according to Clinical Trial Act, to secure the scientific quality and data reliability. In reality, the appropriate conduct of this study adhering to the protocol is confirmed by on-site inspection, e-mail, and telephone. When the study conduct was monitored, a

monitor completes the monitoring report, and submits to Study-Representing Physician.

Monitoring and audit follow the otherwise provided SOP (Appendices I and J). For monitoring and audit, Study-Representing Physician or others provides all the clinical study-related records such as source data, but Study-Representing Physician or others involved in monitoring and audit should keep confidential the medical information possible to identify patients.

１５-５. Inspection by CRB or regulatory authority

When CRB or regulatory authority inspects according to Clinical Trial Act, Study-Representing Physician or others provides with all the clinical study-related records such as source data, and should keep confidential the medical information possible to identify patients.

１５-６. Control of incompatibility

１５-６-１. About incompatibility and serious incompatibility

"Incompatibility" refers to incompliance with regulations, study protocol, and SOP as well as study data tampering and manipulating. "Serious incompatibility" means what influences the human rights and safety of patients, study progression, and reliability of the study results. For example, the incompatibility case is incompliance with the inclusion and exclusion criteria, study discontinuation criteria, and combination use-prohibited therapies, excluding the cases of deviation from the study protocol for medically inevitable reasons to circumvent emergent risk in patients.

１５-６-２. Duration to collect incompatibility information

From the date of consent obtained until completion of the study period.

１５-６-３. Report of incompatibility

（For detail, refer to Appendix H: SOP at occurrence of incompatibility）

Investigator reports the study-related and confirmed incompatibility to administrator of the participating medical institution, and to Study-Representing Physician, too. The records on incompatibility are appropriately maintained and stored. Reporting methods are as shown below.

[Report type]

1) Routine report

Investigator or Subinvestigator shares information on incompatibility, if recognized,

and promptly fills out EDC, and reports to Study-Representing Physician.

2) Regular report

According to Article 59, Enforcement Regulations of Clinical Trial Act, Study-Representing Physician periodically reports onset status of the clinical study-related incompatibility with the Enforcement Regulations or the protocol to CRB. Calculating from the day when the protocol was submitted to MHLW, regular report is made yearly within 2 months after every annual term is due. In addition, any incompatibility case to be reported to MHLW should be consulted with MHLW for confirmation every time, according to Article 60, Enforcement Regulations, Clinical Trial Act.

[Procedure for reporting]

1) Report to administrator of the participating medical institution

Investigator or Subinvestigator reports the recognized case of incompatibility even to administrator of the participating medical institution.

2) Report to IMDC

Study-Representing Physician reports to the IMDC in writing as needed, and asks to review the measures taken for the incompatibility.

The IMDC reviews the report, and reports the necessary measures to be taken for handling of patients or the possibility to continue the study to Study-Representing Physician in writing.

3) Report to CRB

Study-Representing Physician hears opinions from the IDMC as needed, and then, if considered serious, promptly reports to CRB using a designated form. When the case is considered incompatible but not serious, regular report is made using a designated form.

4) Report to MHLW

According to Article 60, Enforcement Regulations of Clinical Trial Act, Study-Representing Physician reports to MHLW through the regular report in writing, every time consulting with MHLW if there is any incompatibility to be reported.

5) Duty of study secretariat

Study secretariat which received any incompatibility report from Investigator or Subinvestigator asks Study-Representing Physician or IDMC, if necessary, to judge the seriousness and effects of the case. In addition, Study secretariat takes measures such as temporal discontinuation of enrollment or announcement to the participating medical institutions, as needed.

6) Announcement to participating medical institutions

After hearing from CRB, Study-Representing Physician summarizes the serious incompatibility case in a predetermined form, lays down the methods to prevent from reoccurrence, and informs all the participating medical institutions.

１５-７. Report to administrator of participating medical institution

Events are summarized below, which are related to quality control and necessary to report to administrator of participating medical institutions, other than the items related to above mentioned “Section 10: Measures to be taken for AE onset” and incompatibility.

１) Investigator reports status of study progression to administrator of the participating medical institution, pursuant to provisions of the institution.

２) Investigator reports the essential items to administrator of the participating medical institution at completion (or discontinuation) of the study.

3) Study-Representing Physician, etc., completes and publishes the report on primary evaluation endpoints, summary report, and their overview without any delay, and reports to administrator of the participating medical institution.

4) Study-Representing Physician, etc., promptly reports to administrator of the participating medical institution about what CRB commented.

１５-８. Regular report to CRB

Study-Representing Physician reports progression status of specified clinical study to CRB. Calculating from the day when the protocol was submitted to MHLW, the report is yearly made within 2 months after every relevant annual term is due. In addition, when regular report to CRB was carried out, Study-Representing Physician promptly provides information for other Investigators to that effect. Those informed Investigators promptly report the information to administrators of the participating medical institutions.

１５-９. Regular report to MHLW

Study-Representing Physician reports study progression status to MHLW within one month after the day when CRB commented.

１５-１０. Management for implementation of double-blind study

Investigator or Subinvestigator keeps the study double-blinded during the study period. For example, interview about the tests (urine sugar tests and urinary ketone body tests) able to distinguish whether the study drug is an active drug or placebo or about change in urine volume should be avoided as much as possible.

16: Study fund and conflict of interest (COI)

16-1. Study fund

This clinical study is sponsored by Boehringer Ingelheim Japan, based on the study contract with Niigata University. The study fund is born by Boehringer Ingelheim Japan, based on funding from Boehringer Ingelheim Germany and US Eli Lilly and Company (hereinafter referred to as US Lilly). Although Eli Lilly Japan K.K. is not involved in the fund flow, parent company US Lilly is involved, and cooperates with Boehringer Ingelheim Japan to promote sales of empagliflozin (Refer to Fig. 4).

Boehringer Ingelheim Japan, Boehringer Ingelheim Germany, and US Lilly are involved in funding and planning the study and providing information for proper use of the study drug, but not involved in planning, implementing, analyzing, and publishing this study. When these three companies are asked by Study-Representing Physician for opinions on planning and implementing this study, they can comment only from the scientific and ethical viewpoints based on proper use of this drug and purpose for this study.

[Figure 4] Found flow of EMPA-ICD

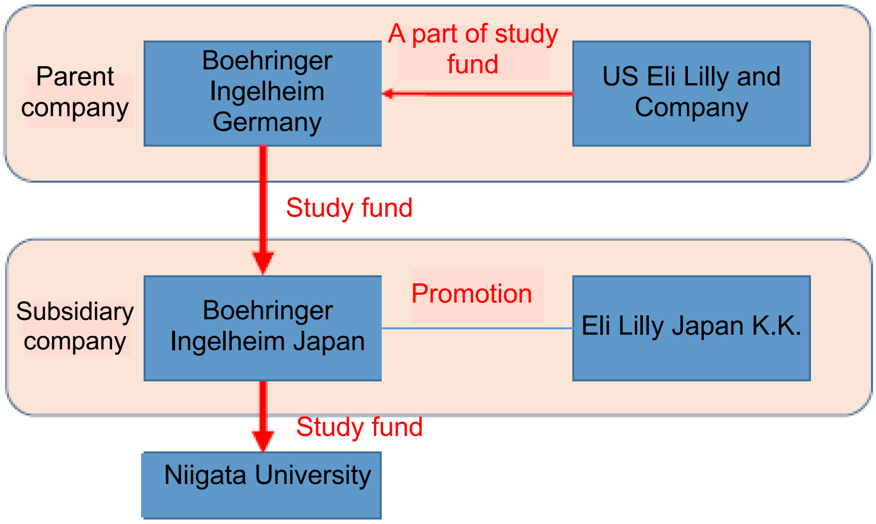

16-2. Conflict of interest (COI)

Persons engaged in this study appropriately create the COI management criteria according to the Clinical Trial Act, to control the COI management plan. Specifically, the COI management about involvement of manufacturing/marketing business license holder is planned based on fact affirmation at each of the participating medical institutions, and reported to CRB. Comment from the CRB should be responded, if any. The contents of COI

are disclosed in response to the requests of academic conferences or medical journals for presentation or publication of the study results.

The COI is disclosed as below according to Clinical Trial Act.

Study-Representing Physician Tohru Minamino, Department of Cardiovascular Biology and Medicine, Niigata University Graduate School of Medical and Dental Sciences<br>COI with Boehringer Ingelheim Japan: Personal interest relationship over annual ¥1 million

１７: Publication of study results and attribution of right

１７-１. Registration of clinical study

This study is planned to be registered in jRCT (https://jrct.niph.go.jp/), to disclose information. Participating medical institution or study secretariat is involved in registration by enrollment of 1st patient.

１７-２. Publication and attribution of study results

Study-Representing Physician announces the study results upon completion without any delay. The study results are announced after prior review and approval by Study-Representing Physician, Chief Researcher, and Steering Committee. In addition, Study-Representing Physician completes a report on primary evaluation endpoints or summary report and overview by the deadline provided by Clinical Trial Act, and submits to administration of the participating medical institutions. Simultaneously, Study-Representing Physician asks CBR for its comments, and publishes them.

Authors to publish papers and to present at academic conferences are appropriately selected by Study-Representing Physician and Chief Researcher, according to the author requirements (authorship) in International Committee of Medical Journal Editors. All the data obtained in this study are attributed to the study organization.

Boehringer Ingelheim Japan, its affiliated companies, and third parties marketing or promoting the products of Boehringer Ingelheim Japan are allowed to utilize the present study results and reports so as to provide information for medical institutions or to use as reference data in approval application of drugs and medical devices without any charge in the world.

Presentation and attribution of the metabolome analysis results are based on the collaborative study contract by Department of Cardiovascular Biology and Medicine, Niigata University Graduate School of Medical and Dental Sciences with Institute for Advanced Biosciences, Keio University.

Disclosure of exploratory analysis results obtained in this study is likely to be less beneficial

for patients, and concerned even to induce misunderstanding or uneasiness. Thus, the individual analysis results are not disclosed at present. However, if information likely to seriously influence life or the results that it can be considered medically beneficial to disclose were obtained during the study processes, they may be disclosed.

１８: Revision of study protocol

If considered necessary, Study-Representing Physician consults with Steering Committee to decide revision of the study protocol. The decision is again reviewed at CRB and communicated to Investigator. Then, administrator of the participating medical institution approves the study based on the revised protocol. If considered necessary, patient information is promptly revised in association with revision of the study protocol, reviewed according to the same procedure, and approved by administrator of the participating medical institution. Using the updated patient information, patients are again asked about their willing whether to continuously participate in the study, to obtain the consent.

１９: Completion, discontinuation, or suspension of study

１９-１. Completion of study

When the study is completed at each of the participating medical institutions, Investigator reports to administrator of the participating medical institution in writing.

１９-２. Discontinuation or suspension of study

When the study drug is able to be definitely judged as effective or ineffective in the context of study objectives and contents at the time when No. of patients are not enough enrolled as planned, or judged as inappropriate to continue the study because of serious information on the study-related safety, the IMDC recommends Study-Representing Physician to discontinue or suspend the study. When decided to discontinue or suspend, Study-Representing Physician promptly communicates the reasons for discontinuation and measures taken for patients to the participating medical institution in writing.

When the study was discontinued or suspended according to judgment or condition of the participating medical institution, Investigator promptly reports to Study-Representing Physician.

２０: About Certified Review Board (CRB)

CRB at which this study is asked to be reviewed is as follows.

Name of CRB: Niigata University Central Clinical Study Review Committee
Location of CRB: 1-754 Asahimachi-dori, Chuo-ku, Niigata

Telephone: 025-368-9343
Facsimile: 025-227-0720
e-Mail address: crbcr@adm.niigata-u.ac.jp

２１: Study system

See Appendix K.

２２: References

1) Eur Heart J. 2008; 29: 1377-85.
2) Circulation. 2013; 128: 694-701.
3) Guidelines for diagnosis and treatment of cardiovascular diseases (2009 Joint Working Group Report).
Guidelines for Treatment of Chronic Heart Failure (2010 revised version).
4) Guidelines for diagnosis and treatment of cardiovascular diseases (2009 Joint Working Group Report).
Guidelines for Risks and Prevention of Sudden Cardiac Death (2010 revised version).
5) N Engl J Med. 2015 Nov 26;373（22）:2117-28.
6) Diabetes 2016;65:1190–1195.
7) Trends Endocrinol. Metab. 2014; 25: 42–52.
8) Treatment Guide for Diabetes 2016-2017, ed. The Japan Diabetes Society.
9) Clin Ther. 2014;36:1606-15.
10) Lancet Diabetes Endocrinol. 2014;2:369-84.
11) Diabetes 2014;63:1738–1747.

Patient Information Sheet

Comparison of empagliflozin or placebo for prevention of lethal ventricular arrhythmia in type 2 diabetic patients with implantable cardioverter defibrillators (ICD)

EMPA-ICD

We are going to explain the content of this clinical study to you.

This document aims to give you information in addition to our explanation and to deepen your understanding of the study. Please read this document thoroughly before you decide whether you would like to participate in the study.

You are free to decide whether to participate in the study. Even if you start participating in the study, you may stop participating at any time. Your refusal to participate will never affect your future treatment.

Before you can decide whether to participate in the study, you have to know as much as possible about the content of the study. If you have any questions or there are any words you don't understand, please feel free to contact us.

EMPA-ICD Study Group

Prepared on: October 17, 2018 (ver. 1.3)

Introduction

Our hospital verifies the usefulness of treatments and constantly seeks new ones to provide the treatment that is most suitable for various diseases in patients.

Heart disease and diabetes are treated with therapies that are supported by data from research performed in clinical settings. In clinical settings, such research is called clinical research. Medical activities associated with clinical research may be conducted only after obtaining patients' consent.

Unlike usual treatment, clinical research that aims to study a new treatment is partly investigative. There is a rule that, before clinical research is conducted, it must be reviewed and approved to ensure that it will be conducted in a way that is safe for participants and that involves sufficient ethical considerations for patients. A Certified Review Board, which reviews and gives opinions on clinical research in compliance with the Clinical Trials Act, reviewed this study titled "Comparison of empagliflozin or placebo for prevention of lethal ventricular arrhythmia in type 2 diabetic patients with implantable cardioverter defibrillators (ICD)" and confirmed that the content of the study is appropriate and that patient rights are protected. Moreover, the procedures necessary for conducting clinical research, including notifications to the Ministry of Health, Labour and Welfare and registration in the Japan Registry of Clinical Trials (hereafter called "jRCT"), have been appropriately performed.

Certified Review Board of this study

**Name of Certified Review Board:**	**Niigata University Central Review Board of Clinical Research**
**Location of the Certified Review Board:**	**754, Ichibancho, Asahimachidori, Chuo-ku, Niigata-shi, Niigata, Japan**
**Phone no. of the department in charge:**	**025-368-9343**
**Fax no. of the department in charge:**	**025-227-0720**
**E-mail address of the department in charge:**	**crbcr@adm.niigata-u.ac.jp**

You can visit the following website to see the content of the reviews and the Certified Review Board: https://www.crbcr.niigata-u.ac.jp/

This clinical study was planned to determine whether the drugs used to treat diabetes also improve arrhythmia in patients with diabetes who have an implanted arrhythmia device. Please read through this patient information sheet so that you fully understand the study before you voluntarily decide whether to participate in it. If you decide to participate, please sign a separate consent form. Please feel free to ask your study doctor about any difficult words or phrases in the patient information sheet.

1. Purpose of the Study

Patients with diabetes and an implantable cardioverter-defibrillator (ICD) or implantable cardioverter-defibrillator with biventricular pacing function (CRT-D) (both of which are referred to in the text below as an "arrhythmia device") are known to have arrhythmia more often than patients with an arrhythmia device who do not have diabetes. Blood glucose control appears to be very important in patients with diabetes and an arrhythmia device, but the guidelines of the Japanese Circulation Society do not specifically recommend a blood glucose target level or diabetes drugs. Currently, doctors themselves decide on target blood glucose levels during treatment and on dosing regimens.

In July 2014, sodium-glucose co-transporter 2 (SGLT2) inhibitors (drugs that inhibit the transport of glucose and sodium) were approved as a new treatment to reduce blood glucose levels by causing glucose to be excreted in the urine. SGLT2 inhibitors have various effects, including improving metabolism and autonomic nerve functioning, in addition to reducing blood glucose. These effects are expected to have a favorable impact in patients with cardiovascular diseases. This was found for the SGLT2 inhibitor empagliflozin, which in 2015 was reported to improve the prognosis of patients with diabetes who are at high risk of cardiovascular complications; in this case, “prognosis” refers to the onset or worsening of heart disease, hospitalization because of heart disease, or death resulting from heart disease. Moreover, in 2017, canagliflozin, another SGLT2 inhibitor, was reported also to improve prognosis. The mechanism by which SGLT2 inhibitors improve prognosis related to heart disease has not been fully clarified, but researchers think the positive effect of SGLT2 inhibitors on autonomic nerves may have a favorable impact on arrhythmia associated with heart disease.

Therefore, we planned this study to determine the effect of the SGLT2 inhibitor empagliflozin on the number of arrhythmias automatically recorded by an implanted arrhythmia device. The study will be performed in patients with diabetes who have an implanted arrhythmia device. Patients will be treated with empagliflozin or placebo (see next section), and the two groups will be compared.

What is placebo?

A placebo is a study drug that looks the same as the investigational drug, in this case the SGLT2 inhibitor, but does not contain any active ingredient. Even though the placebo does not contain any active ingredient, in some people it may have an effect (the so-called placebo effect) because they are aware that they are “taking medicine.” Therefore, the efficacy and safety of the investigational drug are properly evaluated by comparing it with the placebo. Such a method that uses a placebo and in which you and your study doctor are not told which study drug you are taking is called the double-blind method.

2. Summary of the Study

2.1. Patients who can participate

Patients who meet all of the following conditions can participate:

1) Patients aged 20 years or older (men and women)
2) Patients who underwent ICD or CRT-D implantation surgery more than 24 weeks before the study (regardless of the reason for the implantation)
3) Patients diagnosed with type 2 diabetes who can receive the study drugs (HbA1c 6.5% to 10% in blood tests, regardless of whether they have received previous treatment)
4) Patients who can provide written consent to participate in the study

2.2. Patients who cannot participate

Patients who meet any of the following conditions cannot participate in the study:

1) Patients who are taking an SGLT2 inhibitor
2) Patients who started treatment with an SGLT2 inhibitor within the past 24 weeks and then discontinued it
3) Patients with a past history of hypersensitivity to empagliflozin (Jardiance®)
4) Patients who experienced complications caused by abnormal blood glucose (diabetic ketoacidosis, diabetic coma, or a hypoglycemic attack requiring intervention by a third person) within the past 24 weeks
5) Patients with a severe infection or injury and those who will undergo surgery (excluding surgical operations to replace the battery cell in the arrhythmia device)
6) Patients with type 1 diabetes
7) Patients with severe cardiac failure rated as New York Heart Association (NYHA) Class IV (please see note below)
8) Patients with severe renal impairment (i.e., patients with an estimated glomerular filtration rate [eGFR] of less than 30 mL/min/1.73 m^2 or who are receiving dialysis therapy)
9) Patients with a serious hepatic function disorder (aspartate transaminase [AST] or alanine transaminase [ALT] more than 3 times higher than the normal local laboratory value)
10) Patients with pituitary or adrenal insufficiency
11) Malnourished patients (patients who are fasting, eat irregularly, have a poor diet or are debilitated)
12) Patients with a history of excessive alcohol intake
13) Patients who have a gastrointestinal disorder like diarrhea or vomiting and are likely to be dehydrated
14) Patients with a urinary tract or genital infection
15) Patients who are pregnant, of childbearing potential or breastfeeding or who want to

become pregnant while participating in this study

16) Patients with a low weight (BMI less than 18.5 kg/m^2)

17) Patients with any of the following events within the past 24 weeks: change of antiarrhythmic drug, catheter ablation for ventricular arrhythmia, coronary revascularization, open heart surgery, development of coronary artery disease, stroke or transient ischemic attack, infection requiring hospitalization, or cardiac failure requiring hospitalization

18) Patients using an arrhythmia device that is unable to record some non-sustained ventricular tachycardia episodes

19) Patients with non-remitted malignant tumor

20) Patients whom the study doctor judges as inappropriate to participate in the study

Note: The NYHA Functional Classification is used to classify symptoms into four classes, Class I to IV, as follows:

Class I	No limitation of physical activity. Ordinary physical activity does not cause undue fatigue, palpitation, dyspnea (shortness of breath), or anginal pain.
Class II	Slight limitation of physical activity. Comfortable at rest. Ordinary physical activity results in fatigue, palpitation, dyspnea (shortness of breath), or anginal pain.
Class III	Marked limitation of physical activity. Comfortable at rest. Less than ordinary activity causes fatigue, palpitation, dyspnea (shortness of breath), or anginal pain.
Class IV	Unable to carry on any physical activity without discomfort. Symptoms of heart failure and/or anginal pain may be present even at rest. If any physical activity is undertaken, discomfort increases.

2.3. Discontinuation of the Study

The study may be discontinued in any of the following cases. If it is discontinued, tests, including blood tests and echocardiography, will be performed for evaluation wherever possible.

Please see “Test Items and Schedule” on page 8 for the detailed testing schedule.

1) When it is determined to be difficult to continue the study because the underlying disease or complication is aggravated

2) When it is determined to be difficult to continue the study because of the onset of an adverse event

3) When it is determined to be difficult to continue the study because the patient moved

4) When it is determined to be difficult to continue the study because the patient was transferred to another hospital
5) When the patient asks to discontinue participation in the study or withdraws consent
6) When the patient takes an SGLT2 inhibitor after taking any study drug
7) When the patient experiences any of the following events, which are likely to influence the onset of severe arrhythmia, after taking any study drug: change of antiarrhythmic drug, catheter ablation for ventricular arrhythmia, coronary revascularization, open heart surgery, development of coronary artery disease, stroke or transient ischemic attack, infection requiring hospitalization, or cardiac failure requiring hospitalization
8) When the study doctor determines that it is difficult to continue the study for any other reason

2.4. Methods

1) Study procedures

In this study, patients will take study drugs in addition to their usual treatment. Patients who consented to participate will be randomly assigned in a 1:1 ratio to receive empagliflozin, an SGLT2 inhibitor (empagliflozin group), or placebo (placebo group). The placebo looks the same as the SGLT2 inhibitor, but the placebo does not contain the active ingredient (i.e., it does not contain empagliflozin). A computer will determine the allocation to each group, so neither the patient nor the study doctor can choose the group, and they will not know the result of the allocation until the follow-up period is completed and everything has been prepared for the data analysis. This is a very precise study method called the double-blind method and it makes it possible to properly evaluate the efficacy and safety of the SGLT2 inhibitor in comparison with the placebo (see “What is placebo?” under 1. Purpose of the Study). After patients have given informed consent and the initial eligibility tests have been performed, treatment will start according to the result of the computer-based allocation and will continue for 24 weeks.

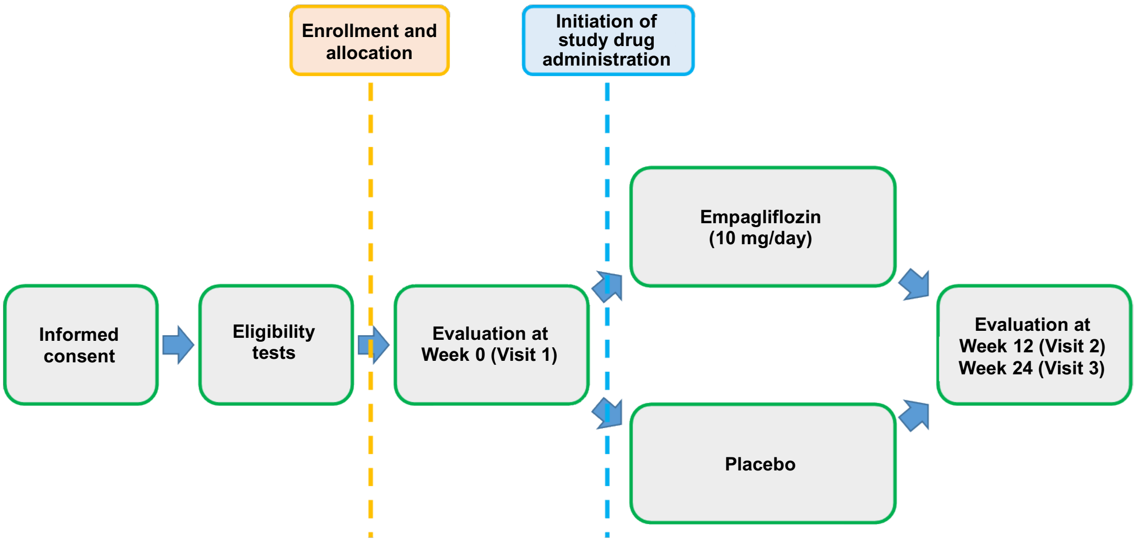

If you are treated at another hospital, please let us know the name of the hospital, the disease you are treated for there, and the drugs you are using. In addition, if you use any drugs that you have bought at pharmacies or other stores, please tell us about them. This is important so that we can conduct the study safely. If you are treated at another hospital, please note that we will notify the hospital of your participation in the clinical study by sending them the letter "Notification of your patient's participation in a clinical study and what we would like you to do."

2) Drugs to be used in the study

The drugs to be used in the study (free of charge) will be prescribed separately from other drugs. Study drugs may be supplied to you all at once at Week 0 (Visit 1) or in installments at subsequent visits. Your study doctor will carefully determine your availability for study visits and your symptoms, consider the best choice to ensure your safety and be responsible for taking appropriate action.

In this study, you will take one tablet of your study drug (empagliflozin 10 mg or placebo) once a day before or after breakfast. As mentioned above, both you and your study doctor will not know which group you are allocated to, so please take your study drug for 24 weeks and follow the instructions of your study doctor. Predetermined tests will be performed at the start of treatment and at Week 24 (Visit 3) or the time of study discontinuation.

At Week 12 (Visit 2), Week 24 (Visit 3) or the time of study discontinuation, please return all of the remaining study drugs and empty bottles to us or your study doctor.

3) Test Items and Schedule

[Test Schedule]

Enrollment and allocation (between Screening and Visit 1)

Initiation of study drug administration (between Visit 1 and Visit 2)

	Before treatment		Treatment period		
	Screening	Visit 1	Visit 2	Visit 3	At time of discontinuation
	Week -12 to 0	Week 0	Week 12	Week 24	
		–	(± 4 weeks)	(± 4 weeks)	
**Patient information/ informed consent**	○				
**Patient background**	○				
**Interview/physical examination**	○	○	○	○	△
**Confirmation of drug compliance**			○	○	△
**Confirmation of compliance with study procedures**		○	○	○	△
**Confirmation of other drugs being used**	○	○	○	○	△
**Height**	○				△
**Body weight/body temperature**	○	○	△	○	△
**Blood pressure/ pulse rate**		○	△	○	△
**Hematological tests**	○	○	△	○	△
**Hematological tests (blood glucose/lipids)**	○	○	△	○	△
**Hematological tests (special)**		○		○	△
**12-lead ECG**		○		○	△
**Holter monitoring**		○		○	△
**Echocardiography**	○	○		○	△
**^{123}I-MIBG myocardial scintigraphy**		△		△	△
**Adverse events**	○	○	○	○	△
**Evaluation of arrhythmia device**		○		○	△

○, Essential; △, Optional.

Optional items (△) may or may not be performed. Please contact your study doctor for further information. Wherever possible, fasting blood will be sampled after you have rested lying face up on an examination table for 30 minutes.

1. Patients who visit the hospital in the morning will undergo blood sampling without eating after dinner on the day before the test (no breakfast on the test day).

2. Patients who visit the hospital in the afternoon will undergo blood sampling without eating after breakfast on the test day (no lunch on the test day).

3. After informed consent and eligibility screening, at Week 0 (Visit 1) pre-treatment tests will be performed and then your study drug will be prescribed.

4. If any tests (hematological tests, 12-lead ECG, Holter monitoring, echocardiography, or ^{123}I-MIBG myocardial scintigraphy) are performed within 3 months before the tests at Week 0 (Visit 1), the data from those tests will be used.

5. Although no tests are scheduled at Week 12 (Visit 2), if you are not doing well, please consult with us. In addition, please bring empty bottles after taking medicine.
6. Repeat tests are scheduled at Week 24 (Visit 3) or at the time of study discontinuation.
   Please bring all of remaining drugs and empty bottles with you at your visits.
7. You will wear a Holter monitor for 24 hours and will need to visit the hospital on the following day to return it.

[Test Items]
Background information

You will be interviewed about the following information: gender, age, smoking history, alcohol-drinking history, arrhythmia that caused implantation with arrhythmia device and underlying diseases, make and type of arrhythmia treatment device, comorbidity and past history, drugs you are taking (called "concomitant medication"), and past history of non-drug treatment.

Physical examination

You will be asked about your subjective symptoms (for the NYHA classification).

Confirmation of drug compliance and concomitant medication

You will be asked whether you forgot to take any study drugs and whether there was any change in your concomitant medication.

Confirmation of compliance with the procedures in the protocol

Your study doctor will check whether the tests and treatment adhere to the study protocol.

Height, body weight, and body temperature

Height will be measured only during eligibility screening, but body weight and temperature will be measured at every visit.

Blood pressure and pulse rate

As a general rule, these will be measured in a sitting position after you have rested for more than 5 minutes.

Hematological tests (blood sugar and lipids): About 17 mL of your blood will be collected for the tests.

The following items will be measured, all of which are general items that tell us about your condition:

Hematology (red blood cell count, white blood cell count, hemoglobin, hematocrit, and platelet count), blood biochemistry (total protein, albumin, AST, ALT, alkaline phosphatase, total bilirubin, lactate dehydrogenase, blood urea nitrogen, sodium, potassium, chloride, uric acid, creatinine, eGFR), and brain natriuretic peptide.

Blood glucose and lipid tests: Fasting blood glucose and hemoglobin A1c, triglyceride, total cholesterol, high-density lipoprotein cholesterol, and low-density lipoprotein cholesterol

Note: HbA1c, AST, ALT, and eGFR are essential for eligibility tests.

Special blood tests (central measurement): About 14 to 34 mL of your blood will be collected for the

tests. Please see “Test Items and Schedule” on page 8 for the detailed blood test schedule.

The following items will be measured in the study tests (you will not be charged for the costs of the tests that are not covered by medical insurance). Essential tests: blood ketone body fraction, blood catecholamine concentration, erythropoietin, and reticulocytes. Optional tests: DNA and RNA tests and metabolome analysis.

The tests will be performed at central measurement institutions (SRL, Inc., Niigata University, and Institute for Advanced Biosciences of Keio University), as follows:

Blood ketone body fraction (acetoacetic acid, 3-hydroxybutyric acid, and total ketone bodies), blood catecholamine concentration (adrenalin, noradrenalin, and dopamine), erythropoietin, and reticulocytes: SRL, Inc.

DNA tests (telomere length, G-tail length) and RNA tests (P53, P21, and P16): Niigata University. The DNA and RNA tests will not analyze genetic data containing personal information.

Metabolome analysis: Institute for Advanced Biosciences, Keio University.

12-lead ECG tests

The following items will be measured by electrocardiography:

Heart rate, rhythm, PQ interval, QRS interval, and QT interval

Holter monitoring

Holter monitoring will measure the following items, which are detailed measures that are unlikely to be detected by an arrhythmia device:

Total recoding time, total number of ventricular premature contractions (VPCs), number of single VPCs, number of occurrences of two consecutive VPCs, and number of ventricular tachycardia

Echocardiographic tests

Echocardiography will measure the following items, which are general items to evaluate cardiac function:

left ventricular ejection fraction, E wave, A wave, E/A, early diastolic velocity at the septal mitral annulus, early diastolic velocity at the lateral mitral annulus, left ventricular diastolic function, tricuspid regurgitation peak velocity, and left atrial volume index

^{123}I-MIBG myocardial scintigraphy tests

The following items will be measured by ^{123}I-MIBG myocardial scintigraphy to determine the status of the autonomic nervous system (^{123}I-MIBG myocardial scintigraphy is widely used in daily clinical practice to estimate the severity or prognosis of heart disease; however, it takes about 3 to 6 hours, so the tests at both Week 0 and Week 24 or the time of study discontinuation are optional):

H/M (heart-to-mediastinum) ratio and washout rate

Safety evaluation

Adverse events: Onset of any unfavorable or unintended signs, symptoms, or diseases will be determined.

Evaluation of arrhythmia device

The data recorded in the device, including the number of ventricular and atrial arrhythmias and biological monitoring indicators, will be output and used.

If data are available from the 24 weeks before Week 0 or the time of evaluation after informed consent, these data will be used as the data at Week 0 (Visit 1). The data from the 24 weeks after the start of study treatment will be used as the data at Week 24 (Visit 3).

Note: The information on patient health and any genetic elements that may possibly be transmitted to children is not included in the study endpoints.

Please feel free to ask your study doctor about any unknown words or phrases.

2.5. Scale of the study

This study will be conducted at university hospitals and general hospitals throughout Japan.

The target sample size is 210 participants (105 per group).

Expected study period: Until October 2022 (expected enrollment period: until April 2020)

If you participate in the study, the expected participation period is about 24 weeks.

(Please see the table in 2.4. Methods (1).)

3. How the samples and data will be used and how long they will be stored

The data to be collected in the study will be appropriately managed at sites and data centers so that personal information is protected. Investigators, who have specific access rights, may access the collected data stored at data centers.

The data to be obtained and managed in the study will not contain information that identifies individuals, such as patient name and birth date. For data management, the data of each patient will be anonymized by coding them independently of the study and will then be securely stored and analyzed.

After measurements have been made, samples collected at each site will be destroyed appropriately according to local rules. Samples collected for special blood tests will be anonymized with the study enrollment number and collected and measured in central measurement institutions (SRL, Inc., Niigata University, and Keio University).

In the DNA tests (telomere length, G-tail length) and RNA tests (P53, P21, P16) to be performed in the study, no analysis of genetic data containing personal information will be performed. Remaining samples will be appropriately stored with unidentifiable personal information, as specified by Niigata University School of Medicine.

Remaining samples after completion of measurements will be stored at the laboratory of

Cardiovascular Medicine, Niigata University School of Medicine, and will be additionally analyzed after their availability is confirmed, in accordance with predetermined procedures, if new information is obtained with respect to the study and the representative investigator determines that additional analysis is required. Because analysis will be performed with unidentifiable personal information, your personal information will also not be divulged in case of additional analysis.

Samples and data will be stored for 5 years after discontinuation or completion of the study and will then be destroyed; individuals will be unidentifiable.

4. Expected advantages and disadvantages of participation in the study

If you participate in the study, your condition will be regularly examined. Blood tests, electrocardiography, Holter monitoring, echocardiography, and ^{123}I-MIBG myocardial scintigraphy would enable your health condition to be accurately understood and help future treatment. The special blood tests, which cover unknown areas, are unlikely to help future treatment. The results of the study are likely to help treatment of other patients with your disease in the future. However, during the study, you will have to restrict your diet and visit the hospital for tests, which will have an impact on your life and take time. In addition, you will be charged the costs for some of the tests (see 12. Study-related costs for patients).

Moreover, if you are allocated to receive the placebo (which has the same appearance as the SGLT2 inhibitor but does not contain any active ingredient), no effect on diabetes is expected. However, you will be receiving treatment with other diabetes medications, as described below, which can minimize this disadvantage of receiving placebo.

If new information is obtained during the study that may affect the continuation of patient participation in the study (such as information on a new serious adverse reaction), updated information will be provided to patients as needed. At that time, we will ask you if you want to continue your participation in the study. At such a time, we ask that you please decide voluntarily whether you want to continue.

5. Expected effects and most frequently observed adverse reactions

Because the drug to be used in the study (empagliflozin) has the effect of reducing blood glucose, your blood glucose levels are expected to decrease. However, almost all current treatments not only have effects but also cause adverse reactions. Some adverse reactions rarely lead to severe symptoms.

[Expected adverse reactions to empagliflozin]

[1] Clinically significant adverse reactions and frequencies

Hypoglycemia (2.3%), dehydration (0.1%), ketoacidosis (frequency unknown), pyelonephritis (frequency unknown), sepsis (frequency unknown), necrotizing fasciitis of the perineum

(Fournier's gangrene; frequency unknown)

[2] Other adverse reactions (0.1% to less than 5%)

Constipation, abdominal distention, thirst, hunger, cystitis, urinary tract infection, vulvovaginal candidiasis, asymptomatic bacteriuria, balanitis, genital pruritus, pollakiuria, polyuria, urine output increased, rash, hyperlipidemia, hemoconcentration, dizziness, dysgeusia, weight decreased, presence of urine ketone bodies

You may experience other adverse reactions. Use of the study drug in combination with other drugs may increase the risk of adverse reactions, including hypoglycemia. If you get any other disease or feel different from usual while participating in the study, please immediately contact your study doctor, who will change your treatment or treat your symptoms appropriately.
Please see "11. What we would like you to do" for further information.

In this study, a larger amount of blood than usual (17 to 51 mL per visit more than usual) will be collected because some components will be examined for the study in addition to the blood tests that are performed in your usual care. In ^{123}I-MIBG myocardial scintigraphy, you will be exposed to a little radiation, which appears to hardly cause any injury to health (some special blood tests and ^{123}I-MIBG myocardial scintigraphy are optional).
You may participate in additional tests for this study, which may take up more of your time.

6. Other treatments

Besides empagliflozin, other diabetes medications are available, including other SGLT2 inhibitors, sulfonylurea drugs, dipeptidyl peptidase 4 inhibitors, rapid-acting insulin secretagogue, α-glucosidase inhibitors, biguanides, thiazolidine drugs, insulin preparations and glucagon-like peptide-1 analogues. Glycemic control is possible with these drugs, which have different mechanisms of action and pros and cons. In addition, glucose levels can be controlled by non-drug methods, such as diet and exercise therapies. Even if you do not participate in this study, you will continue to receive appropriate treatment, including medical treatment. Please fully consult with your study doctor to select such treatments.

7. If any additional treatment is required

If your study doctor determines that your glycemic control is poor, you will receive additional treatment, which may include increasing the dose of the diabetes medication you started before participating in the study or prescribing a diabetes medication other than the SGLT2 inhibitor. Please follow the instructions of your study doctor.

8. Treatment after completion of the study

After completion of the study, you will receive treatment depending on your condition and wishes.

Please make sure to return remaining study drugs and empty bottles to your study doctor at the time of the Week 24 tests or the time of study discontinuation.

9. Your decision to participate in the study is voluntary

Your decision to participate is voluntary. Even if you refuse to participate, you will not be at a disadvantage because you will still receive the best treatment for your condition.

If you decide to participate, you may consult with us, including your study doctor, and may withdraw from the study at any time, in which case you will also receive the best treatment for your condition and never be disadvantaged.

10. Protection of your human rights

Whenever we report on your disease, symptoms, physical conditions, and tests at scientific meetings or academic conferences, to protect your privacy your personal information, including your name, birth date, etc., will never be used in such reports.

By signing the consent form, you tell us that you agree to the above.

11. What we would like you to do

During the study, please follow our instructions, including those of your study doctor. In particular, wherever possible please follow test procedures, including those regarding meals and resting. If any abnormalities occur, please immediately contact us or your study doctor (see 17. Contacts for the Study).

The appendix “Major adverse reactions to empagliflozin” describes details of possible major adverse reactions to the investigational drug. If you experience any such adverse reactions, please contact us using the contacts as described in “17. Contacts for the Study” below.

Please return all of the remaining drugs and empty bottles at Week 12 (Visit 2), Week 24 (Visit 3) or the time of discontinuation to us or your study doctor. We will destroy them appropriately.

If you are injured in this study, despite following our instructions and those of your study doctor, we will be responsible for treating you appropriately.

If you visit another hospital, please let us know the name of the hospital, the disease you are treated for there, and **the drugs you are using. In addition, if you use any drugs that you have bought at pharmacies or other stores, please tell us about them.**

If you go to another hospital or clinic, please make sure to notify the doctor at the hospital or clinic of your participation in the study.

12. Study-related costs for patients

The drugs to be used in the study (free of charge) will be prescribed separately from other drugs, and the costs of the investigational drug administered during the study and those of special blood tests not covered by insurance will be covered by study expenses. The costs of usual care will be covered by your public medical insurance, that means your insurance will pay the costs of general blood tests, electrocardiography, Holter monitoring, echocardiography, and ^{123}I-MIBG myocardial scintigraphy because these tests will be performed as part of your usual care. Such tests are part of the usual care of patients with heart disease and those with diabetes.

Participation in the study will cause some burden such as an increase in the amount of blood collected and the time required for additional testing. Therefore, to compensate you for your participation we will give you a QUO card worth ¥20,000 at both Week 0 and at the time of completion of the study (Week 24 or discontinuation for any reason).

13. Compensation for study-related injury

The study was planned scientifically by referring to previous studies, and it will be conducted carefully. However, if you experience any injury, such as an adverse reaction, during or after the clinical study, doctors will appropriately examine and treat you. Although you will be asked to pay for the costs of such treatment beyond what the public medical insurance covers for your usual care, you will be reimbursed for any costs associated with any study-related injury by clinical study insurance.

Because this study is not a clinical trial for the development of a new drug by a pharmaceutical company, participants with a study-related injury will not receive special compensation from the company organizing the trial. However, the representative study investigator has clinical study insurance to cover any injury related to the clinical study. Please note that patients will not receive any compensation if the investigational drug has no effect or if patients receive no therapeutic benefit because they received the placebo.

Please read through the “Summary of Compensation System for the Study” for more information on the compensation we will give to you in case of injury.

If you have any questions, please contact your study doctor (see 17. Contacts for the Study).

14. Possible Conflicts of Interest

The study group performing this study includes cardiovascular and diabetes specialists, and the representative investigator is Tohru Minamino, Department of Cardiovascular Medicine, Niigata University Graduate School of Medical and Dental Sciences. During the study, the committee, which

consists of independent specialists who are not participating in the conduct of the study, will monitor the status of conflicts of interest. Before the start of the study, the representative investigator will set a standard for conflicts of interest and manage conflicts of interest in all persons involved in the study. The conflicts of interest management plan was reviewed by the Certified Review Board.

The drugs to be used in the study will be provided by Nippon Boehringer Ingelheim Co., Ltd. The study is funded under the study agreement executed between the company and Niigata University. Such funding will be provided to Nippon Boehringer Ingelheim Co., Ltd, by Boehringer Ingelheim Germany and Eli Lilly and Company (US). Eli Lilly and Company (US), a parent company of Eli Lilly Japan K.K., and not Eli Lilly Japan K.K. is involved in the flow of the study funds and is cooperating with Nippon Boehringer Ingelheim Co., Ltd, to promote empagliflozin. Please refer to the below figure for information on the flow of funds.

Employees of the respective company are not involved in either the conduct or analysis of the study or in decision-making that may affect the results of the study.

[Flow of study funds]

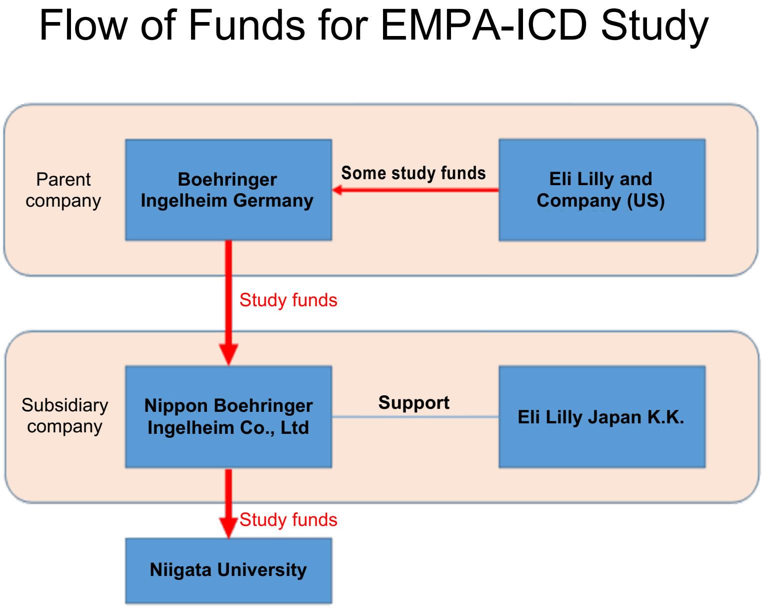

<Announcement of Conflicts of Interest>

Under the Clinical Trials Act, we report the following conflicts of interest (COI):

1. Representative Investigator: Tohru Minamino, Department of Cardiovascular Medicine, Niigata University Graduate School of Medical and Dental Sciences
   COI with Nippon Boehringer Ingelheim Co., Ltd: Personal interest relationship of more than ¥1 million annually

15. Monitoring and auditing

To determine whether the study is conducted properly and safely, patient human rights are protected, and the results of treatments and tests are accurately reported, third parties may visit study sites, including our hospital, to evaluate study documents by monitoring and auditing them. During monitoring and auditing, the following third parties may perform source data verification of your medical records and test results: the contract research organization (CRO) Micron, Inc., healthcare professionals of medical institutions, and data managers.

The monitoring and auditing will be performed only by the third parties for whom the permission to access medical records has been reviewed by the Certified Review Board and who are permitted to access medical records by the director of the hospital. Under laws and regulations, the third parties are liable for maintaining the confidentiality of personal information. Therefore, the personal information they obtain will never be divulged. By signing the consent form, you tell us that you approve the monitoring and auditing of the study.

Besides monitoring and auditing, the Certified Review Board and regulatory authorities (Ministry of Health, Labour and Welfare) may investigate the study, which may possibly include accessing personal information; however, the information will never be divulged. Approval of the investigation by the Certified Review Board or regulatory authorities will be obtained by using the consent form.

Monitoring and auditing organization: Micron, Inc.

16. Publication of the information on the study

Because the summary of the study will be registered in a publication database (the system for publication of the summary of protocols and clinical research, jRCT), you may find the content, status, results, etc. of the study on the internet. Your personal information will never be disclosed, but you may see the protocol that describes the study. If you want more information, please contact your study doctor.

jRCT website:
https://jrct.niph.go.jp/

17. Contacts for the Study

If you have any questions about the study or you suffer any injury, please feel free to contact us or your study doctor.

Representative Investigator

Tohru Minamino, Part-time Lecturer

Department of Cardiovascular Medicine, Niigata University Graduate School of Medical and Dental Sciences

<Contact for the study>
EMPA-ICD Research Office
Department of Cardiovascular Medicine, Niigata University Graduate School of Medical and Dental Sciences
Asahimachi-Dori 1-757, Chuo-ku, Niigata, Niigata 951-8510, Japan
Tel: 025-227-2185, FAX: 025-227-0774

Investigator and contact at each study site (in order of the Japanese syllabary)

■ Tachikawa General Hospital, Tachikawa Medical Center
Principal investigator: Masaaki Okabe (Director of the Hospital), Cardiovascular Medicine
Address: Asahioka 1-24, Nagaoka-shi, Niigata, 940-8621
Tel: 0258-33-3111, Fax: 0258-33-8811

■ Kitasato University Hospital
Principal investigator: Shinichi Niwano, Medical Professor, Cardiovascular Medicine
Address: 1-15-1, Kitazato, Minami, Sagamihara, Kanagawa, 252-0375
Tel: 042-778-8111 Fax: 042-778-9371

■ Kumamoto University Hospital
Principal investigator: Kenichi Tsujita, Professor, Cardiovascular Medicine
Address: 1-1-1 Honjo, Chuo-ku, Kumamoto City, Kumamoto 860-8556
Tel: 096-344-2111, Fax: 096-373-5906

■ Gunma Prefectural Cardiovascular Center
Principal investigator: Shigeto Naito, Director of the center

Address: 3-12, Kameizumi-town, Maebashi-city, Gunma 371-0004
Tel: 027-269-7455, Fax: 027-269-1492

■ Kokura Kinen Hospital
Principal investigator: Kenji Ando, Medical Director, Cardiovascular Medicine
Address: 3-2-1 Asano Kokurakita-Ku, Kitakyushu, Fukuoka 802-8555
Tel: 093-511-2000, Fax: 093-511-3240

■ National Cerebral and Cardiovascular Center Hospital
Principal investigator: Kengo Kusano, Director of the Cardiovascular Department
Address: 5-7-1 Fujishirodai, Suita, Osaka, 565-8565
Tel: 06-6833-5012, Fax: 06-6833-9865

■ Saitama Medical University International Medical Center
Principal investigator: Ritsushi Kato, Professor, Division of Cardiology, Department of Cardiology
Address: 1397-1, Yamane, Hidaka-City, Saitama 350-1298
Tel: 042-984-4111, Fax: 042-984-4740

■ Sakakibara Heart Institute
Principal investigator: Junichi Nitta, Cardiovascular Medicine
Address: 3-16-1 Asahi-cho, Fuchu, Tokyo, 183-0003
Tel: 042-314-3111, Fax: 042-314-3150

■ Sapporo Medical University Hospital
Principal investigator: Tetsuji Miura, Professor, Department of Cardiovascular, Renal and Metabolic Medicine
Address: 16-291, Minami-ichijo-nishi, Chuo-ku, Sapporo 060-8543
Tel: 011-611-2111, Fax: 011-621-8059

■ Jichi Medical University Saitama Medical Center
Principal investigator: Takeshi Mitsuhashi, Associate Professor, Cardiovascular Medicine
Address: 1-847 Amanuma-cho, Omiya-ku, Saitama 330-8503
Tel: 048-647-2111, Fax: 048-648-5166

■ Jichi Medical University Hospital

Principal investigator: Kazuomi Kario, Professor, Cardiovascular Medicine
Address: 3311-1 Yakushiji, Shimotsuke-shi, Tochigi 329-0498
Tel: 0285-44-2111, Fax: 0285-44-8169

■ Chiba University Hospital
Principal investigator: Yoshio Kobayashi, Specially Appointed Assistant Professor, Cardiovascular Medicine
Address: 1-8-1 Inohana, Chuo-ku, Chiba-shi, Chiba, 260-8677
Tel: 043-222-7171, Fax: 043-224-3830

■ University of Tsukuba Hospital
Principal investigator: Yukio Sekiguchi, Associate Professor, Cardiovascular Medicine
Address: 2-1-1 Amakubo, Tsukuba, Ibaraki 305-8576
Tel: 029-853-3900, Fax: 029-853-3904

■ Tokyo Women's Medical University Hospital
Principal investigator: Nobuhisa Hagiwara, Professor, Cardiovascular Medicine
Address: 8-1. Kawada-cho, Shinjuku-ku, Tokyo, 162-8666
Tel: 03-3353-8111, Fax: 03-3356-0441

■ Nagoya University Hospital
Principal investigator: Toyoaki Murohara, Professor, Cardiovascular Medicine
Address: 65 Tsurumai-cho, Showa-ku, Nagoya 466-8560
Tel: 052-741-2111, Fax: 052-744-2785

■ Niigata City General Hospital
Principal investigator: Kazuyoshi Takahashi, Director and Deputy Center Head of the Department of Cardiovascular Medicine
Address: 463-7 Shumoku, Chuo-ku, Niigata City, Niigata 950-1197
Tel: 025-281-5151, Fax: 025-281-5187

■ Niigata University Graduate School of Medical and Dental Sciences
Principal investigator: Tohru Minamino, Professor, Department of Cardiovascular Medicine
Address: 754, Ichibancho, Asahimachidori, Chuo-ku, Niigata-shi, Niigata 951-8510
Tel: 025-227-2185, Fax: 025-227-0774

■ Hirosaki University Hospital
Principal investigator: Hirofumi Tomita, Professor, Cardiology and Nephrology
Address: 53 Honcho, Hirosaki, Aomori 036-8563
Tel: 0172-33-5111, Fax: 0172-39-5189

■ Fukushima Medical University Hospital
Principal investigator: Yasuchika Takeishi, Professor, Cardiovascular Medicine
Address: 1 Hikarigaoka, Fukushima-city, Fukushima 960-1295
Tel: 024-547-1111, Fax: 024-547-1998

■ Hokkaido University Hospital
Principal investigator: Toshihisa Anzai, Professor, Department of Cardiovascular Medicine
Address: 5 Kita14jonishi, Kita-Ku, Sapporo, Hokkaido 060-8648
Tel: 011-716-1161, Fax: 011-706-7627

If you decide to participate in the study after fully understanding the content of the study, please sign and date the consent form. Thereafter, you will receive the patient information sheet and consent form for you to keep.

Consent Form

Dear Director of XX Hospital

I hereby give voluntary consent to participate in the study titled "Double-blind, placebo-controlled study to evaluate the change in the number of severe arrhythmias after empagliflozin intervention in patients with an implanted arrhythmia treatment device complicated by type 2 diabetes mellitus" (EMPA-ICD) after receiving a thorough explanation about the following contents of the patient information sheet from my study doctor and fully understanding the explanation and contents regarding my participation in the study:

- A Certified Review Board reviewed the study.
- The study was approved by the Certified Review Board, and the protocol was submitted to the Ministry of Health, Labour and Welfare.
- Purpose of the study
- Summary of the study
- How samples and data will be used and how long they will be stored
- Expected advantages and possible risks of participation in the study
- Expected effects and adverse reactions, and disadvantages to patients
- Other treatments
- Actions after completion of the clinical study
- My decision to participate in the study is voluntary
- Protection of my human rights
- What I will have to do
- Study-related costs for patients
- Compensation for the study
- Possible conflicts of interest
- Monitoring and auditing
- Disclosure of the study information
- Contacts for the study

* Optional tests (Please check one of the following boxes for each test.)

^{123}I-MIBG myocardial scintigraphy	□ Consent □ No consent
DNA and RNA tests, metabolome analysis	□ Consent □ No consent

(Patient) I understand that I can withdraw this consent at any time.

Consent date: MM/DD/YYYY ____________________

Name (signature): ____________________

(Study doctor)

Explanation date: MM/DD/YYYY ____________________

Department: ____________________

Name: ____________________

(Explanation assistant)

Explanation date: MM/DD/YYYY ____________________

Name: (Affiliation) ____________________

(Copy) For the patient

Consent Form

Dear Director of XX Hospital

I hereby give voluntary consent to participate in the study titled "Double-blind, placebo-controlled study to evaluate the change in the number of severe arrhythmias after empagliflozin intervention in patients with an implanted arrhythmia treatment device complicated by type 2 diabetes mellitus" (EMPA-ICD) after receiving a thorough explanation about the following contents of the patient information sheet from my study doctor and fully understanding the explanation and contents regarding my participation in the study:

- A Certified Review Board reviewed the study.
- The study was approved by the Certified Review Board, and the protocol was submitted to the Ministry of Health, Labour and Welfare.
- Purpose of the study
- Summary of the study
- How the samples and data will be used and how long they will be stored
- Expected advantages and possible risks of participation in the study
- Expected effects and adverse reactions, and disadvantages to patients
- Other treatments
- Actions after completion of the clinical study
- My decision to participate in the study is voluntary
- Protection of my human rights
- What I will have to do
- Study-related costs for patients
- Compensation for the study
- Possible conflicts of interest
- Monitoring and auditing
- Disclosure of the study information
- Contacts for the study

* Optional tests (Please check one of the following boxes for each test.)

^{123}I-MIBG myocardial scintigraphy	□ Consent □ No consent
DNA and RNA tests, metabolome analysis	□ Consent □ No consent

(Patient) I understand that I can withdraw this consent at any time.

Consent date: MM/DD/YYYY ____________________

Name (signature): ____________________

(Study doctor)

Explanation date: MM/DD/YYYY ____________________

Department: ____________________

Name: ____________________

(Explanation assistant)

Explanation date: MM/DD/YYYY ____________________

Name: (Affiliation) ____________________

*** Consent Withdrawal Form ***

Dear Director of XX Hospital

I hereby wish to WITHDRAW my consent to participate in the study titled "Double-blind, placebo-controlled study to evaluate the change in the number of severe arrhythmias after empagliflozin intervention in patients with an implanted arrhythmia treatment device complicated by type 2 diabetes mellitus" (EMPA-ICD).

If there are any anonymized registered data (including data from the performed tests):

- ☐ I approve the use of such data.
- ☐ I do not approve the use of such data.

(Patient)

Consent withdrawal date: MM/DD/YYYY

Name (signature): ____________________

I confirm the withdrawal of the consent.

(Study doctor)

Consent withdrawal confirmation date: MM/DD/YYYY ________

Department: ____________________

Name: ____________________

Name: (Affiliation) ____________________

Information on Adverse Reactions

Comparison of empagliflozin or placebo for prevention of lethal ventricular arrhythmia in type 2 diabetic patients with implantable cardioverter defibrillators (ICD)

EMPA-ICD

This document describes major adverse reactions that may be caused by treatment with the investigational drug. Rarely, some adverse reactions may lead to severe symptoms. Please read through this document, and **immediately contact your study doctor if you are worried that you have any of the symptoms described in this document or other symptoms.** Your study doctor will change your treatment or appropriately treat your symptoms.

If there are any words in this document that you don't understand or if you have any questions, please feel free to contact us.

EMPA-ICD Study Group

Prepared on: October 17, 2018 (ver. 1.0)

1. Hypoglycemia

A condition in which your blood sugar (glucose) level is lower than normal because of the effect of the investigational drug.

<Major symptoms>

Hunger, palpitation, light-headed feeling, headache, dizziness/vertigo, cold sweat

<Actions to be taken and points to bear in mind>

- If you have any of the above symptoms, it is important to first take sugar/glucose*.
  - * If you are taking any alpha-glucosidase inhibitor, please take glucose and not sugar.
- If the symptoms do not get better, please immediately contact your study doctor.
- The combination the study drug with another drug you are taking may cause hypoglycemia. Therefore, if you are taking any other drugs, please inform your study doctor of the name of the drugs.

2. Dehydration

The investigational drug may increase the frequency of urination or volume of urine, possibly causing dehydration.

<Major symptoms>

Thirst, lethargy, dizziness/vertigo, low urine volume

<Actions to be taken and points to bear in mind>

- To prevent dehydration, please be careful to drink water frequently while taking the investigational drug. Please do not decide to stop drinking water.
- If any of the following conditions apply to you, please pay special attention to the onset of symptoms of dehydration:
  - Participating in the study during a high temperature season
  - Diarrhea or vomiting
  - Poor glucose control
  - Older person
  - Taking a diuretic agent
  - Renal impairment

3. Ketoacidosis

<Major symptoms>

Nausea and vomiting, decreased appetite, abdominal pain, abnormal thirst, lethargy, shortness of breath (dyspnea), consciousness disorder

<Actions to be taken and points to bear in mind>

- If you experience any of the above symptoms, please immediately visit a medical institution.
- Please avoid excessively restricting sugar, that means do not excessively restrict carbohydrate-containing foods such as rice, breads, and noodles.
- If you meet any of the following conditions, please pay special attention to the symptoms listed above:
  - Recent dose reduction or discontinuation of insulin preparations
  - A doctor found reduced insulin secretion function
  - Older person
  - Taking a diuretic agent
  - Renal impairment
  - Reduced food intake
  - Infection
  - Likely to be dehydrated

4. Urinary tract infection, genital infection

Because the investigational drug reduces blood glucose levels by causing glucose to be excreted in the urine, urinary tract and genital infections may be likely to occur.

<Major symptoms>

Urinary tract infection: Increased urination frequency, pain during urination, residual urine

Genital infection: Change in color or odor of vaginal discharge (in women), genital itching (pruritus)

<Actions to be taken and points to bear in mind>

Because the above symptoms may lead to serious infections, please be careful to do the following things to prevent infections:

- Do not avoid urinating.
- Keep the genital area clean.
- Be careful to always be fully hydrated.

5. Skin symptoms

Skin symptoms may be caused by an allergy to the investigational drug.

<Major symptoms>

Redness, itchiness, acne-like rash

<Actions to be taken and points to bear in mind>

Because the above symptoms may lead to major adverse reactions, please immediately contact your study doctor if you notice any skin symptoms.

(For patients)

Ver. 1.0

Prepared on: October 17, 2018

Summary of Compensation System for the Study

This study will be conducted carefully, but nevertheless we have prepared a compensation system in case of injury caused by adverse reactions, etc. This document aims to elaborate the information on the compensation for the study described in the Informed Consent Form. Please keep this document in a safe place along with the patient information sheet and a copy of the consent form.
If you experience any symptom you have never had before, get sick or notice anything different while you are participating in the study, please quickly contact your study doctor, who will immediately provide you with appropriate treatment.

1. Principle of compensation

1) Only the injury caused by (participation in) the study that is causally related to the study will be compensated, and any third party will not be liable for damages resulting from the study.
2) Even after signing the study consent form, you may file a lawsuit claiming liability for damages if such liability arises. This compensation system will not preclude your right to claim liability for damages.

2. Events that will not be compensated

1) Events will not be compensated for which there is no direct causal relationship between the study and the injury, such as a traffic accident on the way to the hospital. The person responsible for causing the injury will be liable for damages resulting from such events.
2) Events whose causal relationship with the study is ruled out, such as those that have a clear causal relationship with something else, those for which the temporal relationship between the use of the study drug and the injury makes it unreasonable to expect that the injury resulted from the study, and those that are irrational.
3) You will not be compensated if you do not receive a therapeutic benefit because of “no effect of the investigational product,” etc.

3. Limited compensation

1) If you acted with gross negligence, such as making a false declaration and not following the study doctor’s instructions, you will not be compensated or will be only partly compensated.

4. Compensation payment

If you have any Grade of Disability grade 1, 2 or 3 sequelae, as specified in the National and Employee's Pension Insurance Systems, or if you die, the amount we will pay will be calculated depending on the type and degree of disability and by referring to the amounts of benefits permitted under such systems.

5. Claiming compensation

1) If you suffer any injury, please contact your study doctor.
2) Please note that for compensation payment you may be asked to submit necessary documents, including a copy of your health insurance card.
3) In principle, compensation will be transferred to the bank or post office account designated by you. Please note that it usually takes 2 to 3 months to complete the transfer procedures.

If you have any questions about the compensation system, please feel free to contact your study doctor.

Procedures for Special Blood Tests

Comparison of empagliflozin or placebo for prevention of lethal ventricular arrhythmia in type 2 diabetic patients with implantable cardioverter defibrillators (ICD)

EMPA-ICD

Representative Investigator: Tohru Minamino
Professor, Department of Cardiovascular Medicine, Niigata University Graduate School of Medical and Dental Sciences

1 : Purpose and scope of the procedures

This document describes the procedures for performing special blood tests at each study site during the study.

2 : Schedule for special blood tests

	Baseline		Treatment period		
	Eligibility screening	Visit 1	Visit 2	Visit 3	At the time of discontinuation
	Week -12 to 0	Week 0	Week 12	Week 24	
		–	(± 4 weeks)	(± 4 weeks)	
**Hematological tests (special)**		○*		○	△

○, Essential; △, Optional

Wherever possible, fasting blood will be collected after patients have rested in supine position on the examination table for 30 minutes at Week 0* (baseline) and Week 24 (or the time of discontinuation).

*Results of tests performed between informed consent and Week 0 will be used.

3 : Special blood test endpoints

(1) Blood catecholamine fraction: adrenalin, noradrenalin, dopamine

(2) Blood ketone body fraction: acetoacetic acid, 3-hydroxybutyric acid, total ketone bodies

(3) Erythropoietin

(4) Reticulocyte count

(5) DNA tests: telomere length, G-tail length (no genetic analysis)

(6) RNA tests: P53, P21, P16

(7) Metabolome analysis

4 : Procedures for performing special blood tests

*** For blood sampling, please use the spitz tubes and the tubes for submission that were delivered to the study sites before the study.**

(1) Blood catecholamine fraction (essential)

[Spitz tube to be used: EDTA-2Na]

■ Procedures at each study site:

[1] Collect venous blood (one 7-mL tube) from the upper arm of the patient.

[2] Mix well and promptly separate the plasma by centrifuging at 2,000 rpm for 10 minutes

at a low temperature (4°C).

[3] Transfer the supernatant into a designated container (poly-spitz tube pre-labeled for submission to SRL) and store frozen at -20°C.

[4] Tubes will be collected by SRL.

Test item	Sampling volume	Tube for sampling	Treatment before submission	Tube for submission	Volume to be submitted	Submission and storage
**3 fractions of catecholamines**	Whole blood 7 mL	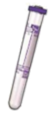 Purple cap (with EDTA-2Na)	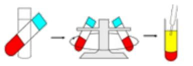 ・After sampling, mix well and promptly separate the plasma by centrifuging approximately at 2,000 rpm for 10 minutes at low temperature (4°C).<br>・Transfer the supernatant into a designated container (poly-spitz tube pre-labeled for submission to SRL) and store frozen at -20°C.	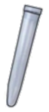	Plasma 1.5 mL	**Frozen**

(2) Blood ketone body fraction (essential)

(3) Erythropoietin (essential)

[Spitz tube to be used: Serum Separator Tube AutoSep]

■ Procedures at each study site ([1] to [3] for both blood ketone body fraction and erythropoietin)

[1] Collect venous blood (one 5-mL tube) from the upper arm of the patient.

[2] After sampling, promptly centrifuge with cooling at 2,000 rpm for 10 minutes.

[3] Wait for clot retraction under refrigeration if possible and then separate the serum within 2 hours.

■ For blood ketone body fraction:

[4] Transfer 0.3 mL of the supernatant into a designated container (poly-spitz tube pre-labeled for submission to SRL) and store frozen at -20°C.

[5] Tubes will be collected by SRL.

■ For erythropoietin:

[4] Transfer 0.8 mL of the supernatant into a designated container (poly-spitz tube pre-labeled for submission to SRL) and store refrigerated.

[5] Tubes will be collected by SRL.

Test item	Sampling volume	Tube for sampling	Treatment before submission	Tube for submission	Volume to be submitted	Submission and storage

Test item	Sampling volume	Tube for sampling → Treatment before submission → Tube for submission	Volume to be submitted	Submission and storage
**Blood ketone body fraction**	Whole blood 5-4 mL	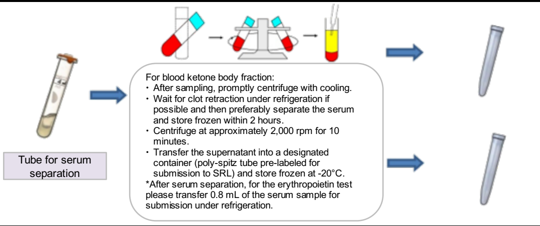	Serum 0.3 mL	**Frozen**
**Erythropoietin**			Serum 0.8 mL	**Refrigerated**

* Blood ketone body fraction ⇒ Because acetoacetate is unstable, please separate the serum and, after completion of clot retraction, store it frozen wherever possible.

(4) Reticulocyte count (essential)

[Spitz tube to be used: EDTA-2K]

■ Procedures at each study site:

[1] Collect venous blood (one 2-mL tube) from the upper arm of the patient.

[2] Store refrigerated.

[3] Tubes will be collected by SRL.

Test item	Sampling volume	Tube for sampling → Treatment before submission → Tube for submission	Volume to be submitted	Submission and storage
**Reticulocyte count**	Whole blood 2 mL	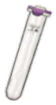 Collect into and submit in a pre-labeled tube. Purple cap (with EDTA-2K)	Whole blood 2 mL	**Refrigerated**

(5) DNA tests: telomere length, G-tail length (optional)

[Spitz tube to be used: EDTA-2Na tube (5 mL)]

■ Procedures at each study site:

[1] Collect venous blood (two 5-mL tubes) from the upper arm of the patient.

[2] Store the spitz tube frozen at -20°C (no sample transfer).

[3] Send **frozen** within 3 days (not including the sampling day).

■ Procedures at the Department of Cardiovascular Biology and Medicine, Division of Molecular Aging and Cell Biology, Niigata University:

[1] Extract genomic DNA with Puregene Blood Core Kit (Qiagen).

[2] In obtained DNA (1 ng/uL), measure telomere length with LightCycler 480 (Roche Applied Science), Light Cycler 480 SYBR Green I Master (Roche), and the following primer:

[Primer]

Telomere: CGGTTTGTTTGGGTTTGGGTTTGGGTTTGGGTTTGGGTT,
GGCTTGCCTTACCCTTACCCTTACCCTTACCCTTACCCT
36B4; CAGCAAGTGGGAAGGTGTAATCC, CCCATTCTATCATCAACGGGTACAA

*** Do not perform analysis of genetic information.**

(6) RNA tests: P53, P21, P16 (optional)

[Spitz tube to be used: PAXgene tube (BD)]

- Procedures at each study site:

[1] Collect venous blood (two 2.5-mL tubes) from the upper arm of the patient.
[2] Let stand for 2 hours or more at room temperature.
[3] Store the spitz tube frozen at -20°C (no sample transfer).
[4] Send **frozen** within 3 days (not including the sampling day).

- Procedures at the Department of Cardiovascular Biology and Medicine, Division of Molecular Aging and Cell Biology, Niigata University:

[1] Extract RNA with PAXgene blood RNA kit (Qiagen).
[2] Prepare cDNA from the obtained RNA with QuantiTect Reverse Transcription Kit (QIAGEN).
[3] Quantitatively evaluate senescence markers（P53, P21, P16）with Taqman Universal ProbeLibrary (Roche Applied Science) with the Light Cycler 480 Probes Master (Roche) and the following primers:

[Primers]
CDKN1A(p21); cgaagtcagttccttgtggag, catgggttctgacggacat
CDKN2A(p16); gtggacctggctgaggag, ctttcaatcggggatgtctg
Tp53; aggccttggaactcaaggat, cccttttggacttcaggtg
RPLP0(60s); gatgcccagggaagacag,acaatgaagcattttggataa

*** Do not perform analysis of genetic information.**

(7) Metabolome analysis (optional)

[Spitz tube to be used: EDTA-2Na tube (5 mL)]: VENOJECT II Evacuated Tube (Code No. VP-NA050K)

- Procedures at each study site:

[1] Collect venous blood (one 5-mL tube) from the upper arm of the patient.
[2] Mix well and promptly separate the plasma by centrifuging at 3,000 rpm for 10 minutes

at low temperature (4°C).

[3] Aliquot the supernatant into designated containers (Thermo cryotubes, 200 μL each) and store frozen at -80°C.

[4] Send **frozen** within 3 days (not including the sampling day).

■ Procedures at the Department of Cardiovascular Medicine, Division of Molecular Aging and Cell Biology, Niigata University

[1] Extract metabolites by the Metabolite Extraction Method of the Institute for Advanced Biosciences, Keio University.

[2] Store at -80°C and send frozen to the Institute for Advanced Biosciences, Keio University.

5: How to pack samples for special blood tests

For tests (5) to (7), pack as follows:

[From the top]

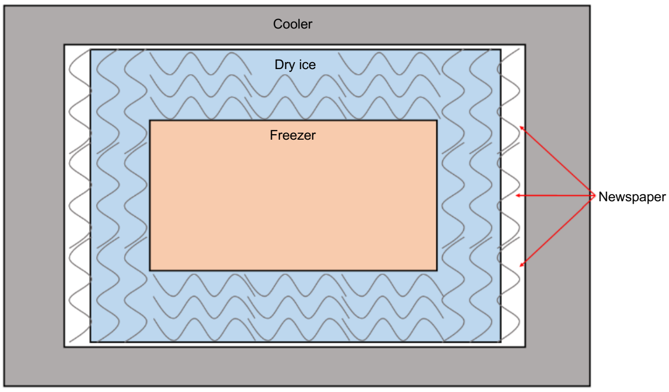

[From the side]

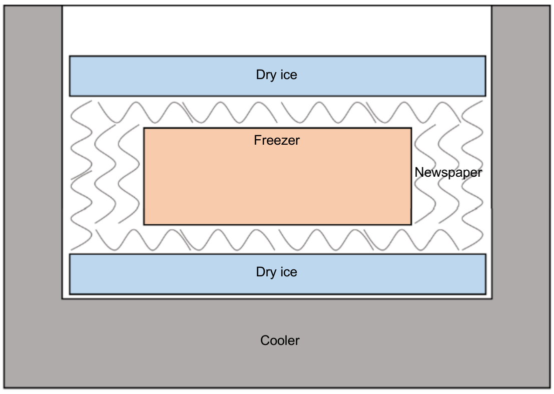

[Inside the freezer]

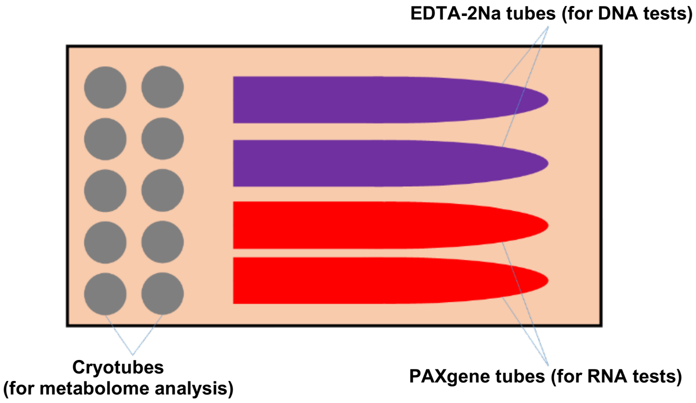

6: How to send samples of special blood tests

For tests (5) to (7), send samples to the following address and designate a delivery time in the morning:

Room 533, West Research Building, Department of Cardiovascular Medicine,<br>Niigata University Graduate School of Medical and Dental Sciences<br>Asahimachi-Dori 1-757, Chuo-ku, Niigata, Niigata 951-8510, Japan<br>Tel: 025-227-2189

Procedures for Holter Monitoring

Comparison of empagliflozin or placebo for prevention of lethal ventricular arrhythmia in type 2 diabetic patients with implantable cardioverter defibrillators (ICD)

EMPA-ICD

Representative Investigator: Tohru Minamino
Professor, Department of Cardiovascular Medicine, Niigata University Graduate School of Medical and Dental Sciences

1: Purpose and scope of the procedures

This document describes the procedures for performing Holter monitoring at each study site during the study.

2: Summary of Holter monitoring

Ventricular premature contraction (VPC) recorded by Holter monitor is a predictor of the onset of severe arrhythmia in patients with structural heart diseases, including old myocardial infarction [1] and dilated cardiomyopathy [2], and those with reduced left ventricular ejection fraction [3]. Holter monitoring will evaluate the number of VPCs not recorded by the arrhythmia device to determine the effect of the SGLT2 inhibitor.

3: Schedule for Holter monitoring

	Baseline		Treatment period		
	Eligibility screening	Visit 1	Visit 2	Visit 3	At the time of discontinuation
	Week -12 to 0	Week 0	Week 12	Week 24	
		–	(± 4 weeks)	(± 4 weeks)	
Holter monitoring		○*		○	△

○, Essential; △, Optional

To be performed at Week 0* (baseline) and Week 24 (can also be performed at the time of discontinuation).

*Results of Holter monitoring performed within 12 weeks before the test at Week 0 will be used even if they are obtained before informed consent.

4: Holter monitoring endpoints

(1) Total recording time

(2) Total number of VPCs

(3) Number of single VPCs

(4) Number of two-consecutive VPCs

(5) Number of ventricular tachycardia (at least three ventricular premature beats with a maximum mean R-R interval of 600 ms)

5: Procedures for performing Holter monitoring

・ Record with NASA and CM5 leads wherever possible.

・An experienced technician should attach electrodes and pay attention to the following points:

[1] Before attaching electrodes, pretreat with an alcohol pad or skin pretreatment to facilitate fixing and reduce skin resistance.

[2] After attaching electrodes, handle excess lead cords appropriately. Follow the procedures described in the instruction manual of each product.

[3] Determine the waveform amplitude and any murmur with the Holter monitor before starting the recording.

[4] Tell the patient about cautions to be taken during the test (for example, not using electronic devices, including electric blankets and cell phones, not touching electrodes, and not wearing clothes that are likely to cause static electricity).

・Record for 24 hours wherever possible.

・An experienced technician should scan and analyze the records.

5: How to evaluate Holter monitoring

・A cardiovascular specialist, principal investigator or subinvestigator should determine the number of recorded VPCs.

・Record the total recording time, total number of VPCs, number of single VPCs, number of occurrences of two consecutive VPCs, and number of ventricular tachycardia (at least three ventricular premature beats with a maximum mean R-R interval of 600 ms).

・Enter data in the electronic case report form.

6: References

[1] Circulation 1993; 87: 312-322.

[2] N Engl J Med 2004; 350: 2151-2158.

[3] Circulation 2000; 101: 40-46.

Procedures for Echocardiography

Comparison of empagliflozin or placebo for prevention of lethal ventricular arrhythmia in type 2 diabetic patients with implantable cardioverter defibrillators (ICD)

EMPA-ICD

Representative Investigator: Tohru Minamino
Professor, Department of Cardiovascular Medicine, Niigata University Graduate School of Medical and Dental Sciences

1: Purpose and scope of the procedures

This document describes the procedures for performing echocardiography at each study site during the study.

2: Schedule for echocardiography

	Baseline		Treatment period		
	Eligibility screening	Visit 1	Visit 2	Visit 3	At the time of discontinuation
	Week -12 to 0	Week 0	Week 12	Week 24	
		–	(± 4 weeks)	(± 4 weeks)	
**Echocardiography**	○*	○*		○	△

○, Essential; △, Optional

To be performed during eligibility screening and at Week 0* (baseline) and Week 24 (or the time of discontinuation).

*Results of tests performed within 12 weeks before the test at Week 0 will be used even if they are obtained before informed consent.

3: Echocardiography endpoints

(1) LVEF

(2) E wave

(3) A wave

(4) sep-e'

(5) lat-e'

(6) peak TRV

(7) HR

4: Procedures for performing echocardiography

Measurements should be performed by an experienced technician according to the updated ASE guidelines [1] [2] and as described below. Compliance with these instructions should be confirmed by a cardiovascular specialist or the principal investigator or a sub-investigator.

<table>
<tr><th colspan="2">Left ventricular ejection fraction (%EF)</th></tr>
<tr><td>To be measured from the apical four- and two-chamber views by using the biplane disk summation (modified Simpson) method.
If no asynergy is present in the left ventricle, the measurement may be performed with the apical four-chamber view by the single-plane disk summation method.
The volume and %EF calculated from the left ventricular diameter obtained from the left ventricular long axis view cannot be substituted.</td><td>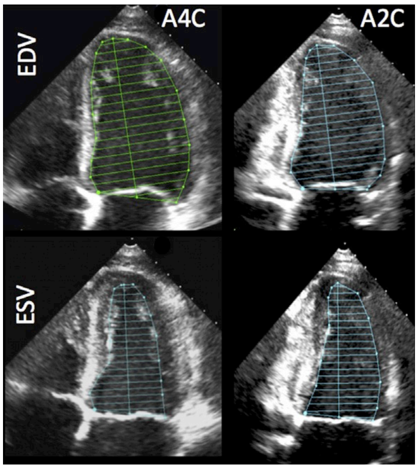
</td></tr>
<tr><th colspan="2">Left atrial volume (LAV)</th></tr>
<tr><td>To be measured at end systole (when the left atrial chamber is at its greatest dimension) by using the biplane disk summation method on the apical four- and two- chamber views.</td><td>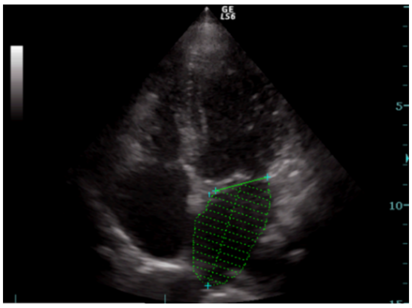
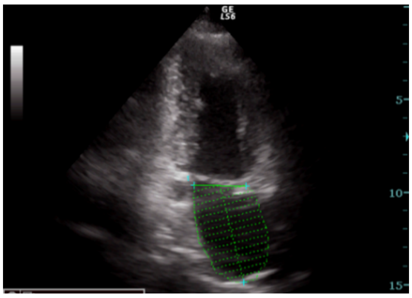</td></tr>
</table>

<table>
<tr><th colspan="2">Transmitral flow velocity (E, A)</th></tr>
<tr><td>On the apical left ventricular long-axis view, a sample volume (preferably 3 mm) should be placed at the tip of the mitral valve at the maximum mitral valve opening in the early diastolic filling phase, and the center of the axis of blood flow into the left ventricle should be observed by color Doppler.
At the end of expiration during normal breathing, record stable velocity patterns for at least three beats; one of these recordings should be used to measure the maximal velocity at early diastole (E) and atrial contraction (A).</td><td>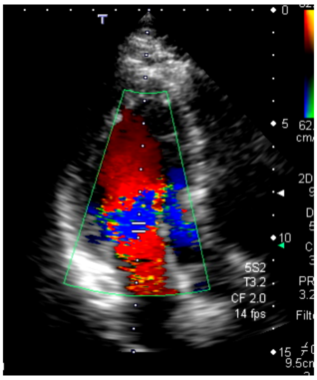

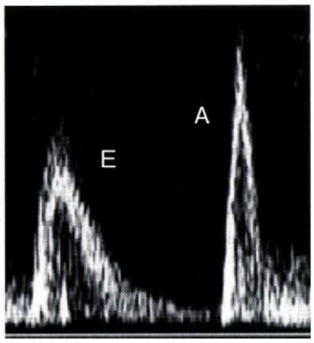
</td></tr>
<tr><th colspan="2">Velocities of mitral annulus (lat-e', sep-e')</th></tr>
<tr><td>Early diastolic velocity (e’) of the septal and lateral mitral annulus should be measured by tissue Doppler imaging in the apical four-chamber view, where the ultrasound beam is positioned parallel to the direction of the mitral annular motions, preferably with a 10-mm sample volume.

When using the device without the tissue Doppler imaging mode, the velocities of mitral annulus should be measured by pulsed Doppler imaging for blood flow at lower Doppler gain, minimum filter, and a velocity range of approximately 20 to -20 cm/sec.</td><td>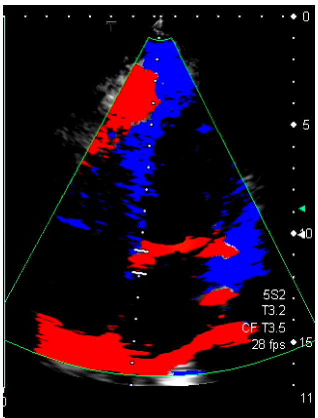

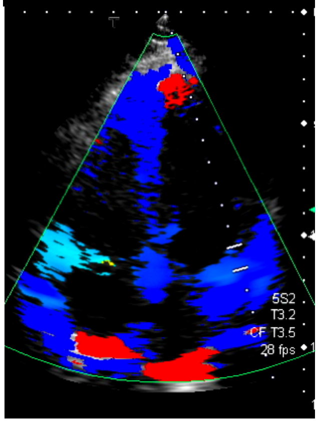
</td></tr>
</table>

	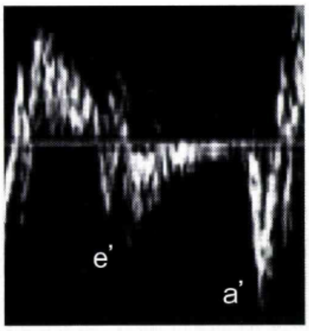
**Tricuspid regurgitation peak velocity (peak TRV)**	
On the apical four-chamber view or the parasternal view (aortic valve level), a sample volume (preferably 3 mm) should be placed at the center of the axis of tricuspid regurgitation observed by color Doppler.<br>At the end of expiration during normal breathing, record stable velocity patterns for at least three beats; one of these recordings should be used to measure the maximal velocity of tricuspid regurgitation (TR).	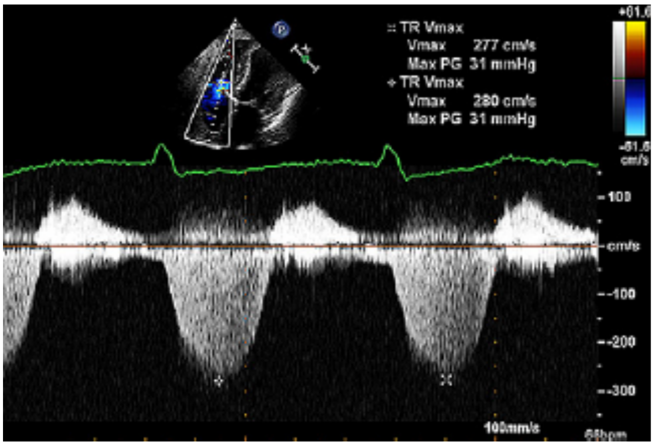
**Heart rate (HR)**	
Record mean heat rate during echocardiography.<br>* For atrial fibrillation, use the mean of three heart rates with an average R-R interval.	

5: References

[1] J Am Soc Echocardiogr 2015;28:1-39.

[2] J Am Soc Echocardiogr 2016;29:277-314.

Procedures for ^{123}I-MIBG Myocardial Scintigraphy

Comparison of empagliflozin or placebo for prevention of lethal ventricular arrhythmia in type 2 diabetic patients with implantable cardioverter defibrillators (ICD)

EMPA-ICD

Representative Investigator: Tohru Minamino
Professor, Department of Cardiovascular Medicine, Niigata University Graduate School of Medical and Dental Sciences

1: Purpose and scope of the procedures

This document describes the procedures for performing ^{123}I-MIBG myocardial scintigraphy at each study site during the study.

2: Summary of ^{123}I-MIBG myocardial scintigraphy

3(meta)-iodobenzylguanidine (MIBG), which is used in ^{123}I-MIBG myocardial scintigraphy, is an imaging agent for nerve function that has the same dynamics as noradrenaline at sympathetic nerve endings. Thus, the degree of myocardial accumulation of MIBG reflects myocardial sympathetic nerve ending function and can be used to evaluate abnormal sympathetic nerve function, such as myocardial denervation. Various reports [1]-[5] indicate that MIBG can predict fatal arrhythmia and cardiac sudden death, so it is useful to periodically perform the test to evaluate change over time in the predicted adverse cardiovascular events [6]. The test is also used to determine therapeutic effects of drugs [7][8], and guidelines indicate its usefulness [9]. Therefore, in clinical practice it appears to be helpful to repeat the test to predict patient prognosis and evaluate treatment effects over time. Although it has been suggested that the difference in the camera or collimator causes site error, a standardization method has been established for determining measured values [10].

3: Schedule for ^{123}I-MIBG myocardial scintigraphy

	Baseline		Treatment period		
	Eligibility screening	Visit 1	Visit 2	Visit 3	At the time of discontinuation
	Week -12 to 0	Week 0	Week 12	Week 24	
		–	(± 4 weeks)	(± 4 weeks)	
^{123}I-MIBG myocardial scintigraphy		△*		△	△

Can be performed at Week 0* (baseline) and Week 24 (or the time of discontinuation). The test is optional at both time points.

*Results of the test performed within 12 weeks before the test at Week 0 will be used even if they are obtained before informed consent.

4: ^{123}I-MIBG myocardial scintigraphy endpoints

(1) Heart-to-mediastinum ratio (HMR)

(2) Washout rate (WR)

5: Procedures for performing ^{123}I-MIBG myocardial scintigraphy

- A digital gamma camera with a widely used low-energy collimator should be prepared for imaging supine patients from an antero-posterior direction with the entire chest included in the field of view.
- Inject 111 MBq of ^{123}I-MIBG as an intravenous bolus into the cubital vein. Adjust the dose depending on the patient's age and weight, as applicable.
- Acquire the anterior (early) view 15 minutes after nuclide injection (acquisition time according to local conditions).
- Acquire the anterior (late) view 3 to 6 hours after nuclide injection (acquisition time according to local conditions).
- Whenever possible, patients should not use droxidopa, reserpine, tricyclic antidepressants, or labetalol hydrochloride because these drugs limit accumulation of ^{123}I-MIBG to the heart.
- A SPECT result report is not required, but it may be performed at the discretion of each site if needed.

[Example]

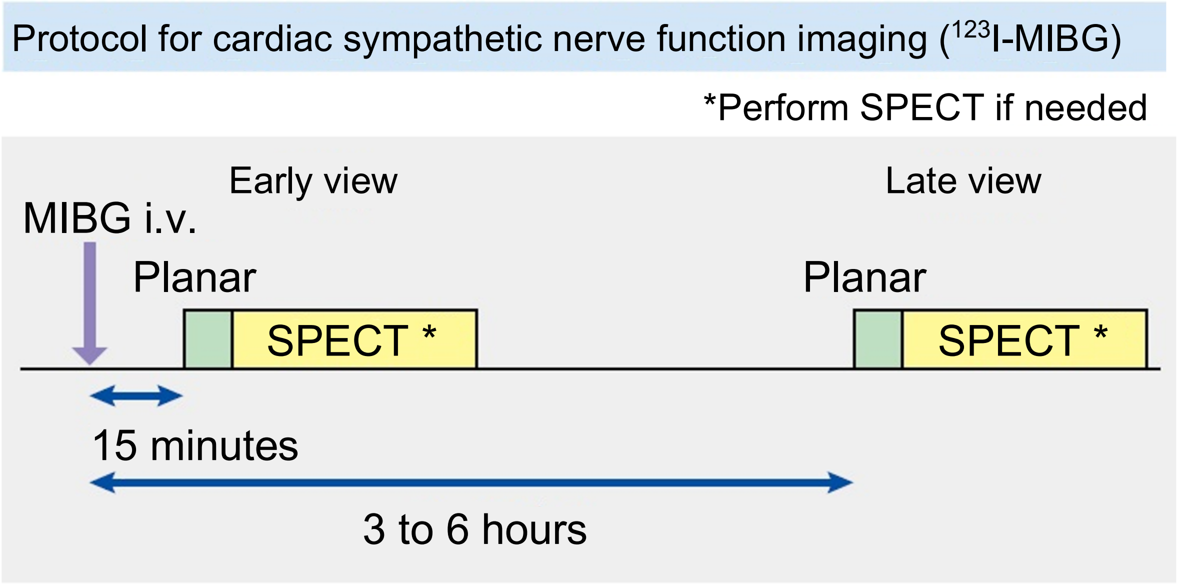

6: How to evaluate ^{123}I-MIBG myocardial scintigraphy

・How to evaluate: With the mediastinum as the background, set the region of interest in the heart (H) and the mediastinum (M). Determine the number of counts per pixel, measure the heart-to-mediastinum ratio (HMR) as a measure of myocardial accumulation and calculate the washout rate (WR) as a measure of the rate of release from the myocardium.
・Prepare the report with smartMIBG Heart (Fuji Film RI Pharma Co. Ltd.) [4].
・Report (1) the heart-to-mediastinum ratio (early and late views) and (2) the washout rate with the electronic data capture (EDC) system for the case report form (CRF).

7: References

[1] J Nucl Med 2009; 50: 61-67.
[2] Heart 2007; 93: 1213-1218.
[3] Nucl Med 2008; 49: 225-233.
[4] Eur J Nucl Med Mol Imaging 2006; 33: 866-870.
[5] J Am Coll Cardiol 2009; 53: 426-435.
[6] J Nucl Med 2008; 49: 907-914.
[7] Am Heart J 1997; 133: 353-358.
[8] J Nucl Cardiol 2001; 8: 4-9.
[9] Guidelines for Clinical Use of Cardiac Nuclear Medicine (JCS 2010)
[10] J Nucl Cardiol 2014; 21: 970-978.

Department of Cardiovascular Medicine, Niigata University Graduate School of Medical and Dental Sciences

Adverse events reporting procedures

Comparison of empagliflozin or placebo for prevention of lethal ventricular arrhythmia in type 2 diabetic patients with implantable cardioverter defibrillators (ICD)

Representative Investigator: Tohru Minamino, Department of Cardiovascular Medicine, Niigata University Graduate School of Medical and Dental Sciences, Niigata, Japan

Ver.1.2

9/28/2018

Contents

1. Purpose and scope of the document 2
2. Definition of adverse events in the study (Figure 1) 2
3. Definition and role of each person involved in the study 6
4. Reportable events 7
5. Procedures for reporting AEs 8
6. Where to report events that require expedited reporting 13
7. Breaking emergency key code 13
8. Storage of materials 13
9. History of changes 13

1. Purpose and scope of the document

This document defines adverse events and, to help standardize procedures, describes the role and workflow of people involved in the study.

2. Definition of adverse events in the study (Figure 1)

Adverse Event (AE)

An adverse event is any unfavorable or unintended disease or sign (e.g., an abnormal laboratory finding) in participants, regardless of the causal relationship with the study.

(1) Serious Adverse Event (SAE)

A Serious Adverse Event (SAE) is:

1) an AE that results in death,
2) a life-threatening AE,
3) an AE that requires hospitalization for treatment or prolongation of an existing hospitalization,
4) an AE that results in persistent or significant disability or incapacity,
5) an AE that results in a congenital anomaly, or
6) another event or reaction considered medically significant.

The phrase “considered medically significant” in point 6) above refers to an event that is appropriately medically evaluated as endangering the patient and requiring medical or surgical treatment so that it does not lead to an SAE as defined in points 1) to 5) above.

Patients may be hospitalized during the study period for study management or for social reasons (e.g., for fluid infusion if the hospital is too far away for the patient to visit the hospital for treatment and return home the same day). If hospitalization for such reasons was scheduled at initiation of the study, it was already recorded in the source document at the time of the eligibility screening tests. Therefore, these hospitalizations do not have to be reported as SAEs if they are conducted as scheduled.

(2) AEs of Special Interest

The term AE of Special Interest (AESI) refers to any specific AE that has been identified at the project level as being of particular concern for prospective safety monitoring and safety assessment during the study, e.g., potential AEs on the basis of knowledge from other compounds in the same class. Principal Investigators must report AESIs to the Representative Investigator within the same timeframe that applies to SAEs. The definitions of the AESIs in this study are as follows:

1) Liver disorder

A liver disorder is defined as the following changes in liver function:

AST and/or ALT increased **≥ 3-fold** above the upper limit of local laboratory reference ranges plus total bilirubin measured in the same blood sample increased **≥ 2-fold** above the upper limit of local laboratory normal ranges

AND/OR

AST and/or ALT increased **≥ 5-fold** above the upper limit of local laboratory normal ranges

The above findings are warning signs for a liver disorder. In patients with such laboratory abnormalities, the Principal Investigator or Subinvestigator should collect samples for measuring liver function test values as early as possible and follow up the liver function according to their clinical decision.

If a participant experiences any clinical symptom of liver disorder (jaundice, encephalopathy of unknown cause, coagulopathy of unknown cause, right upper abdominal pain, etc.) without laboratory test results (ALT, AST, total bilirubin), a Principal Investigator or Subinvestigator should perform the necessary blood tests to evaluate the above liver function test values.

2) Decreased kidney function

Decreased kidney function is defined as creatinine levels **≥ 2-fold** above the level at Week 0 and above the normal upper limit.

In patients with decreased kidney function, the Principal Investigator or Subinvestigator should collect samples for measuring creatinine as early as possible and follow up the kidney function according to their clinical decision.

3) Acidosis

If metabolic acidosis, ketoacidosis, or diabetic ketoacidosis (DKA) is suspected, the patient should be closely examined according to the clinical decision and clinical course until a diagnosis has been established and/or the patient has recovered.

According to the Japanese Clinical Practice Guideline for Diabetes 2016 of the Japan Diabetes Society, DKA is **a combination of hyperglycemia (glucose ≥ 250 mg/dL), hyperketonemia (elevation of beta-hydroxybutyric acid), and acidosis (pH < 7.3 and bicarbonate < 18 mEq/L)** resulting from extreme insulin deficiency and an increase of counterregulatory hormones that affect insulin.

A diagnosis of DKA does not require all of the above criteria to be met and should be based on a clinical decision. The mechanism of action of empagliflozin may change the clinical symptoms of DKA and may be associated with an onset of DKA at a lower glucose level than the above values.

4) Events associated with leg amputation

Events associated with leg amputation are defined as amputation (through the bones), disarticulation (through the joints), and autoamputation (spontaneous detachment of the part of the lower extremities that can no longer exist) and do not include other treatments without debridement (removal of callus or necrotic tissue); treatment of a stump (repair of stump, abscess drainage, wound repair, etc.); or amputation or disarticulation of extremities, such as nail excision and avulsion.

Leg amputation, disarticulation, and autoamputation should be individually reported. The report should describe the treatment date, level of amputation or disarticulation, medical condition leading to the relevant treatment, and, if applicable, any known risk factor for leg amputation in the patient.

5) Necrotizing fasciitis of the perineum (Fournier's gangrene)

Cases of necrotizing fasciitis, a serious infection that causes necrosis of the subcutaneous tissue, that occur in the perineum are called Fournier's gangrene. Physicians should pay attention to symptoms such as genital or perineal pain or tenderness, erythema or swelling, and fever or malaise because they may indicate suspected Fournier's gangrene.

Patients should be treated promptly because serious outcomes have been reported.

(3) Disease or the like

Among the AEs, those possibly related to the research are referred to as “disease or the like.”

A “disease or the like” set forth in Article 54 of the regulations for enforcing the Clinical Trials Act (hereafter called “Serious Diseases”) is reportable to the research institution administrator and the Certified Review Board.

(4) Events that require expedited reporting

In the study, the events that require expedited reporting are SAEs, AESIs, and Serious Diseases.

(Figure 1: Definition of adverse events)

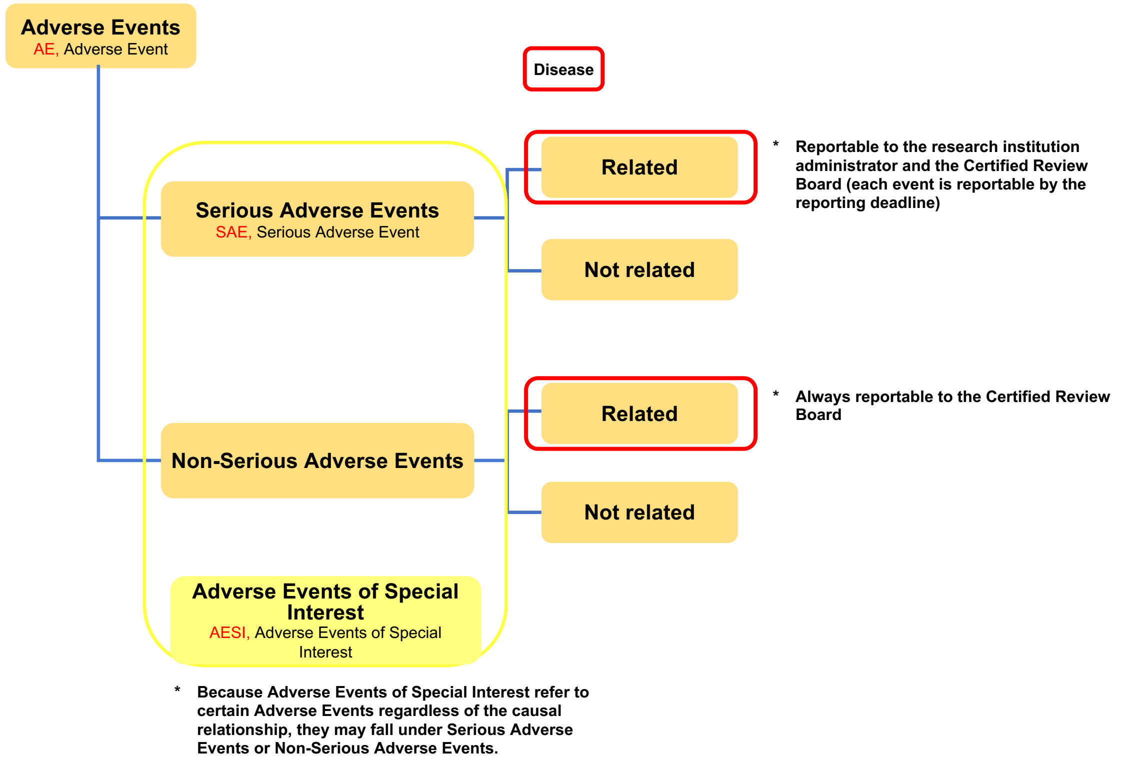

* **If an Adverse Event belongs to multiple categories, it should be reported according to the most serious category.**

<Serious Adverse Event>

[1] Adverse Event that results in death
[2] Life-threatening Adverse Event
[3] Adverse Event that requires hospitalization for treatment or prolongation of existing hospitalization
[4] Adverse Event that results in persistent or significant disability or incapacity
[5] Adverse Event that results in a congenital anomaly
[6] Other event or reaction that is considered medically significant

* Serious illnesses shall be specified in Article 54 of the Enforcement Regulations of the Clinical Trials Act.

<Adverse Event of Special Interest>

[1] Liver disorder:
- a) AST and/or ALT increased ≥ 3-fold and total bilirubin increased ≥ 2-fold (relative to the upper limit of local laboratory reference ranges [ULLN])
- b) AST and/or ALT increased ≥ 5-fold (relative to ULLN)

[2] Decreased kidney function: creatinine ≥ 2-fold above Week 0 and beyond the normal upper limit
[3] Acidosis: metabolic acidosis, ketoacidosis, and diabetic ketoacidosis
[4] Leg amputation: amputation, disarticulation, and autoamputation
[5] Necrotizing fasciitis of the perineum and genitalia (Fournier's gangrene)

3. Definition and role of each person involved in the study

(1) Representative Investigator

Tohru Minamino, Department of Cardiovascular Medicine, Niigata University Graduate School of Medical and Dental Sciences, Niigata, Japan

The Representative Investigator of the study is responsible for maintaining and storing detailed records, such as electronic case report forms (electronic data capture, hereafter called "EDC") for all reported AEs associated with the study. In addition, to enforce the Clinical Trials Act, he will report AEs to the Certified Review Board in compliance with Article 54 of the regulations. If he receives any notification of a disease or the like from the Principal Investigator of a research institution, he will immediately inform the Principal Investigators of the other research institutions participating in the study.

In accordance with the recommendations of the Data and Safety Monitoring Board, he will notify all Principal Investigators participating in the study about safety information obtained during the study (other than information on a disease or the like).

(2) Research institutions:

The institutions that enroll patients in the study have the responsibility and obligation to collect and report all AEs that occur during the study (including SAEs/AESIs, other non-serious AEs, and diseases or the like).

(3) Principal Investigator:

The physician who supervises study operations at participating research institutions. For all AEs that occur at the research institution, the Principal Investigator is responsible for preparing, appropriately reporting, maintaining and storing detailed records, including EDC, etc. The Principal Investigator will report diseases or the like to the research institution administrator. The Principal Investigator will promptly notify the Representative Investigator of events that require expedited reporting, as specified for the study. If the Principal Investigator receives information from the Representative Investigator on a disease or the like at another research institution, he/she will promptly report the content of the provided information to the research institution administrator.

(4) Research office:

The office organized by the Representative Investigator. It is responsible for operations related to administrative research procedures in general, including those for AE reporting at the Department of Cardiovascular Medicine, Niigata University Graduate School of Medical and Dental Sciences.

(5) Assistant research office:

Clinical Research Department, Micron, Inc.

The assistant research office will support the Representative Investigator and the research office in study operations, including those related to AE reporting.

(6) Designated contact person for safety:

This person will be designated by the Representative Investigator and will be the contact for all safety-related communications and data and information exchange under the pharmacovigilance agreement executed between Niigata University and Nippon Boehringer Ingelheim Co., Ltd.

(7) Pharmacovigilance Department, Nippon Boehringer Ingelheim Co., Ltd:

The department in charge of safety operations management at the company funding the study. The department will be the contact for all safety-related communications and data and information

exchange.

(8) Datacenter:
Center for Clinical Research and Innovation (CCRI), Osaka City University Hospital, Osaka, Japan
The division in charge of data management for the study. It will collect information on AEs by EDC.

(9) Data and Safety Monitoring Board:
The board will monitor and evaluate the progress of the study and safety data, etc., and, as a third party, recommend to the Representative Investigator to continue, modify, or discontinue the study.

4. Reportable events

Reportable AEs

AEs that occur during the study between informed consent and Week 24 (or the time of discontinuation) are reportable.

Information to be recorded

In case of AEs, the Principal Investigator or Subinvestigator should record the following items:

(1) Name of AE and onset date
(2) Severity
(3) Whether it is an AESI
(4) Treatment and outcome, and date outcome is confirmed
(5) Causal relationship to the study
(6) Administration status of study drug (continued or discontinued)

[Outcome]

Outcome should be categorized into one of six categories: Recovered, Recovering, Not recovered, With sequelae, Death, and Unknown. In the cases of Recovered, Recovering, or Death, the date when outcome is confirmed is the date when the outcome was actually determined. If follow-up is discontinued because the outcome is Not recovered or Unknown, the date outcome is confirmed is the date when the Principal Investigator or Subinvestigator last confirms the outcome; the reasons for discontinuing follow-up or Unknown should be written in the comment section. If any disease or the like occurs, the patient should be followed until the outcome is determined.

[Causal relationship to study]

The causal relationship to study should be assessed in the two categories described below. If it is evaluated as “Able to be ruled out,” the reason should be written in the comment section.

[1] Causal relationship can be ruled out: Cases where a temporal relationship between the AE and study is unreasonable or where there is a medical explanation for the AE onset other than the study.

[2] Causal relationship cannot be ruled out (possibly related): In cases where the case [1] does not

apply, pursuant to the Clinical Trials Act, AEs whose causal relationship to the study cannot be ruled out are defined as "disease or the like."

5. Procedures for reporting AEs

All AEs

Record all AEs associated with the study in EDC, etc.

The AE reporting procedures specified in the Clinical Trials Act and specific for the study are described below. For AEs that occur during the study, reporting under the safety information reporting system for pharmaceuticals and medical devices and spontaneous reporting to the marketing authorization holder should be done appropriately, as specified by each research institution.

(1) Events that require expedited reporting

If a Principal Investigator or Subinvestigator notices the onset of any event that requires expedited reporting at the research institution, the investigator should promptly report it to the Representative Investigator. The Representative Investigator should report it according to each regulation. In the study, events that require expedited reporting refer to Serious Diseases, SAEs, and AESIs.

1) Serious Diseases

If the Representative Investigator is notified of Serious Diseases, in compliance with Article 54 of the regulations for enforcement of the Clinical Trials Act the investigator should promptly inform the Principal Investigators at the other research institutions and report the event to the Certified Review Board. The date of awareness is the date on which the Principal Investigator or Subinvestigator becomes aware of the onset of the event.

<table>
<tr><th></th><th>Expectedness
(*)</th><th>Seriousness</th><th>Reportable within</th></tr>
<tr><td rowspan="5">Other than the study with unapproved or off-label use of pharmaceuticals</td><td>Regardless of expectedness</td><td>Death (excluding infections)</td><td>15 days</td></tr>
<tr><td>Increasing concern about unexpectedness of the onset</td><td rowspan="2">Inpatient hospitalization or prolongation of existing hospitalization
・Disability
・Death or likelihood of leading to disability
・Death or Serious Disease, as described above
・Congenital disease or abnormality in later generations</td><td>15 days</td></tr>
<tr><td>Expected</td><td>30 days</td></tr>
<tr><td>Unexpected</td><td>Infection-related disease or the like</td><td>15 days</td></tr>
<tr><td>Regardless of expectedness</td><td>Infection-related:
・Death
・Inpatient hospitalization or prolongation of existing hospitalization
・Disability
・Death or likelihood it will lead to disability
・Death or Serious Disease, as described above
・Congenital disease or abnormality in later</td><td>15 days</td></tr>
</table>

		generations	

* To be determined on the basis of the protocol or patient information sheet or the precautions described in the package insert, container, or wrapper of the pharmaceuticals used in the study.

2) SAE and AESI

If the Representative Investigator receives initial and follow-up reports on SAEs and AESIs, as a matter of principle the investigator should report them to the Pharmacovigilance Department, Nippon Boehringer Ingelheim Co., Ltd within 24 hours.

(2) Periodic reporting

To comply with Article 59 of regulations for enforcement of the Clinical Trials Act, the Representative Investigator should perform periodic reporting on the status of the clinical study and the occurrence of a disease or the like. Periodic reporting should be done within 2 months of the relevant surveillance period, which as a matter of principle is at the end of every year after the date when the protocol was submitted to the Minister of Health, Labour and Welfare.

In addition, under Article 60 of the regulations for enforcement of the Clinical Trials Act, periodic reporting to the Minister of Health, Labour and Welfare should be done regarding the matters set forth in Article 59, paragraph 1, item (i) of such regulations, within 1 month after the date when the Certified Review Board gives its opinion.

(3) Type of report form

1) Expedited report

[1] Serious Diseases

Form for reporting to the Certified Review Board: The report on a disease or the like associated with pharmaceuticals “Unified Form 8,” as specified in the “Regarding Unified Forms for the Clinical Trials Act” (Administrative communication of the Research and Development Division, Health Policy Bureau, Ministry of Health, Labour and Welfare [MHLW] (April 9, 2018)).

[2] SAEs/AESIs

Form for reporting to the Pharmacovigilance Department, Nippon Boehringer Ingelheim Co., Ltd: “Unified Form 8” and the study-specific attachment.

2) Periodic report

[1] Form for reporting to the Certified Review Board: Periodic disease report “Unified Form 6,”as specified in the “Regarding Unified Forms for the Clinical Trials Act” (Administrative communication of the Research and Development Division, Health Policy Bureau, MHLW (April 9, 2018)).

[2] Form for reporting to the Minister of Health, Labour and Welfare: Periodic report “Attachment Form 3,” as specified in “Regarding regulations for enforcement of the Clinical Trials Act (Notification No. 1 0228 issued by the Director of Office of Economic Affairs Division, Research and Development Division, Health Policy Bureau, Ministry of Health, Labour and Welfare,

February 20, 2018).”

(4) Procedures for reporting AEs (Figures 2 and 3)

1) Reporting events that require expedited reporting

A. Procedures for reports from research institutions

[1] If a Principal Investigator or Subinvestigator notices the onset of any event that requires expedited reporting, the investigator should fill out the predetermined information in the report form and send it to the research office by fax or e-mail. For Serious Diseases, the report should be sent to the research institution administrator. For events other than Serious Diseases that require expedited reporting, the report should be sent to the research institution administrator, if necessary, as specified by each research institution. Regarding the causal relationship, the relationship between the relevant event and the study should be evaluated as “Related” or “Not related.” If the relationship between an event and the participant’s participation in the study is classified as Unknown, in the report the event will be regarded as being related to the study.

[2] If the research office receives an expedited report, it should immediately submit a written report to the Representative Investigator and the Data and Safety Monitoring Board. However, AESI should be reported to the Data and Safety Monitoring Board as necessary at the discretion of the Representative Investigator

[3] The Representative Investigator should report Serious Diseases to the Certified Review Board by the reporting deadline.

[4] In addition, the Representative Investigator should inform the Principal Investigators at each research institution, and those Principal Investigators should promptly report the relevant information to the research institution administrator. If the Representative Investigator receives initial and follow-up reports on any event that requires expedited reporting, as a matter of principle the investigator should submit a written report to the Pharmacovigilance Department, Nippon Boehringer Ingelheim Co., Ltd within 24 hours.

[5] The Data and Safety Monitoring Board should review the contents of the reporting event that requires expedited reporting and should recommend future actions, including those related to a potential need for code breaking, handling of cases, and continuation/discontinuation of the research, to the Representative Investigator in writing; the procedures of the Data and Safety Monitoring Board will be described separately.

[6] After receiving the recommendation from the Data and Safety Monitoring Board, the Representative Investigator should communicate the information for such expedited reporting as well as the decision on the continuation, modification, or discontinuation of the study to all research institutions. The Representative Investigator should inform the research institutions of AESIs if he deems it necessary.

B. Complementary collection by the assistant research office of events that require expedited reporting

[1] The assistant research office should regularly check the information entered by research institutions into EDC (apart from on Saturdays, Sundays, or holidays). If they find any event that

falls into the category of events that require expedited reporting, as defined in paragraph 2 of the procedures, the assistant research office will promptly ask the relevant research institution to collect the required information by using the report form and to submit the necessary documents.

[2] The assistant research office should promptly report the information for expedited reporting complementarily collected as described in [1] above to the Representative Investigator, and the Data and Safety Monitoring Board as necessary, using each report form. However, AESI should be reported to the Data and Safety Monitoring Board as necessary at the discretion of the Representative Investigator.

(Figure 2)

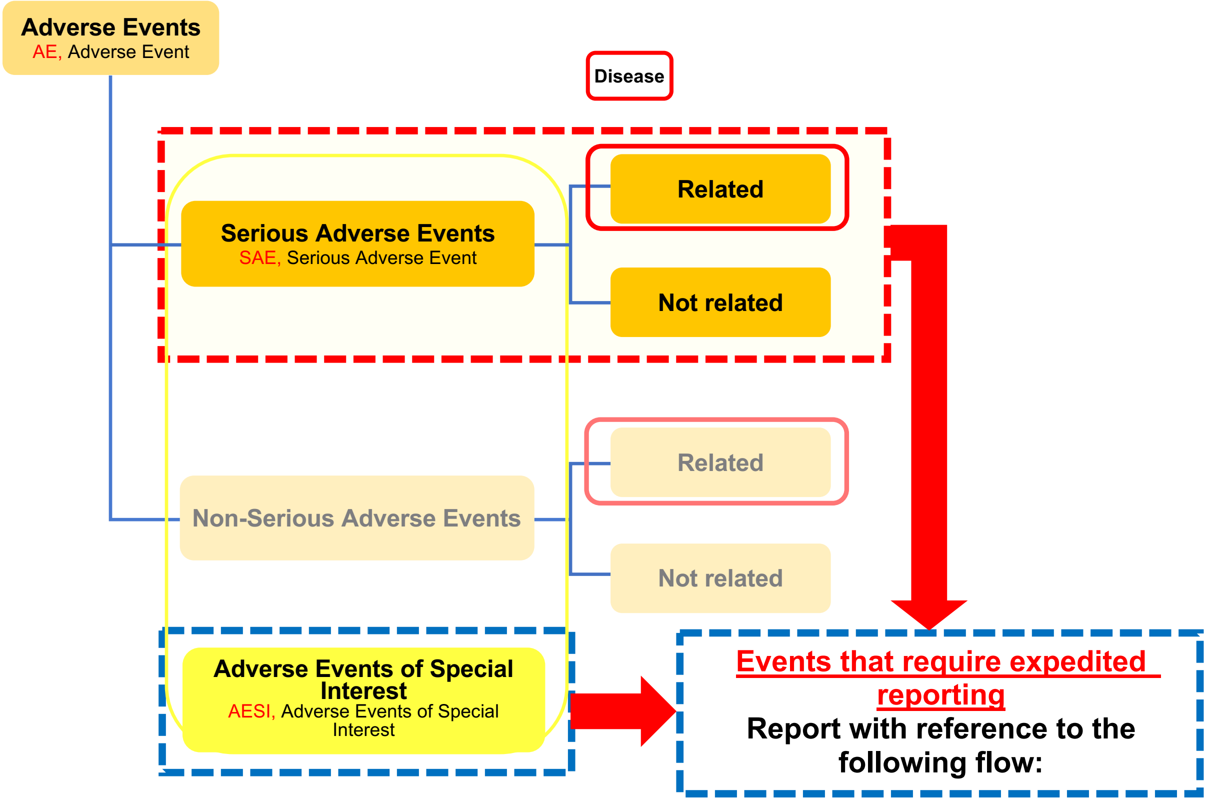

* All AE information should be entered into the case report form.

(Figure 3)

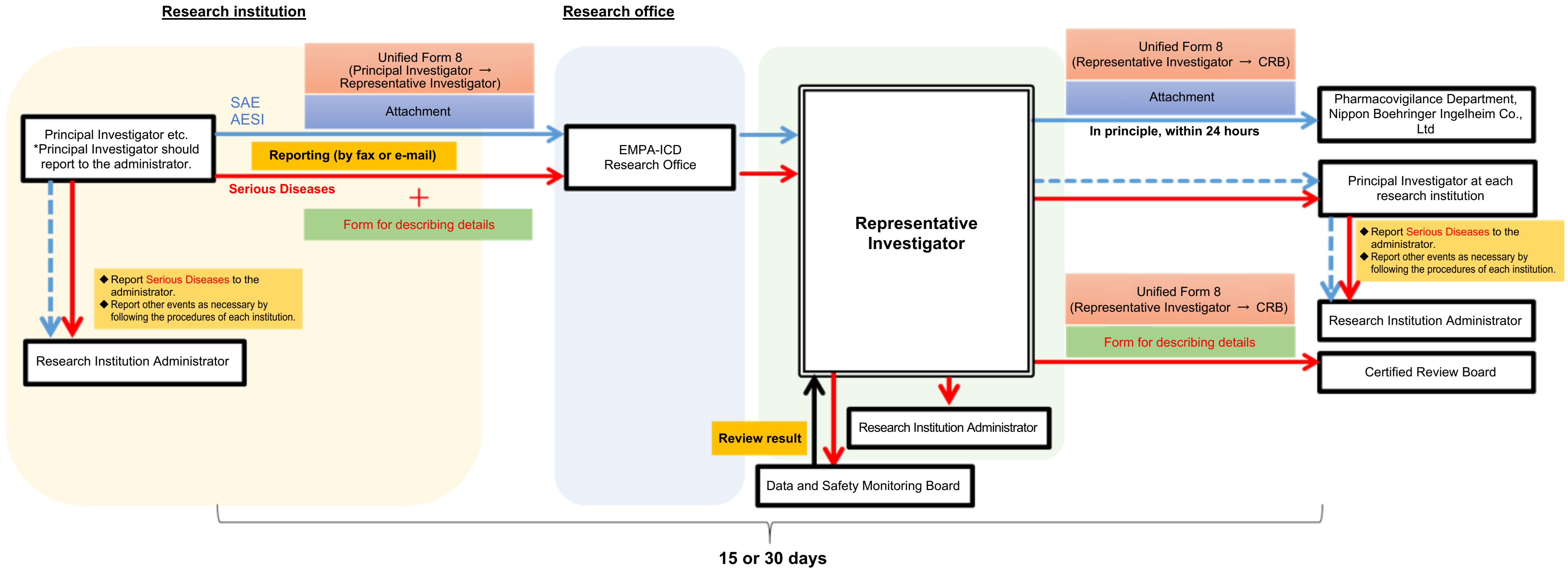
Research institution
Research office
Unified Form 8 (Principal Investigator → Representative Investigator)
Attachment
SAE
AESI
Principal Investigator etc. *Principal Investigator should report to the administrator.
Reporting (by fax or e-mail)
Serious Diseases
+
Form for describing details
◆ Report Serious Diseases to the administrator.
◆ Report other events as necessary by following the procedures of each institution.
Research Institution Administrator
EMPA-ICD Research Office
Representative Investigator
Unified Form 8 (Representative Investigator → CRB)
Attachment
In principle, within 24 hours
Pharmacovigilance Department, Nippon Boehringer Ingelheim Co., Ltd
Principal Investigator at each research institution
◆ Report Serious Diseases to the administrator.
◆ Report other events as necessary by following the procedures of each institution.
Unified Form 8 (Representative Investigator → CRB)
Form for describing details
Research Institution Administrator
Certified Review Board
Review result
Research Institution Administrator
Data and Safety Monitoring Board
15 or 30 days

6. Where to report events that require expedited reporting

(1) Events that require expedited reporting should be sent by fax or e-mail, as follows:
Fax: 03-6262-2815 (EMPA-ICD Assistant Research Office)
E-mail: empa-icd@micron-kobe.com (e-mail can be used if a fax machine is not available.)

7. Breaking emergency key code

If code breaking is required for reported AEs, follow “Procedures for Management of Breaking Emergency Key Code.”

8. Storage of materials

Store materials related to AEs for the storage period described in the protocol.

9. History of changes

Version	Description of change
1.0	Initial preparation
1.1	Modification related to enforcement of the Clinical Trials Act
1.2	Additional description of required action in case of necrotizing fasciitis of the perineum (Fournier's gangrene)

Department of Cardiovascular Medicine, Niigata University Graduate School of Medical and Dental Sciences

SOP in case of protocol violations

Comparison of empagliflozin or placebo for prevention of lethal ventricular arrhythmia in type 2 diabetic patients with implantable cardioverter defibrillators (ICD)

Representative Investigator: Tohru Minamino, Department of Cardiovascular Medicine, Niigata University Graduate School of Medical and Dental Sciences

Ver.1.1

8/31/2018

Contents

1. Purpose and scope of the document ... 3
2. Definition of protocol violation in the study ... 3
3. Definition and role of each person involved in the study ... 3
4. Reportable protocol violations ... 4
Reportable protocol violations ... 4
Timing of protocol violation reporting ... 4
5. Procedures for reporting protocol violations ... 4
6. Where to report protocol violations ... 7
7. Storage of materials ... 7
8. History of changes ... 8

1. Purpose and scope of the document

This document defines protocol violation and describes the role and workflow of people involved in the study to help standardize procedures for evaluating and reporting protocol violations that occur during the conduct of the study.

2. Definition of protocol violation in the study

Protocol violation

"Protocol violation" refers to incompliance with regulations, study protocol, and SOP and to tampering with and manipulating study data.

Serious protocol violation

"Serious protocol violation" refers to occurrences that influence the human rights and safety of patients, the progress of the study, and the reliability of the study results. For example, cases of serious protocol violation include incompliance with inclusion and exclusion criteria or study discontinuation criteria and combined use of treatments where combined use is prohibited; however, serious protocol violation does not include cases of deviation from the study protocol for medically necessary reasons to avoid an emergent risk in patients.

3. Definition and role of each person involved in the study

(1) Representative Investigator
Tohru Minamino, Department of Cardiovascular Medicine, Niigata University Graduate School of Medical and Dental Sciences
The Representative Investigator of the study should consider the wellbeing of participants; ensure that Principal Investigators, Subinvestigators, and other people involved in the study comply with regulations and the protocol; report and recognize protocol violations; and share information in a timely manner with people involved in the study. In addition, regarding all reported protocol violations associated with the study, the Representative Investigator is responsible for maintaining and storing detailed records, including electronic case report forms (electronic data capture, hereafter called "EDC"). If, on the basis of the information obtained during the study, the protocol violation is deemed to be a serious protocol violation after receiving the required opinion from the Data and Safety Monitoring Board or if it is reported as a serious protocol violation by a Principal Investigator, the Representative Investigator should promptly request the opinion of the Certified Review Board. The Representative Investigator should also take measures to prevent recurrence of the protocol violation; communicate such measures to Principal Investigators, Subinvestigators, and other people involved in the study; and ensure that recurrence is prevented. After obtaining the opinion from the Certified Review Board, any additional information that is required should be promptly communicated.

(2) Research institution administrator:
The administrator will receive reports on all protocol violations occurring at a research institution that is enrolling patients in the study.

(3) Principal Investigator:
The physician who supervises study operations at participating research institutions. If the

Principal Investigator notices an protocol violation, he/she should promptly report it to the research institution administrator and notify the Representative Investigator accordingly. In addition, the Principal Investigator is responsible for preparing and appropriately reporting, maintaining, and storing detailed records, including EDC, etc., regarding all protocol violations occurring at the research institution.

(4) Subinvestigator:
The physician who performs study operations at the research institutions. If the Subinvestigator notices a protocol violation, he/she should promptly report it to the Principal Investigator.

(5) Research office:
The office organized by the Representative Investigator. It is responsible for the operations for research administrative procedures in general, including those related to protocol violation reporting at the Department of Cardiovascular Medicine, Niigata University Graduate School of Medical and Dental Sciences.

(6) Datacenter:
Center for Clinical Research and Innovation (CCRI), Osaka City University Hospital
The division in charge of data management for the study. It will collect information on protocol violations from data entered into the study EDC.

(7) Data and Safety Monitoring Board:
The board will monitor and evaluate the progress of the study and safety data, etc., and, as a third party, assess the severity of the protocol violation or the measures to prevent recurrence and inform the Representative Investigator of its opinion.

4. Reportable protocol violations

Reportable protocol violations

All protocol violations that occur during the study between informed consent and Week 24 (or the time of discontinuation) are reportable.

Timing of protocol violation reporting

Principal Investigators and Subinvestigators (hereafter called “Principal Investigators, etc.”) should promptly report any protocol violation when they notice one, including not only protocol violations discovered by their own inspections but also those discovered by monitoring and auditing.

5. Procedures for reporting protocol violations

Regarding recognized protocol violations associated with the study, the Principal Investigators, etc., should report detailed records by creating them in EDC, etc., and should maintain and store them. Reporting procedures for protocol violations specified in the Clinical Trials Act and for those specified in the study protocol are shown below.

(1) Routine report (Figure 1)
1) If Principal Investigators, etc., notice any protocol violation at the research institution, they

should promptly report it to the Representative Investigator and the research institution administrator.

■ **Principal Investigators** should report any protocol violation they notice to the research institution administrator and should notify the Representative Investigator of it by using EDC.
■ **Subinvestigators** should promptly report any protocol violation they notice to the Principal Investigator; however, if they are concerned that reporting it to the Principal Investigator might preclude appropriate reporting, they should report it directly to the Representative Investigator and the head of the research institution. EDC should be used for reporting the protocol violation to the Representative Investigator.

2) The Representative Investigator should make the final decision about the degree of protocol violation (serious or nonserious) after obtaining the required opinion from the Data and Safety Monitoring Board.

3) The Representative Investigator should promptly report the protocol violation to the Certified Review Board by using the designated form, especially if, in the opinion of the Data and Safety Monitoring Board, the protocol violation is serious. If a protocol violation is not deemed serious, a periodic report should be sent to the Certified Review Board by using the designated form.

4) After obtaining the opinion of the Certified Review Board, the Representative Investigator should summarize the serious protocol violation in a predetermined form and develop measures to prevent its recurrence.

5) The Representative Investigator should send the summary of the serious protocol violation and the measures to prevent its recurrence to all physicians involved in the study.

(Figure 1)

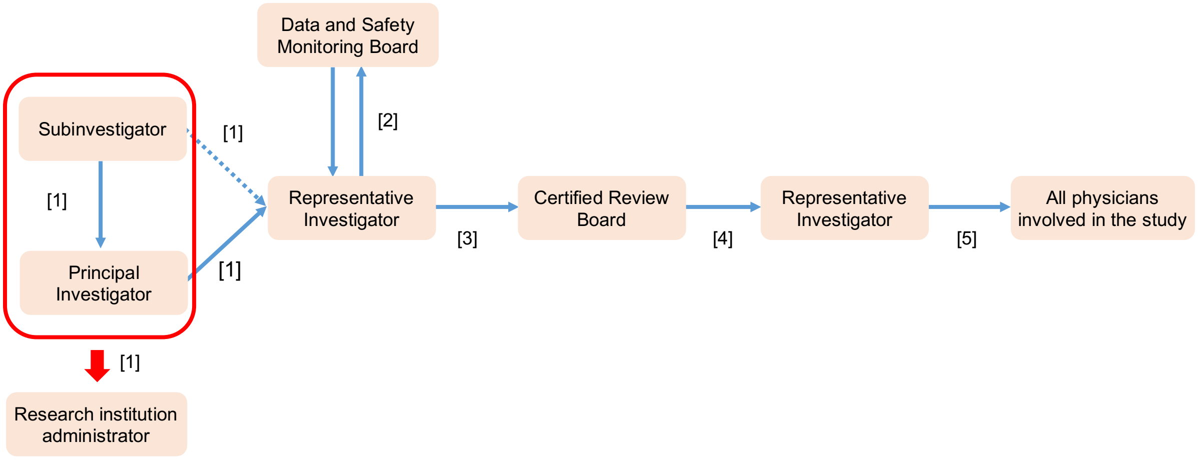

(2) Periodic report

According to Article 59, Enforcement Regulations of Clinical Trials Act, the Representative

Investigator should periodically report the occurrence of and response to a study-related protocol violation in accordance with the Enforcement Regulations or the protocol. Periodic reporting should be done within 2 months of the relevant surveillance period, which as a matter of principle is at the end of every year after the date when the protocol was submitted to the Minister of Health, Labour and Welfare.

In addition, in accordance with Article 60, Enforcement Regulations, Clinical Trials Act, if any protocol violation is reported to the MHLW, the MHLW should be asked for confirmation of receipt every time.

Types of report form

1) Routine report

Serious protocol violation

Use the report on pharmaceuticals and diseases or the like “Unified Form 7” to report a serious protocol violation to the Certified Review Board.

* Specified in “Regarding Unified Forms for the Clinical Trials Act” (administrative communication of the Research and Development Division, Health Policy Bureau, MHLW, April 9, 2018).

Summary of and measures to prevent recurrence of protocol violation

Using attachments specified for the study, communicate the protocol violation to Principal Investigators, Subinvestigators, and other people involved in the clinical study and ensure that recurrence is prevented.

2) Periodic report

Form for reporting to the Certified Review Board: Periodic disease report “Unified Form 5”

* Specified in “Regarding Unified Forms for the Clinical Trials Act” (administrative communication of the Research and Development Division, Health Policy Bureau, MHLW, April 9, 2018).

6. Where to report protocol violations

Principal Investigators and Subinvestigators should report protocol violations by using EDC. If any problem occurs during reporting, contact the research office.

Contact for Public Queries	Name of the person in charge	Shinya Fujiki
	Affiliation of the person in charge	Niigata University Graduate School of Medical and Dental Sciences
	Department of the person in charge	Department of Cardiovascular Medicine
	Postal code of the institution of the person in charge	951-8510
	Address of the institution of the person in charge	1-757 Asahimachidori, Chuo-ku,<br>Niigata 951-8510, Japan
	Phone no.	025-227-2185
	Fax no.	025-227-0774
	E-mail address	shinya_fukuji@yahoo.co.jp

7. Storage of materials

Store materials related to protocol violations for the period specified in the protocol.

8. History of changes

Version	Description of change
1.0	Initial preparation
1.1	Modification related to enforcement of the Clinical Trials Act

Comparison of empagliflozin or placebo for prevention of lethal ventricular arrhythmia in type 2 diabetic patients with implantable cardioverter defibrillators (ICD)

Monitoring procedures

Representative Investigator
Tohru Minamino, Professor, Department of Cardiovascular Medicine,
Niigata University Graduate School of Medical and Dental Sciences
Address: 1-757 Asahimachidori, Chuo-ku, Niigata 951-8510, Japan
Tel no.: 025-227-2185
Fax no.: 025-227-0774

Version 1.1 prepared on August 31, 2018

1 Purpose and scope of the document

This document describes the procedures and other matters that are necessary so that monitors can appropriately conduct monitoring of the “Comparison of empagliflozin or placebo for prevention of lethal ventricular arrhythmia in type 2 diabetic patients with implantable cardioverter defibrillators” (hereafter called the “study”) for the Representative Investigator.

2 Implementation system and responsibilities

2.1 Responsibilities of the Representative Investigator

(1) The Representative Investigator should arrange for monitors to conduct monitoring to ensure the proper conduct of the study from the standpoint of ensuring reliability of the study and protecting participants and to examine the progress of the study and whether the study is being conducted in accordance with the Clinical Trials Act and the protocol.

(2) The Representative Investigator should provide the necessary instruction and management for monitoring by monitors.

2.2 Monitor responsibilities

(1) Monitors should confirm their commitment to protecting human rights and ensuring the safety of participants and ensuring appropriate conduct of the study in accordance with the Clinical Trials Act, updated Trial Plan, and the protocol and should confirm that written consent to participate in the study has been obtained from all participants. In addition, they should confirm that records are accurate by using medical records and procedure documents as source documents for case report forms.

(2) If monitors find any matter or protocol violation that is likely to affect the appropriate conduct of the study or if they find any deviation from the protocol, they should promptly notify the Principal Investigator of the matter and take appropriate action to prevent its recurrence.

(3) Each time they conduct monitoring, monitors should prepare a monitoring report that summarizes important matters they discovered or facts about a disease or the like, protocol violation, etc., and submit it to the Principal Investigator. The monitoring report should include the following items:

[1] Date of monitoring
[2] Name of monitor
[3] Name of the Principal Investigator, Subinvestigator, and research assistant from whom monitors heard explanations during monitoring.
[4] Summary of monitoring results (including the summary and important matters discovered or facts about inspected items, deviations, and defects, and conclusions)
[5] Matters reported to the Representative Investigator as specified in “2. 2 (2).”
[6] The action to be taken on the matters specified in [5], and the monitor’s findings on such

action.

2.3 Monitor requirements

Monitors should have intimate knowledge of the Clinical Trials Act, Trial Plan, protocol, and informed consent form and procedures, should understand the ethical principles for research* and should have the scientific and clinical knowledge necessary for monitoring. They should also have received training in all of the following:

(1) General business manner
(2) Basic knowledge of natural science, including medicine, pharmacy, nursing, and laboratory medicine
(3) Clinical Trials Act
(4) The investigational product, Trial Plan, and protocol, informed consent form and other research-related materials, standard operating procedures, etc.

* Declaration of Helsinki - Ethical Principles for Medical Research Involving Human Subjects (World Medical Association)
Clinical Trials Act, laws and regulations for protection of personal information

3 Procedures for conducting monitoring

3.1 Appointment of monitors

The Representative Investigator should appoint study monitors (Form 1) after confirming that they meet "2.3 Monitor requirements" based on their resume, education history, etc. Monitors appointed by the Representative Investigator must not be involved in the study.

3.2 Advance preparation for monitoring

3.2.1 Identification of source documents

Wherever possible, at each research institution monitors should confirm the source documents with the Principal Investigator before informed consent is obtained.

3.3 Procedures for monitoring

The study should be monitored by on-site monitoring and remote monitoring.

3.3.1 Monitoring methods

3.3.1.1 On-site monitoring

On-site monitoring refers to the monitoring conducted by monitors when they visit research institutions.

3.3.1.2 Remote monitoring

Remote monitoring refers to the monitoring conducted by monitors without visiting the research

institutions; in this type of monitoring, monitors extract and use the data entered into electronic case report forms. In principle, remote monitoring includes confirming data with Principal Investigators or Subinvestigators and may include confirming data with other research assistants.

3.4 Monitoring items

3.4.1 On-site monitoring

1) First time

On-site monitoring should be conducted at the first 10 institutions that enroll participants. The monitors should use the source documents to confirm that informed consent was obtained from all patients who have been enrolled in the study at the time of monitoring. In addition, the eligibility of 1 to 3 random patients should be confirmed by reviewing the source documents. If there is any problem with confirming eligibility, on-site monitoring should be repeated at the respective research institution.

2) Second time

At the time of enrollment of all subjects, for 10 institutions on which the first on-site monitoring has not been conducted, confirmation of the status of consent from all subjects and the source documentation verification against electronic case report form data of 1 to 3 random subjects should be performed. The data entered in electronic case report form at the time of monitoring will be subjected to the source documentation verification against electronic case report form data. Any institutions in which no patients have been enrolled will not be subjected to monitoring. Instead, the second on-site monitoring should be conducted on the institutions on which the first on-site monitoring has been conducted in descending order of the number of enrolled subjects. In this case, the status of consent should be confirmed for the subjects with unconfirmed status.

3.4.2 Remote monitoring

1) Items to be monitored
   - The presence or absence of the description of concomitant medication
   - Status of the implementation of test and observation items (presence or absence of deviations)
   - Appropriateness of entered data

2) Timing
   - First time: After completion of the first on-site monitoring, remote monitoring should be conducted on the first 5 institutions which enrolled patients of the institutions on which on-site monitoring has not been conducted.
   - Second time: The monitoring should be conducted, at the time of enrollment of all subjects, on 5 institutions on which the first remote monitoring has not been conducted.

3.5 Description of on-site monitoring

3.5.1 Informed consent

- Confirm source documents, such as informed consent forms of patients who consented to participate in the study, and that voluntary written consent to participate in the study was obtained from participants. In addition, confirm that any study procedure was not initiated before informed consent.
- Confirm that informed consent was obtained by using the patient information sheet approved by the Certified Review Board.

3.5.2 Eligibility

Confirm eligibility by verifying source documents regarding the following items:

- Allocation factors
- Inclusion and exclusion criteria
- Use of contraindicated drugs/treatments

3.5.3 Participant safety

- Confirm the presence or absence of adverse events. If any adverse event occurs, confirm the name of the adverse event, date of onset, severity, treatment, causal relationship, and subsequent course and validate the continuation of the study. In addition, if no follow-up has been performed, ask that the status of the participant be checked.
- If any serious adverse event occurs, confirm that the procedures specified in the protocol have been performed. In addition, confirm consistency of the report on the serious adverse event.
- If any adverse event occurs that is possibly related to the study (disease or the like), confirm that the procedures specified in the protocol have been performed.
- Confirm whether or not any participant discontinued the study. If any participant discontinued the study, confirm the date of study discontinuation, reason for the discontinuation, and, in the case of the study discontinuation because of an adverse event, outcome and subsequent course. If the outcome and subsequent course have not been reviewed, ask that they be reviewed.

4 Monitoring report

Monitors should prepare a monitoring report within 15 business days after conducting monitoring and submit it to the Principal Investigator at the monitored site. If necessary, the Principal Investigator should notify the Representative Investigator of the content of the monitoring report. The Representative Investigator should inform other Principal Investigators of the content of the notification.

5 Quality assessment

If monitors discover any protocol violation as a result of monitoring, they should discuss the cause

of the protocol violation with the Principal Investigator and, if necessary, with the Representative Investigator and should take actions to prevent it reoccurring.

6 Confidentiality

Monitors must not divulge participants' secret, identity-related information and other personal information obtained during monitoring.

7 Storage of materials

Principal Investigators and the Representative Investigator should store the monitoring report and other records of monitoring operations that were submitted by monitors.

8 History of preparations and amendments

Version	Date of preparation and amendment	Reason for and description of preparation and amendment
1.0	November 27, 2017	Initial preparation
1.1	August 31, 2018	Maintenance of descriptions

Form 1

MM/DD/YYYY

Appointment of Monitors

Representative Investigator

Tohru Minamino, Part-time Lecturer
Department of Cardiovascular Medicine
Niigata University Graduate School of
Medical and Dental Sciences

I hereby confirm that the following persons have the scientific and clinical knowledge necessary for monitoring and have received the necessary training, and I appoint them as monitors for the study.

Comparison of empagliflozin or placebo for prevention of lethal ventricular arrhythmia in type 2 diabetic patients with implantable cardioverter defibrillators (ICD)

Affiliation	Name

Comparison of empagliflozin or placebo for prevention of lethal ventricular arrhythmia in type 2 diabetic patients with implantable cardioverter defibrillators (ICD) [EMPA-ICD]

Audit procedures

Representative Investigator: Tohru Minamino,
Professor, Department of Cardiovascular Medicine,
Niigata University Graduate School of Medical and Dental Sciences

1 Purpose and scope

This document describes the procedures and other necessary matters so that the Representative Investigator can arrange for responsible auditors and other auditors (hereafter called “auditors”) to appropriately perform an audit to examine whether the study is being conducted in accordance with the Clinical Trials Act and the protocol. Auditing should ensure the reliability of materials collected from the study from the perspective of ensuring reliability of the study and protecting participants in the “Comparison of empagliflozin or placebo for prevention of lethal ventricular arrhythmia in type 2 diabetic patients with implantable cardioverter defibrillators” (hereafter called the “study”).

The following investigators are included in this procedure:

Representative Investigator: Tohru Minamino, Part-time Lecturer, Department of Cardiovascular Medicine, Niigata University Graduate School of Medical and Dental Sciences

Principal Investigator: Responsible person at participating research institutions

2 Implementation system and responsibilities

2.1 Responsibilities of the Representative Investigator

(1) To ensure the quality of the study, the Representative Investigator should appoint auditors and arrange for them to perform an audit for the purpose of determining independently and separately from quality control operations, including usual monitoring, that the study is being conducted in accordance with the Declaration of Helsinki, the Act on Securing Quality, Efficacy and Safety of Products Including Pharmaceuticals and Medical Devices and the Clinical Trials Act (hereafter called Related Laws and Rules), and the protocol and procedures for the study; that the study is being appropriately conducted by confirming through source data verification, etc.; and that the reliability of records is fully ensured.

(2) The Representative Investigator should ensure that audits are performed according to this procedure and that the audit plan is based on this procedure.

2.2 Auditor responsibilities

As required, auditors should perform an on-site audit at research institutions and should confirm that the study is being appropriately conducted and the reliability of data is fully ensured through source document verification, etc.

2.3 Auditor requirements

(1) Continuously educated and trained in Related Laws and Rules and audit-related matters

(2) Familiar with Related Laws and Rules and the study procedures

(3) Able to evaluate audit results from comprehensive and overall aspects

(4) Able to recognize problems related to clinical research and appropriately decide on analysis of and action to solve problems

(5) Able to appropriately express opinions and provide proper advice and recommendations based on the review and evaluation of the audit

(6) Able to maintain a fair and nondiscriminatory attitude

2.4 Appointment of Auditors

The Representative Investigator should appoint auditors for the study (Form 1) after confirming that they meet "2.3. Auditor requirements" by reviewing their resume, education history, etc. Auditors appointed by the Representative Investigator must not be involved in the conduct of the study or monitoring at the research institution being audited.

3 Type of audit

The Representative Investigator should arrange for auditors to perform an audit for clinical research and its system.

4 Audit procedures

4.1 Preparation of the audit plan

Auditors should prepare the audit plan. The Representative Investigator should confirm the content of the audit plan prepared by the auditors.
Matters to be described in the audit plan are specified in Form 2.

4.2 Audit for clinical research

(1) Matters to be audited:

[1] Representative Investigator
[2] Principal investigators etc. (including subinvestigators and assistant investigators)
[3] Research office (institution registration, patient enrollment, data management, statistical analysis)
[4] Certified Review Board and its office
[5] Laboratory tests
[6] Storage of materials
[7] Management of study drugs
[8] Monitoring
[9] Other matters as required (contract research organization etc.)

(2) Timing and frequency of the audit

In principle, the Representative Investigator should arrange for auditors to perform an audit at the following time and frequency:
[1] Time specified in the audit plan
[2] Time and frequency considered most appropriate in discussions between the Representative Investigator and the auditors.

(3) Preparation for audit

Before performing an audit, auditors should obtain or access the research institution's standard operating procedures for conducting clinical studies to confirm the institution's procedures for conducting research and accepting audits. Auditors should coordinate in

advance the acceptance of the audit and date with the Principal Investigator, etc., and the division to be audited and then ask to perform the audit.

(4) Conduct of audit
Auditors should perform the audit according to the audit plan. The following materials should be audited:
[1] Various standard operating procedures
[2] Study materials
- Certified Review Board records
- Records of approval by the heads of research institutions for the conduct of clinical research
- Materials submitted in advance to the Certified Review Board by the Representative Investigator
- Records on study registration (including jRCT)
- Records on Conflicts of Interest
- Records on means for compensating participants in case of injury
- Records on obtaining informed consent (from all participants)
- Records on the procedures for providing safety information during the study
- Source documents including medical records (The status should be confirmed for about 10% of enrolled patients of institutions which were extracted.)
- Documents created as specified in the protocol and the Clinical Trials Act
- Materials on agreements with the Contract Research Organization (CRO) and Site Management Organization (SMO)
- Other necessary materials

(5) Review of audit results
Auditors should summarize the matters discovered and confirmed by the audit as audit records, evaluate such records and consider problems and necessary action, etc.

(6) Preparation and submission of an audit report
Auditors should prepare an audit report on the basis of the audit records, including matters discovered or confirmed by the audit, and then, after affixing their signature or name and seal, they should submit the audit report to the Principal Investigator. Matters to be included in the audit report are described in Form 3. The Principal Investigator should notify the Representative Investigator of the content of the report, if necessary. The Representative Investigator should inform other Principal Investigators of the content of the notification.

(7) Response to the audit report
If necessary, the Principal Investigator should prepare and submit to the auditors the response to the submitted audit report.
Auditors who receive the response to the audit report should confirm that appropriate measures were taken to remedy problems and that such problems have been remedied. After determining that problems have been remedied, auditors should prepare a response confirmation and submit it to the Principal Investigator to whom the audit report was submitted under (6) and the head of the research institution. Moreover, if auditors determine that the

response to the suggestions in the audit is insufficient, they should describe the response as such in the confirmation of response, submit the confirmation of response to the Principal Investigator to whom the audit report was submitted under (6) and the head of the research institution and submit a copy to the Representative Investigator.
Auditors may perform a follow-up audit to confirm that appropriate remedial measures have been taken.

(8) Issuance of audit certificate
After completion of all audits for the study, auditors should send the Representative Investigator an audit certificate that includes the response, response confirmation, and follow-up audit (if applicable), as mentioned under the preceding item.

4.3 Audit for clinical research system

The Representative Investigator should arrange for auditors to perform an audit for clinical research system according to "4.2 Audit for clinical research". However, departments to be audited, materials to be audited, the time and frequency of audit, etc. should be determined through discussions with auditors

5 Forms to be used in audit

The following forms are attached and should be used for study audits (the documents other than the attached forms may be used if they describe the same items):
(1) Appointment of Auditors (Form 1)
(2) Resume, training history (Reference form)
(3) Audit Plan (Form 2)
(4) Audit Report (Form 3)
(5) Response to Audit Report (Form 4)
(6) Response Confirmation (Form 5)
(7) Audit Certificate (Form 6)

6 Confidentiality

Auditors will assume the same obligation as the Principal Investigator, etc., to not divulge participants' secret, identity-related information and other personal information obtained during the audit.

7 Storage of materials

The Representative Investigator should store the audit report and other records of operations for the audit submitted by auditors (including the available audit evidence) for the period specified in the protocol.

8 History of preparations and amendments

Version	Date of preparation and amendment	Reason for and description of amendment
1.0	November 27, 2017	Initial preparation
1.1	August 31, 2018	Maintenance of descriptions

Form 1

MM/DD/YYYY

Appointment of Auditors

Representative Investigator
Tohru Minamino, Part-time Lecturer, Department of Cardiovascular Medicine, Niigata University Graduate School of Medical and Dental Sciences

I hereby confirm that the following persons have the knowledge necessary for performing an audit and have received the necessary education, and I appoint them as auditors for the study.

Comparison of empagliflozin or placebo for prevention of lethal ventricular arrhythmia in type 2 diabetic patients with implantable cardioverter defibrillators (ICD)

Role	Affiliation	Name
Responsible auditor		
Auditor		
Auditor		

Form 2

MM/DD/YYYY

Audit Plan
(Clinical study audit)

Dear Representative Investigator,
Tohru Minamino, Part-time Lecturer, Department of Cardiovascular Medicine,
Niigata University Graduate School of Medical and Dental Sciences

Responsible auditor

____

Clinical study title	Comparison of empagliflozin or placebo for prevention of lethal ventricular arrhythmia in type 2 diabetic patients with implantable cardioverter defibrillators (ICD)
Audit scope<br>(Items and materials to be audited)	
Audit duration and period	
Auditor	
Division to be audited	

Note: The responsible auditor should prepare the required number of original copies of this form and submit one of them to the Representative Investigator.

Form 3

MM/DD/YYYY

Audit Report
(Clinical study audit)

Dear Principal Investigator and head of the research institution,

AND

Representative Investigator,
Tohru Minamino, Part-time Lecturer, Department of Cardiovascular Medicine,
Niigata University Graduate School of Medical and Dental Sciences

Responsible auditor

_______________________ Seal

Clinical study title	Comparison of empagliflozin or placebo for prevention of lethal ventricular arrhythmia in type 2 diabetic patients with implantable cardioverter defibrillators (ICD)

Audited division	
Type of audit	
Audited scope, materials, and matters	
Audit date	
Reference document for audit	☐ Clinical Trials Act ☐ Protocol ☐ Procedures
Person who responds to the audit	
Auditor	
Audit results [Opinion and proposed improvement]	
Attached materials	
Remark	

Note: The responsible auditor should prepare the required number of original copies of this form and submit one each to the audited Principal Investigator, the head of the research institution, and the Representative Investigator.

Form 4

MM/DD/YYYY

Response to Audit Report

Dear Responsible auditor

Principal Investigator

____

Clinical study title	Comparison of empagliflozin or placebo for prevention of lethal ventricular arrhythmia in type 2 diabetic patients with implantable cardioverter defibrillators (ICD)

Suggested matters	
Response<br><br>[Description, responsible person, and timing of the action taken on suggested matters]	

Form 5

MM/DD/YYYY

Response Confirmation

Dear Principal Investigator and head of the research institution,

AND

Representative Investigator,
Tohru Minamino, Part-time Lecturer, Department of Cardiovascular Medicine,
Niigata University Graduate School of Medical and Dental Sciences

Responsible auditor

Clinical study title	Comparison of empagliflozin or placebo for prevention of lethal ventricular arrhythmia in type 2 diabetic patients with implantable cardioverter defibrillators (ICD)

Suggested matters	
Response<br><br>[Description, responsible person, and timing of the action taken on suggested matters]	
Confirmed matters	

Note: The responsible auditor should prepare the required number of original copies of this form and submit one each to the audited Principal Investigator, the head of the research institution, and the Representative Investigator.

Form 6

MM/DD/YYYY

Audit Certificate

Dear Representative Investigator,
Tohru Minamino, Part-time Lecturer, Department of Cardiovascular Medicine,
Niigata University Graduate School of Medical and Dental Sciences

Responsible auditor

______________________ Seal

I hereby certify that the audit has been performed for the following clinical study:

Clinical study title	Comparison of empagliflozin or placebo for prevention of lethal ventricular arrhythmia in type 2 diabetic patients with implantable cardioverter defibrillators (ICD)

Audited division	
Type of audit	
Audited scope, materials, and matters	
Audit date	
Auditor	
Date when the audit report was submitted and the person to whom it was submitted	

Note: The responsible auditor should prepare the required number of original copies of this form and submit one to the Representative Investigator.

Study management

1. Research organization

This study is an investigator-initiated study and will be conducted by a research organization consisting of the Representative Investigator; the study adviser; the person in charge of supporting research and development programs; the person who coordinates and manages research; the person who, in addition to the Representative Investigator and Principal Investigators, supervises research; a steering committee; a Data and Safety Monitoring Board; an event assessment committee; a statistical analysis body; a research office; a project management body; a data management body; a monitoring body; an audit body; central measurement bodies; the people responsible for tests; a study drug management body; a study drug allocation body; and the research institutions (Principal Investigators), etc.

(1) Principle Investigator

This investigator is responsible for planning, conducting, analyzing, and disseminating the results of the study and will supervise the overall study.

Tohru Minamino, Professor, Department of Cardiovascular Medicine, Niigata University Graduate School of Medical and Dental Sciences, Niigata, Japan

(2) Study adviser

This adviser will advise on the conduct and analysis of the study and the dissemination of its results.

Yoshifusa Aizawa, Department of Research and Development, Tachikawa General Hospital, Tachikawa Medical Center

(3) Person in charge of supporting research and development programs

This person will clarify the direction of the overall study and support the efficient planning and operation of a series of processes, including conception, strategy development, dissemination of results (including practical application), and optimization of the necessary multiple clinical studies and basic research, etc. By critically evaluating the protocol (or development strategy) in terms of clinical pharmacology (especially drug efficacy evaluation and research ethics), general clinical practice or the laws and regulations related to clinical research, this person will also support the basic framework of the most effective and efficient (optimized) protocol for the clinical development program.

Koichi Node, Professor, Cardiovascular Medicine, Saga Medical School Faculty of Medicine, Saga University

(4) Person who coordinates and manages research

This person will ensure smooth coordination and management of the study by applying knowledge and approaches for planned and efficient operational management of clinical research.

Shinya Fujiki, Department of Cardiovascular Medicine, Niigata University Graduate School of Medical and Dental Sciences

(5) Person who, in addition to the Representative Investigator and Principal Investigators, supervises research

The owner of patent rights on pharmaceuticals to be used in the study or the person who procures research funds for the study. This person will supervise the study.

Shinya Fujiki, Department of Cardiovascular Medicine, Niigata University Graduate School of Medical and Dental Sciences

(6) Steering committee

This committee will plan, conduct, analyze and announce the study, recognize problems during conduct of the study, discuss solutions and coordinate any actions required for study operations.

Chairperson	Toyoaki Murohara, Professor, Cardiovascular Medicine, Nagoya University
Members (in random order)	Toshihisa Anzai, Professor, Cardiovascular Medicine, Hokkaido University
	Kenji Ando, Medical Director, Cardiovascular Medicine, Kokura Kinen Hospital
	Junichi Nitta, Senior Director, Cardiovascular Medicine, Sakakibara Heart Institute
	Masaaki Okabe, Director of the hospital, Tachikawa General Hospital
	Ritsushi Kato, Professor, Department of Cardiology, Saitama Medical University International Medical Center
	Kazuomi Kario, Professor, Cardiovascular Medicine, Jichi Medical University
	Kengo Kusano, Director of the Cardiovascular Department, National Cerebral and Cardiovascular Center
	Yoshio Kobayashi, Professor, Cardiovascular Medicine, Chiba University Hospital
	Shingo Sasaki, Associate Professor, Cardiology and Nephrology, Hirosaki University
	Morio Shoda, Professor, Cardiovascular Medicine, Tokyo Women's Medical University
	Yukio Sekiguchi, Associate Professor, Cardiovascular Medicine, University of Tsukuba
	Kazuyoshi Takahashi, Director of the Department of Cardiovascular Medicine, Niigata City General Hospital
	Yasuchika Takeishi, Professor, Cardiovascular Medicine, Fukushima Medical University
	Kenichi Tsujita, Professor, Cardiovascular Medicine, Kumamoto University
	Hirofumi Tomita, Professor, Cardiology and Nephrology, Hirosaki University
	Shigeto Naito, Director, Cardiovascular Medicine, Gunma Prefectural Cardiovascular Center
	Shinichi Niwano, Professor, Cardiovascular Medicine, Kitasato University
	Nobuhisa Hagiwara, Professor, Cardiovascular Medicine, Tokyo Women's Medical University
	Tetsuji Miura, Professor, Department of Cardiovascular, Renal and Metabolic Medicine, Sapporo Medical University
	Takeshi Mitsuhashi, Associate Professor, Cardiovascular Medicine, Jichi Medical

University Saitama Medical Center

(7) Data and Safety Monitoring Board

This board evaluates safety-related data and will consider the need to amend the protocol and the appropriateness of continuing the study and make respective recommendations to the sponsor.

Chairperson	Naohiko Takahashi, Professor, Cardiovascular Medicine, Oita University
Members (in order of the Japanese syllabary)	Kojiro Ueki, Director of the Diabetes Research Center, Research Institute, National Center for Global Health and Medicine<br>Yohei Ohno, Lecturer, Cardiovascular Medicine, Tokai University<br>Koichiro Kuwahara, Professor, Cardiovascular Medicine, Shinshu University<br>Motoaki Sano, Associate Professor, Cardiovascular Medicine, Keio University

(8) Event assessment committee

This committee will evaluate the data related to each arrhythmia event (an endpoint), consider the appropriateness of continuing the study and make recommendations to the sponsor.

Chairperson	Hiroshi Furushima, Director of the Furushima Clinic
Members	Hirotaka Sugiura, Director, Cardiovascular Medicine, Niigata Medical Center<br>Shinsuke Okada, Director, Cardiovascular Medicine, Niigata Medical Center

(9) Statistical analysis body

The body, which is responsible for statistical activities, will be the statistical experts for the study and perform the statistical analysis of the study results.

Responsible person	Takahiro Tanaka, Specially Appointed Assistant Professor, Clinical Research Quality Control division, Clinical and Translational Research Center, Niigata University Medical & Dental Hospital

(10) Research office

The research office will perform all administrative operations required for supporting and conducting the study and will support participating research institutions and investigators to ensure that the study progresses smoothly.

Shinya Fujiki, Department of Cardiovascular Medicine, Niigata University Graduate School of Medical and Dental Sciences
Address: 757 Ichibancho, Asahimachi-dori, Chuo-ku, Niigata city, Niigata 951-8510, Japan
Tel: 025-227-2185; Fax: 025-227-0774

Assistant research office
Clinical Research Department, Micron, Inc.
Responsible person: Tomoka Kanahama

Address: Nihonbashi NishikawaBldg. 4F, 1-5-3, Nihonbashi, Chuo-ku, Tokyo, 103-0027, Japan
Tel: 03-6262-2812; Fax: 03-6262-2815

(11) Project management body

This body will support the research office in maintaining the desired quality of the study and ensure that the study progresses efficiently and effectively.

Kenichi Iijima, Specially Appointed Assistant Professor, Department of Cardiovascular Medicine, Niigata University Graduate School of Medical and Dental Sciences
Address: 757 Ichibancho, Asahimachi-dori, Chuo-ku, Niigata city, Niigata 951-8510, Japan
Tel: 025-227-2185; Fax: 025-227-0774

(12) Data management body

This body will determine the order of data cleaning and fixing for enrolled participants and perform data management operations.

Clinical Research Quality Control division, Clinical and Translational Research Center, Niigata University Medical & Dental Hospital
Responsible person: Takahiro Tanaka
Address: 754 Ichibancho, Asahimachi-dori, Chuo-ku, Niigata city, Niigata 951-8520, Japan
Tel: 025-223-6161

Data center
Center for Clinical Research and Innovation (CCRI), Osaka City University Hospital
Responsible person: Keiko Ota
Address: ABENO MEDIX 6F, 1-2-7 Asahi-machi, Abeno-ku, Osaka-City, Osaka 545-0051, Japan
Tel: 06-6645-3470

(13) Monitoring body

This body will perform monitoring operations to confirm appropriate conduct of the study and the reliability, etc., of the data.

Clinical Research Department, Micron, Inc
Responsible person: Hiroyuki Shichijo
Address: Nihonbashi NishikawaBldg. 4F, 1-5-3, Nihonbashi, Chuo-ku, Tokyo, 103-0027, Japan
Tel: 03-6262-2812; Fax: 03-6262-2815

(14) Audit body

This body will perform audit operations as required to ensure the reliability of the study results.

Micron, Inc.: Reliability Assurance
Responsible person: Akiko Shibuya
Address: Nihonbashi NishikawaBldg. 4F, 1-5-3, Nihonbashi, Chuo-ku, Tokyo, 103-0027, Japan
Tel: 03-6262-2812; Fax: 03-6262-2815

(15) Central measurement bodies

These bodies will receive samples from study sites, perform the measurements described below and prepare a report of the respective results.

Measurement of blood ketone body fraction, blood catecholamine concentration, erythropoietin, and reticulocytes:
SRL, Inc.
Address: 2-1-1 Nishishinjuku, Shinjuku-ku, Tokyo 163-0409, Japan
Tel: 03-6279-0900 (main no.)

Measurement of telomere length, G-tail length and P53, P21, and P16:
Cardiovascular Medicine, Niigata University Graduate School of Medical and Dental Sciences
Address: 757 Ichibancho, Asahimachi-dori, Chuo-ku, Niigata city, Niigata 951-8510, Japan
Tel: 025-227-2185; Fax: 025-227-0774

Metabolome analysis:
Institute for Advanced Biosciences, Keio University
Address: 246-2, Mizukami, Kakuganji, Tsuruoka, Yamagata 997-0052, Japan
Tel: 0235-29-0528; Fax: 0235-29-0574

(16) People responsible for tests

These people will define the procedures for various tests and ensure that the quality of the tests is maintained.

Holter monitoring:
Kenichi Iijima, Specially Appointed Assistant Professor, Department of Cardiovascular Medicine, Niigata University Graduate School of Medical and Dental Sciences
Address: 757 Ichibancho, Asahimachi-dori, Chuo-ku, Niigata city, Niigata 951-8510, Japan
Tel: 025-227-2185; Fax: 025-227-0774

Echocardiography:
Ayumi Nakabo, Department of Cardiovascular Medicine, Niigata University Graduate School of

Medical and Dental Sciences
Address: 757 Ichibancho, Asahimachi-dori, Chuo-ku, Niigata city, Niigata 951-8510, Japan
Tel: 025-227-2185; Fax: 025-227-0774

^{123}I-MIBG myocardial scintigraphy:
Yosuke Horii, Assistant Professor, Department of Radiology and Radiation Oncology, Niigata University Graduate School of Medical and Dental Sciences
Address: 757 Ichibancho, Asahimachi-dori, Chuo-ku, Niigata city, Niigata 951-8510, Japan
Tel: 025-227-2185; Fax: 025-227-0774

(17) Study drug management body
Implementation Management division, Clinical and Translational Research Center, Niigata University Medical & Dental Hospital
Responsible person: Seiichi Maruyama
Address: 1-754 Asahimachi-dori, Chuo-ku, Niigata, 951-8520, Japan
Tel: 025-223-6161

(18) Study drug allocation body
Implementation Management division, Clinical and Translational Research Center, Niigata University Medical & Dental Hospital
Responsible person: Seiichi Maruyama
Address: 1-754 Asahimachi-dori, Chuo-ku, Niigata, 951-8520, Japan
Tel: 025-223-6161

Person responsible for study drug allocation
Department of Medical Statistics, Osaka City University Graduate School of Medicine and Faculty of Medicine
Responsible person: Hisako Yoshida
Address: Asahi-machi 1-5-7, Abeno-ku, Osaka-City, Osaka 545-8586, Japan
Tel: 06-6645-3894

(19) Study contact person
EMPA-ICD Assistant research office
Clinical Research Department, Micron, Inc.
Tomoka Kanahama
Address: Nihonbashi NishikawaBldg. 4F, 1-5-3, Nihonbashi, Chuo-ku, Tokyo, 103-0027, Japan

Tel: 03-6262-2812; Fax: 03-6262-2815

(20) Research Funder*

Nippon Boehringer Ingelheim Co., Ltd

Address: ThinkPark Tower, 2-1-1 Osaki, Shinagawa-ku, Tokyo 141-6017, Japan

Tel: 03-6417-2958; Fax: 03-5435-2981

(*Some of the research expenses will be borne by Eli Lilly and Company [US].)

2. Research institutions expected to participate (in order of the Japanese syllabary)

Institution	Affiliation	Principal Investigator	Address	Tel no. (main)
Tachikawa General Hospital, Tachikawa Medical Center	Cardiovascular Medicine	Masaaki Okabe	Asahioka 1-24, Nagaoka-shi, Niigata, 940-8621	0258-33-3111
Kitasato University Hospital	Cardiovascular Medicine	Shinichi Niwano	1-15-1, Kitazato, Minami, Sagamihara, Kanagawa, 252-0375	042-778-8111
Kumamoto University Hospital	Cardiovascular Medicine	Kenichi Tsujita	1-1-1 Honjo, Chuo-ku, Kumamoto City, Kumamoto 860-8556	096-344-2111
Gunma Prefectural Cardiovascular Center	Cardiovascular Medicine	Shigeto Naito	3-12, Kameizumi-town, Maebashi-city, Gunma 371-0004	027-269-7455
Kokura Kinen Hospital	Cardiovascular Medicine	Kenji Ando	3-2-1 Asano Kokurakita-Ku, Kitakyushu, Fukuoka 802-8555	093-511-2000
National Cerebral and Cardiovascular Center	Cardiovascular Department	Kengo Kusano	5-7-1 Fujishirodai, Suita, Osaka, 565-8565	06-6833-5012
Saitama Medical University International Medical Center	Department of Cardiology	Ritsushi Kato	1397-1, Yamane, Hidaka-City, Saitama 350-1298	042-984-4111
Sakakibara Heart Institute	Cardiovascular Medicine	Junichi Nitta	3-16-1 Asahi-cho, Fuchu, Tokyo, 183-0003	042-314-3111
Sapporo Medical University Hospital	Department of Cardiovascular, Renal and Metabolic Medicine	Tetsuji Miura	16-291, Minami-ichijo-nishi, Chuo-ku, Sapporo 060-8543	011-611-2111

Jichi Medical University Saitama Medical Center	Cardiovascular Medicine	Takeshi Mitsuhashi	1-847 Amanuma-cho, Omiya-ku, Saitama 330-8503	048-647-2111
Jichi Medical University Hospital	Cardiovascular Medicine	Kazuomi Kario	3311-1 Yakushiji, Shimotsuke-shi, Tochigi 329-0498	0285-44-2111
Chiba University Hospital	Cardiovascular Medicine	Yoshio Kobayashi	1-8-1 Inohana, Chuo-ku, Chiba-shi, Chiba, 260-8677	043-222-7171
University of Tsukuba Hospital	Cardiovascular Medicine	Yukio Sekiguchi	2-1-1 Amakubo, Tsukuba, Ibaraki 305-8576	029-853-3900
Tokyo Women's Medical University Hospital	Cardiovascular Medicine	Nobuhisa Hagiwara	8-1. Kawada-cho, Shinjuku-ku, Tokyo, 162-8666	03-3353-8111
Nagoya University Hospital	Cardiovascular Medicine	Toyoaki Murohara	65 Tsurumai-cho, Showa-ku, Nagoya 466-8560	052-741-2111
Niigata City General Hospital	Cardiovascular Medicine	Kazuyoshi Takahashi	463-7 Shumoku, Chuo-ku, Niigata City, Niigata 950-1197	025-281-5151
Niigata University Graduate School of Medical and Dental Sciences	Cardiovascular Medicine	Tohru Minamino	757 Ichibancho, Asahimachi-dori, Chuo-ku, Niigata city, Niigata 951-8510	025-227-2185
Hirosaki University Hospital	Cardiology and Nephrology	Hirofumi Tomita	53 Honcho, Hirosaki, Aomori 036-8563	0172-33-5111
Fukushima Medical University Hospital	Cardiovascular Medicine	Yasuchika Takeishi	1 Hikarigaoka, Fukushima-city, Fukushima 960-1295	024-547-1111
Hokkaido University Hospital	Department of Cardiovascular Medicine	Toshihisa Anzai	5 Kita14jonishi, Kita-Ku, Sapporo, Hokkaido 060-8648	011-716-1161

Placebo-controlled, double-blind study in type 2 diabetes-complicated patients with arrhythmia device implanted to evaluate change in severe arrhythmia after empagliflozin intervention

Comparison of empagliflozin or placebo for prevention of lethal ventricular arrhythmia in type 2 diabetic patients with implantable cardioverter defibrillators

[EMPA-ICD]

Protocol

Ver. 2.4

Study duration planned: From publication date in jRCT till October 2023

Issued: Mar. 11, 2022 Ver. 2.4

Table of contents

1 : Overview 8
1-1. Study design 8
1-2. Objectives 8
1-3. Patients 8
1-3-1. Inclusion criteria 8
1-4. Treatment 10
1-5. Evaluation endpoints 10
1-5-1. Primary endpoint 10
1-5-2. Secondary endpoints 10
1-5-3. Exploratory evaluation endpoints 11
1-5-4. Safety evaluation items 12
1-6. Study schedule 13
1-7. Target sample size and study duration planned 13
2 : Study background 14
3 : Objective 15
4 : Summary of study drug 15
4-1. Active drug 15
4-2. Placebo 16
5 : Patients 16
5-1. Patients 16
5-2. Inclusion criteria 16
5-3. Exclusion criteria 17
5-4. Discontinuation criteria 18
6 : Informed consent 19
6-1. Procedure to obtain informed consent 19
6-2. In case where information likely to affect participation in the study was obtained 20
7 : Study methods 20
7-1. Study design 20
7-2. Study outline 20
7-3. Target sample size and study duration planned 21
7-4. Patient enrollment and allocation 21
7-5. Blinding 22
7-6. Treatment method 22
7-7. Actions taken for additional treatment 22

７-８. Provisions for handling of study drug (Procedure for management and delivery)........ 25
７-９. Combination treatment........ 25
８: Observation and test items........ 28
８-１. Schedule for observation and tests (Refer to Table 1)........ 28
８-２. Observation and test items........ 29
９:　Evaluation endpoints........ 33
９-１. Primary endpoint........ 33
９-２. Secondary endpoints........ 34
９-３. Exploratory evaluation endpoints........ 35
９-４. Safety evaluation items........ 36
１０: Measures to be taken for AE onset........ 36
１０-１. About adverse reaction (AE)........ 36
１０-２. Duration to collect information on adverse events........ 37
１０-３. Investigation items of adverse events........ 37
１０-４. Report of adverse events........ 39
１１: Data collection........ 40
１１-１. Completion of case report form (CRF) and report........ 40
１１-２. Data management........ 41
１２: Statistical analysis........ 41
１２-１. Definition of analysis populations........ 41
１２-１-１. Full analysis set (FAS）........ 41
１２-１-２. Per protocol set (PPS)........ 41
１２-１-３. Safety analysis set (SAS)........ 41
１２-２. Analysis of primary endpoint........ 42
１２-３. Analysis of secondary, exploratory, and safety endpoints........ 42
１２-４. Significance level........ 42
１２-５. Handling of missing data........ 42
１３: Target sample size and rationales for setting........ 42
１４: Ethics........ 44
１４-１. Ethical review........ 44
１４-２. Protection of personal information........ 44
１４-３. Compensation for health impairment........ 45
１４-４. Patient’s benefit and disadvantage........ 45
１４-５. Patient’s expenses........ 45
１５: Quality control and assurance........ 46

１５-１. Source data ... 46
１５-２. Handling of data and samples ... 46
１５-３. Record storage ... 47
１５-４. Monitoring and audit ... 47
１５-５. Inspection by CRB or regulatory authority ... 47
１５-６. Control of incompatibility ... 47
１５-６-１. About incompatibility and serious incompatibility ... 47
１５-６-２. Duration to collect incompatibility information ... 48
１５-６-３. Report of incompatibility ... 48
１５-７. Report to administrator of participating medical institution ... 49
１５-８. Regular report to CRB ... 50
１５-９. Regular report to MHLW ... 50
１５-１０. Management for implementation of double-blind study ... 50
１６: Study fund and conflict of interest (COI) ... 50
１６-１. Study fund ... 50
１６-２. Conflict of interest (COI) ... 51
１７: Publication of study results and attribution of right ... 52
１７-１. Registration of clinical study ... 52
１７-２. Publication and attribution of study results ... 52
１８: Revision of study protocol ... 53
１９: Completion, discontinuation, or suspension of study ... 53
１９-１. Completion of study ... 53
１９-２. Discontinuation or suspension of study ... 53
２０: About Certified Review Board (CRB) ... 54
２１: Study system ... 54
２２: References ... 54

Appendices

A. Package insert for empagliflozin
B. Patient information, consent form, consent withdrawal
C. Procedure for special blood tests
D. Procedure for Holter monitoring
E. Procedure for echocardiographic tests
F. Procedure of ^{123}I-MIBG myocardial scintigraphy
G. Procedure to report adverse events
H. SOP at occurrence of incompatibility

I. Procedure for monitoring

J. Procedure for audit

K. Study system

[Abbreviations and definition of terms]

Abbreviation	Spelled-out terms
ADL	activities of daily living
ALT	alanine aminotransferase
ALP	alkaline phosphatase
AST	aspartate aminotransferase
BMI	body mass index
BNP	brain natriuretic peptide
BUN	blood urea nitrogen
CABG	coronary artery bypass grafting
Cl	chloride
COI	conflict of interest
Cr	creatinine
CRC	clinical research coordinator
CRT-D	cardiac resynchronization therapy-defibrillator
DNA	deoxyribonucleic acid
DPP-4	dipeptidyl peptidase-4
e’	early diastolic mitral annular velocity by tissue doppler echocardiography
EDC	electronic data capture
eGFR	estimated glomerular filtration rate
FAS	full analysis set
FDA	Food and drug administration
GLP-1	Glucagon-like peptide-1
HDL-C	high density lipoprotein cholesterol
HbA1c	hemoglobin A1c
HR	heart rate
ICD	implantable cardioverter defibrillator
IRR	Incident rate ratio
jRCT	Japan Registry of Clinical Trials
K	Potassium
LDH	lactate dehydrogenase
LDL-C	low density lipoprotein cholesterol
LVEF	left ventricular ejection fraction

Abbreviation	Spelled-out terms
^{123}I-MIBG	^{123}I-Meta-iodobenzylguanidine
Na	natrium
NSVT	non-sustained ventricular tachycardia
NYHA	New York Heart Association
OGTT	oral glucose tolerance test
PCI	percutaneous coronary intervention
PPS	per protocol set
RNA	ribonucleic acid
SAS	safety analysis set
SGLT2	sodium-dependent glucose transporter 2
SU	sulfonylurea
T-Bil	total bilirubin
TC	total cholesterol
TG	triglyceride
peak TRV	Peak Tricuspid regurgitation velocity
UA	uric acid
VF	ventricular fibrillation
VPC	ventricular premature complex
VT	ventricular tachycardia

1 : Overview

1 - 1 . Study design

Investigator-initiated multicenter, prospective, placebo-controlled, randomized, double-blind, parallel group comparison study

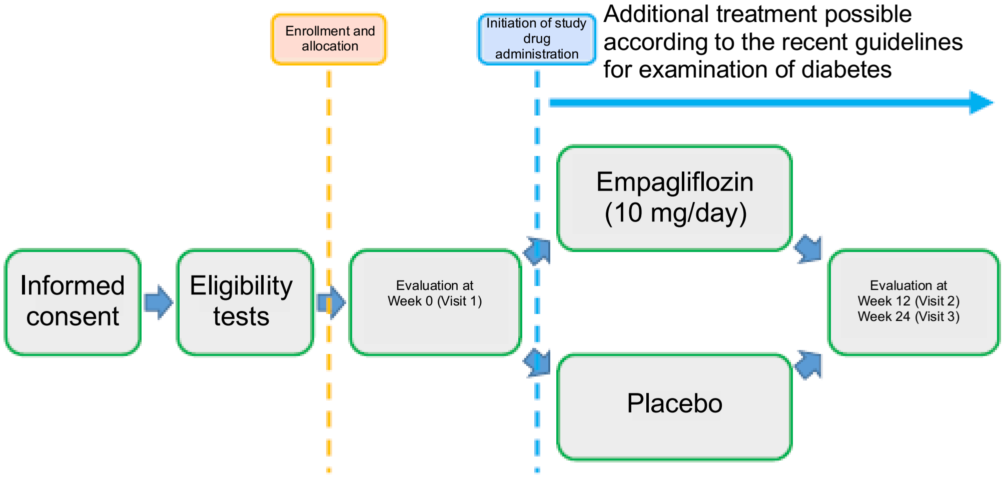

1 - 2 . Objectives

To evaluate whether empagliflozin decreases the number of events attributable to severe arrhythmia in type 2 diabetes-complicated patients with arrhythmia device implanted.

1 - 3 . Patients

Type 2 diabetes-complicated patients with arrhythmia treatment device (ICD/CRT-D) implanted

1 - 3 - 1 . Inclusion criteria

1) Patients aged 20 years or more when written consent was obtained (regardless of gender).
2) Patients who received ICD or CRT-D implantation surgery more than 24 weeks before the eligibility tests (regardless of implantation purpose: primary or secondary prevention).
3) Patients diagnosed with type 2 diabetes whom Investigator or Subinvestigator judged as possible to be administered empagliflozin (regardless whether drugs other than SGLT2 inhibitor are used to treat diabetes).
4) Patients who themselves granted consent to participation in the study in writing.

1 - 3 - 2 . Exclusion criteria

1) Patients using SGLT2 inhibitor at the time of eligibility tests.
2) Patients who were administered SGLT2 inhibitor within 24 weeks before the eligibility tests.
3) Patients who have past history of hypersensitivity to empagliflozin (Jardiance® Tablets).
4) Patients who have past history of diabetic ketoacidosis, diabetic coma, or hypoglycemic attack (hypoglycemia or protracted hypoglycemia requiring intervention of a third person) within 24 weeks before the eligibility tests.
5) Patients severely infected, before surgery, or seriously injured at the time of eligibility tests (excluding surgical operation to replace battery cell).
6) Patients receiving examination for type 1 diabetes.
7) Patients with cardiac failure of NYHA Class Ⅳ.
8) Patients with severe renal impairment (eGFR less than 30 mL/min/1.73 m^2 within 3 months after the consent was obtained or receiving renal dialysis).
9) Patients with serious hepatic function disorder (AST or ALT more than 3 times as high as the institutional standards within 3 months after the consent was obtained).
10) Patients with pituitary or adrenal insufficiency.
11) Patients who are under malnutritional or fasting condition, irregularly taking foods, with poor dietary intake or debilitated.
12) Patients with history of excessive alcohol intake.
13) Patients who have gastrointestinal disorder like diarrhea or vomiting at the time of eligibility tests and are likely to be dehydrated.
14) Patients with urinary tract or genital infection at the time of eligibility tests.
15) Patients who are pregnant, likely to be pregnant, or breastfeeding at the time of eligibility tests or want to be pregnant while participating in this study.
16) Patients whose BMI is less than 18.5 kg/m^2 at the time of eligibility tests.
17) Patients in whom the following events were observed to be likely to affect onset of severe arrhythmia within 24 weeks before the eligibility tests: alteration of antiarrhythmic drug, catheter ablation for ventricular arrhythmia, coronary revascularization, open-heart surgery, development of coronary artery disease, stroke or transient ischemic stroke seizure, infection requiring hospitalization, and cardiac failure requiring hospitalization.
18) Patients who are using an arrhythmia device unable to record nonsustained ventricular tachycardia (NSVT).
19) Patients who are complicated by non-remitted malignant tumor at the time of eligibility tests.

20) Patients whom Investigator or Subinvestigator judges as inappropriate to participate in this clinical study.

*See Sections 5-2 and 5-3 for rationales for setting the inclusion and exclusion criteria.

1-4. Treatment

Patients receive either of the following treatments after the eligibility tests, according to case enrollment No. assigned on the Web site for enrollment.

1) Study drug

   Empagliflozin 10 mg or placebo

2) Dosage and administration

   To orally administer once daily before or after breakfast for 24 weeks

1-5. Evaluation endpoints

1-5-1. Primary endpoint

The number of severe arrhythmia events (NSVT/VT/VF) recorded in the arrhythmia device is evaluated at Week 0 and 24 (or study discontinuation), to calculate the following: Difference in No. of severe arrhythmia events between the empagliflozin and placebo groups.

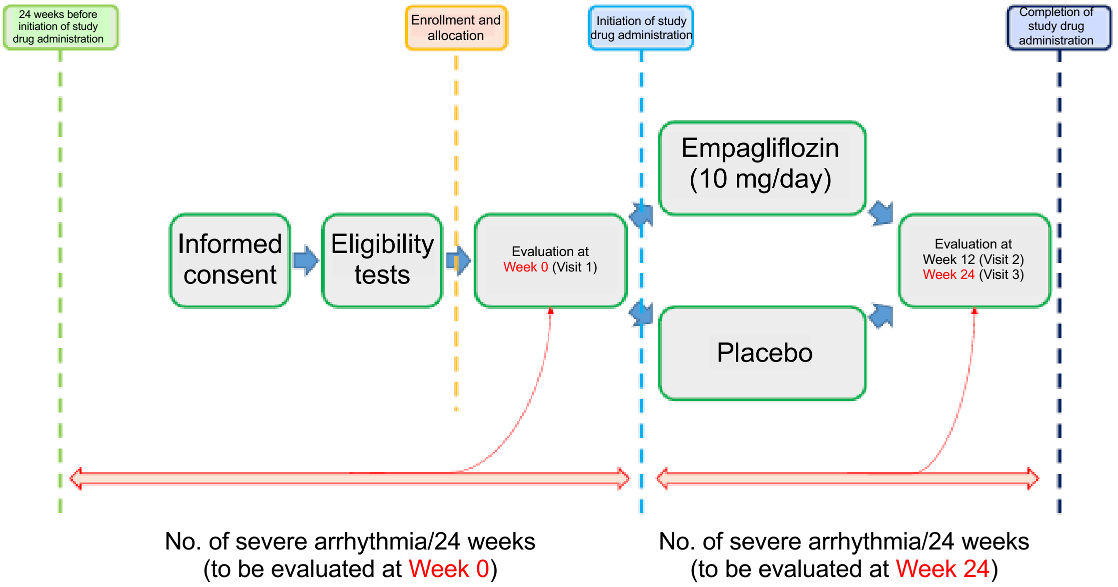

*The retrospective 24-week records are evaluated at Week 0 or 24, as shown above in Figure:

1-5-2. Secondary endpoints

1) The number of severe arrhythmia events (NSVT/VT/VF) and appropriate device operations (anti-tachycardia pacing/shock operation) recorded in the arrhythmia

device is evaluated at Week 0 and 24 (or study discontinuation), to calculate the following.

(1-1) Change in the number of severe arrhythmia events

(1-2) Incidence of severe arrhythmia events before and after treatment

(1-3) No. of severe arrhythmia events at Week 24

(1-4) Presence or absence of severe arrhythmia event at Week 24

(1-5) Change rate of No. of appropriate device operations

(1-6) Change in No. of appropriate device operation

(1-7) Incidence of appropriate device operation before and after treatment

(1-8) No. of appropriate device operations at Week 24

(1-9) Presence or absence of appropriate device operation at Week 24

These variables are compared between the empagliflozin and placebo groups.

In addition, the following are evaluated at Week 0 and 24 (or study discontinuation), to compare the change rate, change, and values at Week 24 between the empagliflozin and placebo groups.

2) Holter electrocardiographic tests: Total recording time, total No. of VPC, No. of single VPC, No. of two-consecutive VPC, and No. of ventricular tachycardia.

3) Blood ketone body fraction (acetoacetic acid, 3-hydroxybutyric acid, and total ketone bodies): blood concentration of each ketone body.

4) Blood catecholamine fraction (adrenalin, noradrenalin, and dopamine): blood concentration of catecholamine.

Correlation between difference in No. of severe arrhythmia events and blood ketone body or catecholamine is comparatively evaluated between the empagliflozin and placebo groups.

1-5-3. Exploratory evaluation endpoints

1) No. of atrial fibrillation events recorded in the arrhythmia device and biological monitoring indices are evaluated at Week 0 and 24, to calculate the following.

(1-1) Change rate of No. of atrial fibrillation event

(1-2) Change in No. of atrial fibrillation events

(1-3) Incidence of atrial fibrillation event before and after treatment

(1-4) No. of atrial fibrillation events at Week 24

(1-5) Presence or absence of atrial fibrillation event at Week 24

(1-6) Change rate of biological monitoring index

(1-7) Change in biological monitoring index

(1-8) Biological monitoring index at Week 24

These are compared between the empagliflozin and placebo groups.

In addition, the following are evaluated at Week 0 and 24 (or study discontinuation), to compare the change rate, change, and value at Week 24 between the empagliflozin and placebo groups.

2) NYHA classification of cardiac function
3) Body weight
4) Blood pressure, pulse rate, and body temperature
5) Blood glucose: HbA1c and fasting blood glucose
6) Serum lipid, serum uric acid, and cardiac failure marker: TC, HDL-C, LDL-C, TG, UA, and BNP
7) Renal function: Serum Cr, eGFR (estimated), BUN, Na, K, and Cl
8) Hepatic function: Total protein, albumin, AST, ALT, ALP, T-Bil, and LDH
9) Hematological value: RBC count, WBC count, Hb, Ht, and Plt
10) Erythropoietin
11) Reticulocytes
12) DNA tests: Telomere length
13) RNA tests: P53, P21, and P16
14) Metabolome analysis
15) 12-lead ECG: Pulse rate, PQ interval, QRS interval, and QT interval
16) Systolic and diastolic capacity measured by echocardiography test: LVEF, E wave, A wave, E/A, sep-e’, lat-e’, E/e’, LAVI, peak TRV, and HR
17) ^{123}I-MIBG myocardial scintigraphy-obtained sympathetic activity index: Heart-to-mediastinum ratio (H/M), and washout rate

1-5-4. Safety evaluation items

1) Adverse events and reactions (ARs) which developed during a period from initiation of administration until Week 24 (or study discontinuation).

1-6. Study schedule

Enrollment and allocation | Initiation of study drug administration

	Before treatment		Treatment period		
	Eligibility test	visit 1	visit 2	visit 3	Study discontinuation[*11]
	Week -12 to 0	Week 0<br>−	Week 12<br>± 4 weeks	Week 24[*10]<br>± 4 weeks	
Patient information/Obtaining of consent	○				
Patient background	○				
Interview/Physical examination	○	○	○	○	△
Confirmation of drug compliance			○	○	△
Confirmation of study procedure compliance		○	○	○	△
Confirmation of drugs in combination use	○	○	○	○	△
Height	○				
Body weight/Body temperature	○	○	△	○	△
Blood pressure/Pulse rate[*1]		○	△	○	△
Hematological test	○[*4, 5]	○[*4]	△	○	△
Hematological test (blood glucose/lipids)[*2]	○[*4, 5]	○[*4]	△	○	△
Hematological test (special)[*2]		○[*6, 7, 8]		○[*7, 8]	△[*7, 8]
12-lead ECG		○[*4]		○	△
Holter monitoring		○[*4]		○	△
Echocardiography	○[*4]	○[*4]		○	△
^{123}I-MIBG myocardial scintigraphy		△[*4, 8]		△[*8]	△[*8]
Adverse events[*3]	○	○	○	○	△
Evaluation of arrhythmia device		○[*9]		○	△

*1 to 11: See Section 8-1 for detail.

1-7. Target sample size and study duration planned

1) Sample size of patients: 210 (105 each for active drug and placebo)

2) Treatment observation: From the disclosure date in jRCT till October 2021 (Deadline of enrollment: April 2021)

3) Study duration: From the disclosure date in jRCT till October 2023

2 : Study background

The mortality rate of diabetes-complicated patients with cardiac disorder and the rate of rehospitalization for cardiac failure aggravation are both high, and diabetes is known to worsen prognosis of patients with cardiac disorder.[1)] This status similarly occurs in patients with implantable cardioverter defibrillator (ICD) implanted or with cardiac resynchronization therapy-defibrillator (CRT-D) implanted for treatment of arrhythmia, indicating that risky defibrillating operation and ICD-related death risk are increased.[2)]

Thus, treatment of diabetes is considered important in cardiac disorder patients implanted with such arrhythmia treatment devices, but the guidelines of the Japanese Circulation Society do not specifically recommend target level of blood glucose and diabetes drugs.[3,4)] In current status, the dosing regimen is decided at physician's discretion.

Sodium-dependent glucose transporter 2 (SGLT2) inhibitor is a hypoglycemic drug with new action mechanism, which selectively and reversibly inhibits glucose resorption by SGLT (expressed at proximal tubular site S1 to 2) at the proximal renal tubules to excrete excessive blood glucose into urine, independently of insulin secretion. In a recent EMPA-REG OUTCOME Study, it was shown that administration of SGLT2 inhibitor empagliflozin significantly suppressed cardiovascular death or vascular events (cerebral infarction and myocardial infarction, etc.).[5)] Although the study showed the pleiotropic favorable effects of SGLT2, including hypotensive effects and body weight reduction, the mechanism involved in outcome is not known, yet. We authors focus on increase in blood concentration of ketone body after administration of SGLT2 inhibitor.[6)] Ketone body produced mostly in the liver is expected to alleviate oxidative stress or suppress sympathetic nerves in the tissues,[7)] and is thus presumed to suppressively control arrhythmia in the heart (Refer to Fig. 1).

The primary objective in the present study is to demonstrate whether SGLT2 inhibitor improves or not the number of arrhythmia events, by evaluating the arrhythmia events in patients implanted with an arrhythmia device before and after intervention. In addition, ketone body and catecholamine are assayed to evaluate their relationship to arrhythmia. Taking it into consideration the EMPA-REG OUTCOME study results suggesting that Ht increase after administration of SGLT2 inhibitor was likely to be related to prognosis improvement, change in reticulocytes and erythropoietin related to those results is also evaluated. The authors also pay attention to change in aging-related substances and evaluate them together.

The present study is expected to contribute to establishment of better therapy for diabetes not only in patients with arrhythmia device implanted but also in diabetes-complicated patients with cardiac disorder.

Figure 1.

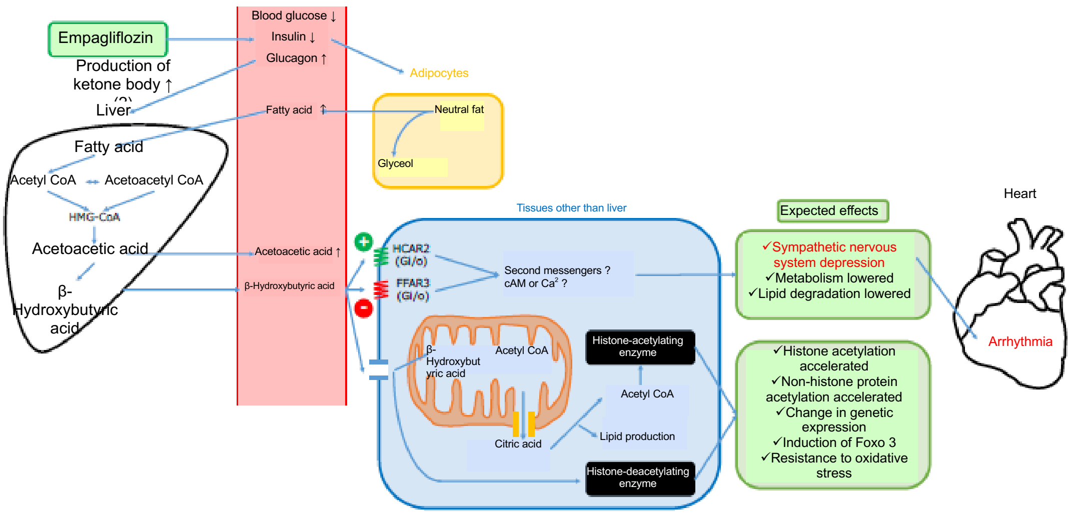

3 : Objective

The principal objective is to demonstrate whether or not empagliflozin improves the number of severe arrhythmia events in type 2 diabetes-complicated patients with arrhythmia device implanted.

Study hypothesis:

Administration of empagliflozin improves the number of severe arrhythmia events in type 2 diabetes-complicated patients with ICD or CRT-D implanted.

4 : Summary of study drug

The study drug shown below, supplied from Boehringer Ingelheim Japan, is used in this study.

4 - 1 . Active drug

Non-proprietary name: Empagliflozin

Chemical name: (1S)-1, 5-Anhydro-1-C-{4-chloro-3-[(4-{[(3S)-oxolan-3-yl]oxy} phenyl)methyl]phenyl}-D-glucitol

Brand name: Jardiance® Tablets 10 mg

Refer to the package insert (Appendix A) for detail.

Investigator or Subinvestigator treats patients according to the updated package insert, keeping in mind the revised revision and referring to Recommendation for Proper Use of

SGLT2 Inhibitors (See Section 7-8).

4-2. Placebo

Placebo is tablet apparently identical to that of active drug, not containing the effective ingredient.

5: Patients

5-1. Patients

Type 2 diabetes-complicated patients with arrhythmia device (ICD/CRT-D) implanted.

5-2. Inclusion criteria

1) Patients aged 20 years or more when the written consent was obtained (regardless of gender).
2) Patients who received ICD or CRT-D implantation surgery more than 24 weeks before the eligibility tests (regardless of implantation purpose: primary or secondary prevention)
3) Patients diagnosed with type 2 diabetes whom Investigator or Subinvestigator judged as possible to be administered empagliflozin (regardless whether drugs other than SGLT2 inhibitor are used to treat diabetes).
4) Patients who themselves granted consent to participation in the study in writing.

[Rationales for setting]

1) Adult patients able to grant consent to participation in the study by themselves, without setting the upper limit of age in order to evaluate in the same patient population as in routine clinical practice. Elderly patients should be carefully judged to be included in this study.
2) Patients with arrhythmia device implanted are selected, to be sure to record occurrence of severe arrhythmia.
3) When to newly diagnose with type 2 diabetes, Treatment Guide for Diabetes (2016-2017), ed. The Japan Diabetes Society[8)] is used. In the case where type 2 diabetes was previously diagnosed, the disease is what was diagnosed according to the diagnosis criteria in those days.
4) The setting complies with Clinical Trial Act in Japan.

[Treatment Guide for Diabetes[8)]]

1) Diabetes type is diagnosed when any one of 1 to 4 glycemic state below is confirmed

by the initial tests.

① Early morning fasting blood glucose level: ≥ 126 mg/dL

② Two-hour blood glucose level after 75 g OGTT: ≥ 200 mg/dL

③ Casual blood glucose level: ≥ 200 mg/dL

④ HbA1c: ≥ 6.5%

2) Diagnosis with diabetes is possible, if diabetes type could be reconfirmed by the tests on another day. However, the blood glucose level is essential to meet the diabetes criteria in either of the initial or repeated tests.

3) If simultaneous measurement of blood glucose and HbA1c is both sure to indicate diabetic type, diabetes can be diagnosed only by the initial tests.

4) Diagnosis with diabetes is possible only in the initial tests, if blood glucose level indicates diabetes type and one of the following symptoms is recognized.

① Typical diabetic symptoms such as thirst, polydipsia, polyuria, and weight loss.

② Definite diabetic retinopathy

5-3. Exclusion criteria

1) Patients using SGLT2 inhibitor at the time of eligibility tests.

2) Patients who were administered SGLT2 inhibitor within 24 weeks before the eligibility tests.

3) Patients with past history of hypersensitivity to empagliflozin (Jardiance® Tablets).

4) Patients with past history of diabetic ketoacidosis, diabetic coma, or hypoglycemic attack (hypoglycemia or protracted hypoglycemia requiring intervention by a third person) within 24 weeks before the eligibility tests.

5) Patients severely infected at the time of eligibility tests, or seriously injured before surgery (excluding surgical operation to replace battery cell).

6) Patients diagnosed with type 1 diabetes.

7) Patients with cardiac failure of NYHA Class Ⅳ.

8) Patients with severe renal impairment (eGFR less than 30 mL/min/1.73 m^2 within 3 months after consent was obtained or receiving renal dialysis).

9) Patients with serious hepatic function disorder (AST or ALT more than 3 times as high as the institutional standards within 3 months after consent was obtained).

10) Patients with pituitary or adrenal insufficiency.

11) Patients under malnutritional or fasting condition, irregularly taking foods, with poor dietary intake or debilitated.

12) Patients with history of excessive alcohol intake.

13) Patients who have gastrointestinal disorder like diarrhea or vomiting at the time of eligibility tests and are likely to be dehydrated.

14) Patients with urinary tract or genital infection at the time of eligibility tests.

15) Patients who are pregnant, likely to be pregnant, or breastfeeding at the time of eligibility tests or want to be pregnant while participating in this study.

16) Patients whose BMI is less than 18.5 kg/m^2 at the time of eligibility tests.

17) Patients in whom the following events likely to affect onset of severe arrhythmia were observed within 24 weeks before the eligibility tests: alteration of antiarrhythmic drug, catheter ablation for ventricular arrhythmia, coronary revascularization, open-heart surgery, development of coronary artery disease, stroke or transient ischemic stroke seizure, infection requiring hospitalization, or cardiac failure requiring hospitalization.

18) Patients who are using an arrhythmia device unable to record NSVT.

19) Patients who are complicated by non-remitted malignant tumor at the time of eligibility tests.

20) Patients whom Investigator or Subinvestigator judges as inappropriate to participate in this clinical study.

[Rationales for setting]

1) and 2) Likely to affect the evaluation endpoints.

3) to 15) The package insert requires this drug to be contraindicated or carefully administered. Previous studies[9,10] show the safety in an eGFR range from 30 to 45 mL/min/1.73 m^2.

16) Administration of SGLT2 inhibitor is reported to cause weight loss.

17) and 18) Likely to affect the evaluation endpoints.

19) Patient’s safety is taken into consideration. If remitted at enrollment, the patient is not applicable to the exclusion criteria.

20) Likely to affect the study results, if compliance with the study protocol is concerned because the patient is usually poor in drug adherence or highly likely to miss visit to the hospital, eventually to drop out of the study. Investigator or Subinvestigator makes final decision whether to exclude or not.

5-4. Discontinuation criteria

Should one of the following events occur after enrollment, the study is discontinued. The observation, test, and evaluation are carried out as soon as possible after study discontinuation.

1) When Investigator or Subinvestigator judges that the study is hard to be continued because the primary disease or complication is aggravated.
2) When Investigator or Subinvestigator judges that the study is hard to be continued because of adverse event onset.
3) When Investigator or Subinvestigator judges that the study is hard to be continued because the patient moved.
4) When Investigator or Subinvestigator judges that the study is hard to be continued because the patient moved to another hospital.
5) Patient requires discontinuation of participation in the study or withdrawal of consent.
6) SGLT2 inhibitor was taken after study drug was administered.
7) When the following events likely to influence onset of severe arrhythmia are observed: alteration of antiarrhythmic drug, catheter ablation for ventricular arrhythmia, coronary revascularization, open-heart surgery, development of coronary artery disease, stroke or transient ischemic stroke seizure, infection requiring hospitalization, or cardiac failure requiring hospitalization.
8) When Investigator or Subinvestigator judges that the study is hard to be continued for reasons other than above.

When to discontinue the study for reasons cited above, the reasons why it was judged hard to continue the study are clearly stated in medical record. In addition, when a patient requires discontinuation of participation in the study or withdrawal of consent, withdrawal of consent in writing is obtained from the patient him-or herself, using “Consent Withdrawal Form” (Appendix B).

６: Informed consent

When to initiate the study, “Informed Consent” is obtained in advance according to the updated “Clinical Trial Act.”

６-１. Procedure to obtain informed consent

Investigator or Subinvestigator verbally and carefully explains to patients about the study content using the Certified Review Board (CRB)-approved “Patient Information” (Appendix B) prior to confirmation of the eligibility. Upon confirming that patients understood enough the explanation, Investigator or Subinvestigator asks them to participate in the study, and obtains Consent Form (Appendix B) voluntarily completed by patients themselves.

If consented, patients date and sign the completed Consent Form. Investigator or Subinvestigator who explained also date and sign the form. Even when a clinical research coordinator (CRC) complements the explanation, CRC also dates and signs the form.

Patients themselves receive a duplicate of “Patient Information” and “Consent Form.” Investigator retains the original Consent Form at the participating medical institution for 5 years after the study was reported to be discontinued or completed.

6-2. In case where information likely to affect participation in the study was obtained

If information likely to affect patient’s volition to continuously participate in the study was obtained during the study period, Investigator or Subinvestigator immediately explains the information to patients, again to confirm whether patients are willing to continuously participate in the study. Then, patient information is simultaneously revised, reviewed at CRB, and approved at the participating medical institution. Finally, the consent to continuous participation in the study is again obtained from patients in writing.

7: Study methods

7-1. Study design

Investigator-initiated multicenter, prospective, placebo-controlled, randomized, double-blind, parallel group comparison study

7-2. Study outline

Investigator or Subinvestigator obtains the consent from patients, confirms their eligibility, enrolls them, and initiates treatment according to the group allocation. Patients are randomly allocated to the empagliflozin and placebo groups, and administered study drug for 24 weeks under double-blinded condition. Patients participate in the study during a period from obtaining of consent until completion of the treatment observation period (Refer to Fig. 2).

Figure 2.

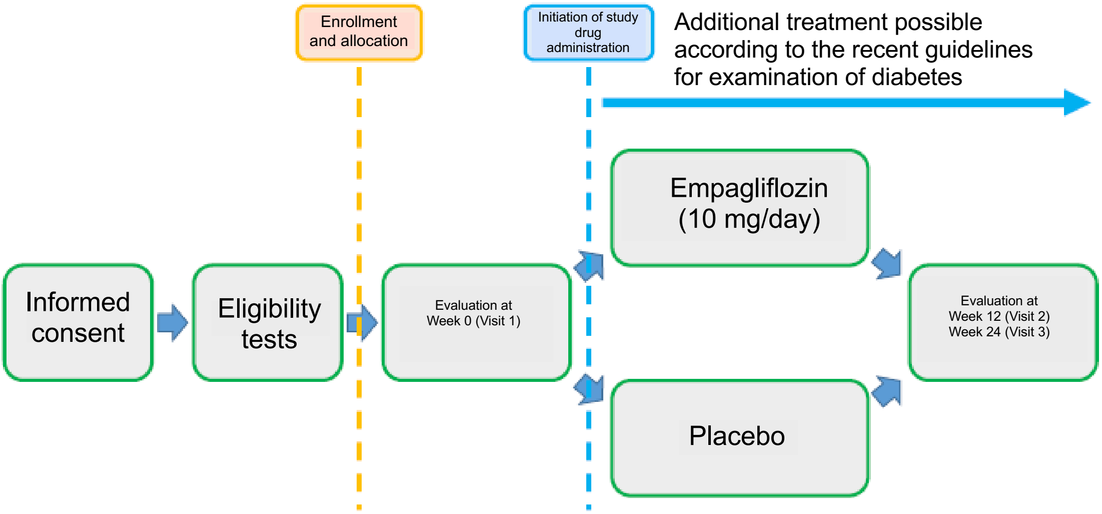

7-3. Target sample size and study duration planned

1) Sample size of patients: 210 (105 each for active drug and placebo)
2) Treatment observation: From the disclosure date in jRCT till October 2021 (Deadline of enrollment: April 2021)
3) Study duration: From the disclosure date in jRCT till October 2023

7-4. Patient enrollment and allocation

Patient enrollment and allocation follows the centralized method. After confirming the patient's eligibility, Investigator or Subinvestigator soon accesses Web site for patient enrollment, and inputs and transmits information necessary for enrollment. The transmitted eligibility is immediately confirmed on the Web site and registered in the database, if eligible, thereafter giving patient enrollment No. and treatment allocation. If ineligible, it is notified to that effect.

Patients are allocated to the treatment groups at a ratio of 1:1 by the stratified allocation method using the following factors. A person responsible for allocation of study drug decides the allocation scheme.

Stratification factors

1) LVEF
2) Age
3) Gender

7-5. Blinding

This study is designed as double-blind. After randomization, patients, Investigator, Subinvestigator, and study staffs involved in this study all can not know the allocation results until completion of data fixation. After completion of the study, the person responsible for allocation of study drug opens the key (unblind) after fixation of the database.

The blinding codes are opened, only under an emergency situation where an appropriate medical treatment is necessary for serious adverse events or the patient's safety is necessary to be secured. If the key was opened, it is immediately reported to Study-Representing Physician, when participation in the study is discontinued.

7-6. Treatment method

Following allocation of patient enrollment No., study drug is delivered from Niigata University. Investigator or Subinvestigator at each participating medical institution begins treatment after receiving the study drug. Administration of the study drug is continued for 24 weeks.

7-7. Actions taken for additional treatment

When Investigator or Subinvestigator judged that the blood glucose level is insufficiently controlled, it is allowed to use or increase diabetes drugs other than SGLT2 inhibitor, although attention should be carefully paid to adverse reactions such as hypoglycemia or dehydration. The therapeutic purpose follows Treatment Guide for Diabetes (2016-2017)[8] in Japan. Special attention is necessary to be paid to elderly patients, because the target blood glucose level may be different. After completion of the treatment period, treatment is continued with an optional diabetes drug (Refer to Fig. 3).

Figure 3.

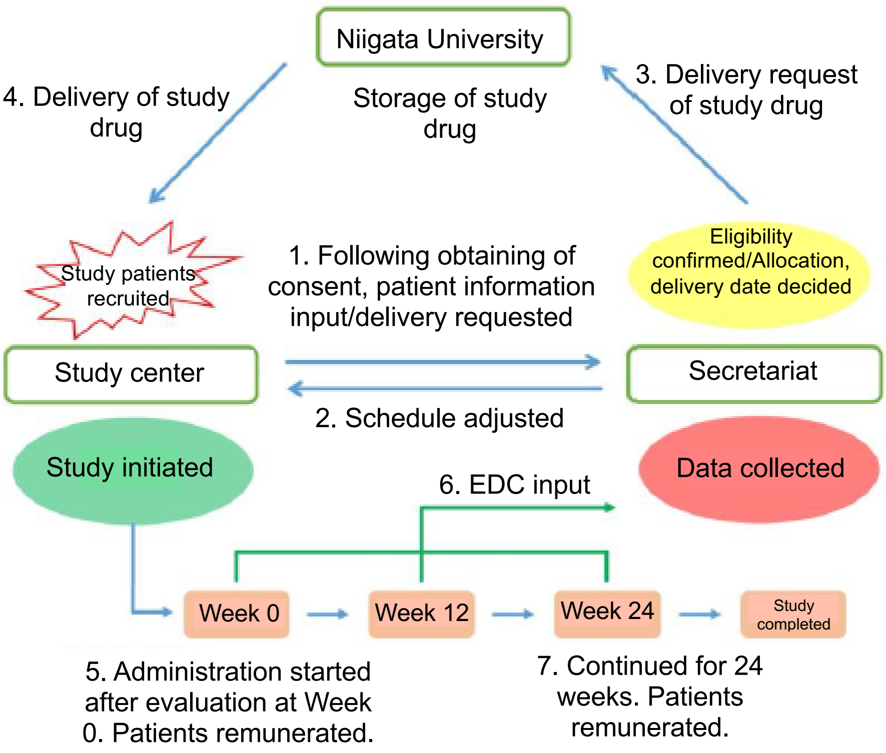

1) Empagliflozin group:

Empagliflozin 10 mg is once daily orally administered before or after breakfast.

2) Placebo group:

Placebo is once daily orally administered before or after breakfast.

In both groups, treatment with empagliflozin is combined with only or some of SU drug, rapid-acting insulin secretagogue, α-glucosidase inhibitor, biguanides, thiazolidine drug, DPP-4 inhibitor, GLP-1 receptor agonist, insulin preparation, or diet/exercise therapy. Selection of treatment is at Investigator or Subinvestigator discretion.

[Therapeutic target for diabetes[8]]

To normalize blood glucose level[Note 1)]: HbA1c less than 6.0%

To prevent complication[Note 2)]: HbA1c less than 7.0%

When hard to intensify treatment[Note 3)]: HbA1c less than 8.0%

The treatment target is individually set up, taking into consideration age, disease duration, organ damage, risky hypoglycemia, or support system.

Note 1): If possible to achieve only by an appropriate diet/exercise therapy, or by drug therapy without causing adverse reactions like hypoglycemia.

Note 2): The target level of HbA1c is less than 7.0% from a viewpoint of prevention of complication. The applicable blood glucose level is roughly less than 130 mg/dL at fasting and less than 180 mg/dL 2 hrs after a meal.

Note 3): When hard to intensify treatment because of adverse reaction like hypoglycemia or other reasons.

[Target level of blood glucose in elderly diabetes patients[8]]

The therapeutic target in elderly patients is individually set up, even taking into consideration cognitive function, basic ADL, instrumental ADL, and comorbidities, in addition to age, disease duration, risky hypoglycemia, and support system. However, attention should be enough paid to severe hypoglycemia which becomes more risky in association of aging.

■ Patient characteristics and health condition.[Note 1)]

Category I: ① Normal cognitive function and ② independence of ADL

Category II: ① Mild cognitive impairment to mild dementia or ② instrumental ADL lowered and independence of basic ADL

Category III: ① Moderate or worse dementia, or ② basic ADL lowered, or ③ many comorbidities and functional disorder

■ When drugs are concerned about induction of severe hypoglycemia (e.g., insulin preparations, SU drugs, glinides) are not used.[Note 2)]

Category I: HbA1c less than 7.0%

Category II: HbA1c less than 7.0%

Category III: HbA1c less than 8.0%

■ When drugs are concerned about induction of severe hypoglycemia (e.g., insulin preparations, SU drugs, glinides) are used.[Note 3)]

Category I: HbA1c less than 7.5% (lower limit: 6.5%) in patients aged 65 years or more and less 75 years, and less than 8.0% (lower limit: 7.0%) in patients aged 75 years or more

Category II: HbA1c less than 8.0% (lower limit: 7.0%)

Category III: HbA1c less than 8.5% (lower limit: 7.5%)

Note 1: Refer to Homepage of The Japan Geriatrics Society (http://www.jpn-geriat-soc.or.jp/) for evaluation of cognitive function and basic ADL (for example, dressing, walking, bathing, and using toilet) and instrumental ADL (for example, shopping, preparing meals, medication management, and monetary control). Under the end-of-life condition, treatments to prevent

marked hyperglycemia and subsequent dehydration and acute complications are prioritized.
Note 2: Even in the case of elderly patients with diabetes, the target level of HbA1c to prevent complications is less than 7.0%. However, if the target can be fulfilled by an appropriate dietary or exercise therapy alone or without adverse reactions of drug therapy, the target HbA1c level is less than 6.0%, and if hard to intensify the treatment, the target is less than 8.0%. In these cases, no lower limit is set up. Under a condition applicable to Category III, if combination use of multiple drugs is concerned about adverse events or social support is not enough for serious comorbidity, the target is allowed to be less than 8.5%.
Note 3: When prevention from onset or progression of complication is preferential in consideration of the morbidity duration of diabetes, the target level or lower limit can be set up for individual elderly patients, while taking measures to prevent severe hypoglycemia. When these drugs have been used since before reaching age of 65 years and the blood glucose is below the target level or lower limit shown in Figure, the current situation is maintained, basically, paying attention to occurrence of severe hypoglycemia. Glinides may be classified into a drug not concerned about severe hypoglycemia, in consideration of type, dose, and blood glucose level

[Important precautions]

Before using diabetes drugs, refer to "Guidelines for medical treatment and its safety in the elderly" ed. The Japan Geriatrics Society. When to use these drugs, be careful with onset of adverse reactions, avoiding use of multiple drugs.

7-8. Provisions for handling of study drug (Procedure for management and delivery)

1) Management of study drug: Study drug (active drug and placebo) is delivered all at once to Clinical and Translational Research Center (CTRC), Niigata University Medical & Dental Hospital, from the study drug supplier, and temporarily stored there. A table of vials numbered for active drug and placebo is together delivered. After the study drug vials were delivered, a person responsible for study drug management packs them by 7 vials/patient (for use in 6 months + extra 1 month), cross-checking with the table.

2) Distribution of study drug: The study drug vials are distributed to each participating medical institution by an independent person responsible for study drug management of CTRC, Niigata University Medical & Dental Hospital.

* Refer to SOP for study drug management for detail.

7-9. Combination treatment

Attention should be paid to use of drugs below in both of the treatment groups during the

study period.

1) Combination use-prohibited drugs: all SGLT2 inhibitors.

2) Precautions for coadministration: Drugs whose interaction with empagliflozin is concerned can be used in combination, but attention should be paid to onset of adverse reactions/complications due to interaction.

- Diabetes drugs: e.g., SU drug, rapid-acting insulin secretagogue, α-glucosidase inhibitor, biguanides, thiazolidine drug, pioglitazone, DPP-4 inhibitor, GLP-1 receptor agonist, and insulin preparations.
- Drugs intensifying action to lower the blood glucose level: e.g., β-blockers, salicylates, and monoamine oxidase inhibitor
- Drugs attenuating action to lower the blood glucose level: e.g., adrenalin, corticosteroid, and thyroid hormone.
- Drugs with diuretic action: e.g., loop diuretics and thiazide diuretics

Should a combination use-prohibited drug be used, a measure is taken according to the procedure for “Control of incompatibility (Section 15-6).” To fulfill the study, refer to the updated “Recommendation for Proper Use of SGLT2 Inhibitors.”

Recommendation for Proper Use of SGLT2 Inhibitors:

１. To use in combination with insulin secretagogues like insulin and SU drug, their dose is reduced, paying full attention to hypoglycemia. Patients should be of course educated enough about hypoglycemia.

２. Administration should be careful, if patients are aged 75 years or more or aged 65 to 74 years with geriatric syndrome (sarcopenia, cognitive function lowered, or ADL decreased).

３. An enough measure should be taken for prevention of dehydration, even including explanation to patients. If a diuretic is used in combination, attention should be paid specifically to dehydration.

４. Drugs should be absolutely withdrawn when food intake is not enough because of pyrexia, diarrhea, or vomiting or because of inappetence (sick day).

５. If accompanied by systemic malaise, nausea, vomiting, or weight loss, blood ketone body should be checked: ketoacidosis is likely even though the blood glucose level looks normal.

６. Should cutaneous symptoms like erythema suspected of drug eruption appear, administration should be immediately discontinued, to consult with Department of Dermatology. Adverse reactions should be sure to be reported.

7. To discover urinary tract infection/genital infection, patients should be appropriately interviewed. It is recommended to use questionnaire while interviewing. If discovered, consult with Department of Urology or Gynecology.

SGLT2 Inhibitor Proper Use Committee, The Japan Diabetes Society, revised May 12, 2016.
*Refer to the updated package insert or recommendation by the society, when to follow up.

8 : Observation and test items

8 - 1 . Schedule for observation and tests (Refer to Table 1)

Table 1:

Enrollment and allocation (between Eligibility test and visit 1) | Initiation of study drug administration (between visit 1 and visit 2)

	Before treatment		Treatment period		
	Eligibility test	visit 1	visit 2	visit 3	Study discontinuation*11
	Week -12 to 0	Week 0	Week 12	Week 24*10	
		–	± 4 weeks	± 4 weeks	
Patient information/Obtaining of consent	○				
Patient background	○				
Interview/Physical examination	○	○	○	○	△
Confirmation of drug compliance			○	○	△
Confirmation of study procedure compliance		○	○	○	△
Confirmation of drugs in combination use	○	○	○	○	△
Height	○				
Body weight/Body temperature	○	○	△	○	△
Blood pressure/Pulse rate*1		○	△	○	△
Hematological test	○*4, 5	○*4	△	○	△
Hematological test (blood glucose/lipids)*2	○*4, 5	○*4	△	○	△
Hematological test (special)*2		○*6, 7, 8		○*7, 8	△*7, 8
12-lead ECG		○*4		○	△
Holter monitoring		○*4		○	△
Echocardiography	○*4	○*4		○	△
^{123}I-MIBG myocardial scintigraphy		△*4, 8		△*8	△*8
Adverse events*3	○	○	○	○	△
Evaluation of arrhythmia device		○*9		○	△

○, Essential; △, Optional

*1: Blood pressure/pulse rate is measured in sitting position after resting for more than 5 min as a general rule.

*2: Fasting blood is sampled after resting in supine position on the bed for 30 min as much as possible. In this study, fasting blood sampling is defined as below. If not sampled while fasting and resting, it should be recorded to that effect.

[When blood is sampled before noon]
Blood sampling more than 10 hrs after the last meal: Blood is sampled without taking meal after dinner the day before (no breakfast on the day).
[When blood is sampled after noon]
Blood sampling more than 5 hrs after the last meal: Blood is sampled without taking meal after breakfast on the day (no lunch on the day).

*3: Should adverse event develop, the rationales for judgment (e.g., test value) should be reported.

*4: The test results obtained within 12 weeks before the tests at Week 40 are can be utilized, even before the consent was obtained. The echocardiographic results at the time of eligibility tests are used for allocation to treatment group. Eligibility test results obtained within 3 months of informed consent are available even if they are obtained before 12 weeks prior to Week 0 tests.

*5: AST, ALT, and eGFR are included in the eligibility tests.

*6: The test results obtained after obtaining consent to Week 0 can be utilized.

*7: Special blood sample volume is as follows: 5 mL for ketone body fraction and erythropoietin test; 7 mL for fraction test for catecholamine; 2 mL for reticulocyte sampling; 10 mL for DNA tests (telomere length): 5 mL for RNA tests (P53, P21, and P16); and 5 mL for metabolome analysis. In the DNA or RNA tests, genes categorized into personal information are not analyzed.

*8: Some of blood samplings for DNA tests (telomere length), RNA tests (P53, P21, and P16), and metabolome analysis, and ^{123}I-MIBG myocardial scintigraphy are optional.

*9: If arrhythmia device was evaluated during a period from consent obtaining until Week 0, the results can be utilized, including even the evaluation results obtained by using the remote monitoring system.

*10: The test results evaluated within 4 weeks before and after Week 24 can be utilized. However, the arrhythmia device is checked at Week 24, and correctly recorded, including even the evaluation results obtained by using the remote monitoring system.

*11: When the study was discontinued, the tests are carried promptly out as much as possible.

8-2. Observation and test items

Investigator or Subinvestigator records the following observation and test results in Case Report Form (CRF). In this study, electronic data capture (EDC) is used to report to Data Center. The transmission data do not include information able to identify the patient (Refer to Section 11. Data collection).

1) Patient background at the time of eligibility test.

The following are investigated.

Gender, age, smoking history, alcohol-drinking history, lethal arrhythmia which caused implantation with arrhythmia device (ventricular fibrillation, monomorphic ventricular tachycardia, polymorphic ventricular tachycardia, and non-sustained ventricular tachycardia) and underlying diseases (ischemic heart disease, cardiac valve disease, dilated cardiomyopathy, hypertrophic cardiomyopathy, restrictive cardiomyopathy, arrhythmogenic right ventricular cardiomyopathy, left ventricular non-compaction, mitochondrial cardiomyopathy, other cardiomyopathy, Brugada syndrome, long QT syndrome, and idiopathic ventricular fibrillation, etc.), maker and type of arrhythmia treatment device, comorbidity and past history (atrial fibrillation, hypertension, dyslipidemia, cerebral infarction or cerebral hemorrhage), oral drugs (all).

Past history of non-drug treatment: PCI, CABG, cardiac valvulopathy surgery, and catheter ablation, etc.

2) Interview and physical examination: at the time of eligibility test, and Week 0, 12, and 24 (or study discontinuation).

Interviewed about to judge NYHA class.

3) Confirmation of drug compliance: at Week 12 and 24 (or study discontinuation).

If forgotten to take study drug, input EDC to that effect.

4) Confirmation of study procedure compliance in the protocol: at Week 0, 12, and 24 (or study discontinuation).

To check whether the tests and treatment adhere to the study protocol.

5) Confirmation of drugs in combination use: at Week 0, 12, and 24 (or study discontinuation).

To confirm whether the drug is prohibited to be used in combination or cautioned to be coadministered. In addition, all drugs orally taken at every time point are inspected for the type and dose (for study compliance and safety evaluation).

6) Height, body weight, and body temperature: at the time of eligibility test and Week 0 and 24 (or study discontinuation) (*Optional at Week 12).

Height, body weight, and body temperature in the examination room are measured at visit to the hospital. Height is measured only at the time of eligibility tests.

7) Blood pressure and pulse rate: at Week 0 and 24 (or study discontinuation) (*Optional at Week 12).

Blood pressure (SBP/DBP) and pulse rate are measured in sitting position after resting for more than 5 min, as a general rule.

8) Hematological tests: at the time of eligibility tests (AST, ALT, Cr, and eGRF [estimated values] only) and Week 0 and 24 (or study discontinuation) (*Optional at Week 12).

Hematological (RBC count, WBC count, Hb, Ht, and Plt), blood biochemical (total protein, albumin, AST, ALT, ALP, T-Bil, LDH, BUN, Na, K, Cl, uric acid, Cr, and estimated eGFR), and BNP.

The eligibility tests include AST, ALT, and eGFR.

9) Hematological tests (blood sugar and lipid): at the time of the eligibility tests (only HbA1c) and Week 0 and 24 (or study discontinuation) (*Optional at Week 12)

Blood glucose tests: fasting blood glucose and HbA1c

Lipid tests: TG, TC, HDL-C, and LDL-C

10) Hematological tests (special): at Week 0 and 24 (or study discontinuation) （*Blood sampling for DNA tests (telomere length), RNA tests (P53, P21, and P16), and metabolome analysis are optional）

Blood ketone body fraction: acetoacetic acid, 3-hydroxybutyric acid, and total ketone bodies

Blood catecholamine fraction: adrenalin, noradrenalin, and dopamine

Erythropoietin and reticulocytes

DNA tests: Telomere length

RNA tests: P53, P21, and P16

Metabolome analysis

Refer to Appendix C (Procedure for special blood tests) for test procedure in detail.

*Blood sample volume: 8) + 9) = 17 mL and 10) = 34 mL.

11) 12-lead ECG tests: at Week 0 and 24 (or study discontinuation)

Heart rate, rhythm, PQ interval, QRS interval and QT interval

12) Holter monitoring: at Week 0 and 24 (or study discontinuation)

Total recoding time, total No. of VPC, No. of single VPC, No. of two-consecutive VPC, and No. of ventricular tachycardia

Electrocardiogram is analyzed by cardiologists. Refer to Appendix D (Procedure for Holter monitoring) for the test procedure in detail.

13) Echocardiographic tests: at the time of eligibility tests and Week 0 and 24 (or study discontinuation)

Systolic capacity (LVEF) and diastolic capacity (LVEF, E wave, A wave, E/A, sep-e', lat-e', E/e', LAVI, and peak TRV) are measured: Biplane disk summation method (modified Simpson method) is recommended. For e', both sep-e' and lat-e' are measured. For LAVI, LAV is first measured and corrected by body surface area at

each time point. HR at measurement time is also reported. Refer to Appendix E (Procedure for echocardiographic tests) for the test procedure.

14) ^{123}I-MIBG myocardial scintigraphy tests: at Week 0 and 24 (or study discontinuation) (*Optional).

Front planar image is used to calculate the H/M ratio and washout rate. For the test methods, refer to Appendix F (Procedure of ^{123}I-MIBG myocardial scintigraphy).

15) Adverse events: at the time of eligibility tests and Week 0, 12, and 24 (or study discontinuation).

The presence or absence of symptoms applicable to adverse events and findings are confirmed at the time of examinations. For definition of these and reporting procedure, refer to Appendix G (Procedure to report adverse events).

16) Evaluation of arrhythmia device: at Week 0 and 24 (or study discontinuation).

Week 0: During a period of 24 weeks before Week 0, severe arrhythmia (NSVT/VT/VF), No. of events to appropriately treat severe arrhythmia (anti-tachycardia pacing/shock operation), and biological monitoring indicators are confirmed. In the case where atrial lead is inserted, the number of atrial fibrillations events is also confirmed.

Week 24: Severe arrhythmia (NSVT/VT/VF), No. of events to appropriately treat severe arrhythmia (anti-tachycardia pacing/shock operation), and biological monitoring indicators are confirmed during a period of 24 weeks after intervention. In the case where atrial lead is inserted, the number of atrial fibrillations is also confirmed.

If the setting was changed, what was changed is input in EDC, including the reasons.

9： Evaluation endpoints

9-1. Primary endpoint

The number of severe arrhythmia events (NSVT/VT/VF) recorded in the arrhythmia device is evaluated at Week 0 and 24 (or discontinuation), to calculate the following: Difference in No. of severe arrhythmia events between the empagliflozin and placebo groups.

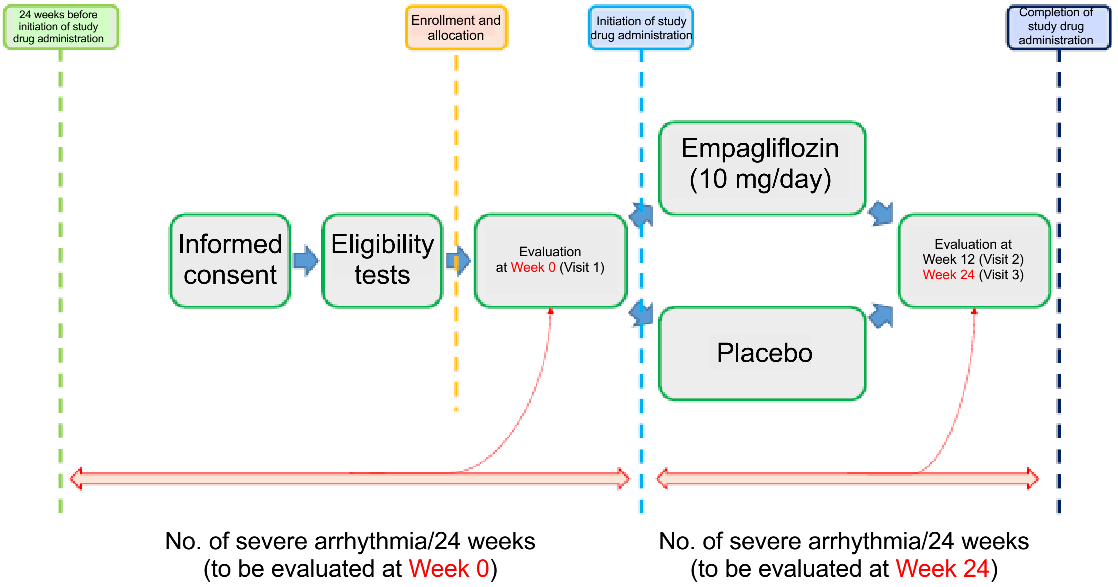

[Severe arrhythmia]

Severe arrhythmia and its appropriate treatment are confirmed by probing information recorded in the arrhythmia device through the remote monitoring system or by programmer's directly retrieving. Actually, when intracardiac ECG is checked to differentially diagnose the type of severe arrhythmia and to treat, type of treatment and appropriate or inappropriate device operation are judged. Each type of arrhythmia is defined as bloew.[7)]

✓Nonsustained ventricular tachycardia (NSVT): Among arrhythmia cases auto diagnosed as NSVT according to the auto diagnosis standards of the device used for individual patients, what cardiologists judges the auto diagnosis as correct. In general, the cases where arrhythmia was sustained more than 5-consecutive VPC and disappeared by ten-consecutive VPC, without being treated by ICD, are auto diagnosed as NSVT in many cases.

✓Ventricular tachycardia (VT)/ventricular fibrillation (VF): Among arrythmia cases auto diagnosed as VT/VF according to the auto diagnosis standards of the device used for individual patients, what cardiologists judges the auto diagnosis as correct.

[Appropriate treatment]

Only when above lethal arrhythmia was treated, meeting the respectively set treatment standards, the treatment is judged as appropriate. When non-lethal arrhythmia (atrial fibrillation or supraventricular tachycardia) was mistakenly treated, the treatment is judged as inappropriate treatment.

[Setting of arrhythmia device]

It is highly likely to disadvantage patients to make identical the setting of arrhythmia monitoring and treatment, the setting is adjusted to individual cases and decided by cardiologists. During the study period, the device setting is not immoderately changed, but can be changed only when a cardiologist in charge of the treatment judges as necessary to change.

[Evaluation of arrhythmia event]

Event Assessment Committee is externally set up to individually evaluate the arrhythmia events data, whether to be appropriate or not, and the results are reported to Study-Representing Physician.

9-2. Secondary endpoints

1) No. of severe arrhythmia events (NSVT/VT/VF) and appropriate device operations (anti-tachycardia pacing/shock operation) recorded in the device are evaluated at Week 0 and 24 (or study discontinuation), to calculate the following.

(1-1) Change in the number of severe arrhythmia events

(1-2) Incidence of severe arrhythmia event before and after treatment

(1-3) No. of severe arrhythmia events at Week 24

(1-4) Presence or absence of severe arrhythmia events at Week 24

(1-5) Change rate of No. of appropriate device operations

(1-6) Change in No. of appropriate device operation
(1-7) Incidence of appropriate device operation before and after treatment
(1-8) No. of appropriate device operations at Week 24
(1-9) Presence or absence of appropriate device operation at Week 24

These variables are compared between the empagliflozin and placebo groups.

In addition, the following are evaluated at Week 0 and 24 (or study discontinuation), to compare the change rate, change, and values at Week 24 between the empagliflozin and placebo groups.

2) Holter monitoring: Total recording time, total No. of VPC, No. of single VPC, No. of two-consecutive VPC, and No. of ventricular tachycardia.
3) Blood ketone body fraction (acetoacetic acid, 3-hydroxybutyric acid, and total ketone bodies): Blood concentration of each ketone body.
4) Blood catecholamine fraction (adrenalin, noradrenalin, and dopamine): Blood concentration of each catecholamine.

The correlation between difference in No. of severe arrhythmia events and blood ketone body or catecholamine is evaluated in comparison between the empagliflozin and placebo groups.

9-3. Exploratory evaluation endpoints

1) The number of atrial fibrillation events and biological monitoring index recorded in the arrhythmia device is evaluated at Week 0 and 24, to calculate the following.

(1-1) Change rate of No. of atrial fibrillation event
(1-2) Change in No. of atrial fibrillation events
(1-3) Incidence of atrial fibrillation event before and after treatment
(1-4) No. of atrial fibrillation events at Week 24
(1-5) Presence or absence of atrial fibrillation event at Week 24
(1-6) Change rate of biological monitoring index
(1-7) Change in biological monitoring index
(1-8) Biological monitoring index at Week 24

These are compared between the empagliflozin and placebo groups.

In addition, the following items are evaluated at Week 0 and 24 (or study discontinuation), to compare the change rate, change, and value at Week 24 between the empagliflozin and placebo groups.

2) NYHA classification of cardiac function
3) Body weight

4) Blood pressure, pulse rate, and body temperature
5) Blood glucose: HbA1c and fasting blood glucose
6) Serum lipid, serum uric acid, and cardiac failure marker: TC, HDL-C, LDL-C, TG, UA, and BNP
7) Renal function: Serum Cr, eGFR (estimated), BUN, Na, K, and Cl
8) Hepatic function: Total protein, albumin, AST, ALT, ALP, T-Bil, and LDH
9) Hematological value: RBC count, WBC count, Hb, Ht, and Plt
10) Erythropoietin
11) Reticulocytes
12) DNA tests: Telomere length
13) RNA tests: P53, P21, and P16
14) Metabolome analysis
15) 12-lead ECG: Pulse rate, PQ interval, QRS interval, and QT interval
16) Systolic and diastolic capacity measured by echocardiographic test: LVEF, E wave, A wave, E/A, sep-e’, lat-e’, E/e’, LAVI, peak TRV, and HR
17) ^{123}I-MIBG myocardial scintigraphy-obtained sympathetic activity index: Heart-to-mediastinum ratio (H/M), and washout rate

９-４. Safety evaluation items

1) Adverse events and adverse reactions which developed during a period from the day of consent obtained until Week 24 (or study discontinuation).

１０: Measures to be taken for AE onset

１０-１. About adverse reaction (AE)

An adverse event is defined to be any untoward medical occurrence in patients administered a study drug (including laboratory abnormalities), which does not necessarily have to have a causal relationship with this study. Of such adverse events, those whose causal relationship to the present study cannot be ruled out are called "disease or the like." For onset of any adverse event, Investigator or Subinvestigator promptly takes appropriate measures to treat them, to secure the patient’s safety. In addition, when administration of study drug was discontinued or treatment was essential for adverse events, Investigator or Subinvestigator tells patients to that effect. For measures to be taken adverse events, refer to Appendix G (Procedure to report adverse events).

[Adverse reactions of empagliflozin expected from the package insert] (Refer to Appendix A)
Severe adverse reaction: hypoglycemia, dehydration, ketoacidosis, pyelonephritis, and

sepsis

Other adverse reactions: Infections (cystitis, urinary tract infection, asymptomatic bacteriuria, vulvovaginal candidiasis, trichomoniasis, bacterial vaginitis, vulvovaginitis, and Vaginal moniliasis); Reproductive disorders (balanitis, genital pruritus, balanoposthitis, vulvovaginal discomfort, and vulvovaginal pruritus); Metabolism and nutrition disorders (hyperlipidemia and fluid volume decreased); Blood and lymphatic system disorders (hemoconcentration); Nerve disorders (dizziness and dysgeusia); Gastrointestinal disorders (constipation and abdominal distention); Skin and subcutaneous tissue disorders (rash, pruritus, and urticaria); Renal and urinary disorders (pollakiuria, polyuria, urine output increased, and dysuria); General disorders (thirst and feeling hungry), and Investigations (weight decreased, urine ketone body present, and blood ketone body present)

According to the guidance of US Food and Drug Administration (FDA) (dated August 29, 2018), attention is paid even to onset of perineal necrotizing fasciitis (Fournier's gangrene) as a severe adverse reaction.

１０-２. Duration to collect information on adverse events

Adverse events are collected, which developed during a period from the day of consent obtained until Week 24 (or study discontinuation). Any onset of disease or the like is followed up until the outcome is established

１０-３. Investigation items of adverse events

Investigator or Subinvestigator investigates development of adverse events for the following items.

1) Name of adverse event and onset date
2) Seriousness
3) Specifically noteworthy adverse events
4) Treatment and outcome, and confirmation date of outcome
5) Causal relationship to the study
6) Administration status of study drug (to be continued or discontinued)

[Definition of serious adverse event]

① that results in death,
② that is life threatening,
③ that requires hospitalization for treatment or prolongation of existing hospitalization,
④ that results in persistent or significant disability or incapacity,

⑤ that results in a congenital anomaly, and
⑥ Other events or reactions considered medically significant

A wording of “Considered medically significant” in No. 6 above refers to an event which is appropriately medically judged as endangering the patient, requiring medical or surgical treatment so as not to lead to serious adverse events defined in No.1 to 5 above. Patients may be hospitalized during the study period for study management or social reasons (ex. because of the day to implement fluid infusion, or not day trip treatment but hospitalization necessary: the hospital is far away to visit). This case of hospitalization or other cases where hospitalization was scheduled at initiation of the study are already reported by the source data at the time of eligibility tests. Therefore, these cases are not necessary to be reported as serious adverse event, if conduced as scheduled.

[Definition of specifically noteworthy adverse event]
① Liver disorder: AST, ALT, and T-Bil increased over the standards set up in this study.
② Kidney function decreased: Cr increased over the standard set up in this study.
③ Acidosis: Metabolic acidosis, ketoacidosis, and diabetic acidosis.
④ Leg amputation-associated events: Amputation, disarticulation, and autoamputation.
⑤ Perineal necrotizing fasciitis (Fournier's gangrene)

[Outcome]
Outcome is judged by 6 categories: “Recovered, Recovering, Not recovered, With sequelae, Death, and Unknown.” In the cases of Recovered, Recovering, or Death, the confirmation day of outcome is set as the day when the outcome was actually recognized. In the case where the follow-up is terminated because the outcome is Not recovered or Unknown, the confirmation day of outcome refers to the day when Investigator or Subinvestigator last confirmed the outcome, and the reasons for termination of follow-up or Unknown are filled out in the comment space.

[Causal relationship to study]
The causal relationship to study is judged by the following two categories. When judged as “Able to be ruled out,” the reason is filled out in the comment space.
① Causal relationship can be ruled out: In the cases where time relationship between adverse event and study is unreasonable, or where the cause of adverse event onset can be explained even by medical relevance other than the study.

② Causal relationship cannot be ruled out: In the cases not applicable to the above cases, adverse events whose causal relationship to the study cannot be ruled out are set as "diseases or the likes" pursuant to the Clinical Trials Act.

１０-４. Report of adverse events

(Refer to Appendix G (Procedure to report adverse events) for detail)

When onset of serious adverse event was recognized, Investigator or Subinvestigator promptly reports to Study-Representing Physician in writing. In addition, adverse events whose causal relationship to the study cannot be ruled out (diseases or the likes) are reported even to administrator of the participating medical institution according to the Clinical Trials Act. Of these, serious cases (serious diseases or the likes) are reported even to CRB, according to the provisions. In addition, communication to cooperate with the reports based on “Drug and Medical Device Safety Information Reporting System” based on “Act on Securing Quality, Efficacy and Safety of Products Including Pharmaceuticals and Medical Devices” and the spontaneous report by the manufacturing/marketing business license holder should be appropriately executed according to the provision of each participating medical institution. The methods for reporting are summarized as below.

1) Expedited (alert) report

In this study, serious adverse events (including serious diseases or the like) and specifically noteworthy adverse events are subjected to expedited report. When onset of relevant adverse event was recognized, Investigator or Subinvestigator immediately fills out Adverse Event Report Form as predetermined, reports to Study Secretariat by FAX or email, to input through EDC.

2) Routine report

In the cases where nonserious adverse events shown in “Section 10-3. Investigation items of adverse events” were recognized, Investigator or Subinvestigator inputs through EDC. During the study period, the outcome of relevant events is followed up as much as possible to report.

3) Report to administrator of the participating medical institution

Investigator and Subinvestigator share information on adverse events, and report the adverse events whose causal relationship to the study cannot be ruled out (diseases or the likes) even to administrator of the participating medical institution.

4) Report to Independent Data Monitoring Committee (IDMC)

When considered an adverse event to be serious and unable to be ruled out of the causal relationship to the study (serious disease or the like), Study-Representing Physician promptly reports to IDMC in writing, simultaneously asking to review the

appropriateness of measures taken for the relevant adverse event. The IDMC reviews the report, and reports the future responses including case handling or possible study continuation to Study-Representing Physician in writing

5) Report to CRB

When considered an adverse event to be serious and unable to be ruled out of the causal relationship to the study (serious disease or the like), Study-Representing Physician reports to CRB. In addition, Study-Representing Physician reports a nonserious adverse event to CRB, if it is specially noteworthy as compared to the cases in common clinical practice, even though unable to rule out the causal relationship to the study (nonserious disease or the like). Other nonserious diseases or the likes are periodically reported in writing.

6) Report to Ministry of Health, Labour and Welfare

Study-Representing Physician periodically reports to Ministry of Health, Labour and Welfare in writing, about the items shown in Article 59, Item 1-No.1 of Enforcement Regulations, according to Article 60, Enforcement Regulations of Clinical Trial Act, within one month after the day when CRB commented.

7) Report to Boehringer Ingelheim Japan

Upon receiving an expedited report on adverse event from Study Secretariat, Study-Representing Physician reports to Department of Pharmacovigilance, Boehringer Ingelheim Japan in writing within 24 hrs, including the Study-Representing Physician’s comments.

8) Duty of Study Secretariat

Study Secretariat who received a report from Investigator or Subinvestigator asks Study-Representing Physician about urgency, importance, and degree of influence, and also asks IDMC for judgment, as needed. In addition, if necessary, action is taken to temporarily discontinue patient enrollment or to inform the participating medical institution.

9) To inform the participating medical institutions

Study-Representing Physician communicates the relevant information on adverse event to all the participating medical institutions in response to the IDMC recommendation as needed, including decision to continue, change, or discontinue the study.

１１: Data collection

１１-１. Completion of case report form (CRF) and report

Investigator or Subinvestigator appropriately records the investigation items

predetermined by CRF in examination records or the likes, and stores. In this study, the data are input in EDC to report to Data Center, including no information allowing to identify patients. If any source document exists and its objectivity is assurable, and medical judgment is not exercised, trial collaborators may transcribe the data from source documents to EDC. Investigator or Subinvestigator will inspect, confirm and then electronically sign the contents of the prepared EDC. No personal information to identify patients will be included in such data.

１１-２. Data management

Data Center demands the data, scrutinize and inquire the data, and control, according to the data management plan, to provide the dataset for statistical analysis.

At the Data Center, a person responsible for data management confirms the data, and theoretically checks using computers according to the data management plan. Should inconsistency or missing data be detected, the responsible person asks Investigator or other study staff. In consequence, Investigator or other study staff corrects EDC, if necessary, according to the predetermined method.

The stage were all the data were cleaned is set as tentative data fixation and Case Review Committee is held, as needed. At the Data Center, the Case Review Committee results are reflected on EDC, to finally fix the data.

１２: Statistical analysis

Statistical analysis plain is otherwise provided in detail.

１２-１. Definition of analysis populations

１２-１-１. Full analysis set (FAS）

FAS means a population of all enrolled patients, excluding cases where the study protocol is seriously violated (consent not obtained or seriously deviating from the study procedure).

１２-１-２. Per protocol set (PPS)

PPS means a patient population from which such patients significantly violating study protocol-provided treatment or combination therapy as below are excluded from all enrolled patients.

- Violating the inclusion criteria
- Violating the exclusion criteria
- Violating combination use-prohibited drugs or therapies
- Violating drug compliance rate

１２-１-３. Safety analysis set (SAS)

SAS means a population of all enrolled patients.

１２-２. Analysis of primary endpoint

The number of severe arrhythmia events (NSVT/VT/VF) recorded in the arrhythmia device is evaluated at Week 0 and 24 (or study discontinuation), and subjected to Poisson regression analysis by the generalized linear model, assigning difference in No. of severe arrhythmia events between the empagliflozin and placebo groups as an objective variable.

１２-３. Analysis of secondary, exploratory, and safety endpoints

Descriptive summary statistics are calculated for each endpoint. In continuous variables, difference is estimated based on the least-squares method. In discrete variables, difference in the ratio is estimated based on chi-square distribution. Other analyses in detail are shown in the statistical plan otherwise provided.

１２-４. Significance level

The significance level is set to 5% on both sides.

１２-５. Handling of missing data

The missing data are not imputed as a general rule. Handling of each case is deliberated at Case Review Committee.

１３: Target sample size and rationales for setting

Sample size of patients: 210 (105 each for active drug and placebo)

[Rationales for setting]

The number of patients practically feasible to be enrolled is set to 210, calculating from the number of feasible medical institutions (in total of 20 institutions: 2 to 15 patients/institution). Assuming that 10 patients (5%) be dropped out of this population of patients, 200 patients are estimated to be included in the present study. Assuming the incidence ratio of severe arrhythmia in the placebo to empagliflozin group (IRR=1.44) based on the previous report[9)] and using the generalized linear model for difference in the number of arrhythmia, 99 patients/group are necessary to detect the IRR efficacy 1.44, when the significance level and power are set to 0.05 and 80%, respectively. In this case, the number of patients is within the actually possible number to recruit patients. Thus, the target number of patients was set to 210.

No. of patients necessary by power.

Power	No. of patient necessary
60%	124 (62+62)
70%	156 (78+78)
80%	198 (99+99)
90%	266 (133+133)

[No. of patients at each institution]

Name of institution	No. of patients prospected to be enrolled
Tachikawa General Hospital	10
Kitasato University Hospital	20
Kumamoto University Hospital	5
Gunma Cardiovascular Center	10
Kokura Kinen Hospital	10
National Cerebral and Cardiovascular Center	30
Saitama Medical University International Medical Center	10-20
Sakakibara Heart Institute	3-5
Sapporo Medical University Hospital	10
Jichi Medical University Saitama Medical Center	3
Jichi Medical University Hospital	10
Chiba University Hospital	10
University of Tsukuba Hospital	2
Tokyo Women's Medical University Hospital	10
Nagoya University Hospital	3-4
Niigata City General Hospital	10
Niigata University Medical & Dental Hospital	30
Hirosaki University Hospital	10
Fukushima Medical University Hospital	12
Hokkaido University Hospital	5
Nippon Medical School Hospital	10
Kanazawa University Hospital	10
Shiga University of Medical Science Hospital	5

Nagasaki University Hospital	3
University of Fukui Hospital	5
Yamagata University Hospital	3
Yamaguchi University Hospital	2
Iwate Medical University Hospital	3
Kyoto University Hospital	10
Niigata Prefectural Central Hospital	10
Juntendo University Hospital	10
Juntendo University Urayasu Hospital	10

１４: Ethics

In this clinical study, the human rights and welfare are protected in accordance with the ethical principles in the latest Helsinki Declaration and in compliance with the updated “Clinical Trial Act.”

１４-１. Ethical review

This clinical study is applicable to “Specified Clinical Research.” Thus, prior to initiation of the study, the study protocol is reviewed at CRB, where making an effort to hear the opinions and to take necessary measures, respecting the opinions, if commented. After the review, the approval application is made for implementation of this study at all the participating medical institutions. When the medical institutions all approved, the study is registered in jRCT, notified to MLHW, and then started.

１４-２. Protection of personal information

According to Article 27, Enforcement Regulation of Clinical Trial Act, any personnel involved in this study should carefully consider the patients’ privacy and personal information, and appropriately handle them. Specifically, patients enrolled at the time of eligibility tests are anonymized to protect personal information by giving enrollment No. in the study. In addition, Patient No. Table is made. EDC does not include information such as name and medical card No. able to identify patients. To publish the study results, no information able to identify patients is not included. The patients’ data obtained from the present study are not used for other than the objectives of this study. When used for other than the objectives of this study, consent is otherwise obtained from patients, as needed. If patients themselves require their own personal information to be disclosed, the relevant personal information is disclosed without any delay. In addition, if required to stop the data usage, utilization is discontinued without any delay.

１４-３. Compensation for health impairment

When this study caused some health impairment, the participating medial institution treats the health impairment or takes other necessary measures. The cost necessary for treatment is covered by public medical insurance which the patient joins.

In addition, study drug (active drug and placebo) used in this study is not a domestically available drug itself which Boehringer Ingelheim Japan imports and markets according to the Manufacturing and Marketing Approval, but what Study-Representing Physician personally imports from Boehringer Ingelheim Germany as a study drug. Therefore, any of health impairment caused by adverse reaction is not applicable to Relief System for Sufferers from Adverse Drug Reactions of the PMDA. The active drug used is as effective as Jardiance® Tablets 10 mg distributed in Japan.

In this study, patients join the clinical study insurance to be prepared for study-related health impairment, and compensated for death and residual disability, if any.

１４-４. Patient's benefit and disadvantage

This clinical study is carried out in common clinical practice for patients with type 2 diabetes. Because the active drug is administered according to the dosing regimen approved for this drug, patients are unlikely to be at risk over the routine clinical examination. However, adverse reaction may occur. In the placebo group, blood glucose is concerned to be poorly controlled, but additional administration is possible in that case (Refer to Sections 7-6 and 7-8).

In this study, blood volume to be sampled for the hematological tests is 17 mL for the routine tests and 34 mL for the special tests (e.g., blood ketone body fraction), increasing the necessary sampling volume by 17 to 51 mL/sampling.

In addition, patients are exposed to radioactivity in the ^{123}I-MIBG myocardial scintigraphy tests to evaluate the activity of cardiac sympathetic nerves. However, exposure to radiation in patients is too low, being unlikely to cause a certain influence. Furthermore, patients receive periodic detailed examinations during the study period, to check health condition.

１４-５. Patient's expenses

Examination cost other than study drug during the study period is paid by patients themselves or covered by the health insurance which the patient joins. The cost of "Empagliflozin 10 mg Tablets" and "Placebo Tables" supplied as study drug is paid from the funds for this study. The cost of Hematological tests (special) is also paid from the funds for this study. In addition, QUO Card equivalent to ¥20,000 is paid to each patient at Week 0 and

at completion of the study (Week 24 or study discontinuation).

１５: Quality control and assurance

１５-１. Source data

Source data provided by Clinical Trial Act is defined as below in this study.

１) Reference data to input EDC: Electronic/paper medical cards, nursing records, and test data, etc.

２) Some of EDC inputs: Matters of judgement and findings associated with causal relationship and seriousness of adverse events, which are directly input in EDC because the data applicable to 1) above are not available.

１５-２. Handling of data and samples

The clinical data recorded in source data and EDC or residual samples are retained at the participating medical institutions and Department of Cardiovascular Biology and Medicine, Niigata University Graduate School of Medical and Dental Sciences, for 5 years after the report date of study discontinuation or completion. The remaining samples are limitedly utilized, if new information was obtained and it was decide that additional analysis is necessary. The purposes to utilize the remaining samples and the storage duration should be agreed with patients in advance. To exchange samples and information among the participating medical institutions, Sample and Information Exchange Record is provided. After completion of the storage duration, they are appropriately disposed at the respective medical institutions.

Blood ketone body fraction (acetoacetic acid, 3-hydroxybutyric acid, and total ketone bodies) and blood catecholamine fraction (adrenalin, noradrenalin, and dopamine), erythropoietin, and reticulocytes are retrieved by SRL Inc. from the respective participating medical institutions for analysis. The remaining test samples are retrieved and stored at Department of Cardiovascular Biology and Medicine, Niigata University Graduate School of Medical and Dental Sciences.

In addition, the samples for metabolome analysis is once retrieved to Department of Cardiovascular Biology and Medicine, Niigata University Graduate School of Medical and Dental Sciences, and then forwarded to Institute for Advanced Biosciences, Keio University.

As DNA and RNA tests are performed in Cardiovascular Medicine, Graduate School of Medicine of Juntendo University, samples will be sent to the above facility after being collected by the Department of Cardiovascular Medicine, Niigata University Graduate School of Medical and Dental Sciences.

15-3. Record storage

The following documents essential for the specified clinical study are stored for 5 years by Investigator or Study-Representing Physician after the report data of study discontinuation or completion: Matters/data identifying patients or related to examinations and tests (source data, clinical data recorded in EDC, and related documents such as Patient No. Table), study protocol, study plan, explanation to patients, and consent-related documents (patient information), Clinical Study Report, review-related documents received from CRB, monitoring and audit-related documents, study contract, and summary of drugs to be used.

15-4. Monitoring and audit

This clinical study is applicable to "Specified Clinical Research," and monitored and audited by a third party, according to Clinical Trial Act, to secure the scientific quality and data reliability. In reality, the appropriate conduct of this study adhering to the protocol is confirmed by on-site inspection, e-mail, and telephone. Moreover, instead of on-site source data verification for materials, the confirmation of provided copies of materials and the confirmation of materials using TV conference system will be accepted. When the study conduct was monitored, a monitor completes the monitoring report, and submits to Study-Representing Physician.

Monitoring and audit follow the otherwise provided SOP (Appendices I and J). For monitoring and audit, Study-Representing Physician or others provides all the clinical study-related records such as source data, but Study-Representing Physician or others involved in monitoring and audit should keep confidential the medical information possible to identify patients.

15-5. Inspection by CRB or regulatory authority

When CRB or regulatory authority inspects according to Clinical Trial Act, Study-Representing Physician or others provides with all the clinical study-related records such as source data, and should keep confidential the medical information possible to identify patients.

15-6. Control of incompatibility

15-6-1. About incompatibility and serious incompatibility

"Incompatibility" refers to incompliance with regulations, study protocol, and SOP as well as study data tampering and manipulating. "Serious incompatibility" means what influences the human rights and safety of patients, study progression, and reliability of the study results. For example, the incompatibility case is incompliance with the inclusion and exclusion criteria,

study discontinuation criteria, and combination use-prohibited therapies, excluding the cases of deviation from the study protocol for medically inevitable reasons to circumvent emergent risk in patients.

１５-６-２. Duration to collect incompatibility information

From the date of consent obtained until completion of the study period.

１５-６-３. Report of incompatibility

（For detail, refer to Appendix H: SOP at occurrence of incompatibility）

Investigator reports the study-related and confirmed incompatibility to administrator of the participating medical institution, and to Study-Representing Physician, too. The records on incompatibility are appropriately maintained and stored. Reporting methods are as shown below.

[Report type]

1) Routine report
   Investigator or Subinvestigator shares information on incompatibility, if recognized, and promptly fills out EDC, and reports to Study-Representing Physician.

2) Regular report
   According to Article 59, Enforcement Regulations of Clinical Trial Act, Study-Representing Physician periodically reports onset status of the clinical study-related incompatibility with the Enforcement Regulations or the protocol to CRB. Calculating from the day when the protocol was submitted to MHLW, regular report is made yearly within 2 months after every annual term is due. In addition, any incompatibility case to be reported to MHLW should be consulted with MHLW for confirmation every time, according to Article 60, Enforcement Regulations, Clinical Trial Act.

[Procedure for reporting]

1) Report to administrator of the participating medical institution
   Investigator or Subinvestigator reports the recognized case of incompatibility even to administrator of the participating medical institution.
2) Report to IMDC
   Study-Representing Physician reports to the IMDC in writing as needed, and asks to review the measures taken for the incompatibility.
   The IMDC reviews the report, and reports the necessary measures to be taken for

handling of patients or the possibility to continue the study to Study-Representing Physician in writing.

3) Report to CRB

Study-Representing Physician hears opinions from the IDMC as needed, and then, if considered serious, promptly reports to CRB using a designated form. When the case is considered incompatible but not serious, regular report is made using a designated form.

4) Report to MHLW

According to Article 60, Enforcement Regulations of Clinical Trial Act, Study-Representing Physician reports to MHLW through the regular report in writing, every time consulting with MHLW if there is any incompatibility to be reported.

5) Duty of study secretariat

Study secretariat which received any incompatibility report from Investigator or Subinvestigator asks Study-Representing Physician or IDMC, if necessary, to judge the seriousness and effects of the case. In addition, Study secretariat takes measures such as temporal discontinuation of enrollment or announcement to the participating medical institutions, as needed.

6) Announcement to participating medical institutions

After hearing from CRB, Study-Representing Physician summarizes the serious incompatibility case in a predetermined form, lays down the methods to prevent from reoccurrence, and informs all the participating medical institutions.

１５-７. Report to administrator of participating medical institution

Events are summarized below, which are related to quality control and necessary to report to administrator of participating medical institutions, other than the items related to above mentioned “Section 10: Measures to be taken for AE onset” and incompatibility.

１) Investigator reports status of study progression to administrator of the participating medical institution, pursuant to provisions of the institution.

２) Investigator reports the essential items to administrator of the participating medical institution at completion (or discontinuation) of the study.

3) Study-Representing Physician, etc., completes and publishes the report on primary evaluation endpoints, summary report, and their overview without any delay, and reports to administrator of the participating medical institution.

4) Study-Representing Physician, etc., promptly reports to administrator of the participating medical institution about what CRB commented.

１５-８. Regular report to CRB

Study-Representing Physician reports progression status of specified clinical study to CRB. Calculating from the day when the protocol was submitted to MHLW, the report is yearly made within 2 months after every relevant annual term is due. In addition, when regular report to CRB was carried out, Study-Representing Physician promptly provides information for other Investigators to that effect. Those informed Investigators promptly report the information to administrators of the participating medical institutions.

１５-９. Regular report to MHLW

Study-Representing Physician reports study progression status to MHLW within one month after the day when CRB commented.

１５-１０. Management for implementation of double-blind study

Investigator or Subinvestigator keeps the study double-blinded during the study period. For example, interview about the tests (urine sugar tests and urinary ketone body tests) able to distinguish whether the study drug is an active drug or placebo or about change in urine volume should be avoided as much as possible.

１６: Study fund and conflict of interest (COI)

１６-１. Study fund

This clinical study is sponsored by Boehringer Ingelheim Japan, based on the study contract with Niigata University. The study fund is born by Boehringer Ingelheim Japan, based on funding from Boehringer Ingelheim Germany and US Eli Lilly and Company (hereinafter referred to as US Lilly). Although Eli Lilly Japan K.K. is not involved in the fund flow, parent company US Lilly is involved, and cooperates with Boehringer Ingelheim Japan to promote sales of empagliflozin (Refer to Fig. 4).

Boehringer Ingelheim Japan, Boehringer Ingelheim Germany, and US Lilly are involved in funding and planning the study and providing information for proper use of the study drug, but not involved in planning, implementing, analyzing, and publishing this study. When these three companies are asked by Study-Representing Physician for opinions on planning and implementing this study, they can comment only from the scientific and ethical viewpoints based on proper use of this drug and purpose for this study.

[Figure 4] Found flow of EMPA-ICD

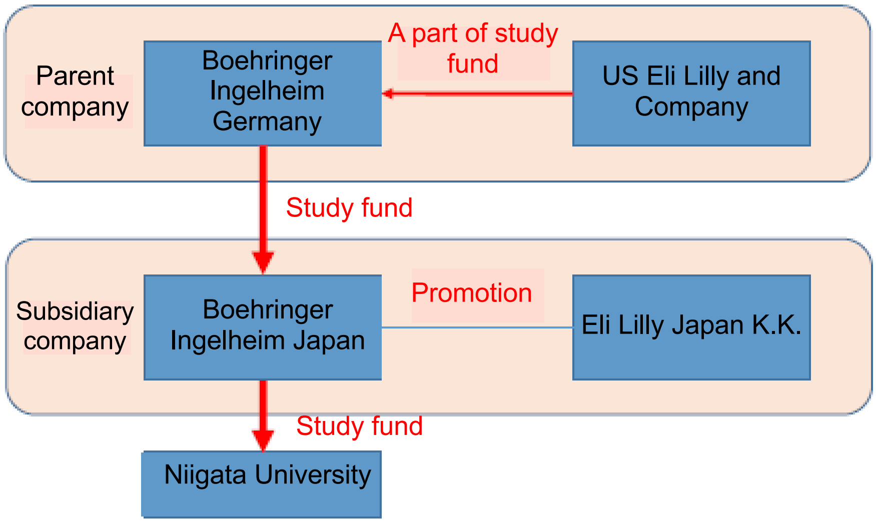

１６-２. Conflict of interest (COI)

Persons engaged in this study appropriately create the COI management criteria according to the Clinical Trial Act, to control the COI management plan. Specifically, the COI management about involvement of manufacturing/marketing business license holder is planned based on fact affirmation at each of the participating medical institutions, and reported to CRB. Comment from the CRB should be responded, if any. The contents of COI are disclosed in response to the requests of academic conferences or medical journals for presentation or publication of the study results.

The COI is disclosed as below according to Clinical Trial Act.

1. Investigator Tetsuji Miura, Department of Cardiovascular, Renal and Metabolic Medicine, Sapporo Medical University Hospital
COI with Nippon Boehringer Ingelheim Co., Ltd: (Himself) Other interest relationship
2. Investigator Yoshio Kobayashi, Department of Cardiology, Chiba University Hospital
COI with Nippon Boehringer Ingelheim Co., Ltd: Personal interest relationship over annual 1 million yen
3. Investigator Toyoaki Murohara, Department of Cardiology, Nagoya University Hospital
COI with Nippon Boehringer Ingelheim Co., Ltd: [1] Total donation of 2 million yen or more annually [2] Personal interest relationship over annual 1 million yen
COI with Eli Lilly Japan K.K.: Personal interest relationship over annual 1 million yen
4. Subinvestigator Yasuya Inden, Department of Cardiology, Nagoya University Hospital
COI with Nippon Boehringer Ingelheim Co., Ltd: Personal interest relationship over annual 1

million yen<br>5. Subinvestigator Wataru Shimizu, Department of Cardiovascular Medicine, Nippon Medical School Hospital<br>COI with Nippon Boehringer Ingelheim Co., Ltd: Personal interest relationship over annual 2.5 million yen

１７: Publication of study results and attribution of right

１７-１. Registration of clinical study

This study is planned to be registered in jRCT (https://jrct.niph.go.jp/), to disclose information. Participating medical institution or study secretariat is involved in registration by enrollment of 1st patient.

１７-２. Publication and attribution of study results

Study-Representing Physician announces the study results upon completion without any delay. The study results are announced after prior review and approval by Study-Representing Physician, Chief Researcher, and Steering Committee. In addition, Study-Representing Physician completes a report on primary evaluation endpoints or summary report and overview by the deadline provided by Clinical Trial Act, and submits to administration of the participating medical institutions. Simultaneously, Study-Representing Physician asks CBR for its comments, and publishes them.

Authors to publish papers and to present at academic conferences are appropriately selected by Study-Representing Physician and Chief Researcher, according to the author requirements (authorship) in International Committee of Medical Journal Editors. All the data obtained in this study are attributed to the study organization.

Boehringer Ingelheim Japan, its affiliated companies, and third parties marketing or promoting the products of Boehringer Ingelheim Japan are allowed to utilize the present study results and reports so as to provide information for medical institutions or to use as reference data in approval application of drugs and medical devices without any charge in the world.

Presentation and attribution of the metabolome analysis results are based on the collaborative study contract by Department of Cardiovascular Biology and Medicine, Niigata University Graduate School of Medical and Dental Sciences with Institute for Advanced Biosciences, Keio University.

Disclosure of exploratory analysis results obtained in this study is likely to be less beneficial for patients, and concerned even to induce misunderstanding or uneasiness. Thus, the individual analysis results are not disclosed at present. However, if information likely to

seriously influence life or the results that it can be considered medically beneficial to disclose were obtained during the study processes, they may be disclosed.

１８: Revision of study protocol

If considered necessary, Study-Representing Physician consults with Steering Committee to decide revision of the study protocol as needed. The decision is reviewed at CRB and communicated to Investigator. Then, administrator of the participating medical institution approves the study based on the revised protocol. If considered necessary, patient information is promptly revised in association with revision of the study protocol, reviewed according to the same procedure, and approved by administrator of the participating medical institution. If any onset of the event possibly affecting patient's will to continue to participate in the study leads to revision of the informed consent form, according to the procedures of the protocol 6-2, using the approved updated informed consent form, written consent to continue to participate in the study should be obtained from patients again.

１９: Completion, discontinuation, or suspension of study

１９-１. Completion of study

When the study is completed at each of the participating medical institutions, Investigator reports to administrator of the participating medical institution in writing.

１９-２. Discontinuation or suspension of study

When the study drug is able to be definitely judged as effective or ineffective in the context of study objectives and contents at the time when No. of patients are not enough enrolled as planned, or judged as inappropriate to continue the study because of serious information on the study-related safety, the IMDC recommends Study-Representing Physician to discontinue or suspend the study. When decided to discontinue or suspend, Study-Representing Physician promptly communicates the reasons for discontinuation and measures taken for patients to the participating medical institution in writing.

When the study was discontinued or suspended according to judgment or condition of the participating medical institution, Investigator promptly reports to Study-Representing Physician.

２０: About Certified Review Board (CRB)

CRB at which this study is asked to be reviewed is as follows.

Name of CRB: Niigata University Central Clinical Study Review Committee
Location of CRB: 1-754 Asahimachi-dori, Chuo-ku, Niigata
Telephone: 025-368-9343
Facsimile: 025-227-0720
e-Mail address: crbcr@adm.niigata-u.ac.jp

２１: Study system

See Appendix K.

２２: References

1) Eur Heart J. 2008; 29: 1377-85.
2) Circulation. 2013; 128: 694-701.
3) Guidelines for diagnosis and treatment of cardiovascular diseases (2009 Joint Working Group Report).
Guidelines for Treatment of Chronic Heart Failure (2010 revised version).
4) Guidelines for diagnosis and treatment of cardiovascular diseases (2009 Joint Working Group Report).
Guidelines for Risks and Prevention of Sudden Cardiac Death (2010 revised version).
5) N Engl J Med. 2015 Nov 26;373（22）:2117-28.
6) Diabetes 2016;65:1190–1195.
7) Trends Endocrinol. Metab. 2014; 25: 42–52.
8) Treatment Guide for Diabetes 2016-2017, ed. The Japan Diabetes Society.
9) Clin Ther. 2014;36:1606-15.
10) Lancet Diabetes Endocrinol. 2014;2:369-84.
11) Diabetes 2014;63:1738–1747.

Patient Information Sheet

Comparison of empagliflozin or placebo for prevention of lethal ventricular arrhythmia in type 2 diabetic patients with implantable cardioverter defibrillators (ICD)

EMPA-ICD

We are going to explain the content of this clinical study to you.

This document aims to give you information in addition to our explanation and to deepen your understanding of the study. Please read this document thoroughly before you decide whether you would like to participate in the study.

You are free to decide whether to participate in the study. Even if you start participating in the study, you may stop participating at any time. Your refusal to participate will never affect your future treatment.

Before you can decide whether to participate in the study, you have to know as much as possible about the content of the study. If you have any questions or there are any words you don't understand, please feel free to contact us.

EMPA-ICD Study Group

Prepared on: May 20, 2022 (ver. 2.5)

Introduction

Our hospital verifies the usefulness of treatments and constantly seeks new ones to provide the treatment that is most suitable for various diseases in patients.

Heart disease and diabetes are treated with therapies that are supported by data from research performed in clinical settings. In clinical settings, such research is called clinical research. Medical activities associated with clinical research may be conducted only after obtaining patients' consent.

Unlike usual treatment, clinical research that aims to study a new treatment is partly investigative. There is a rule that, before clinical research is conducted, it must be reviewed and approved to ensure that it will be conducted in a way that is safe for participants and that involves sufficient ethical considerations for patients. A Certified Review Board, which reviews and gives opinions on clinical research in compliance with the Clinical Trials Act, reviewed this study titled "Comparison of empagliflozin or placebo for prevention of lethal ventricular arrhythmia in type 2 diabetic patients with implantable cardioverter defibrillators (ICD)" and confirmed that the content of the study is appropriate and that patient rights are protected. Moreover, the procedures necessary for conducting clinical research, including notifications to the Ministry of Health, Labour and Welfare and registration in the Japan Registry of Clinical Trials (hereafter called "jRCT"), have been appropriately performed.

Certified Review Board of this study

**Name of Certified Review Board:**	**Niigata University Central Review Board of Clinical Research**
**Location of the Certified Review Board:**	**754, Ichibancho, Asahimachidori, Chuo-ku, Niigata-shi, Niigata, Japan**
**Phone no. of the department in charge:**	**025-368-9343**
**Fax no. of the department in charge:**	**025-227-0720**
**E-mail address of the department in charge:**	**crbcr@adm.niigata-u.ac.jp**

You can visit the following website to see the content of the reviews and the Certified Review Board: https://www.crbcr.niigata-u.ac.jp/

This clinical study was planned to determine whether the drugs used to treat diabetes also improve arrhythmia in patients with diabetes who have an implanted arrhythmia device. Please read through this patient information sheet so that you fully understand the study before you voluntarily decide whether to participate in it. If you decide to participate, please sign a separate consent form. Please feel free to ask your study doctor about any difficult words or phrases in the patient information sheet.

1. Purpose of the Study

Patients with diabetes and an implantable cardioverter-defibrillator (ICD) or implantable cardioverter-defibrillator with biventricular pacing function (CRT-D) (both of which are referred to in the text below as an "arrhythmia device") are known to have arrhythmia more often than patients with an arrhythmia device who do not have diabetes. Blood glucose control appears to be very important in patients with diabetes and an arrhythmia device, but the guidelines of the Japanese Circulation Society do not specifically recommend a blood glucose target level or diabetes drugs. Currently, doctors themselves decide on target blood glucose levels during treatment and on dosing regimens.

In July 2014, sodium-glucose co-transporter 2 (SGLT2) inhibitors (drugs that inhibit the transport of glucose and sodium) were approved as a new treatment to reduce blood glucose levels by causing glucose to be excreted in the urine. SGLT2 inhibitors have various effects, including improving metabolism and autonomic nerve functioning, in addition to reducing blood glucose. These effects are expected to have a favorable impact in patients with cardiovascular diseases. This was found for the SGLT2 inhibitor empagliflozin, which in 2015 was reported to improve the prognosis of patients with diabetes who are at high risk of cardiovascular complications; in this case, “prognosis” refers to the onset or worsening of heart disease, hospitalization because of heart disease, or death resulting from heart disease. Moreover, in 2017, canagliflozin, another SGLT2 inhibitor, was reported also to improve prognosis. The mechanism by which SGLT2 inhibitors improve prognosis related to heart disease has not been fully clarified, but researchers think the positive effect of SGLT2 inhibitors on autonomic nerves may have a favorable impact on arrhythmia associated with heart disease.

Therefore, we planned this study to determine the effect of the SGLT2 inhibitor empagliflozin on the number of arrhythmias automatically recorded by an implanted arrhythmia device. The study will be performed in patients with diabetes who have an implanted arrhythmia device. Patients will be treated with empagliflozin or placebo (see next section), and the two groups will be compared.

What is placebo?

A placebo is a study drug that looks the same as the investigational drug, in this case the SGLT2 inhibitor, but does not contain any active ingredient. Even though the placebo does not contain any active ingredient, in some people it may have an effect (the so-called placebo effect) because they are aware that they are “taking medicine.” Therefore, the efficacy and safety of the investigational drug are properly evaluated by comparing it with the placebo. Such a method that uses a placebo and in which you and your study doctor are not told which study drug you are taking is called the double-blind method.

2. Summary of the Study

2.1. Patients who can participate

Patients who meet all of the following conditions can participate:

1) Patients aged 20 years or older (men and women)
2) Patients who underwent ICD or CRT-D implantation surgery more than 24 weeks before the study (regardless of the reason for the implantation)
3) Patients diagnosed with type 2 diabetes who can receive the study drugs (regardless of whether they have received previous treatment)
4) Patients who can provide written consent to participate in the study

2.2. Patients who cannot participate

Patients who meet any of the following conditions cannot participate in the study:

1) Patients who are taking an SGLT2 inhibitor
2) Patients who started treatment with an SGLT2 inhibitor within the past 24 weeks and then discontinued it
3) Patients with a past history of hypersensitivity to empagliflozin (Jardiance®)
4) Patients who experienced complications caused by abnormal blood glucose (diabetic ketoacidosis, diabetic coma, or a hypoglycemic attack requiring intervention by a third person) within the past 24 weeks
5) Patients with a severe infection or injury and those who will undergo surgery (excluding surgical operations to replace the battery cell in the arrhythmia device)
6) Patients with type 1 diabetes
7) Patients with severe cardiac failure rated as New York Heart Association (NYHA) Class IV (please see note below)
8) Patients with severe renal impairment (i.e., patients with an estimated glomerular filtration rate [eGFR] of less than 30 mL/min/1.73 m^2 or who are receiving dialysis therapy)
9) Patients with a serious hepatic function disorder (aspartate transaminase [AST] or alanine transaminase [ALT] more than 3 times higher than the normal local laboratory value)
10) Patients with pituitary or adrenal insufficiency
11) Malnourished patients (patients who are fasting, eat irregularly, have a poor diet or are debilitated)
12) Patients with a history of excessive alcohol intake
13) Patients who have a gastrointestinal disorder like diarrhea or vomiting and are likely to be dehydrated
14) Patients with a urinary tract or genital infection
15) Patients who are pregnant, of childbearing potential or breastfeeding or who want to

become pregnant while participating in this study

16) Patients with a low weight (BMI less than 18.5 kg/m^2)

17) Patients with any of the following events within the past 24 weeks: change of antiarrhythmic drug, catheter ablation for ventricular arrhythmia, coronary revascularization, open heart surgery, development of coronary artery disease, stroke or transient ischemic attack, infection requiring hospitalization, or cardiac failure requiring hospitalization

18) Patients using an arrhythmia device that is unable to record some non-sustained ventricular tachycardia episodes

19) Patients with non-remitted malignant tumor

20) Patients whom the study doctor judges as inappropriate to participate in the study

Note: The NYHA Functional Classification is used to classify symptoms into four classes, Class I to IV, as follows:

Class I	No limitation of physical activity. Ordinary physical activity does not cause undue fatigue, palpitation, dyspnea (shortness of breath), or anginal pain.
Class II	Slight limitation of physical activity. Comfortable at rest. Ordinary physical activity results in fatigue, palpitation, dyspnea (shortness of breath), or anginal pain.
Class III	Marked limitation of physical activity. Comfortable at rest. Less than ordinary activity causes fatigue, palpitation, dyspnea (shortness of breath), or anginal pain.
Class IV	Unable to carry on any physical activity without discomfort. Symptoms of heart failure and/or anginal pain may be present even at rest. If any physical activity is undertaken, discomfort increases.

2.3. Discontinuation of the Study

The study may be discontinued in any of the following cases. If it is discontinued, tests, including blood tests and echocardiography, will be performed for evaluation wherever possible.
Please see "Test Items and Schedule" on page 8 for the detailed testing schedule.

1) When it is determined to be difficult to continue the study because the underlying disease or complication is aggravated

2) When it is determined to be difficult to continue the study because of the onset of an adverse event

3) When it is determined to be difficult to continue the study because the patient moved

4) When it is determined to be difficult to continue the study because the patient was transferred to another hospital
5) When the patient asks to discontinue participation in the study or withdraws consent
6) When the patient takes an SGLT2 inhibitor after taking any study drug
7) When the patient experiences any of the following events, which are likely to influence the onset of severe arrhythmia, after taking any study drug: change of antiarrhythmic drug, catheter ablation for ventricular arrhythmia, coronary revascularization, open heart surgery, development of coronary artery disease, stroke or transient ischemic attack, infection requiring hospitalization, or cardiac failure requiring hospitalization
8) When the study doctor determines that it is difficult to continue the study for any other reason

2.4. Methods

1) Study procedures

In this study, patients will take study drugs in addition to their usual treatment. Patients who consented to participate will be randomly assigned in a 1:1 ratio to receive empagliflozin, an SGLT2 inhibitor (empagliflozin group), or placebo (placebo group). The placebo looks the same as the SGLT2 inhibitor, but the placebo does not contain the active ingredient (i.e., it does not contain empagliflozin). A computer will determine the allocation to each group, so neither the patient nor the study doctor can choose the group, and they will not know the result of the allocation until the follow-up period is completed and everything has been prepared for the data analysis. This is a very precise study method called the double-blind method and it makes it possible to properly evaluate the efficacy and safety of the SGLT2 inhibitor in comparison with the placebo (see “What is placebo?” under 1. Purpose of the Study). After patients have given informed consent and the initial eligibility tests have been performed, treatment will start according to the result of the computer-based allocation and will continue for 24 weeks.

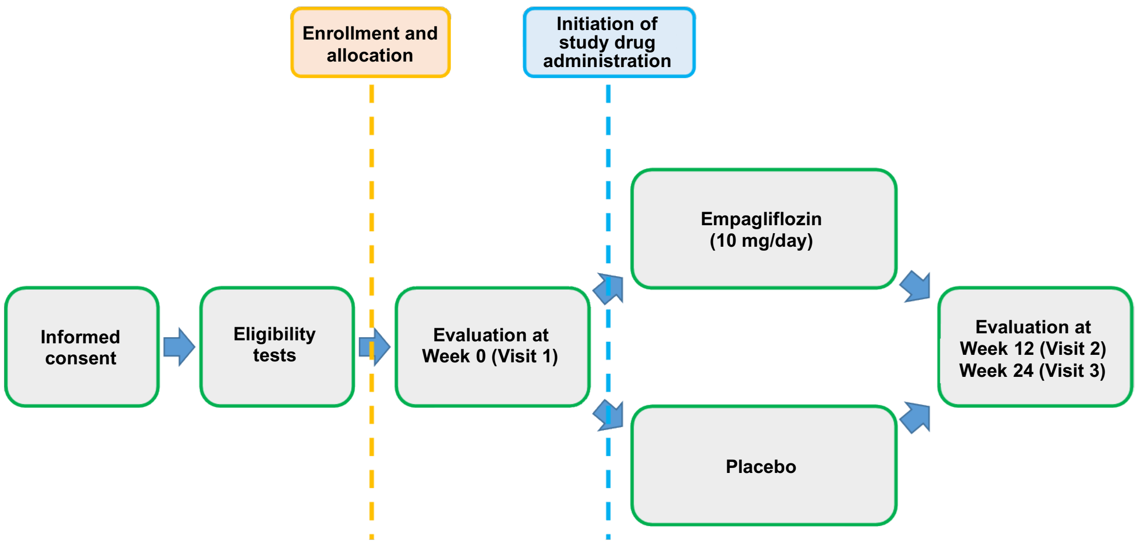

If you are treated at another hospital, please let us know the name of the hospital, the disease you are treated for there, and the drugs you are using. In addition, if you use any drugs that you have bought at pharmacies or other stores, please tell us about them. This is important so that we can conduct the study safely. If you are treated at another hospital, please note that we will notify the hospital of your participation in the clinical study by sending them the letter “Notification of your patient’s participation in a clinical study and what we would like you to do.”

2) Drugs to be used in the study

The drugs to be used in the study (free of charge) will be prescribed separately from other drugs. Study drugs may be supplied to you all at once at Week 0 (Visit 1) or in installments at subsequent visits. Your study doctor will carefully determine your availability for study visits and your symptoms, consider the best choice to ensure your safety and be responsible for taking appropriate action.

In this study, you will take one tablet of your study drug (empagliflozin 10 mg or placebo) once a day before or after breakfast. As mentioned above, both you and your study doctor will not know which group you are allocated to, so please take your study drug for 24 weeks and follow the instructions of your study doctor. Predetermined tests will be performed at the start of treatment and at Week 24 (Visit 3) or the time of study discontinuation.

At Week 24 (Visit 3) or the time of study discontinuation, please return all of the remaining study drugs and empty bottles to us or your study doctor.

3) Test Items and Schedule

[Test Schedule]

Enrollment and allocation (between Screening and Visit 1) / Initiation of study drug administration (between Visit 1 and Visit 2)

	Before treatment		Treatment period		
	Screening	Visit 1	Visit 2	Visit 3	At time of discontinuation
	Week -12 to 0	Week 0	Week 12	Week 24	
		–	(± 4 weeks)	(± 4 weeks)	
Patient information/ informed consent	○				
Patient background	○				
Interview/physical examination	○	○	○	○	△
Confirmation of drug compliance			○	○	△
Confirmation of compliance with study procedures		○	○	○	△
Confirmation of other drugs being used	○	○	○	○	△
Height	○				△
Body weight/body temperature	○	○	△	○	△
Blood pressure/ pulse rate		○	△	○	△
Hematological tests	○	○	△	○	△
Hematological tests (blood glucose/lipids)	○	○	△	○	△
Hematological tests (special)		○		○	△
12-lead ECG		○		○	△
Holter monitoring		○		○	△
Echocardiography	○	○		○	△
^{123}I-MIBG myocardial scintigraphy		△		△	△
Adverse events	○	○	○	○	△
Evaluation of arrhythmia device		○		○	△

○, Essential; △, Optional.

Optional items (△) may or may not be performed. Please contact your study doctor for further information. Wherever possible, fasting blood will be sampled after you have rested lying face up on an examination table for 30 minutes.

1. Patients who visit the hospital in the morning will undergo blood sampling without eating after dinner on the day before the test (no breakfast on the test day).
2. Patients who visit the hospital in the afternoon will undergo blood sampling without eating after breakfast on the test day (no lunch on the test day).
3. After informed consent and eligibility screening, at Week 0 (Visit 1) pre-treatment tests will be performed and then your study drug will be prescribed.
4. If any tests (hematological tests, 12-lead ECG, Holter monitoring, echocardiography, or ^{123}I-MIBG myocardial scintigraphy) are performed within 3 months before the tests at Week 0 (Visit 1), the data from those tests will be used.

5. Although no tests are scheduled at Week 12 (Visit 2), if you are not doing well, please consult with us. In addition, please bring empty bottles after taking medicine.
6. Repeat tests are scheduled at Week 24 (Visit 3) or at the time of study discontinuation.
   Please bring all of remaining drugs and empty bottles with you at your visits.
7. <u>You will wear a Holter monitor for 24 hours and will need to visit the hospital on the following day to return it.</u>

[Test Items]

Background information

You will be interviewed about the following information: gender, age, smoking history, alcohol-drinking history, arrhythmia that caused implantation with arrhythmia device and underlying diseases, make and type of arrhythmia treatment device, comorbidity and past history, drugs you are taking (called “concomitant medication”), and past history of non-drug treatment.

Physical examination

You will be asked about your subjective symptoms (for the NYHA classification).

Confirmation of drug compliance and concomitant medication

You will be asked whether you forgot to take any study drugs and whether there was any change in your concomitant medication.

Confirmation of compliance with the procedures in the protocol

Your study doctor will check whether the tests and treatment adhere to the study protocol.

Height, body weight, and body temperature

Height will be measured only during eligibility screening, but body weight and temperature will be measured at every visit.

Blood pressure and pulse rate

As a general rule, these will be measured in a sitting position after you have rested for more than 5 minutes.

Hematological tests (blood sugar and lipids): About 17 mL of your blood will be collected for the tests.

The following items will be measured, all of which are general items that tell us about your condition:

Hematology (red blood cell count, white blood cell count, hemoglobin, hematocrit, and platelet count), blood biochemistry (total protein, albumin, AST, ALT, alkaline phosphatase, total bilirubin, lactate dehydrogenase, blood urea nitrogen, sodium, potassium, chloride, uric acid, creatinine, eGFR), and brain natriuretic peptide.

Blood glucose and lipid tests: Fasting blood glucose and hemoglobin A1c, triglyceride, total cholesterol, high-density lipoprotein cholesterol, and low-density lipoprotein cholesterol

Note: AST, ALT, and eGFR are essential for eligibility screening.

Special blood tests (central measurement): About 14 to 34 mL of your blood will be collected for the

tests. Please see "Test Items and Schedule" on page 8 for the detailed blood test schedule.

The following items will be measured in the study tests (you will not be charged for the costs of the tests that are not covered by medical insurance). Essential tests: blood ketone body fraction, blood catecholamine concentration, erythropoietin, and reticulocytes. Optional tests: DNA and RNA tests and metabolome analysis.

The tests will be performed at central measurement institutions (SRL, Inc., Niigata University, Institute for Advanced Biosciences of Keio University, and Graduate School of Medicine of Juntendo University), as follows:

Blood ketone body fraction (acetoacetic acid, 3-hydroxybutyric acid, and total ketone bodies), blood catecholamine concentration (adrenalin, noradrenalin, and dopamine), erythropoietin, and reticulocytes: SRL, Inc.

DNA tests (telomere length) and RNA tests (P53, P21, and P16): Graduate School of Medicine of Juntendo University. The DNA and RNA tests will not analyze genetic data containing personal information.

Metabolome analysis: Institute for Advanced Biosciences, Keio University.

12-lead ECG tests

The following items will be measured by electrocardiography:

Heart rate, rhythm, PQ interval, QRS interval, and QT interval

Holter monitoring

Holter monitoring will measure the following items, which are detailed measures that are unlikely to be detected by an arrhythmia device:

Total recoding time, total number of ventricular premature contractions (VPCs), number of single VPCs, number of occurrences of two consecutive VPCs, and number of ventricular tachycardia

Echocardiographic tests

Echocardiography will measure the following items, which are general items to evaluate cardiac function:

left ventricular ejection fraction, E wave, A wave, E/A, early diastolic velocity at the septal mitral annulus, early diastolic velocity at the lateral mitral annulus, left ventricular diastolic function, tricuspid regurgitation peak velocity, and left atrial volume index

^{123}I-MIBG myocardial scintigraphy tests

The following items will be measured by ^{123}I-MIBG myocardial scintigraphy to determine the status of the autonomic nervous system (^{123}I-MIBG myocardial scintigraphy is widely used in daily clinical practice to estimate the severity or prognosis of heart disease; however, it takes about 3 to 6 hours, so the tests at both Week 0 and Week 24 or the time of study discontinuation are optional):

H/M (heart-to-mediastinum) ratio and washout rate

Safety evaluation

Adverse events: Onset of any unfavorable or unintended signs, symptoms, or diseases will be determined.

Evaluation of arrhythmia device

The data recorded in the device, including the number of ventricular and atrial arrhythmias and biological monitoring indicators, will be output and used.

If data are available from the 24 weeks before Week 0 or the time of evaluation after informed consent, these data will be used as the data at Week 0 (Visit 1). The data from the 24 weeks after the start of study treatment will be used as the data at Week 24 (Visit 3).

Note: The information on patient health and any genetic elements that may possibly be transmitted to children is not included in the study endpoints.

Please feel free to ask your study doctor about any unknown words or phrases.

2.5. Scale of the study

This study will be conducted at university hospitals and general hospitals throughout Japan.

The target sample size is 210 participants (105 per group).

Expected study period: Until October 2023 (expected enrollment period: until April 2021)

If you participate in the study, the expected participation period is about 24 weeks.

(Please see the table in 2.4. Methods (1).)

3. How the samples and data will be used and how long they will be stored

The data to be collected in the study will be appropriately managed at sites and data centers so that personal information is protected. Investigators, who have specific access rights, may access the collected data stored at data centers.

The data to be obtained and managed in the study will not contain information that identifies individuals, such as patient name and birth date. For data management, the data of each patient will be anonymized by coding them independently of the study and will then be securely stored and analyzed.

After measurements have been made, samples collected at each site will be destroyed appropriately according to local rules. Samples collected for special blood tests will be anonymized with the study enrollment number and collected and measured in central measurement institutions (SRL, Inc., Niigata University, Keio University, and Juntendo University).

In the DNA tests (telomere length) and RNA tests (P53, P21, P16) to be performed in the study, no analysis of genetic data containing personal information will be performed. Remaining samples will be appropriately stored with unidentifiable personal information, as specified by Niigata University School of Medicine.

Remaining samples after completion of measurements will be stored at the laboratory of Cardiovascular Medicine, Niigata University School of Medicine, and will be additionally analyzed after their availability is confirmed, in accordance with predetermined procedures, if new information is obtained with respect to the study and the representative investigator determines that additional analysis is required. Because analysis will be performed with unidentifiable personal information, your personal information will also not be divulged in case of additional analysis.

Samples and data will be stored for 5 years after discontinuation or completion of the study and will then be destroyed; individuals will be unidentifiable.

4. Expected advantages and disadvantages of participation in the study

If you participate in the study, your condition will be regularly examined. Blood tests, electrocardiography, Holter monitoring, echocardiography, and ^{123}I-MIBG myocardial scintigraphy would enable your health condition to be accurately understood and help future treatment. The special blood tests, which cover unknown areas, are unlikely to help future treatment. The results of the study are likely to help treatment of other patients with your disease in the future. However, during the study, you will have to restrict your diet and visit the hospital for tests, which will have an impact on your life and take time. In addition, you will be charged the costs for some of the tests (see 12. Study-related costs for patients).

Moreover, if you are allocated to receive the placebo (which has the same appearance as the SGLT2 inhibitor but does not contain any active ingredient), no effect on diabetes is expected. However, you will be receiving treatment with other diabetes medications, as described below, which can minimize this disadvantage of receiving placebo.

If new information is obtained during the study that may affect the continuation of patient participation in the study (such as information on a new serious adverse reaction), updated information will be provided to patients as needed. At that time, we will ask you if you want to continue your participation in the study. At such a time, we ask that you please decide voluntarily whether you want to continue.

5. Expected effects and most frequently observed adverse reactions

Because the drug to be used in the study (empagliflozin) has the effect of reducing blood glucose, your blood glucose levels are expected to decrease. However, almost all current treatments not only have effects but also cause adverse reactions. Some adverse reactions rarely lead to severe symptoms.

[Expected adverse reactions to empagliflozin]

[1] Clinically significant adverse reactions and frequencies

Hypoglycemia (2.3%), dehydration (0.1%), ketoacidosis (frequency unknown), pyelonephritis

(frequency unknown), sepsis (frequency unknown), necrotizing fasciitis of the perineum (Fournier's gangrene; frequency unknown)

[2] Other adverse reactions (0.1% to less than 5%)

Constipation, abdominal distention, thirst, hunger, cystitis, urinary tract infection, vulvovaginal candidiasis, asymptomatic bacteriuria, balanitis, genital pruritus, pollakiuria, polyuria, urine output increased, rash, hyperlipidemia, hemoconcentration, dizziness, dysgeusia, weight decreased, presence of urine ketone bodies

You may experience other adverse reactions. Use of the study drug in combination with other drugs (especially sulfonylurea or insulin preparations) may increase the risk of adverse reactions, including hypoglycemia. If you get any other disease or feel different from usual while participating in the study, please immediately contact your study doctor, who will change your treatment or treat your symptoms appropriately.
Please see “11. What we would like you to do” for further information.

In this study, a larger amount of blood than usual (17 to 51 mL per visit more than usual) will be collected because some components will be examined for the study in addition to the blood tests that are performed in your usual care. In ^{123}I-MIBG myocardial scintigraphy, you will be exposed to a little radiation, which appears to hardly cause any injury to health (some special blood tests and ^{123}I-MIBG myocardial scintigraphy are optional).
You may participate in additional tests for this study, which may take up more of your time.

6. Other treatments

Besides empagliflozin, other diabetes medications are available, including other SGLT2 inhibitors, sulfonylurea drugs, dipeptidyl peptidase 4 inhibitors, rapid-acting insulin secretagogue, α-glucosidase inhibitors, biguanides, thiazolidine drugs, insulin preparations and glucagon-like peptide-1 analogues. Glycemic control is possible with these drugs, which have different mechanisms of action and pros and cons. In addition, glucose levels can be controlled by non-drug methods, such as diet and exercise therapies. Even if you do not participate in this study, you will continue to receive appropriate treatment, including medical treatment. Please fully consult with your study doctor to select such treatments.

7. If any additional treatment is required

If your study doctor determines that your glycemic control is poor, you will receive additional treatment, which may include increasing the dose of the diabetes medication you started before participating in the study or prescribing a diabetes medication other than the SGLT2 inhibitor. Please

follow the instructions of your study doctor.

8. Treatment after completion of the study

After completion of the study, you will receive treatment depending on your condition and wishes.

Please make sure to return remaining study drugs and empty bottles to your study doctor at the time of the Week 24 tests or the time of study discontinuation.

9. Your decision to participate in the study is voluntary

Your decision to participate is voluntary. Even if you refuse to participate, you will not be at a disadvantage because you will still receive the best treatment for your condition.

If you decide to participate, you may consult with us, including your study doctor, and may withdraw from the study at any time, in which case you will also receive the best treatment for your condition and never be disadvantaged.

10. Protection of your human rights

Whenever we report on your disease, symptoms, physical conditions, and tests at scientific meetings or academic conferences, to protect your privacy your personal information, including your name, birth date, etc., will never be used in such reports.

By signing the consent form, you tell us that you agree to the above.

11. What we would like you to do

During the study, please follow our instructions, including those of your study doctor. In particular, wherever possible please follow test procedures, including those regarding meals and resting. If any abnormalities occur, please immediately contact us or your study doctor (see 17. Contacts for the Study).

The appendix "Major adverse reactions to empagliflozin" describes details of possible major adverse reactions to the investigational drug. If you experience any such adverse reactions, please contact us using the contacts as described in "17. Contacts for the Study" below.

Please return all of the remaining drugs and empty bottles at Week 24 (Visit 3) or the time of discontinuation to us or your study doctor. We will destroy them appropriately.

If you are injured in this study, despite following our instructions and those of your study doctor, we will be responsible for treating you appropriately.

If you visit another hospital, please let us know the name of the hospital, the disease you are treated for there, and **the drugs you are using. In addition, if you use any drugs that you have**

bought at pharmacies or other stores, please tell us about them.

If you go to another hospital or clinic, please make sure to notify the doctor at the hospital or clinic of your participation in the study.

12. Study-related costs for patients

The drugs to be used in the study (free of charge) will be prescribed separately from other drugs, and the costs of the investigational drug administered during the study and those of special blood tests not covered by insurance will be covered by study expenses. The costs of usual care will be covered by your public medical insurance, that means your insurance will pay the costs of general blood tests, electrocardiography, Holter monitoring, echocardiography, and ^{123}I-MIBG myocardial scintigraphy because these tests will be performed as part of your usual care. Such tests are part of the usual care of patients with heart disease and those with diabetes.

Participation in the study will cause some burden such as an increase in the amount of blood collected and the time required for additional testing. Therefore, to compensate you for your participation we will give you a QUO card worth ¥20,000 at both Week 0 and at the time of completion of the study (Week 24 or discontinuation for any reason).

13. Compensation for study-related injury

The study was planned scientifically by referring to previous studies, and it will be conducted carefully. However, if you experience any injury, such as an adverse reaction, during or after the clinical study, doctors will appropriately examine and treat you. Although you will be asked to pay for the costs of such treatment beyond what the public medical insurance covers for your usual care, you will be reimbursed for any costs associated with any study-related injury by clinical study insurance.

Because this study is not a clinical trial for the development of a new drug by a pharmaceutical company, participants with a study-related injury will not receive special compensation from the company organizing the trial. However, the representative study investigator has clinical study insurance to cover any injury related to the clinical study. Please note that patients will not receive any compensation if the investigational drug has no effect or if patients receive no therapeutic benefit because they received the placebo.

Please read through the “Summary of Compensation System for the Study” for more information on the compensation we will give to you in case of injury.

If you have any questions, please contact your study doctor (see 17. Contacts for the Study).

14. Possible Conflicts of Interest

The study group performing this study includes cardiovascular and diabetes specialists, and the representative investigator is Tohru Minamino, Department of Cardiovascular Medicine, Niigata

University Graduate School of Medical and Dental Sciences. During the study, the committee, which consists of independent specialists who are not participating in the conduct of the study, will monitor the status of conflicts of interest. Before the start of the study, the representative investigator will set a standard for conflicts of interest and manage conflicts of interest in all persons involved in the study. The conflicts of interest management plan was reviewed by the Certified Review Board.

The drugs to be used in the study will be provided by Nippon Boehringer Ingelheim Co., Ltd. The study is funded under the study agreement executed between the company and Niigata University. Such funding will be provided to Nippon Boehringer Ingelheim Co., Ltd, by Boehringer Ingelheim Germany and Eli Lilly and Company (US). Eli Lilly and Company (US), a parent company of Eli Lilly Japan K.K., and not Eli Lilly Japan K.K. is involved in the flow of the study funds and is cooperating with Nippon Boehringer Ingelheim Co., Ltd, to promote empagliflozin. Please refer to the below figure for information on the flow of funds.

Employees of the respective company are not involved in either the conduct or analysis of the study or in decision-making that may affect the results of the study.

[Flow of study funds]

Flow of Funds for EMPA-ICD Study

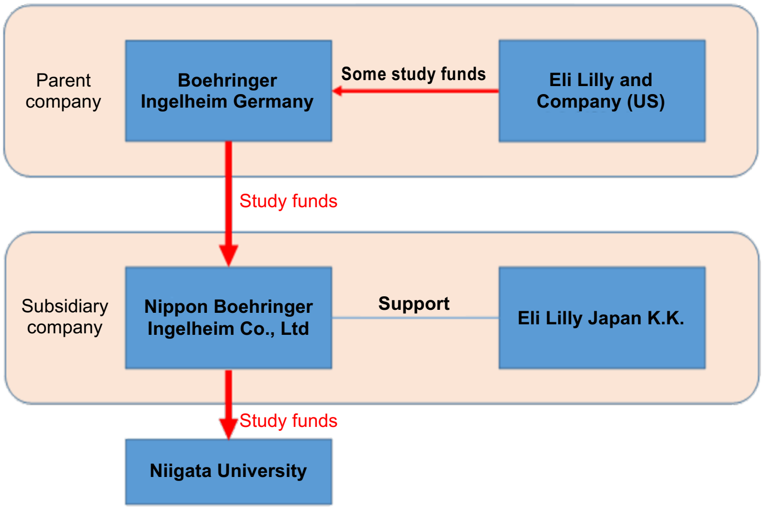

<Announcement of Conflicts of Interest>

Under the Clinical Trials Act, we report the following conflicts of interest (COI):

1. Principal investigator: Tetsuji Miura, Department of Cardiovascular, Renal and Metabolic Medicine, Sapporo Medical University Hospital
   COI with Nippon Boehringer Ingelheim Co., Ltd: Other interest relationship
2. Principal investigator: Yoshio Kobayashi, Cardiovascular Medicine, Chiba University Hospital
   COI with Nippon Boehringer Ingelheim Co., Ltd: Personal interest relationship of more than ¥1

million annually

3. Principal investigator: Toyoaki Murohara, Cardiovascular Medicine, Nagoya University Hospital
   COI with Nippon Boehringer Ingelheim Co., Ltd: 1) Total donation of ¥2 million or more annually, 2) personal interest relationship of over ¥1 million annually
   COI with Eli Lilly Japan K.K.: Personal interest relationship of over ¥1 million annually
4. Subinvestigator: Yasuya Inden, Cardiovascular Medicine, Nagoya University Hospital
   COI with Nippon Boehringer Ingelheim Co., Ltd: Personal interest relationship of over ¥1 million annually
5. Subinvestigator: Wataru Shimizu, Cardiovascular Medicine, Nippon Medical School Hospital
   COI with Nippon Boehringer Ingelheim Co., Ltd: Personal interest relationship of over ¥2.5 million annually

15. Monitoring and auditing

To determine whether the study is conducted properly and safely, patient human rights are protected, and the results of treatments and tests are accurately reported, third parties may visit study sites, including our hospital, to evaluate study documents by monitoring and auditing them. During monitoring and auditing, the following third parties may perform source data verification of your medical records and test results: the contract research organization (CRO) Micron, Inc., healthcare professionals of medical institutions, and data managers.

The monitoring and auditing will be performed only by the third parties for whom the permission to access medical records has been reviewed by the Certified Review Board and who are permitted to access medical records by the director of the hospital. Under laws and regulations, the third parties are liable for maintaining the confidentiality of personal information. Therefore, the personal information they obtain will never be divulged. By signing the consent form, you tell us that you approve the monitoring and auditing of the study.

Besides monitoring and auditing, the Certified Review Board and regulatory authorities (Ministry of Health, Labour and Welfare) may investigate the study, which may possibly include accessing personal information; however, the information will never be divulged. Approval of the investigation by the Certified Review Board or regulatory authorities will be obtained by using the consent form.

Monitoring and auditing organization: Micron, Inc.

16. Publication of the information on the study

Because the summary of the study will be registered in a publication database (the system for publication of the summary of protocols and clinical research, jRCT), you may find the content, status, results, etc. of the study on the internet. Your personal information will never be disclosed, but you may see the protocol that describes the study. If you want more information, please contact your

study doctor.

jRCT website:
https://jrct.niph.go.jp/

17. Contacts for the Study

If you have any questions about the study or you suffer any injury, please feel free to contact us or your study doctor.

Representative Investigator

Tohru Minamino, Part-time Lecturer
Department of Cardiovascular Medicine, Niigata University Graduate School of Medical and Dental Sciences

<Contact for the study>
EMPA-ICD Research Office
Department of Cardiovascular Medicine, Niigata University Graduate School of Medical and Dental Sciences
Asahimachi-Dori 1-757, Chuo-ku, Niigata, Niigata 951-8510, Japan
Tel: 025-227-2185, FAX: 025-227-0774

Investigator and contact at each study site (in order of the Japanese syllabary)

■ Tachikawa General Hospital, Tachikawa Medical Center
Principal investigator: Masaaki Okabe (Director of the Hospital), Cardiovascular Medicine
Address: Asahioka 1-24, Nagaoka-shi, Niigata, 940-8621
Tel: 0258-33-3111, Fax: 0258-33-8811

■ Iwate Medical University Hospital
Principal investigator: Shingen Owada, Lecturer, Cardiovascular Medicine
Address: 2-1-1 Idaidori, Yahaba-cho, Shiwa-gun, Iwate 028-3695
Tel: 019-613-7111, Fax: 019-907-7279

■ Kanazawa University Hospital
Principal investigator: Takeshi Kato, Assistant Professor, Cardiovascular Medicine
Address: 13-1, Takaramachi, Kanazawa-shi, Ishikawa 920-8641
Tel: 076-265-2259, Fax: 076-234-4210

■ Kitasato University Hospital
Principal investigator: Shinichi Niwano, Medical Professor, Cardiovascular Medicine

Address: 1-15-1, Kitazato, Minami, Sagamihara, Kanagawa, 252-0375
Tel: 042-778-8111 Fax: 042-778-9371

■ Kyoto University Hospital
Principal investigator: Yugo Yamashita, Professor, Cardiovascular Medicine
Address: 54 Shogoin-Kawahara-cho, Sakyo-ku, Kyoto 606-8507
Tel: 075-751-4255, Fax: 075-751-3299

■ Kumamoto University Hospital
Principal investigator: Kenichi Tsujita, Professor, Cardiovascular Medicine
Address: 1-1-1 Honjo, Chuo-ku, Kumamoto City, Kumamoto 860-8556
Tel: 096-344-2111, Fax: 096-373-5906

■ Gunma Prefectural Cardiovascular Center
Principal investigator: Shigeto Naito, Director of the center
Address: 3-12, Kameizumi-town, Maebashi-city, Gunma 371-0004
Tel: 027-269-7455, Fax: 027-269-1492

■ Kokura Kinen Hospital
Principal investigator: Kenji Ando, Medical Director, Cardiovascular Medicine
Address: 3-2-1 Asano Kokurakita-Ku, Kitakyushu, Fukuoka 802-8555
Tel: 093-511-2000, Fax: 093-511-3240

■ National Cerebral and Cardiovascular Center
Principal investigator: Kengo Kusano, Director of the Cardiovascular Department
Address: 6-1 Kishibe-Shimmachi, Suita, Osaka, 564-8565
Tel: 06-6170-1070, Fax: 06-6170-1782

■ Saitama Medical University International Medical Center
Principal investigator: Ritsushi Kato, Professor, Division of Cardiology, Department of Cardiology
Address: 1397-1, Yamane, Hidaka-City, Saitama 350-1298
Tel: 042-984-4111, Fax: 042-984-4740

■ Sakakibara Heart Institute
Principal investigator: Junichi Nitta, Cardiovascular Medicine

Address: 3-16-1 Asahi-cho, Fuchu, Tokyo, 183-0003
Tel: 042-314-3111, Fax: 042-314-3150

■ Sapporo Medical University Hospital
Principal investigator: Tetsuji Miura, Professor, Department of Cardiovascular, Renal and Metabolic Medicine
Address: 16-291, Minami-ichijo-nishi, Chuo-ku, Sapporo 060-8543
Tel: 011-611-2111, Fax: 011-621-8059

■ Shiga University of Medical Science Hospital
Principal investigator: Yoshihisa Nakagawa, Professor, Cardiovascular Medicine
Address: Seta-Tsukinowa-cho, Otsu, Shiga 520-2192
Tel: 077-548-2111, Fax: 077-543-5839

■ Jichi Medical University Saitama Medical Center
Principal investigator: Tomio Umemoto, Cardiovascular Medicine
Address: 1-847 Amanuma-cho, Omiya-ku, Saitama 330-8503
Tel: 048-647-2111, Fax: 048-648-5166

■ Jichi Medical University Hospital
Principal investigator: Kazuomi Kario, Professor, Cardiovascular Medicine
Address: 3311-1 Yakushiji, Shimotsuke-shi, Tochigi 329-0498
Tel: 0285-58-7344, Fax: 0285-44-8169

■ Juntendo University Urayasu Hospital
Principal investigator: Takashi Tokano, Associate Professor, Cardiovascular Medicine
Address: 2-1-1 Tomioka, Urayasu-shi, Chiba 279-0021
Tel: 047-353-3111, Fax: 047-382-8011

■ Juntendo University Hospital
Principal investigator: Hidemori Hayashi, Associate Professor, Cardiovascular Medicine
Address: 3-1-3 Hongo, Bunkyo-ku, Tokyo 113-8431
Tel: 03-3813-3111, Fax: 03-5689-0627

■ Chiba University Hospital
Principal investigator: Yusuke Kondo, Specially Appointed Assistant Professor, Cardiovascular Medicine
Address: 1-8-1 Inohana, Chuo-ku, Chiba-shi, Chiba, 260-8677
Tel: 043-222-7171, Fax: 043-224-3830

■ University of Tsukuba Hospital
Principal investigator: Masaki Ieda, Professor, Cardiovascular Medicine
Address: 2-1-1 Amakubo, Tsukuba, Ibaraki 305-8576
Tel: 029-853-3900, Fax: 029-853-3904

■ Tokyo Women's Medical University Hospital
Principal investigator: Morio Shoda, Professor, Cardiovascular Medicine
Address: 8-1. Kawada-cho, Shinjuku-ku, Tokyo, 162-8666
Tel: 03-3353-8111, Fax: 03-3356-0441

■ Nagasaki University Hospital
Principal investigator: Koji Maemura, Professor, Cardiovascular Medicine
Address: 1-chōme-7-1 Sakamoto, Nagasaki, 852-8501
Tel: 095-819-7200, Fax: 095-819-7215

■ Nagoya University Hospital
Principal investigator: Toyoaki Murohara, Professor, Cardiovascular Medicine
Address: 65 Tsurumai-cho, Showa-ku, Nagoya 466-8560
Tel: 052-741-2111, Fax: 052-744-2785

■ Niigata Prefectural Central Hospital
Principal investigator: Saigawa Takashi, Director of the Department of Cardiovascular Medicine Cardiovascular Medicine
Address: 205 Shinnan-cho, Joetsu-City, Niigata 943-0192
Tel: 025-522-7711, Fax: 025-521-3720

■ Niigata City General Hospital
Principal investigator: Kazuyoshi Takahashi, Director of the Department of Cardiovascular Medicine
Address: 463-7 Shumoku, Chuo-ku, Niigata City, Niigata 950-1197

Tel: 025-281-5151, Fax: 025-281-5187

■ Niigata University Graduate School of Medical and Dental Sciences
Principal investigator: Tohru Minamino, Part-time Lecturer, Cardiovascular Medicine
Address: 754, Ichibancho, Asahimachidori, Chuo-ku, Niigata-shi, Niigata 951-8510
Tel: 025-227-2185, Fax: 025-227-0774

■ Nippon Medical School Hospital
Principal investigator: Yoshiaki Kubota, Assistant Professor and Clinical Fellow, Cardiovascular Medicine
Address: 1-1-5 Sendagi, Bunkyo-ku, Tokyo 113-8603
Tel: 03-3822-2131, Fax: 03-5685-0987

■ Hirosaki University Hospital
Principal investigator: Hirofumi Tomita, Professor, Cardiology and Nephrology
Address: 53 Honcho, Hirosaki, Aomori 036-8563
Tel: 0172-33-5111, Fax: 0172-39-5189

■ University of Fukui Hospital
Principal investigator: Hiroshi Tada, Professor, Cardiovascular Medicine
Address: 23-3 Matsuoka-shimoaizuki, Eiheiji-cho, Yoshida-gun, Fukui 910-1193
Tel: 0776-61-8800, Fax: 0776-61-8801

■ Fukushima Medical University Hospital
Principal investigator: Takashi Kaneshiro, Professor, Cardiovascular Medicine
Address: 1 Hikarigaoka, Fukushima-city, Fukushima 960-1295
Tel: 024-547-1111, Fax: 024-547-1998

■ Hokkaido University Hospital
Principal investigator: Toshihisa Anzai, Professor, Department of Cardiovascular Medicine
Address: 5 Kita14jonishi, Kita-Ku, Sapporo, Hokkaido 060-8648
Tel: 011-716-1161, Fax: 011-706-7627

■ Yamagata University Hospital

Principal investigator: Masafumi Watanabe, Professor, First Department of Internal Medicine
Address: 2-2-2 Iida-Nishi, Yamagata-city, Yamagata, 990-9585
Tel: 023-628-5302, Fax: 023-628-5305

■ Yamaguchi University Hospital
Principal investigator: Masafumi Yano, Professor, Department of Medicine and Clinical Science
Address: 1-1-1 Minami-Kogushi, Ube, Yamaguchi 755-8505
Tel: 0836-22-2111, Fax: 0836-22-2246

If you decide to participate in the study after fully understanding the content of the study, please sign and date the consent form. Thereafter, you will receive the patient information sheet and consent form for you to keep.

Consent Form

Dear Director of XX Hospital

I hereby give voluntary consent to participate in the study titled "Double-blind, placebo-controlled study to evaluate the change in the number of severe arrhythmias after empagliflozin intervention in patients with an implanted arrhythmia treatment device complicated by type 2 diabetes mellitus" (EMPA-ICD) after receiving a thorough explanation about the following contents of the patient information sheet from my study doctor and fully understanding the explanation and contents regarding my participation in the study:

- A Certified Review Board reviewed the study.
- The study was approved by the Certified Review Board, and the protocol was submitted to the Ministry of Health, Labour and Welfare.
- Purpose of the study
- Summary of the study
- How samples and data will be used and how long they will be stored
- Expected advantages and possible risks of participation in the study
- Expected effects and adverse reactions, and disadvantages to patients
- Other treatments
- Actions after completion of the clinical study
- My decision to participate in the study is voluntary
- Protection of my human rights
- What I will have to do
- Study-related costs for patients
- Compensation for the study
- Possible conflicts of interest
- Monitoring and auditing
- Disclosure of the study information
- Contacts for the study

* Optional tests (Please check one of the following boxes for each test.)

^{123}I-MIBG myocardial scintigraphy	□ Consent □ No consent
DNA and RNA tests, metabolome analysis	□ Consent □ No consent

(Patient) I understand that I can withdraw this consent at any time.

Consent date: MM/DD/YYYY ______________________

Name (signature): ______________________

(Study doctor)

Explanation date: MM/DD/YYYY ______________________

Department: ______________________

Name: ______________________

(Explanation assistant)

Explanation date: MM/DD/YYYY ______________________

Name: (Affiliation) ______________________

(Copy) For the patient

Consent Form

Dear Director of XX Hospital

I hereby give voluntary consent to participate in the study titled “Double-blind, placebo-controlled study to evaluate the change in the number of severe arrhythmias after empagliflozin intervention in patients with an implanted arrhythmia treatment device complicated by type 2 diabetes mellitus” (EMPA-ICD) after receiving a thorough explanation about the following contents of the patient information sheet from my study doctor and fully understanding the explanation and contents regarding my participation in the study:

- A Certified Review Board reviewed the study.
- The study was approved by the Certified Review Board, and the protocol was submitted to the Ministry of Health, Labour and Welfare.
- Purpose of the study
- Summary of the study
- How the samples and data will be used and how long they will be stored
- Expected advantages and possible risks of participation in the study
- Expected effects and adverse reactions, and disadvantages to patients
- Other treatments
- Actions after completion of the clinical study
- My decision to participate in the study is voluntary
- Protection of my human rights
- What I will have to do
- Study-related costs for patients
- Compensation for the study
- Possible conflicts of interest
- Monitoring and auditing
- Disclosure of the study information
- Contacts for the study

* Optional tests (Please check one of the following boxes for each test.)

^{123}I-MIBG myocardial scintigraphy	□ Consent □ No consent
DNA and RNA tests, metabolome analysis	□ Consent □ No consent

(Patient) I understand that I can withdraw this consent at any time.

Consent date: MM/DD/YYYY ____________________

Name (signature): ____________________

(Study doctor)

Explanation date: MM/DD/YYYY ____________________

Department: ____________________

Name: ____________________

(Explanation assistant)

Explanation date: MM/DD/YYYY ____________________

Name: (Affiliation) ____________________

*** Consent Withdrawal Form ***

Dear Director of XX Hospital

I hereby wish to WITHDRAW my consent to participate in the study titled “Double-blind, placebo-controlled study to evaluate the change in the number of severe arrhythmias after empagliflozin intervention in patients with an implanted arrhythmia treatment device complicated by type 2 diabetes mellitus” (EMPA-ICD).

If there are any anonymized registered data (including data from the performed tests):

☐ I approve the use of such data.
☐ I do not approve the use of such data.

(Patient)

Consent withdrawal date: MM/DD/YYYY

Name (signature): ____________________

I confirm the withdrawal of the consent.

(Study doctor)

Consent withdrawal confirmation date: MM/DD/YYYY ________

Department: ____________________

Name: ____________________

Name: (Affiliation) ____________________

Information on Adverse Reactions

Comparison of empagliflozin or placebo for prevention of lethal ventricular arrhythmia in type 2 diabetic patients with implantable cardioverter defibrillators (ICD)

EMPA-ICD

This document describes major adverse reactions that may be caused by treatment with the investigational drug. Rarely, some adverse reactions may lead to severe symptoms. Please read through this document, and **immediately contact your study doctor if you are worried that you have any of the symptoms described in this document or other symptoms.** Your study doctor will change your treatment or appropriately treat your symptoms.

If there are any words in this document that you don't understand or if you have any questions, please feel free to contact us.

EMPA-ICD Study Group

Prepared on: May 15, 2019 (ver. 1.1)

1. Hypoglycemia

A condition in which your blood sugar (glucose) level is lower than normal because of the effect of the investigational drug.

<Major symptoms>

Hunger, palpitation, light-headed feeling, headache, dizziness/vertigo, cold sweat

<Actions to be taken and points to bear in mind>

- If you have any of the above symptoms, it is important to first take sugar/glucose*.
  - * If you are taking any alpha-glucosidase inhibitor, please take glucose and not sugar.
  - * If you experience any of the above symptoms of hypoglycemia while you are taking sulfonylurea or insulin preparations, please eat some food containing sugar.
- If the symptoms do not get better, please immediately contact your study doctor.
- The combination the study drug with another drug you are taking may cause hypoglycemia. Therefore, if you are taking any other drugs, please inform your study doctor of the name of the drugs.

2. Dehydration

The investigational drug may increase the frequency of urination or volume of urine, possibly causing dehydration.

<Major symptoms>

Thirst, lethargy, dizziness/vertigo, low urine volume

<Actions to be taken and points to bear in mind>

- To prevent dehydration, please be careful to drink water frequently while taking the investigational drug. Please do not decide to stop drinking water.
- If any of the following conditions apply to you, please pay special attention to the onset of symptoms of dehydration:
  - Participating in the study during a high temperature season
  - Diarrhea or vomiting
  - Poor glucose control
  - Older person
  - Taking a diuretic agent
  - Renal impairment

3. Ketoacidosis

<Major symptoms>

Nausea and vomiting, decreased appetite, abdominal pain, abnormal thirst, lethargy, shortness of breath (dyspnea), consciousness disorder

<Actions to be taken and points to bear in mind>

- If you experience any of the above symptoms, please immediately visit a medical institution.
- Please avoid excessively restricting sugar, that means do not excessively restrict carbohydrate-containing foods such as rice, breads, and noodles.
- If you meet any of the following conditions, please pay special attention to the symptoms listed above:
  - Taking insulin preparations
  - Recent dose reduction or discontinuation of insulin preparations
  - A doctor found reduced insulin secretion function
  - Older person
  - Taking a diuretic agent
  - Renal impairment
  - Reduced food intake
  - Infection
  - Likely to be dehydrated

4. Urinary tract infection, genital infection

Because the investigational drug reduces blood glucose levels by causing glucose to be excreted in the urine, urinary tract and genital infections may be likely to occur.

<Major symptoms>

Urinary tract infection: Increased urination frequency, pain during urination, residual urine

Genital infection: Change in color or odor of vaginal discharge (in women), genital itching (pruritus)

<Actions to be taken and points to bear in mind>

Because the above symptoms may lead to serious infections, please be careful to do the following things to prevent infections:

- Do not avoid urinating.
- Keep the genital area clean.
- Be careful to always be fully hydrated.

5. Skin symptoms

Skin symptoms may be caused by an allergy to the investigational drug.

<Major symptoms>

Redness, itchiness, acne-like rash

<Actions to be taken and points to bear in mind>

Because the above symptoms may lead to major adverse reactions, please immediately contact your study doctor if you notice any skin symptoms.

(For patients)

Ver. 1.1
Prepared on: February 6, 2019

Summary of Compensation System for the Study

This study will be conducted carefully, but nevertheless we have prepared a compensation system in case of injury caused by adverse reactions, etc. This document aims to elaborate the information on the compensation for the study described in the Informed Consent Form. Please keep this document in a safe place along with the patient information sheet and a copy of the consent form.
If you experience any symptom you have never had before, get sick or notice anything different while you are participating in the study, please quickly contact your study doctor, who will immediately provide you with appropriate treatment.

1. Principle of compensation

1) Only the injury caused by (participation in) the study that is causally related to the study will be compensated, and any third party will not be liable for damages resulting from the study.
2) Even after signing the study consent form, you may file a lawsuit claiming liability for damages if such liability arises. This compensation system will not preclude your right to claim liability for damages.

2. Events that will not be compensated

1) Events will not be compensated for which there is no direct causal relationship between the study and the injury, such as a traffic accident on the way to the hospital. The person responsible for causing the injury will be liable for damages resulting from such events.
2) Events whose causal relationship with the study is ruled out, such as those that have a clear causal relationship with something else, those for which the temporal relationship between the use of the study drug and the injury makes it unreasonable to expect that the injury resulted from the study, and those that are irrational.
3) You will not be compensated if you do not receive a therapeutic benefit because of "no effect of the investigational product," etc.

3. Limited compensation

1) If you acted with gross negligence, such as making a false declaration and not following the study doctor's instructions, you will not be compensated or will be only partly compensated.

4. Compensation payment

If you have any Grade of Disability grade 1, 2 or 3 sequelae, as specified in the National and Employee's Pension Insurance Systems, or if you die, the amount we will pay will be calculated depending on the type and degree of disability and by referring to the amounts of benefits permitted under such systems.

You will receive compensation for medical costs and a medical allowance if you need treatment as a result of a physical disability caused by the adverse reaction to the study drugs (except the effects described in the Informed Consent Form and the Information on Adverse Reactions and those explained to you by your study doctor during informed consent). Please contact your study doctor for the amount.

5. Claiming compensation

1) If you suffer any injury, please contact your study doctor.
2) Please note that for compensation payment you may be asked to submit necessary documents, including a copy of your health insurance card.
3) In principle, compensation will be transferred to the bank or post office account designated by you. Please note that it usually takes 2 to 3 months to complete the transfer procedures.

If you have any questions about the compensation system, please feel free to contact your study doctor.

Procedures for Special Blood Tests

Comparison of empagliflozin or placebo for prevention of lethal ventricular arrhythmia in type 2 diabetic patients with implantable cardioverter defibrillators (ICD)

EMPA-ICD

Representative Investigator: Tohru Minamino
Part-time Lecturer, Department of Cardiovascular Medicine, Niigata University Graduate School of Medical and Dental Sciences

1 : Purpose and scope of the procedures

This document describes the procedures for performing special blood tests at each study site during the study.

2 : Schedule for special blood tests

	Baseline		Treatment period		
	Eligibility screening	Visit 1	Visit 2	Visit 3	At the time of discontinuation
	Week -12 to 0	Week 0	Week 12	Week 24	
		–	(± 4 weeks)	(± 4 weeks)	
Hematological tests (special)		○*		○	△

○, Essential; △, Optional

Wherever possible, fasting blood will be collected after patients have rested in supine position on the examination table for 30 minutes at Week 0* (baseline) and Week 24 (or the time of discontinuation).

*Results of tests performed between informed consent and Week 0 will be used.

3 : Special blood test endpoints

(1) Blood catecholamine fraction: adrenalin, noradrenalin, dopamine

(2) Blood ketone body fraction: acetoacetic acid, 3-hydroxybutyric acid, total ketone bodies

(3) Erythropoietin

(4) Reticulocyte count

(5) DNA tests: telomere length (no genetic analysis)

(6) RNA tests: P53, P21, P16

(7) Metabolome analysis

In principle, the above tests (5) to (7) should be performed only at the sites in Niigata Prefecture.

4 : Procedures for performing special blood tests

*** For blood sampling, please use the spitz tubes and the tubes for submission that were delivered to the study sites before the study.**

(1) Blood catecholamine fraction (essential)

[Spitz tube to be used: EDTA-2Na]

■ Procedures at each study site:

[1] Collect venous blood (one 7-mL tube) from the upper arm of the patient.

[2] Mix well and promptly separate the plasma by centrifuging at 2,000 rpm for 10 minutes at a low temperature (4°C).

[3] Transfer the supernatant into a designated container (poly-spitz tube pre-labeled for submission to SRL) and store frozen at -20°C.

[5] Tubes will be collected by SRL.

Test item	Sampling volume	Tube for sampling → Treatment before submission → Tube for submission	Volume to be submitted	Submission and storage
**3 fractions of catecholamines**	Whole blood 7 mL	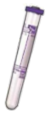 Purple cap (with EDTA-2Na) → 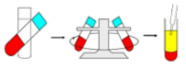 ・After sampling, mix well and promptly separate the plasma by centrifuging approximately at 2,000 rpm for 10 minutes at low temperature (4°C). ・Transfer the supernatant into a designated container (poly-spitz tube pre-labeled for submission to SRL) and store frozen at -20°C. → 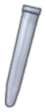	Plasma 1.5 mL	**Frozen**

(2) Blood ketone body fraction (essential)

(3) Erythropoietin (essential)

[Spitz tube to be used: Serum Separator Tube AutoSep]

■ Procedures at each study site ([1] to [3] for both blood ketone body fraction and erythropoietin)

[1] Collect venous blood (one 5-mL tube) from the upper arm of the patient.

[2] After sampling, promptly centrifuge with cooling at 2,000 rpm for 10 minutes.

[3] Wait for clot retraction under refrigeration if possible and then separate the serum within 2 hours.

■ For blood ketone body fraction:

[4] Transfer 0.3 mL of the supernatant into a designated container (poly-spitz tube pre-labeled for submission to SRL) and store frozen at -20°C.

[5] Tubes will be collected by SRL.

■ For erythropoietin:

[4] Transfer 0.8 mL of the supernatant into a designated container (poly-spitz tube pre-labeled for submission to SRL) and store refrigerated.

[5] Tubes will be collected by SRL.

Test item	Sampling volume	Tube for sampling	Treatment before submission	Tube for submission	Volume to be submitted	Submission and storage
**Blood ketone body fraction**	Whole blood 5-4 mL	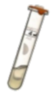 Tube for serum separation	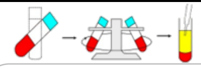 For blood ketone body fraction:<br>・After sampling, promptly centrifuge with cooling.<br>・Wait for clot retraction under refrigeration if possible and then preferably separate the serum and store frozen within 2 hours.<br>・Centrifuge at approximately 2,000 rpm for 10 minutes.<br>・Transfer the supernatant into a designated container (poly-spitz tube pre-labeled for submission to SRL) and store frozen at -20°C.<br>*After serum separation, for the erythropoietin test please transfer 0.8 mL of the serum sample for submission under refrigeration.	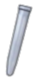	Serum 0.3 mL	**Frozen**
**Erythropoietin**				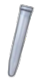	Serum 0.8 mL	**Refrigerated**

* Blood ketone body fraction ⇒ Because acetoacetate is unstable, please separate the serum and, after completion of clot retraction, store it frozen wherever possible.

(4) Reticulocyte count (essential)

[Spitz tube to be used: EDTA-2K]

■ Procedures at each study site:

[1] Collect venous blood (one 2-mL tube) from the upper arm of the patient.

[2] Store refrigerated.

[3] Tubes will be collected by SRL.

Test item	Sampling volume	Tube for sampling	Treatment before submission	Tube for submission	Volume to be submitted	Submission and storage
**Reticulocyte count**	Whole blood 2 mL		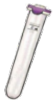 Collect into and submit in a pre-labeled tube. Purple cap (with EDTA-2K)		Whole blood 2 mL	**Refrigerated**

(5) DNA tests: telomere length (optional): In principle, perform only at the sites in Niigata Prefecture.

[Spitz tube to be used: EDTA-2Na tube (5 mL)]

■ Procedures at each study site:

[1] Collect venous blood (two 5-mL tubes) from the upper arm of the patient.

[2] Store the spitz tube frozen at -20°C (no sample transfer).

[3] Send **frozen** within 3 days (not including the sampling day).

■ Procedures at the Cardiovascular Medicine, Graduate School of Medicine of Juntendo University:

[1] Extract genomic DNA with Puregene Blood Core Kit (Qiagen).

[2] In obtained DNA (1 ng/uL), measure telomere length with LightCycler 480 (Roche Applied Science), Light Cycler 480 SYBR Green I Master (Roche), and the following primer:

[Primer]

Telomere: CGGTTTGTTTGGGTTTGGGTTTGGGTTTGGGTTTGGGTT,

GGCTTGCCTTACCCTTACCCTTACCCTTACCCTTACCCT

36B4; CAGCAAGTGGGAAGGTGTAATCC, CCCATTCTATCATCAACGGGTACAA

*** Do not perform analysis of genetic information.**

(6) RNA tests: P53, P21, P16 (optional): In principle, perform only at the sites in Niigata Prefecture.

[Spitz tube to be used: PAXgene tube (BD)]

■ Procedures at each study site:

[1] Collect venous blood (two 2.5-mL tubes) from the upper arm of the patient.

[2] Let stand for 2 hours or more at room temperature.

[3] Store the spitz tube frozen at -20°C (no sample transfer).

[4] Send **frozen** within 3 days (not including the sampling day).

■ Procedures at the Cardiovascular Medicine, Graduate School of Medicine of Juntendo University:

[1] Extract RNA with PAXgene blood RNA kit (Qiagen).

[2] Prepare cDNA from the obtained RNA with QuantiTect Reverse Transcription Kit (QIAGEN).

[3] Quantitatively evaluate senescence markers（P53, P21, P16）with Taqman Universal ProbeLibrary (Roche Applied Science) with the Light Cycler 480 Probes Master (Roche) and the following primers:

[Primers]

CDKN1A(p21); cgaagtcagttccttgtggag, catgggttctgacggacat

CDKN2A(p16); gtggacctggctgaggag, ctttcaatcggggatgtctg

Tp53; aggccttggaactcaaggat, ccctttttggacttcaggtg

RPLP0(60s); gatgcccagggaagacag,acaatgaagcattttggataa

*** Do not perform analysis of genetic information.**

(7) Metabolome analysis (optional): In principle, perform only at the sites in Niigata Prefecture.

[Spitz tube to be used: EDTA-2Na tube (5 mL)]: VENOJECT II Evacuated Tube (Code No. VP-NA050K)

■ Procedures at each study site:

[1] Collect venous blood (one 5-mL tube) from the upper arm of the patient.

[2] Mix well and promptly separate the plasma by centrifuging at 3,000 rpm for 10 minutes at low temperature (4°C).

[3] Aliquot the supernatant into designated containers (Thermo cryotubes, 200 μL each) and store frozen at -80°C.

[4] Send **frozen** within 3 days (not including the sampling day).

■ Procedures at the Department of Cardiovascular Medicine, Niigata University

[1] Extract metabolites by the Metabolite Extraction Method of the Institute for Advanced Biosciences, Keio University.

[2] Store at -80°C and send frozen to the Institute for Advanced Biosciences, Keio University.

5: How to pack samples for special blood tests

For tests (5) to (7), pack as follows:

[From the top]

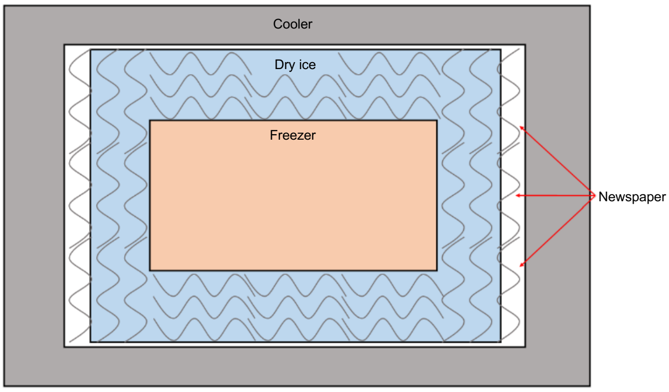

[From the side]

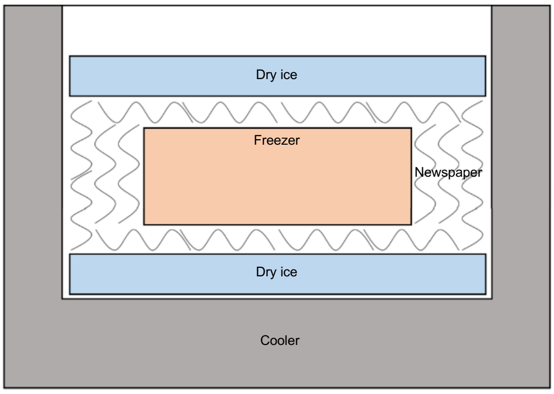

[Inside the freezer]

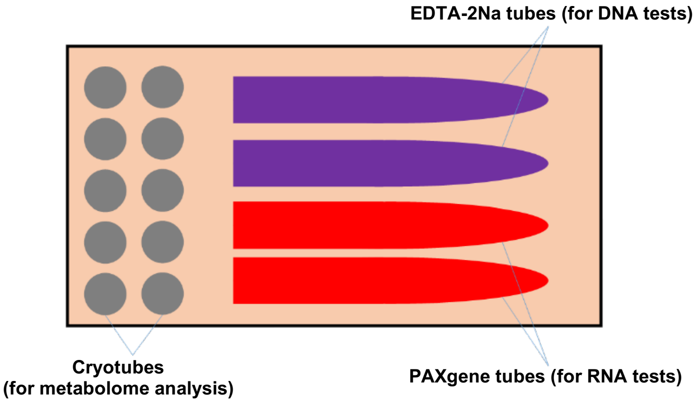

6: How to send samples of special blood tests

For tests (5) to (7), send samples to the following address and designate a delivery time in the morning:

Room 533, West Research Building, Department of Cardiovascular Medicine,<br>Niigata University Graduate School of Medical and Dental Sciences<br>Asahimachi-Dori 1-757, Chuo-ku, Niigata, Niigata 951-8510, Japan<br>Tel: 025-227-2189

Procedures for Holter Monitoring

Comparison of empagliflozin or placebo for prevention of lethal ventricular arrhythmia in type 2 diabetic patients with implantable cardioverter defibrillators (ICD)

EMPA-ICD

Representative Investigator: Tohru Minamino
Part-time Lecturer, Department of Cardiovascular Medicine, Niigata University Graduate School of Medical and Dental Sciences

1: Purpose and scope of the procedures

This document describes the procedures for performing Holter monitoring at each study site during the study.

2: Summary of Holter monitoring

Ventricular premature contraction (VPC) recorded by Holter monitor is a predictor of the onset of severe arrhythmia in patients with structural heart diseases, including old myocardial infarction [1] and dilated cardiomyopathy [2], and those with reduced left ventricular ejection fraction [3]. Holter monitoring will evaluate the number of VPCs not recorded by the arrhythmia device to determine the effect of the SGLT2 inhibitor.

3: Schedule for Holter monitoring

	Baseline		Treatment period		
	Eligibility screening	Visit 1	Visit 2	Visit 3	At the time of discontinuation
	Week -12 to 0	Week 0	Week 12	Week 24	
		–	(± 4 weeks)	(± 4 weeks)	
Holter monitoring		○*		○	△

○, Essential; △, Optional

To be performed at Week 0* (baseline) and Week 24 (can also be performed at the time of discontinuation).

*Results of Holter monitoring performed within 12 weeks before the test at Week 0 will be used even if they are obtained before informed consent.

4: Holter monitoring endpoints

(1) Total recording time

(2) Total number of VPCs

(3) Number of single VPCs

(4) Number of two-consecutive VPCs

(5) Number of ventricular tachycardia (at least three ventricular premature beats with a maximum mean R-R interval of 600 ms)

5: Procedures for performing Holter monitoring

・ Record with NASA and CM5 leads wherever possible.

・An experienced technician should attach electrodes and pay attention to the following points:

[1] Before attaching electrodes, pretreat with an alcohol pad or skin pretreatment to facilitate fixing and reduce skin resistance.

[2] After attaching electrodes, handle excess lead cords appropriately. Follow the procedures described in the instruction manual of each product.

[3] Determine the waveform amplitude and any murmur with the Holter monitor before starting the recording.

[4] Tell the patient about cautions to be taken during the test (for example, not using electronic devices, including electric blankets and cell phones, not touching electrodes, and not wearing clothes that are likely to cause static electricity).

・Record for 24 hours wherever possible.

・An experienced technician should scan and analyze the records.

5: How to evaluate Holter monitoring

・A cardiovascular specialist, principal investigator or subinvestigator should determine the number of recorded VPCs.

・Record the total recording time, total number of VPCs, number of single VPCs, number of occurrences of two consecutive VPCs, and number of ventricular tachycardia (at least three ventricular premature beats with a maximum mean R-R interval of 600 ms).

・Enter data in the electronic case report form.

6: References

[1] Circulation 1993; 87: 312-322.

[2] N Engl J Med 2004; 350: 2151-2158.

[3] Circulation 2000; 101: 40-46.

Procedures for Echocardiography

Comparison of empagliflozin or placebo for prevention of lethal ventricular arrhythmia in type 2 diabetic patients with implantable cardioverter defibrillators (ICD)

EMPA-ICD

Representative Investigator: Tohru Minamino
Part-time Lecturer, Department of Cardiovascular Medicine, Niigata University Graduate School of Medical and Dental Sciences

1: Purpose and scope of the procedures

This document describes the procedures for performing echocardiography at each study site during the study.

2: Schedule for echocardiography

	Baseline		Treatment period		
	Eligibility screening	Visit 1	Visit 2	Visit 3	At the time of discontinuation
	Week -12 to 0	Week 0	Week 12	Week 24	
		–	(± 4 weeks)	(± 4 weeks)	
Echocardiography	○*	○*		○	△

○, Essential; △, Optional

To be performed during eligibility screening and at Week 0* (baseline) and Week 24 (or the time of discontinuation).

*Results of tests performed within 12 weeks before the test at Week 0 will be used even if they are obtained before informed consent. Results of tests performed during eligibility screening, even the results of tests performed more than 12 weeks before the test at Week 0, will be used if they are obtained within 3 months of informed consent.

3: Echocardiography endpoints

(1) LVEF

(2) E wave

(3) A wave

(4) E/A; E/A should be assessed by measuring E and A waves.

(5) sep-e’

(6) lat-e’

(7) E/e’; E/e' should be assessed by measuring E wave and e' (septal and lateral).

(8) LAVI; LAVI should be assessed by measuring LAV.

(9) peak TRV

(10) HR

Values of the above endpoints will be calculated in EDC at each time point.

4: Procedures for performing echocardiography

Measurements should be performed by an experienced technician according to the updated ASE guidelines [1] [2] and as described below. Compliance with these instructions should be confirmed by a cardiovascular specialist or the principal investigator or a sub-investigator.

**Left ventricular ejection fraction (%EF)**	
To be measured from the apical four- and two-chamber views by using the biplane disk summation (modified Simpson) method.<br>If no asynergy is present in the left ventricle, the measurement may be performed with the apical four-chamber view by the single-plane disk summation method.<br>The volume and %EF calculated from the left ventricular diameter obtained from the left ventricular long axis view cannot be substituted.	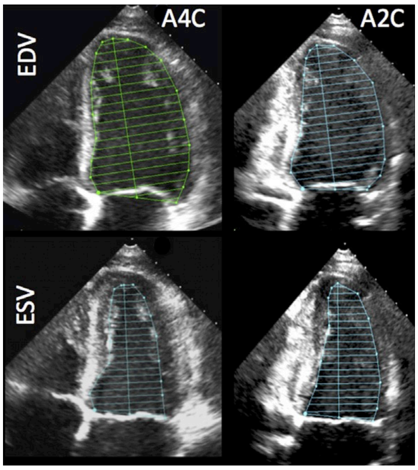
**Left atrial volume (LAV)**	
To be measured at end systole (when the left atrial chamber is at its greatest dimension) by using the biplane disk summation method on the apical four- and two- chamber views.	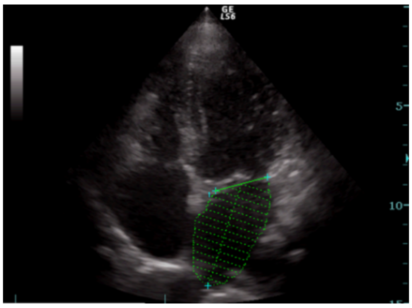<br>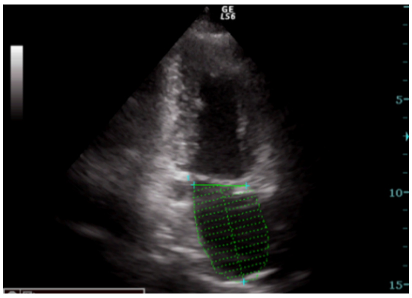

Transmitral flow velocity (E, A)

On the apical left ventricular long-axis view, a sample volume (preferably 3 mm) should be placed at the tip of the mitral valve at the maximum mitral valve opening in the early diastolic filling phase, and the center of the axis of blood flow into the left ventricle should be observed by color Doppler.

At the end of expiration during normal breathing, record stable velocity patterns for at least three beats; one of these recordings should be used to measure the maximal velocity at early diastole (E) and atrial contraction (A).

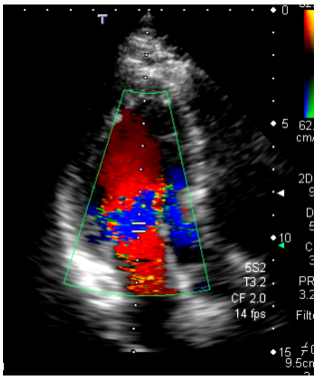

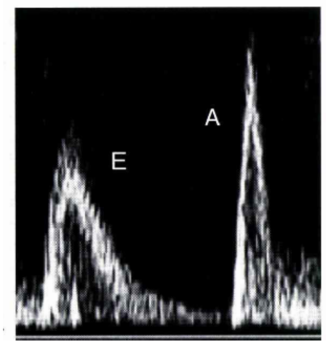

Velocities of mitral annulus (lat-e', sep-e')

Early diastolic velocity (e') of the septal and lateral mitral annulus should be measured by tissue Doppler imaging in the apical four-chamber view, where the ultrasound beam is positioned parallel to the direction of the mitral annular motions, preferably with a 10-mm sample volume.

When using the device without the tissue Doppler imaging mode, the velocities of mitral annulus should be measured by pulsed Doppler imaging for blood flow at lower Doppler gain, minimum filter, and a velocity range of approximately 20 to -20 cm/sec.

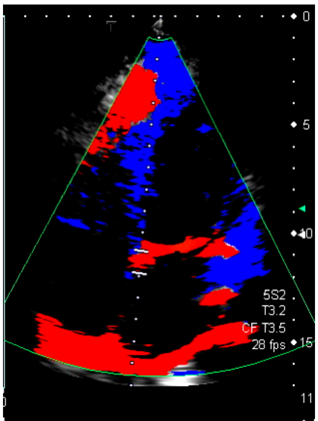

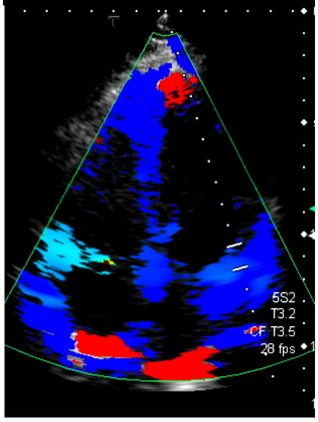

<table>
<tr><td></td><td>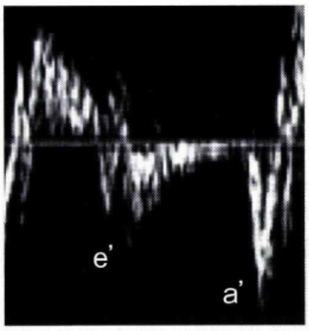
</td></tr>
<tr><td colspan="2">Tricuspid regurgitation peak velocity (peak TRV)</td></tr>
<tr><td>On the apical four-chamber view or the parasternal view (aortic valve level), a sample volume (preferably 3 mm) should be placed at the center of the axis of tricuspid regurgitation observed by color Doppler.
At the end of expiration during normal breathing, record stable velocity patterns for at least three beats; one of these recordings should be used to measure the maximal velocity of tricuspid regurgitation (TR).</td><td>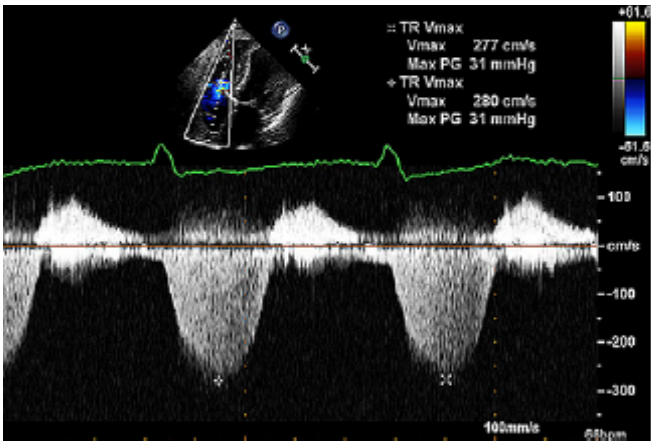
</td></tr>
<tr><td colspan="2">Heart rate (HR)</td></tr>
<tr><td>Record mean heat rate during echocardiography.
* For atrial fibrillation, use the mean of three heart rates with an average R-R interval.</td><td></td></tr>
</table>

5: References

[1] J Am Soc Echocardiogr 2015;28:1-39.

[2] J Am Soc Echocardiogr 2016;29:277-314.

Procedures for ^{123}I-MIBG Myocardial Scintigraphy

Comparison of empagliflozin or placebo for prevention of lethal ventricular arrhythmia in type 2 diabetic patients with implantable cardioverter defibrillators (ICD)

EMPA-ICD

Representative Investigator: Tohru Minamino
Part-time Lecturer, Department of Cardiovascular Medicine, Niigata University Graduate School of Medical and Dental Sciences

1: Purpose and scope of the procedures

This document describes the procedures for performing ^{123}I-MIBG myocardial scintigraphy at each study site during the study.

2: Summary of ^{123}I-MIBG myocardial scintigraphy

3(meta)-iodobenzylguanidine (MIBG), which is used in ^{123}I-MIBG myocardial scintigraphy, is an imaging agent for nerve function that has the same dynamics as noradrenaline at sympathetic nerve endings. Thus, the degree of myocardial accumulation of MIBG reflects myocardial sympathetic nerve ending function and can be used to evaluate abnormal sympathetic nerve function, such as myocardial denervation. Various reports [1]-[5] indicate that MIBG can predict fatal arrhythmia and cardiac sudden death, so it is useful to periodically perform the test to evaluate change over time in the predicted adverse cardiovascular events [6]. The test is also used to determine therapeutic effects of drugs [7][8], and guidelines indicate its usefulness [9]. Therefore, in clinical practice it appears to be helpful to repeat the test to predict patient prognosis and evaluate treatment effects over time. Although it has been suggested that the difference in the camera or collimator causes site error, a standardization method has been established for determining measured values [10].

3: Schedule for ^{123}I-MIBG myocardial scintigraphy

	Baseline		Treatment period		
	Eligibility screening	Visit 1	Visit 2	Visit 3	At the time of discontinuation
	Week -12 to 0	Week 0<br>–	Week 12<br>(± 4 weeks)	Week 24<br>(± 4 weeks)	
^{123}I-MIBG myocardial scintigraphy		△*		△	△

Can be performed at Week 0* (baseline) and Week 24 (or the time of discontinuation). The test is optional at both time points.

*Results of the test performed within 12 weeks before the test at Week 0 will be used even if they are obtained before informed consent.

4: ^{123}I-MIBG myocardial scintigraphy endpoints

(1) Heart-to-mediastinum ratio (HMR)

(2) Washout rate (WR)

5: Procedures for performing ^{123}I-MIBG myocardial scintigraphy

- A digital gamma camera with a widely used low-energy collimator should be prepared for imaging supine patients from an antero-posterior direction with the entire chest included in the field of view.
- Inject 111 MBq of ^{123}I-MIBG as an intravenous bolus into the cubital vein. Adjust the dose depending on the patient's age and weight, as applicable.
- Acquire the anterior (early) view 15 minutes after nuclide injection (acquisition time according to local conditions).
- Acquire the anterior (late) view 3 to 6 hours after nuclide injection (acquisition time according to local conditions).
- Whenever possible, patients should not use droxidopa, reserpine, tricyclic antidepressants, or labetalol hydrochloride because these drugs limit accumulation of ^{123}I-MIBG to the heart.
- A SPECT result report is not required, but it may be performed at the discretion of each site if needed.

[Example]

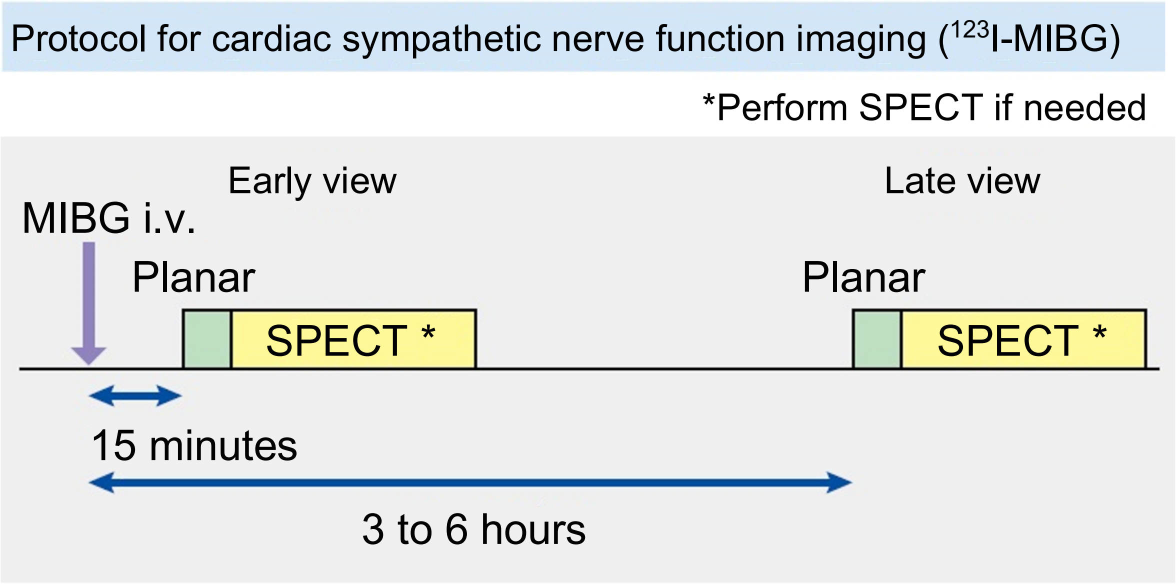

6: How to evaluate ^{123}I-MIBG myocardial scintigraphy

・How to evaluate: With the mediastinum as the background, set the region of interest in the heart (H) and the mediastinum (M). Determine the number of counts per pixel, measure the heart-to-mediastinum ratio (HMR) as a measure of myocardial accumulation and calculate the washout rate (WR) as a measure of the rate of release from the myocardium.
・Prepare the report with smartMIBG Heart (Fuji Film RI Pharma Co. Ltd.) [4].
・Report (1) the heart-to-mediastinum ratio (early and late views) and (2) the washout rate with the electronic data capture (EDC) system for the case report form (CRF).

7: References

[1] J Nucl Med 2009; 50: 61-67.
[2] Heart 2007; 93: 1213-1218.
[3] Nucl Med 2008; 49: 225-233.
[4] Eur J Nucl Med Mol Imaging 2006; 33: 866-870.
[5] J Am Coll Cardiol 2009; 53: 426-435.
[6] J Nucl Med 2008; 49: 907-914.
[7] Am Heart J 1997; 133: 353-358.
[8] J Nucl Cardiol 2001; 8: 4-9.
[9] Guidelines for Clinical Use of Cardiac Nuclear Medicine (JCS 2010)
[10] J Nucl Cardiol 2014; 21: 970-978.

Department of Cardiovascular Medicine, Niigata University Graduate School of Medical and Dental Sciences

Adverse events reporting procedures

Comparison of empagliflozin or placebo for prevention of lethal ventricular arrhythmia in type 2 diabetic patients with implantable cardioverter defibrillators (ICD)

Representative Investigator: Tohru Minamino, Department of Cardiovascular Medicine, Niigata University Graduate School of Medical and Dental Sciences, Niigata, Japan

Ver.1.5

12/13/2019

Contents

1. Purpose and scope of the document 2
2. Definition of adverse events in the study (Figure 1) 2
3. Definition and role of each person involved in the study 6
4. Reportable events 7
5. Procedures for reporting AEs 8
6. Where to report events that require expedited reporting 13
7. Breaking emergency key code 13
8. Storage of materials 13
9. History of changes 14

1. Purpose and scope of the document

This document defines adverse events and, to help standardize procedures, describes the role and workflow of people involved in the study.

2. Definition of adverse events in the study (Figure 1)

Adverse Event (AE)

An adverse event is any unfavorable or unintended disease or sign (e.g., an abnormal laboratory finding) in participants, regardless of the causal relationship with the study.

(1) Serious Adverse Event (SAE)

A Serious Adverse Event (SAE) is:

1) an AE that results in death,
2) a life-threatening AE,
3) an AE that requires hospitalization for treatment or prolongation of an existing hospitalization,
4) an AE that results in persistent or significant disability or incapacity,
5) an AE that results in a congenital anomaly, or
6) another event or reaction considered medically significant.

The phrase “considered medically significant” in point 6) above refers to an event that is appropriately medically evaluated as endangering the patient and requiring medical or surgical treatment so that it does not lead to an SAE as defined in points 1) to 5) above.

Patients may be hospitalized during the study period for study management or for social reasons (e.g., for fluid infusion if the hospital is too far away for the patient to visit the hospital for treatment and return home the same day). If hospitalization for such reasons was scheduled at initiation of the study, it was already recorded in the source document at the time of the eligibility screening tests. Therefore, these hospitalizations do not have to be reported as SAEs if they are conducted as scheduled.

(2) AEs of Special Interest

The term AE of Special Interest (AESI) refers to any specific AE that has been identified at the project level as being of particular concern for prospective safety monitoring and safety assessment during the study, e.g., potential AEs on the basis of knowledge from other compounds in the same class. Principal Investigators must report AESIs to the Representative Investigator within the same timeframe that applies to SAEs. The definitions of the AESIs in this study are as follows:

1) Liver disorder

A liver disorder is defined as the following changes in liver function:

AST and/or ALT increased **≥ 3-fold** above the upper limit of local laboratory reference ranges plus total bilirubin measured in the same blood sample increased **≥ 2-fold** above the upper limit of local laboratory normal ranges

AND/OR

AST and/or ALT increased **≥ 5-fold** above the upper limit of local laboratory normal ranges

The above findings are warning signs for a liver disorder. In patients with such laboratory abnormalities, the Principal Investigator or Subinvestigator should collect samples for measuring liver function test values as early as possible and follow up the liver function according to their clinical decision.

If a participant experiences any clinical symptom of liver disorder (jaundice, encephalopathy of unknown cause, coagulopathy of unknown cause, right upper abdominal pain, etc.) without laboratory test results (ALT, AST, total bilirubin), a Principal Investigator or Subinvestigator should perform the necessary blood tests to evaluate the above liver function test values.

2) Decreased kidney function

Decreased kidney function is defined as creatinine levels **≥ 2-fold** above the level at Week 0 and above the normal upper limit.

In patients with decreased kidney function, the Principal Investigator or Subinvestigator should collect samples for measuring creatinine as early as possible and follow up the kidney function according to their clinical decision.

3) Acidosis

If metabolic acidosis, ketoacidosis, or diabetic ketoacidosis (DKA) is suspected, the patient should be closely examined according to the clinical decision and clinical course until a diagnosis has been established and/or the patient has recovered.

According to the Japanese Clinical Practice Guideline for Diabetes 2016 of the Japan Diabetes Society, DKA is **a combination of hyperglycemia (glucose ≥ 250 mg/dL), hyperketonemia (elevation of beta-hydroxybutyric acid), and acidosis (pH < 7.3 and bicarbonate < 18 mEq/L)** resulting from extreme insulin deficiency and an increase of counterregulatory hormones that affect insulin.

A diagnosis of DKA does not require all of the above criteria to be met and should be based on a clinical decision. The mechanism of action of empagliflozin may change the clinical symptoms of DKA and may be associated with an onset of DKA at a lower glucose level than the above values.

4) Events associated with leg amputation

Events associated with leg amputation are defined as amputation (through the bones), disarticulation (through the joints), and autoamputation (spontaneous detachment of the part of the lower extremities that can no longer exist) and do not include other treatments without debridement (removal of callus or necrotic tissue); treatment of a stump (repair of stump, abscess drainage, wound repair, etc.); or amputation or disarticulation of extremities, such as nail excision and avulsion.

Leg amputation, disarticulation, and autoamputation should be individually reported. The report should describe the treatment date, level of amputation or disarticulation, medical condition leading to the relevant treatment, and, if applicable, any known risk factor for leg amputation in the patient.

5) Necrotizing fasciitis of the perineum and genitalia (Fournier's gangrene)

Cases of necrotizing fasciitis, a serious infection that causes necrosis of the subcutaneous tissue, that occur in the perineum and genitalia are called Fournier's gangrene. Physicians should pay attention to symptoms such as genital or perineal pain or tenderness, erythema or swelling, and fever or malaise because they may indicate suspected Fournier's gangrene.

Patients should be treated promptly because serious outcomes have been reported.

(3) <u>Disease or the like</u>

Among the AEs, those possibly related to the research are referred to as “disease or the like.”

A “disease or the like” set forth in Article 54 of the regulations for enforcing the Clinical Trials Act (hereafter called “Serious Diseases”) is reportable to the research institution administrator and the Certified Review Board.

(4) <u>Events that require expedited reporting</u>

In the study, the events that require expedited reporting are SAEs (including Serious Diseases) and AESIs.

(Figure 1: Definition of adverse events)

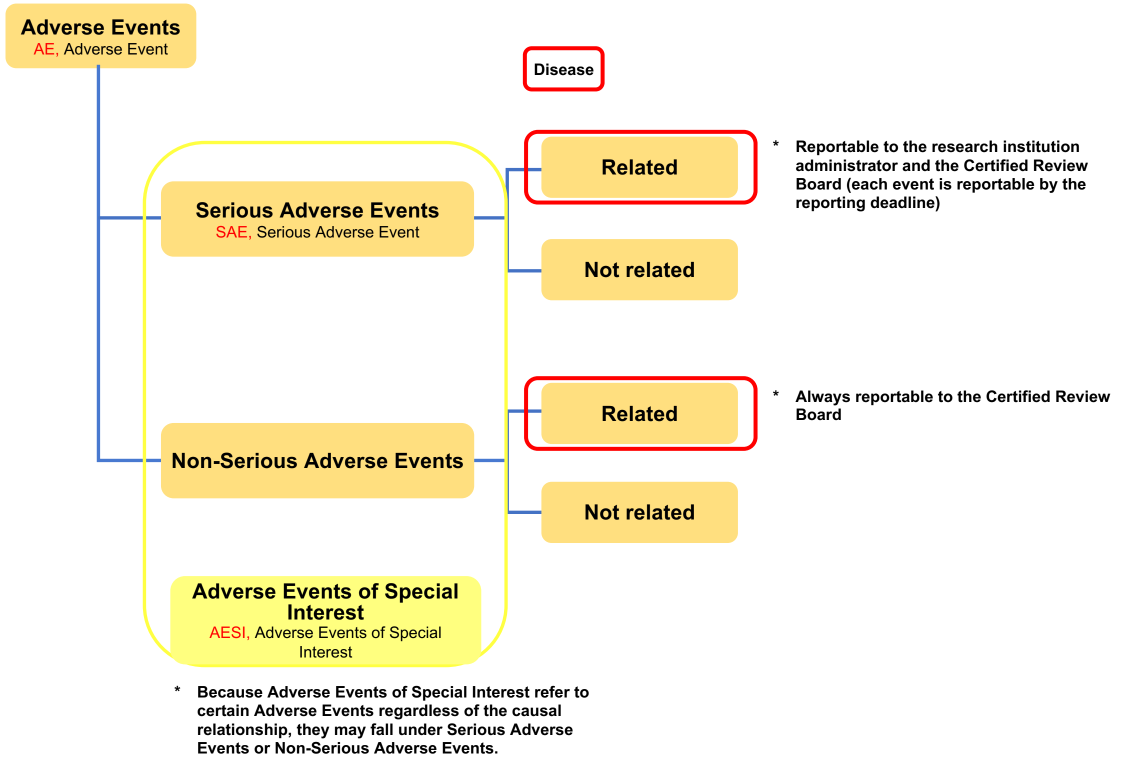

* **If an Adverse Event belongs to multiple categories, it should be reported according to the most serious category.**

<Serious Adverse Event>

[1] **Adverse Event that results in death**
[2] **Life-threatening Adverse Event**
[3] **Adverse Event that requires hospitalization for treatment or prolongation of existing hospitalization**
[4] **Adverse Event that results in persistent or significant disability or incapacity**
[5] **Adverse Event that results in a congenital anomaly**
[6] **Other event or reaction that is considered medically significant**

* Serious illnesses shall be specified in Article 54 of the Enforcement Regulations of the Clinical Trials Act.

<Adverse Event of Special Interest>

[1] **Liver disorder:**
- **a) AST and/or ALT increased ≥ 3-fold and total bilirubin increased ≥ 2-fold (relative to the upper limit of local laboratory reference ranges [ULLN])**
- **b) AST and/or ALT increased ≥ 5-fold (relative to ULLN)**

[2] **Decreased kidney function: creatinine ≥ 2-fold above Week 0 and beyond the normal upper limit**
[3] **Acidosis: metabolic acidosis, ketoacidosis, and diabetic ketoacidosis**
[4] **Leg amputation: amputation, disarticulation, and autoamputation**
[5] **Necrotizing fasciitis of the perineum and genitalia (Fournier's gangrene)**

3. Definition and role of each person involved in the study

(1) Representative Investigator

Tohru Minamino, Department of Cardiovascular Medicine, Niigata University Graduate School of Medical and Dental Sciences, Niigata, Japan

The Representative Investigator of the study is responsible for maintaining and storing detailed records, such as electronic case report forms (electronic data capture, hereafter called “EDC”) for all reported AEs associated with the study. In addition, to enforce the Clinical Trials Act, he will report AEs to the Certified Review Board in compliance with Article 54 of the regulations. If he receives any notification of a disease or the like from the Principal Investigator of a research institution, he will inform the Principal Investigators of the other research institutions participating in the study, as applicable.

In accordance with the recommendations of the Data and Safety Monitoring Board, he will notify all Principal Investigators participating in the study about safety information obtained during the study (other than information on a disease or the like).

(2) Research institutions:

The institutions that enroll patients in the study have the responsibility and obligation to collect and report all AEs that occur during the study (including SAEs/AESIs, other non-serious AEs, and diseases or the like).

(3) Principal Investigator:

The physician who supervises study operations at participating research institutions. For all AEs that occur at the research institution, the Principal Investigator is responsible for preparing, appropriately reporting, maintaining and storing detailed records, including EDC, etc. The Principal Investigator will report diseases or the like to the research institution administrator. The Principal Investigator will promptly notify the Representative Investigator of events that require expedited reporting, as specified for the study. If the Principal Investigator receives information from the Representative Investigator on a disease or the like at another research institution, he/she will promptly report the content of the provided information to the research institution administrator.

(4) Research office:

The office organized by the Representative Investigator. It is responsible for operations related to administrative research procedures in general, including those for AE reporting at the Department of Cardiovascular Medicine, Niigata University Graduate School of Medical and Dental Sciences.

(5) Assistant research office:

Clinical Research Department, Micron, Inc.

The assistant research office will support the Representative Investigator and the research office in study operations, including those related to AE reporting.

(6) Designated contact person for safety:

This person will be designated by the Representative Investigator and will be the contact for all safety-related communications and data and information exchange under the pharmacovigilance agreement executed between Niigata University and Nippon Boehringer Ingelheim Co., Ltd.

(7) Pharmacovigilance Department, Nippon Boehringer Ingelheim Co., Ltd:

The department in charge of safety operations management at the company funding the study. The department will be the contact for all safety-related communications and data and information

exchange.

(8) Datacenter:
Center for Clinical Research and Innovation (CCRI), Osaka City University Hospital, Osaka, Japan
The division in charge of data management for the study. It will collect information on AEs by EDC.

(9) Data and Safety Monitoring Board:
The board will monitor and evaluate the progress of the study and safety data, etc., and, as a third party, recommend to the Representative Investigator to continue, modify, or discontinue the study.

4. Reportable events

Reportable AEs

AEs that occur during the study between informed consent and Week 24 (or the time of discontinuation) are reportable.

Information to be recorded

In case of AEs, the Principal Investigator or Subinvestigator should record the following items:

(1) Name of AE and onset date
(2) Severity
(3) Whether it is an AESI
(4) Treatment and outcome, and date outcome is confirmed
(5) Causal relationship to the study
(6) Administration status of study drug (continued or discontinued)

[Outcome]

Outcome should be categorized into one of six categories: Recovered, Recovering, Not recovered, With sequelae, Death, and Unknown. In the cases of Recovered, Recovering, or Death, the date when outcome is confirmed is the date when the outcome was actually determined. If follow-up is discontinued because the outcome is Not recovered or Unknown, the date outcome is confirmed is the date when the Principal Investigator or Subinvestigator last confirms the outcome; the reasons for discontinuing follow-up or Unknown should be written in the comment section. If any disease or the like occurs, the patient should be followed until the outcome is determined.

[Causal relationship to study]

The causal relationship to study should be assessed in the two categories described below. If it is evaluated as “Able to be ruled out,” the reason should be written in the comment section.

[1] Causal relationship can be ruled out: Cases where a temporal relationship between the AE and study is unreasonable or where there is a medical explanation for the AE onset other than the study.

[2] Causal relationship cannot be ruled out (possibly related): In cases where the case [1] does not

apply, pursuant to the Clinical Trials Act, AEs whose causal relationship to the study cannot be ruled out are defined as "disease or the like."

5. Procedures for reporting AEs

All AEs

Record all AEs associated with the study in EDC, etc.

The AE reporting procedures specified in the Clinical Trials Act and specific for the study are described below. For AEs that occur during the study, reporting under the safety information reporting system for pharmaceuticals and medical devices and spontaneous reporting to the marketing authorization holder should be done appropriately, as specified by each research institution.

(1) Events that require expedited reporting

If a Principal Investigator or Subinvestigator notices the onset of any event that requires expedited reporting at the research institution, the investigator should promptly report it to the Representative Investigator. The Representative Investigator should report it according to each regulation. In the study, events that require expedited reporting refer to SAEs (including Serious Diseases) and AESIs.

1) Serious Diseases

If the Representative Investigator is notified of Serious Diseases, in compliance with Article 54 of the regulations for enforcement of the Clinical Trials Act the investigator should promptly inform the Principal Investigators at the other research institutions and report the event to the Certified Review Board. The date of awareness is the date on which the Principal Investigator or Subinvestigator becomes aware of the onset of the event.

<table>
<tr><th></th><th>Expectedness (*)</th><th>Seriousness</th><th>Reportable within</th></tr>
<tr><td rowspan="5">Other than the study with unapproved or off-label use of pharmaceuticals</td><td>Regardless of expectedness</td><td>Death (excluding infections)</td><td>15 days</td></tr>
<tr><td>Increasing concern about unexpectedness of the onset</td><td rowspan="2">Inpatient hospitalization or prolongation of existing hospitalization
・Disability
・Death or likelihood of leading to disability
・Death or Serious Disease, as described above
・Congenital disease or abnormality in later generations</td><td>15 days</td></tr>
<tr><td>Expected</td><td>30 days</td></tr>
<tr><td>Unexpected</td><td>Infection-related disease or the like</td><td>15 days</td></tr>
<tr><td>Regardless of expectedness</td><td>Infection-related:
・Death
・Inpatient hospitalization or prolongation of existing hospitalization
・Disability
・Death or likelihood it will lead to disability</td><td>15 days</td></tr>
</table>

		・Death or Serious Disease, as described above<br>・Congenital disease or abnormality in later generations	

* To be determined on the basis of the protocol or patient information sheet or the precautions described in the package insert, container, or wrapper of the pharmaceuticals used in the study.

2) SAE and AESI

If the Representative Investigator receives initial and follow-up reports on SAEs and AESIs, as a matter of principle the investigator should report them to the Pharmacovigilance Department, Nippon Boehringer Ingelheim Co., Ltd within 24 hours.

(2) Periodic reporting

To comply with Article 59 of regulations for enforcement of the Clinical Trials Act, the Representative Investigator should perform periodic reporting on the status of the clinical study and the occurrence of a disease or the like. Periodic reporting should be done within 2 months of the relevant surveillance period, which as a matter of principle is at the end of every year after the date when the protocol was submitted to the Minister of Health, Labour and Welfare.

In addition, under Article 60 of the regulations for enforcement of the Clinical Trials Act, periodic reporting to the Minister of Health, Labour and Welfare should be done regarding the matters set forth in Article 59, paragraph 1, item (i) of such regulations, within 1 month after the date when the Certified Review Board gives its opinion.

(3) Type of report form

1) Expedited report

[1] Serious Diseases

Form for reporting to the Certified Review Board: The report on a disease or the like associated with pharmaceuticals “Unified Form 8” and the form for describing the details, as specified in the “Regarding Unified Forms for the Clinical Trials Act” (Administrative communication of the Research and Development Division, Health Policy Bureau, Ministry of Health, Labour and Welfare [MHLW]).

[2] SAEs/AESIs

Form for reporting to the Pharmacovigilance Department, Nippon Boehringer Ingelheim Co., Ltd: “Unified Form 8,” the form for describing the details and the study-specific attachment.

2) Periodic report

[1] Form for reporting to the Certified Review Board: Periodic disease report “Unified Form 6,” as specified in the “Regarding Unified Forms for the Clinical Trials Act” (Administrative communication of the Research and Development Division, Health Policy Bureau, MHLW).

[2] Form for reporting to the Minister of Health, Labour and Welfare: Periodic report “Attachment Form 3,” as specified in “Regarding regulations for enforcement of the Clinical Trials Act.”

(4) Procedures for reporting AEs (Figures 2 and 3)

1) Reporting events that require expedited reporting

A. Procedures for reports from research institutions

[1] If a Principal Investigator or Subinvestigator notices the onset of any event that requires expedited reporting, the investigator should fill out the predetermined information in the report form and send it to the research office by fax or e-mail. For Serious Diseases, the report should be sent to the research institution administrator. For events other than Serious Diseases that require expedited reporting, the report should be sent to the research institution administrator, if necessary, as specified by each research institution. Regarding the causal relationship, the relationship between the relevant event and the study should be evaluated as “Related” or “Not related.” If the relationship between an event and the participant’s participation in the study is classified as Unknown, in the report the event will be regarded as being related to the study.

[2] If the research office receives an expedited report, it should immediately submit a written report to the Representative Investigator. The Representative Investigator should promptly report Serious Diseases to the Data and Safety Monitoring Board in writing and simultaneously request a review of such AEs.

[3] The Representative Investigator should report Serious Diseases to the research institution administrator and the Certified Review Board by the reporting deadline. In addition, the Representative Investigator should inform the Principal Investigators at each research institution, and those Principal Investigators should promptly report the relevant information to the research institution administrator.

[4] If the Representative Investigator receives initial and follow-up reports on any event that requires expedited reporting, as a matter of principle the investigator should submit a written report to the Pharmacovigilance Department, Nippon Boehringer Ingelheim Co., Ltd within 24 hours.

[5] The Data and Safety Monitoring Board should review the contents of the report from the Representative Investigator and should recommend future actions, including those related to a potential need for code breaking, handling of cases, and continuation/discontinuation of the research, to the Representative Investigator in writing; the procedures of the Data and Safety Monitoring Board will be described separately.

[6] After receiving the recommendation from the Data and Safety Monitoring Board, the Representative Investigator should communicate the decision on the continuation, modification, or discontinuation of the study to all research institutions. The Representative Investigator should inform the research institutions of AESIs if he deems it necessary.

B. Complementary collection by the assistant research office of events that require expedited reporting

[1] The assistant research office should regularly check the information entered by research institutions into EDC (apart from on Saturdays, Sundays, or holidays). If they find any event that falls into the category of events that require expedited reporting, as defined in paragraph 2 (4) of the procedures, the assistant research office will promptly ask the relevant research institution to collect the required information by using the report form and to submit the necessary documents.

[2] The assistant research office should use the report form to promptly provide the Representative Investigator with the information about the event that requires expedited reporting and was complementarily collected as described in [1] above.

(Figure 2)

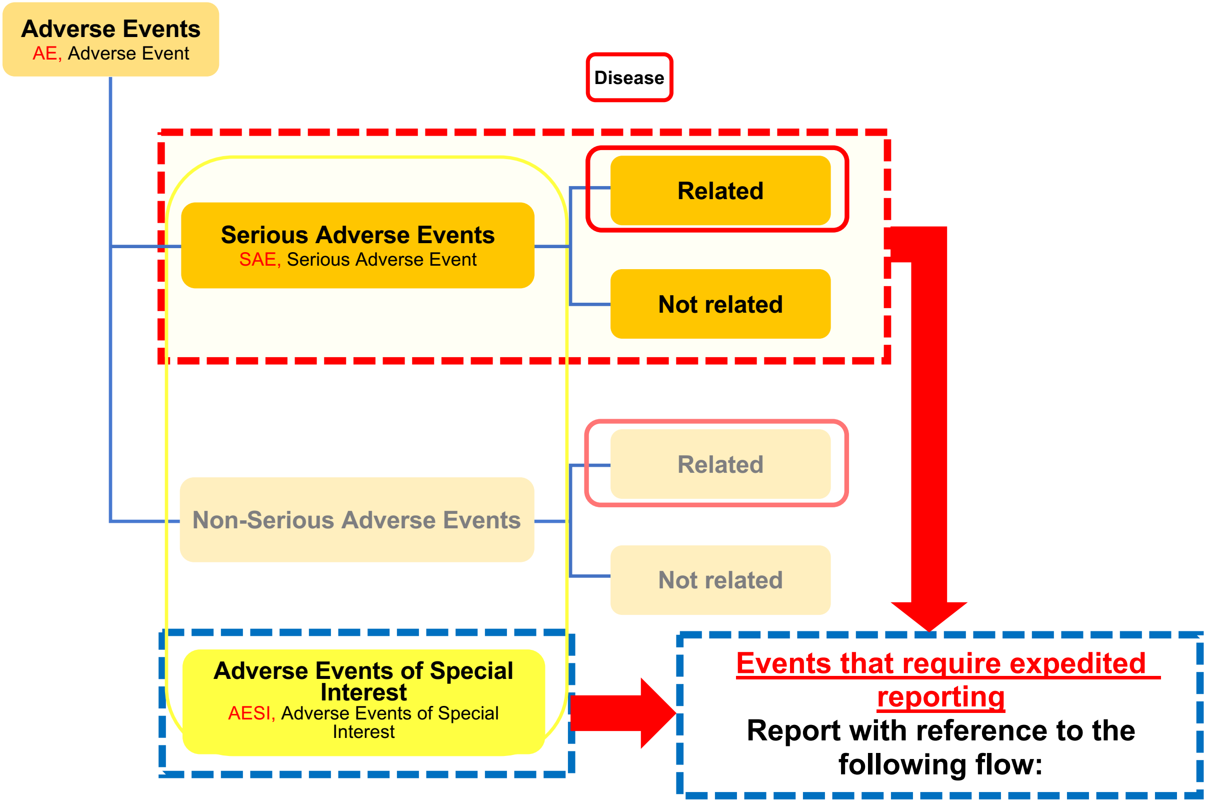

* All AE information should be entered into the case report form.

(Figure 3)

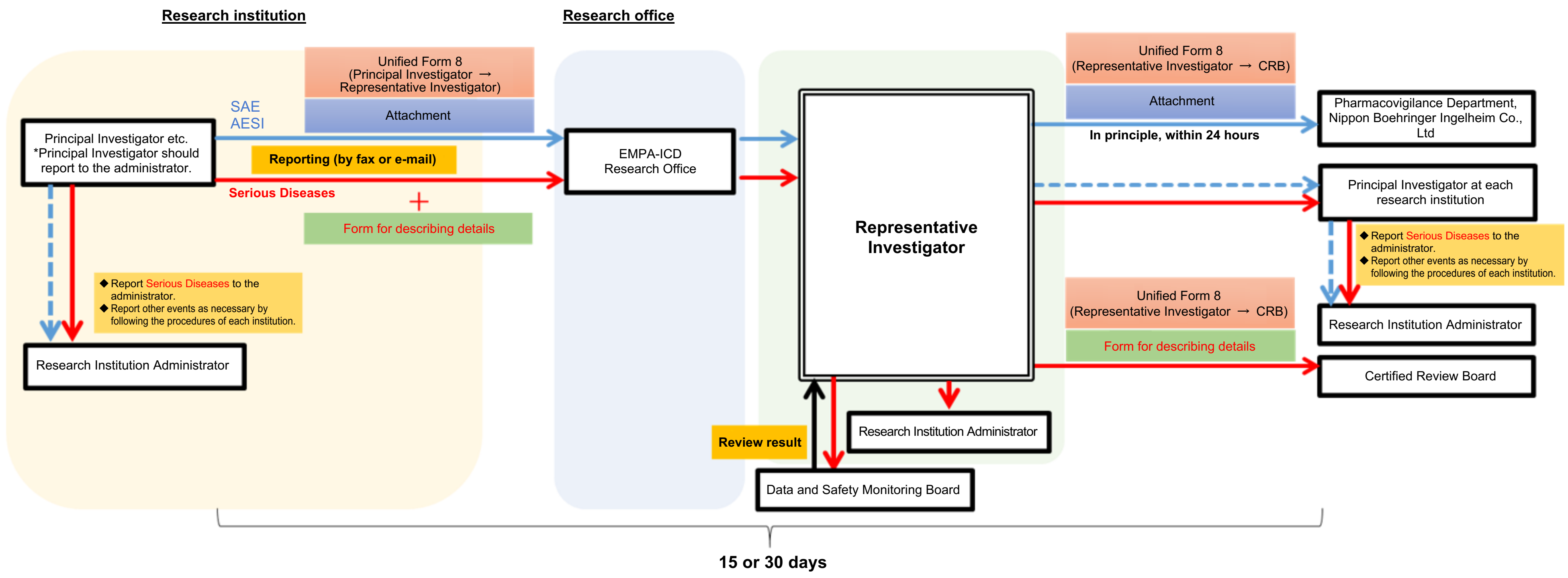
Research institution
Research office
Unified Form 8 (Principal Investigator → Representative Investigator)
Attachment
SAE
AESI
Principal Investigator etc. *Principal Investigator should report to the administrator.
Reporting (by fax or e-mail)
Serious Diseases
+
Form for describing details
EMPA-ICD Research Office
◆ Report Serious Diseases to the administrator.
◆ Report other events as necessary by following the procedures of each institution.
Research Institution Administrator
Representative Investigator
Unified Form 8 (Representative Investigator → CRB)
Attachment
In principle, within 24 hours
Pharmacovigilance Department, Nippon Boehringer Ingelheim Co., Ltd
Principal Investigator at each research institution
◆ Report Serious Diseases to the administrator.
◆ Report other events as necessary by following the procedures of each institution.
Unified Form 8 (Representative Investigator → CRB)
Form for describing details
Research Institution Administrator
Certified Review Board
Review result
Research Institution Administrator
Data and Safety Monitoring Board
15 or 30 days

6. Where to report events that require expedited reporting

(1) Events that require expedited reporting should be sent by fax or e-mail, as follows:

Fax: 03-6262-2815 (EMPA-ICD Assistant Research Office)

E-mail: empa-icd@micron-kobe.com (e-mail can be used if a fax machine is not available.)

7. Breaking emergency key code

If code breaking is required for reported AEs, follow “Procedures for Breaking Emergency Key Code.”

8. Storage of materials

Store materials related to AEs for the storage period described in the protocol.

9. History of changes

Version	Description of change
1.0	Initial preparation
1.1	Modification related to enforcement of the Clinical Trials Act
1.2	Additional description of required action in case of necrotizing fasciitis of the perineum (Fournier's gangrene)
1.3	Update regarding the amendment to the package insert of Jardiance tablets
1.4	• Standardization of words and phrases regarding the Procedures for Breaking Emergency Key Code<br>• Modification of procedure for reporting an SAE to the Data and Safety Monitoring Board
1.5	Modification to statement that the Data and Safety Monitoring Board will review only Serious Adverse Events and possibly related Adverse Events<br>Correction of errors and maintenance of descriptions

Department of Cardiovascular Medicine, Niigata University Graduate School of Medical and Dental Sciences

SOP in case of protocol violations

Comparison of empagliflozin or placebo for prevention of lethal ventricular arrhythmia in type 2 diabetic patients with implantable cardioverter defibrillators (ICD)

Representative Investigator: Tohru Minamino, Department of Cardiovascular Medicine, Niigata University Graduate School of Medical and Dental Sciences

Ver.1.2

3/23/2020

Contents

1. Purpose and scope of the document 3
2. Definition of protocol violation in the study 3
3. Definition and role of each person involved in the study 3
4. Reportable protocol violations 4
Reportable protocol violations 4
Timing of protocol violation reporting 4
5. Procedures for reporting protocol violations 4
6. Where to report protocol violations 7
7. Storage of materials 7
8. History of changes 8

1. Purpose and scope of the document

This document defines protocol violation and describes the role and workflow of people involved in the study to help standardize procedures for evaluating and reporting protocol violations that occur during the conduct of the study.

2. Definition of protocol violation in the study

Protocol violation

"Protocol violation" refers to incompliance with regulations, study protocol, and SOP and to tampering with and manipulating study data.

Serious protocol violation

"Serious protocol violation" refers to occurrences that influence the human rights and safety of patients, the progress of the study, and the reliability of the study results. For example, cases of serious protocol violation include incompliance with inclusion and exclusion criteria or study discontinuation criteria and combined use of treatments where combined use is prohibited; however, serious protocol violation does not include cases of deviation from the study protocol for medically necessary reasons to avoid an emergent risk in patients.

3. Definition and role of each person involved in the study

(1) Representative Investigator

Tohru Minamino, Department of Cardiovascular Medicine, Niigata University Graduate School of Medical and Dental Sciences

The Representative Investigator of the study should consider the wellbeing of participants; ensure that Principal Investigators, Subinvestigators, and other people involved in the study comply with regulations and the protocol; report and recognize protocol violations; and share information in a timely manner with people involved in the study. In addition, regarding all reported protocol violations associated with the study, the Representative Investigator is responsible for maintaining and storing detailed records, including electronic case report forms (electronic data capture, hereafter called "EDC"). If, on the basis of the information obtained during the study, the protocol violation is deemed to be a serious protocol violation after receiving the required opinion from the Data and Safety Monitoring Board or if it is reported as a serious protocol violation by a Principal Investigator, the Representative Investigator should promptly request the opinion of the Certified Review Board. The Representative Investigator should also take measures to prevent recurrence of the protocol violation; communicate such measures to Principal Investigators, Subinvestigators, and other people involved in the study; and ensure that recurrence is prevented. After obtaining the opinion from the Certified Review Board, any additional information that is required should be promptly communicated.

(2) Research institution administrator:

The administrator will receive reports on all protocol violations occurring at a research institution that is enrolling patients in the study.

(3) Principal Investigator:

The physician who supervises study operations at participating research institutions. If the

Principal Investigator notices an protocol violation, he/she should promptly report it to the research institution administrator and notify the Representative Investigator accordingly. In addition, the Principal Investigator is responsible for preparing and appropriately reporting, maintaining, and storing detailed records, including EDC, etc., regarding all protocol violations occurring at the research institution.

(4) Subinvestigator:
The physician who performs study operations at the research institutions. If the Subinvestigator notices a protocol violation, he/she should promptly report it to the Principal Investigator.

(5) Research office:
The office organized by the Representative Investigator. It is responsible for the operations for research administrative procedures in general, including those related to protocol violation reporting at the Department of Cardiovascular Medicine, Niigata University Graduate School of Medical and Dental Sciences.

(6) Datacenter:
Center for Clinical Research and Innovation (CCRI), Osaka City University Hospital
The division in charge of data management for the study. It will collect information on protocol violations from data entered into the study EDC.

(7) Data and Safety Monitoring Board:
The board will monitor and evaluate the progress of the study and safety data, etc., and, as a third party, assess the severity of the protocol violation and inform the Representative Investigator of its opinion.

4. Reportable protocol violations

Reportable protocol violations

All protocol violations that occur during the study between informed consent and Week 24 (or the time of discontinuation) are reportable.

Timing of protocol violation reporting

Principal Investigators and Subinvestigators (hereafter called “Principal Investigators, etc.”) should promptly report any protocol violation when they notice one, including not only protocol violations discovered by their own inspections but also those discovered by monitoring and auditing.

5. Procedures for reporting protocol violations

Regarding recognized protocol violations associated with the study, the Principal Investigators, etc., should report detailed records by creating them in EDC, etc., and should maintain and store them. Reporting procedures for protocol violations specified in the Clinical Trials Act and for those specified in the study protocol are shown below.

(1) Routine report (Figure 1)
1) If Principal Investigators, etc., notice any protocol violation at the research institution, they

should promptly report it to the Representative Investigator and the research institution administrator.

■ **Principal Investigators** should report any protocol violation they notice to the research institution administrator and should notify the Representative Investigator of it by using EDC.
■ **Subinvestigators** should promptly report any protocol violation they notice to the Principal Investigator; however, if they are concerned that reporting it to the Principal Investigator might preclude appropriate reporting, they should report it directly to the Representative Investigator and the head of the research institution. EDC should be used for reporting the protocol violation to the Representative Investigator.

2) The Representative Investigator should make the final decision about the degree of protocol violation (serious or nonserious) after obtaining the required opinion from the Data and Safety Monitoring Board.

3) The Representative Investigator should promptly report the protocol violation to the Certified Review Board by using the designated form, especially if, in the opinion of the Data and Safety Monitoring Board, the protocol violation is serious. If a protocol violation is not deemed serious, a periodic report should be sent to the Certified Review Board by using the designated form.

4) If the Representative Investigator receives a report from a Principal Investigator and a Subinvestigator that a protocol violation is serious or determines himself that the reported protocol violation is serious, the investigator should promptly report it to the other Principal Investigators.

5) After obtaining the opinion of the Certified Review Board, the Representative Investigator should prepare a summary of the serious protocol violation and develop measures to prevent its recurrence.

6) The Representative Investigator should send the summary of the serious protocol violation and the measures to prevent its recurrence to all physicians involved in the study.

(Figure 1)

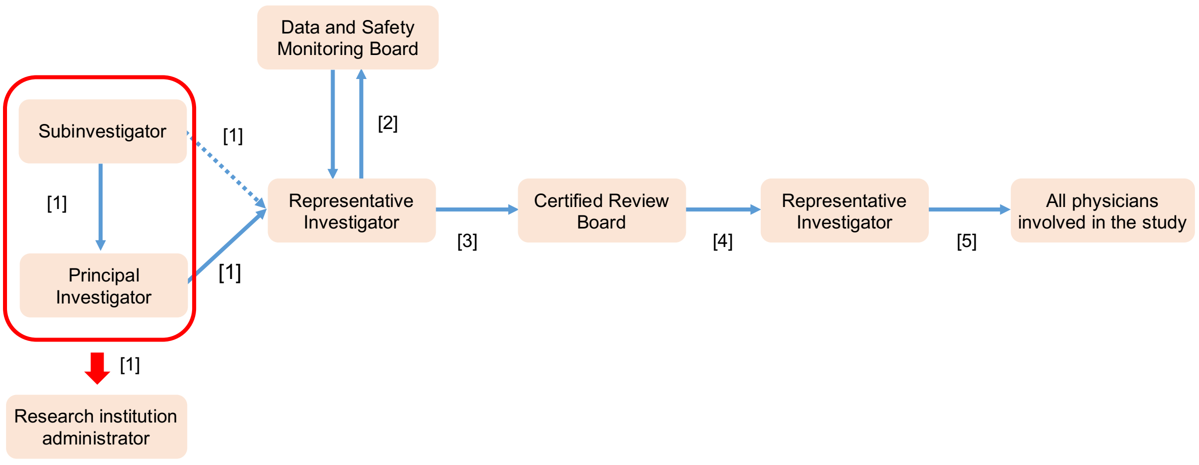

(2) Periodic report

According to Article 59, Enforcement Regulations of Clinical Trials Act, the Representative Investigator should periodically report the occurrence of and response to a study-related protocol violation in accordance with the Enforcement Regulations or the protocol. Periodic reporting should be done within 2 months of the relevant surveillance period, which as a matter of principle is at the end of every year after the date when the protocol was submitted to the Minister of Health, Labour and Welfare.

In addition, in accordance with Article 60, Enforcement Regulations, Clinical Trials Act, if any protocol violation is reported to the MHLW, the MHLW should be asked for confirmation of receipt every time.

Types of report form

1) Routine report

Serious protocol violation

The Representative Investigator should use the serious protocol violation report “Unified Form 7” to report a serious protocol violation to the Certified Review Board.

* Use the updated unified form specified in the “Regarding Unified Forms for the Clinical Trials Act” (Administrative communication of the Research and Development Division, Health Policy Bureau, MHLW).

Summary of and measures to prevent recurrence of protocol violation

The Representative Investigator should communicate the protocol violation to Principal Investigators, Subinvestigators, and other people involved in the clinical study and ensure that recurrence is prevented.

2) Periodic report

Form for reporting to the Certified Review Board: Periodic report “Attachment Form 3”

* Use the updated form specified in “Regarding regulations for enforcement of the Clinical Trials Act.”

6. Where to report protocol violations

Principal Investigators and Subinvestigators should report protocol violations by using EDC. If any problem occurs during reporting, contact the research office.

Contact for Public Queries	Name of the person in charge	Shinya Fujiki
	Affiliation of the person in charge	Niigata University Graduate School of Medical and Dental Sciences
	Department of the person in charge	Department of Cardiovascular Medicine
	Postal code of the institution of the person in charge	951-8510
	Address of the institution of the person in charge	1-757 Asahimachidori, Chuo-ku, Niigata 951-8510, Japan
	Phone no.	025-227-2185
	Fax no.	025-227-0774
	E-mail address	shinya_fukuji@yahoo.co.jp

7. Storage of materials

Store materials related to protocol violations for the period specified in the protocol.

8. History of changes

Version	Description of change
1.0	Initial preparation
1.1	Modification related to enforcement of the Clinical Trials Act
1.2	Clarification of operations, maintenance of descriptions<br>Modification to reflect the amendment of regulations for enforcement of the Clinical Trials Act

Comparison of empagliflozin or placebo for prevention of lethal ventricular arrhythmia in type 2 diabetic patients with implantable cardioverter defibrillators (ICD)

Monitoring procedures

Representative Investigator
Tohru Minamino, Part-time Lecturer, Department of Cardiovascular Medicine,
Niigata University Graduate School of Medical and Dental Sciences
Address: 1-757 Asahimachidori, Chuo-ku, Niigata 951-8510, Japan
Tel no.: 025-227-2185
Fax no.: 025-227-0774

Version 1.3 prepared on July 10, 2020

1 Purpose and scope of the document

This document describes the procedures and other matters that are necessary so that monitors can appropriately conduct monitoring of the "Comparison of empagliflozin or placebo for prevention of lethal ventricular arrhythmia in type 2 diabetic patients with implantable cardioverter defibrillators" (hereafter called the "study") for the Representative Investigator.

2 Implementation system and responsibilities

2.1 Responsibilities of the Representative Investigator

(1) The Representative Investigator should arrange for monitors to conduct monitoring to ensure the proper conduct of the study from the standpoint of ensuring reliability of the study and protecting participants and to examine the progress of the study and whether the study is being conducted in accordance with the Clinical Trials Act and the protocol.

(2) The Representative Investigator should provide the necessary instruction and management for monitoring by monitors.

2.2 Monitor responsibilities

(1) Monitors should confirm their commitment to protecting human rights and ensuring the safety of participants and ensuring appropriate conduct of the study in accordance with the Clinical Trials Act, updated Trial Plan, and the protocol and should confirm that written consent to participate in the study has been obtained from all participants. In addition, they should confirm that records are accurate by using medical records and procedure documents as source documents for case report forms.

(2) If monitors find any matter or protocol violation that is likely to affect the appropriate conduct of the study or if they find any deviation from the protocol, they should promptly notify the Principal Investigator of the matter and take appropriate action to prevent its recurrence.

(3) Each time they conduct monitoring, monitors should prepare a monitoring report that summarizes important matters they discovered or facts about a disease or the like, protocol violation, etc., and submit it to the Principal Investigator. The monitoring report should include the following items:

[1] Date of monitoring
[2] Name of monitor
[3] Name of the Principal Investigator, Subinvestigator, and research assistant from whom monitors heard explanations during monitoring.
[4] Summary of monitoring results (including the summary and important matters discovered or facts about inspected items, deviations, and defects, and conclusions)
[5] Matters reported to the Representative Investigator as specified in "2. 2 (2)."
[6] The action to be taken on the matters specified in [5], and the monitor's findings on such

action.

2.3 Monitor requirements

Monitors should have intimate knowledge of the Clinical Trials Act, Trial Plan, protocol, and informed consent form and procedures, should understand the ethical principles for research* and should have the scientific and clinical knowledge necessary for monitoring. They should also have received training in all of the following:

(1) General business manner

(2) Basic knowledge of natural science, including medicine, pharmacy, nursing, and laboratory medicine

(3) Clinical Trials Act

(4) The investigational product, Trial Plan, and protocol, informed consent form and other research-related materials, standard operating procedures, etc.

* Declaration of Helsinki - Ethical Principles for Medical Research Involving Human Subjects (World Medical Association)
Clinical Trials Act, laws and regulations for protection of personal information

3 Procedures for conducting monitoring

3.1 Appointment of monitors

The Representative Investigator should appoint study monitors (Form 1) after confirming that they meet “2.3 Monitor requirements” based on their resume, education history, etc. Monitors appointed by the Representative Investigator must not be involved in the study.

3.2 Advance preparation for monitoring

3.2.1 Identification of source documents

Wherever possible, at each research institution monitors should confirm the source documents with the Principal Investigator before informed consent is obtained.

3.3 Procedures for monitoring

The study should be monitored by on-site monitoring and remote monitoring.

3.3.1 Monitoring methods

3.3.1.1 On-site monitoring

On-site monitoring refers to the monitoring conducted by monitors when they visit research institutions.

3.3.1.2 Remote monitoring

Remote monitoring refers to the monitoring conducted by monitors without visiting the research

institutions; in this type of monitoring, monitors extract and use the data entered into electronic case report forms. In principle, remote monitoring includes confirming data with Principal Investigators or Subinvestigators and may include confirming data with other research assistants.

3.4 Monitoring items

3.4.1 On-site monitoring

On-site monitoring should be conducted at the first 10 institutions that enroll participants. The monitors should use the source documents to confirm that informed consent was obtained from all patients who have been enrolled in the study at the time of monitoring. In addition, the eligibility of 1 to 3 random patients should be confirmed by reviewing the source documents. If there is any problem with confirming eligibility, on-site monitoring should be repeated at the respective research institution.

3.4.2 Remote monitoring

1) Items to be monitored
   - The presence or absence of the description of concomitant medication
   - Status of the implementation of test and observation items (presence or absence of deviations)
   - Appropriateness of entered data
2) Timing
   - First time: To be conducted after on-site monitoring when 50% of expected participants have been enrolled
   - Second time: To be conducted when all participants have been enrolled

3.5 Description of on-site monitoring

3.5.1 Informed consent

- Confirm source documents, such as informed consent forms of patients who consented to participate in the study, and that voluntary written consent to participate in the study was obtained from participants. In addition, confirm that any study procedure was not initiated before informed consent.
- Confirm that informed consent was obtained by using the patient information sheet approved by the Certified Review Board.

3.5.2 Eligibility

Confirm eligibility by verifying source documents regarding the following items:

- Allocation factors
- Inclusion and exclusion criteria
- Use of contraindicated drugs

3.5.3 Participant safety

- Confirm the presence or absence of adverse events. If any adverse event occurs, confirm the name of the adverse event, date of onset, severity, treatment, causal relationship, and subsequent course and validate the continuation of the study. In addition, if no follow-up has been performed, ask that the status of the participant be checked.
- If any serious adverse event occurs, confirm that the procedures specified in the protocol have been performed. In addition, confirm consistency of the report on the serious adverse event.
- If any adverse event occurs that is possibly related to the study (disease or the like), confirm that the procedures specified in the protocol have been performed.
- Confirm whether or not any participant discontinued the study. If any participant discontinued the study, confirm the date of study discontinuation, reason for the discontinuation, and, in the case of the study discontinuation because of an adverse event, outcome and subsequent course. If the outcome and subsequent course have not been reviewed, ask that they be reviewed.

4 Monitoring report

Monitors should prepare a monitoring report within 15 business days after conducting monitoring and submit it to the Principal Investigator at the monitored site. If necessary, the Principal Investigator should notify the Representative Investigator of the content of the monitoring report. In this case, the Representative Investigator should inform other Principal Investigators of the content of the notification.

5 Quality assessment

If monitors discover any protocol violation as a result of monitoring, they should discuss the cause of the protocol violation with the Principal Investigator and, if necessary, with the Representative Investigator and should take actions to prevent it reoccurring.

6 Confidentiality

Monitors must not divulge participants' secret, identity-related information and other personal information obtained during monitoring.

7 Storage of materials

Principal Investigators and the Representative Investigator should store the monitoring report and other records of monitoring operations that were submitted by monitors.

8 History of preparations and amendments

Version	Date of preparation and amendment	Reason for and description of preparation and amendment

1.0	November 27, 2017	Initial preparation
1.1	August 31, 2018	Maintenance of descriptions
1.2	July 24, 2019	Revision of monitoring plan to reflect addition of research institution
1.3	July 10, 2020	Revision of monitoring plan<br>Maintenance of descriptions

Form 1

MM/DD/YYYY

Appointment of Monitors

Representative Investigator

Tohru Minamino, Part-time Lecturer
Department of Cardiovascular Medicine
Niigata University Graduate School of
Medical and Dental Sciences

I hereby confirm that the following persons have the scientific and clinical knowledge necessary for monitoring and have received the necessary training, and I appoint them as monitors for the study.

Comparison of empagliflozin or placebo for prevention of lethal ventricular arrhythmia in type 2 diabetic patients with implantable cardioverter defibrillators (ICD)

Affiliation	Name

Comparison of empagliflozin or placebo for prevention of lethal ventricular arrhythmia in type 2 diabetic patients with implantable cardioverter defibrillators (ICD) [EMPA-ICD]

Audit procedures

Representative Investigator: Tohru Minamino,
Part-time Lecturer, Department of Cardiovascular Medicine,
Niigata University Graduate School of Medical and Dental Sciences

1 Purpose and scope

This document describes the procedures and other necessary matters so that the Representative Investigator can arrange for responsible auditors and other auditors (hereafter called “auditors”) to appropriately perform an audit to examine whether the study is being conducted in accordance with the Clinical Trials Act and the protocol. Auditing should ensure the reliability of materials collected from the study from the perspective of ensuring reliability of the study and protecting participants in the “Comparison of empagliflozin or placebo for prevention of lethal ventricular arrhythmia in type 2 diabetic patients with implantable cardioverter defibrillators” (hereafter called the “study”).
The following investigators are included in this procedure:
Representative Investigator: Tohru Minamino, Part-time Lecturer, Department of Cardiovascular Medicine, Niigata University Graduate School of Medical and Dental Sciences
Principal Investigator: Responsible person at participating research institutions

2 Implementation system and responsibilities

2.1 Responsibilities of the Representative Investigator

(1) To ensure the quality of the study, the Representative Investigator should appoint auditors and arrange for them to perform an audit for the purpose of determining independently and separately from quality control operations, including usual monitoring, that the study is being conducted in accordance with the Declaration of Helsinki, the Act on Securing Quality, Efficacy and Safety of Products Including Pharmaceuticals and Medical Devices and the Clinical Trials Act (hereafter called Related Laws and Rules); that the study is being appropriately conducted by confirming through source data verification, etc., that the protocol and procedures for the study are being followed; that the materials necessary for the conduct and management of the study are available; and that the reliability of records is fully ensured.

(2) The Representative Investigator should ensure that audits are performed according to this procedure and that the audit plan is based on this procedure.

2.2 Auditor responsibilities

As required, auditors should perform an on-site audit of materials necessary for the conduct and management of the study at research institutions and should confirm that the study is being appropriately conducted through source verification, etc., of data. Instead of on-site source verification of data, they may audit documents by reviewing the provided copies of materials or by remotely reviewing materials via a TV conference system.

2.3 Auditor requirements

(1) Continuously educated and trained in Related Laws and Rules and audit-related matters
(2) Familiar with Related Laws and Rules and the study procedures
(3) Able to evaluate audit results from comprehensive and overall aspects
(4) Able to recognize problems related to clinical research and appropriately decide on analysis of and action to solve problems
(5) Able to appropriately express opinions and provide proper advice and recommendations

based on the review and evaluation of the audit

(6) Able to maintain a fair and nondiscriminatory attitude

2.4 Appointment of Auditors

The Representative Investigator should appoint auditors for the study (Form 1) after confirming that they meet “2.3. Auditor requirements” by reviewing their resume, education history, etc. Auditors appointed by the Representative Investigator must not be involved in the conduct of the study or monitoring at the research institution being audited.

3 Type of audit

The Representative Investigator should arrange for auditors to perform an audit for clinical research.

4 Audit procedures

4.1 Preparation of the audit plan

Auditors should prepare the audit plan. The Representative Investigator should confirm the content of the audit plan prepared by the auditors.
Matters to be described in the audit plan are specified in Form 2.

4.2 Audit for clinical research

(1) Matters to be audited:

[1] Matters regarding the Representative Investigator, Principal Investigator, etc.
[2] Matters regarding the research institution administrator, etc.
[3] Matters regarding the multicenter study
[4] Matters regarding action and reporting at the onset of a disease or the like
[5] Matters regarding preparation of the protocol
[6] Matters regarding management of protocol violations
[7] Matters regarding monitoring
[8] Matters regarding informed consent and participant compensation
[9] Matters regarding Conflicts of Interest management
[10] Matters regarding reviewing and reporting to the Certified Review Board
[11] Matters regarding response to complaints and queries
[12] Matters regarding the announcement of information
[13] Matters regarding ensuring the quality of pharmaceuticals used in clinical research
[14] Matters regarding the handling of personal information
[15] Matters regarding the Trial Plan
[16] Other matters regarding regulations related to the Clinical Trials Act and the protocol

(2) Timing and frequency of the audit
In principle, the Representative Investigator should arrange for auditors to perform an audit when and as often as considered most appropriate in discussions between the Representative Investigator and the auditors.

(3) Preparation for audit
Before performing an audit, auditors should obtain or access the research institution's standard operating procedures for conducting clinical studies to confirm the institution's procedures for conducting research and accepting audits. Auditors should coordinate in advance the acceptance of the audit and date with the Principal Investigator, etc., and the division to be audited and then ask to perform the audit.

(4) Conduct of audit
Auditors should perform the audit according to the audit plan. The following materials should be audited:
[1] Various standard operating procedures
[2] Study materials
- Certified Review Board records
- Records of approval by the heads of research institutions for the conduct of clinical research
- Materials submitted in advance to the Certified Review Board by the Representative Investigator
- Records on study registration (including jRCT)
- Records on Conflicts of Interest
- Records on means for compensating participants in case of injury
- Records on obtaining informed consent (from all participants)
- Records on the procedures for providing safety information during the study
- Documents created as specified in the protocol and the Clinical Trials Act
- Materials on agreements with the Contract Research Organization (CRO) and Site Management Organization (SMO)
- Other necessary materials

(5) Review of audit results
Auditors should summarize the matters discovered and confirmed by the audit as audit records, evaluate such records and consider problems and necessary action, etc.

(6) Preparation and submission of an audit report
Auditors should prepare an audit report on the basis of the audit records, including matters discovered or confirmed by the audit, and then, after affixing their signature or name and seal, they should submit the audit report to the Principal Investigator. Matters to be included in the audit report are described in Form 3. The Principal Investigator should notify the Representative Investigator of the content of the report, if necessary. The Representative Investigator should inform other Principal Investigators of the content of the notification.

(7) Response to the audit report

If necessary, the Principal Investigator should prepare and submit to the auditors the response to the submitted audit report.
Auditors who receive the response to the audit report should confirm that appropriate measures were taken to remedy problems and that such problems have been remedied. After determining that problems have been remedied, auditors should prepare a response confirmation and submit it to the Principal Investigator to whom the audit report was submitted under (6) and the head of the research institution. Moreover, if auditors determine that the response to the suggestions in the audit is insufficient, they should describe the response as such in the confirmation of response, submit the confirmation of response to the Principal Investigator to whom the audit report was submitted under (6) and the head of the research institution and submit a copy to the Representative Investigator.
Auditors may perform a follow-up audit to confirm that appropriate remedial measures have been taken.

(8) Issuance of audit certificate
After completion of all audits for the study, auditors should send the Representative Investigator an audit certificate that includes the response, response confirmation, and follow-up audit (if applicable), as mentioned under the preceding item.

5 Forms to be used in audit

The following forms are attached and should be used for study audits (the documents other than the attached forms may be used if they describe the same items):
(1) Appointment of Auditors (Form 1)
(2) Resume, training history (Reference form)
(3) Audit Plan (Form 2)
(4) Audit Report (Form 3)
(5) Response to Audit Report (Form 4)
(6) Response Confirmation (Form 5)
(7) Audit Certificate (Form 6)

6 Confidentiality

Auditors will assume the same obligation as the Principal Investigator, etc., to not divulge participants' secret, identity-related information and other personal information obtained during the audit.

7 Storage of materials

The Representative Investigator should store the audit report and other records of operations for the audit submitted by auditors (including the available audit evidence) for the period specified in the protocol.

8 History of preparations and amendments

Version	Date of preparation and amendment	Reason for and description of amendment
1.0	November 27, 2017	Initial preparation
1.1	August 31, 2018	Maintenance of descriptions
1.2	July 10, 2020	Revised to reflect a change in the job title of the Representative Investigator
2.0	July 9, 2021	Revised to reflect changes in audit targets and method

Form 1

MM/DD/YYYY

Appointment of Auditors

Representative Investigator
Tohru Minamino, Part-time Lecturer, Department of Cardiovascular Medicine, Niigata University Graduate School of Medical and Dental Sciences

I hereby confirm that the following persons have the knowledge necessary for performing an audit and have received the necessary education, and I appoint them as auditors for the study.

Comparison of empagliflozin or placebo for prevention of lethal ventricular arrhythmia in type 2 diabetic patients with implantable cardioverter defibrillators (ICD)

Role	Affiliation	Name
Responsible auditor		
Auditor		
Auditor		

Form 2

MM/DD/YYYY

Audit Plan
(Clinical study audit)

Dear Representative Investigator,
Tohru Minamino, Part-time Lecturer, Department of Cardiovascular Medicine,
Niigata University Graduate School of Medical and Dental Sciences

Responsible auditor

____

Clinical study title	Comparison of empagliflozin or placebo for prevention of lethal ventricular arrhythmia in type 2 diabetic patients with implantable cardioverter defibrillators (ICD)
Audit scope (Items and materials to be audited)	
Audit duration and period	
Auditor	
Division to be audited	

Note: The responsible auditor should prepare the required number of original copies of this form and submit one of them to the Representative Investigator.

Form 3

MM/DD/YYYY

Audit Report
(Clinical study audit)

Dear Principal Investigator and head of the research institution,

AND

Representative Investigator,
Tohru Minamino, Part-time Lecturer, Department of Cardiovascular Medicine,
Niigata University Graduate School of Medical and Dental Sciences

Responsible auditor

Seal

Clinical study title	Comparison of empagliflozin or placebo for prevention of lethal ventricular arrhythmia in type 2 diabetic patients with implantable cardioverter defibrillators (ICD)

Audited division	
Type of audit	
Audited scope, materials, and matters	
Audit date	
Reference document for audit	☐ Clinical Trials Act ☐ Protocol ☐ Procedures
Person who responds to the audit	
Auditor	
Audit results [Opinion and proposed improvement]	
Attached materials	
Remark	

Note: The responsible auditor should prepare the required number of original copies of this form and submit one each to the audited Principal Investigator, the head of the research institution, and the Representative Investigator.

Form 4

MM/DD/YYYY

Response to Audit Report

Dear Responsible auditor

Principal Investigator

____

Clinical study title	Comparison of empagliflozin or placebo for prevention of lethal ventricular arrhythmia in type 2 diabetic patients with implantable cardioverter defibrillators (ICD)

Suggested matters	
Response<br><br>[Description, responsible person, and timing of the action taken on suggested matters]	

Form 5

MM/DD/YYYY

Response Confirmation

Dear Principal Investigator and head of the research institution,

AND

Representative Investigator,
Tohru Minamino, Part-time Lecturer, Department of Cardiovascular Medicine,
Niigata University Graduate School of Medical and Dental Sciences

Responsible auditor

Clinical study title	Comparison of empagliflozin or placebo for prevention of lethal ventricular arrhythmia in type 2 diabetic patients with implantable cardioverter defibrillators (ICD)

Suggested matters	
Response<br><br>[Description, responsible person, and timing of the action taken on suggested matters]	
Confirmed matters	

Note: The responsible auditor should prepare the required number of original copies of this form and submit one each to the audited Principal Investigator, the head of the research institution, and the Representative Investigator.

Form 6

MM/DD/YYYY

Audit Certificate

Dear Representative Investigator,
Tohru Minamino, Part-time Lecturer, Department of Cardiovascular Medicine,
Niigata University Graduate School of Medical and Dental Sciences

Responsible auditor

______________________ Seal

I hereby certify that the audit has been performed for the following clinical study:

Clinical study title	Comparison of empagliflozin or placebo for prevention of lethal ventricular arrhythmia in type 2 diabetic patients with implantable cardioverter defibrillators (ICD)

Audited division	
Type of audit	
Audited scope, materials, and matters	
Audit date	
Auditor	
Date when the audit report was submitted and the person to whom it was submitted	

Note: The responsible auditor should prepare the required number of original copies of this form and submit one to the Representative Investigator.

Study management

1. Research organization

This study is an investigator-initiated study and will be conducted by a research organization consisting of the Representative Investigator; the study adviser; the person in charge of supporting research and development programs; the person who coordinates and manages research; the person who, in addition to the Representative Investigator and Principal Investigators, supervises research; a steering committee; a Data and Safety Monitoring Board; an event assessment committee; a statistical analysis body; a research office; a project management body; a data management body; a monitoring body; an audit body; central measurement bodies; the people responsible for tests; a study drug management body; a study drug allocation body; and the research institutions (Principal Investigators), etc.

(1) Principle Investigator

This investigator is responsible for planning, conducting, analyzing, and disseminating the results of the study and will supervise the overall study.

Tohru Minamino, Professor and Chairman, Department of Cardiovascular Biology and Medicine, Juntendo University Graduate School of Medicine, Tokyo , Japan

Part-time Lecturer, Department of Cardiovascular Medicine, Niigata University Graduate School of Medical and Dental Sciences, Niigata, Japan

(2) Study adviser

This adviser will advise on the conduct and analysis of the study and the dissemination of its results.

Yoshifusa Aizawa, Department of Research and Development, Tachikawa General Hospital, Tachikawa Medical Center

(3) Person in charge of supporting research and development programs

This person will clarify the direction of the overall study and support the efficient planning and operation of a series of processes, including conception, strategy development, dissemination of results (including practical application), and optimization of the necessary multiple clinical studies and basic research, etc. By critically evaluating the protocol (or development strategy) in terms of clinical pharmacology (especially drug efficacy evaluation and research ethics), general clinical practice or the laws and regulations related to clinical research, this person will also support the basic framework of the most effective and efficient (optimized) protocol for the clinical development program.

Koichi Node, Professor, Cardiovascular Medicine, Saga Medical School Faculty of Medicine, Saga University

(4) Person who coordinates and manages research

This person will ensure smooth coordination and management of the study by applying knowledge and approaches for planned and efficient operational management of clinical research.

Shinya Fujiki, Department of Cardiovascular Medicine, Niigata University Graduate School of

Medical and Dental Sciences

(5) Person who, in addition to the Representative Investigator and Principal Investigators, supervises research

The owner of patent rights on pharmaceuticals to be used in the study or the person who procures research funds for the study. This person will supervise the study.

Shinya Fujiki, Department of Cardiovascular Medicine, Niigata University Graduate School of Medical and Dental Sciences

(6) Steering committee

This committee will plan, conduct, analyze and announce the study, recognize problems during conduct of the study, discuss solutions and coordinate any actions required for study operations.

Chairperson	Toyoaki Murohara, Professor, Cardiovascular Medicine, Nagoya University
Members (in random order)	Toshihisa Anzai, Professor, Cardiovascular Medicine, Hokkaido University
	Kenji Ando, Medical Director, Cardiovascular Medicine, Kokura Kinen Hospital
	Junichi Nitta, Senior Director, Cardiovascular Medicine, Sakakibara Heart Institute
	Masaaki Okabe, Director of the hospital, Tachikawa General Hospital
	Ritsushi Kato, Professor, Department of Cardiology, Saitama Medical University International Medical Center
	Kazuomi Kario, Professor, Cardiovascular Medicine, Jichi Medical University
	Kengo Kusano, Director of the Cardiovascular Department, National Cerebral and Cardiovascular Center
	Yusuke Kondo, Specially Appointed Assistant Professor, Cardiovascular Medicine, Chiba University
	Shingo Sasaki, Associate Professor, Cardiology and Nephrology, Hirosaki University
	Yoshiaki Kubota, Assistant Professor and Clinical Fellow, Cardiovascular Medicine, Nippon Medical School
	Morio Shoda, Professor, Cardiovascular Medicine, Tokyo Women's Medical University
	Masaki Ieda, Professor, Cardiovascular Medicine, University of Tsukuba
	Kazuyoshi Takahashi, Director of the Department of Cardiovascular Medicine, Niigata City General Hospital
	Takashi Kaneshiro, Associate Professor, Cardiovascular Medicine, Fukushima Medical University
	Kenichi Tsujita, Professor, Cardiovascular Medicine, Kumamoto University
	Hirofumi Tomita, Professor, Cardiology and Nephrology, Hirosaki University
	Shigeto Naito, Director, Cardiovascular Medicine, Gunma Prefectural Cardiovascular Center
	Shinichi Niwano, Professor, Cardiovascular Medicine, Kitasato University

Morio Shoda Professor (Fixed Term), Cardiovascular Medicine, Tokyo Women's Medical University
Tetsuji Miura, Professor, Department of Cardiovascular, Renal and Metabolic Medicine, Sapporo Medical University
Tomio Umemoto, Cardiovascular Medicine, Jichi Medical University Saitama Medical Center
Takeshi Kato, Assistant Professor, Cardiovascular Medicine, Kanazawa University Hospital
Yoshihisa Nakagawa, Professor, Cardiovascular Medicine, Shiga University of Medical Science Hospital
Koji Maemura, Professor, Cardiovascular Medicine, Nagasaki University Hospital
Hiroshi Tada, Professor, Cardiovascular Medicine, University of Fukui Hospital
Masafumi Watanabe, Professor, First Department of Internal Medicine, Yamagata University Hospital
Masafumi Yano, Professor, Department of Medicine and Clinical Science, Yamaguchi University Hospital
Shingen Owada, Lecturer, Cardiovascular Medicine, Iwate Medical University Hospital
Yugo Yamashita, Program-Specific Assistant Professor, Cardiovascular Medicine, Kyoto University Hospital
Takashi Saigawa, Director of the Department of Cardiovascular Medicine, Niigata Prefectural Central Hospital
Hidemori Hayashi, Associate Professor, Cardiovascular Medicine, Juntendo University Hospital
Takashi Tokano, Associate Professor, Cardiovascular Medicine, Juntendo University Urayasu Hospital

(7) Data and Safety Monitoring Board

This board evaluates safety-related data and will consider the need to amend the protocol and the appropriateness of continuing the study and make respective recommendations to the sponsor.

Chairperson	Naohiko Takahashi, Professor, Cardiovascular Medicine, Oita University
Members (in order of the Japanese syllabary)	Kojiro Ueki, Director of the Diabetes Research Center, Research Institute, National Center for Global Health and Medicine<br>Yohei Ohno, Lecturer, Cardiovascular Medicine, Tokai University<br>Koichiro Kuwahara, Professor, Cardiovascular Medicine, Shinshu University<br>Motoaki Sano, Associate Professor, Cardiovascular Medicine, Keio University

(8) Event assessment committee

This committee will evaluate the data related to each arrhythmia event (an endpoint), consider the appropriateness of continuing the study and make recommendations to the sponsor.

Chairperson Hiroshi Furushima, Director of the Furushima Clinic
Members Hirotaka Sugiura, Director, Cardiovascular Medicine, Niigata Medical Center
Shinsuke Okada, Director, Cardiovascular Medicine, Niigata Medical Center

(9) Statistical analysis body

The body, which is responsible for statistical activities, will be the statistical experts for the study and perform the statistical analysis of the study results.

Responsible person Takahiro Tanaka, Specially Appointed Assistant Professor, Clinical Research Quality Control division, Clinical and Translational Research Center, Niigata University Medical & Dental Hospital

(10) Research office

The research office will perform all administrative operations required for supporting and conducting the study and will support participating research institutions and investigators to ensure that the study progresses smoothly.

Shinya Fujiki, Department of Cardiovascular Medicine, Niigata University Graduate School of Medical and Dental Sciences
Address: 757 Ichibancho, Asahimachi-dori, Chuo-ku, Niigata city, Niigata 951-8510, Japan
Tel: 025-227-2185; Fax: 025-227-0774

Assistant research office
Project Management, Micron, Inc.
Responsible person: Noriko Kawaharada
Address: Mita 43 MT Bldg. 9F, 3-13-16 Mita, Minato-ku, Tokyo, 108-0073, Japan
Tel: 03-6631-3696; Fax: 03-6631-3697

(11) Project management body

This body will support the research office in maintaining the desired quality of the study and ensure that the study progresses efficiently and effectively.

Kenichi Iijima, Associate Professor, Department of Cardiovascular Biology and Medicine, Juntendo University Graduate School of Medicine
Address: 3-1-3 Hongo, Bunkyo City, Tokyo 113-8421, Japan
Tel: 03-3813-3111; Fax: 03-5689-0627

(12) Data management body

This body will determine the order of data cleaning and fixing for enrolled participants and perform data management operations.

Department of Medical Statistics, Osaka City University Graduate School of Medicine and Faculty

of Medicine
Responsible person: Kanae Takahashi
Address: Asahi-machi 1-5-7, Abeno-ku, Osaka-City, Osaka 545-8586, Japan
Tel: 06-6645-3894

Data center
Center for Clinical Research and Innovation (CCRI), Osaka City University Hospital
Responsible person: Keiko Ota
Address: ABENO MEDIX 6F, 1-2-7 Asahi-machi, Abeno-ku, Osaka-City, Osaka 545-0051, Japan
Tel: 06-6645-3470

(13) Monitoring body

This body will perform monitoring operations to confirm appropriate conduct of the study and the reliability, etc., of the data.

Micron, Inc.: Monitoring
Responsible person: Noriko Kawarada
Address: Mita 43 MT Bldg. 9F, 3-13-16 Mita, Minato-ku, Tokyo, 108-0073, Japan
Tel: 03-6631-3696; Fax: 03-6631-3697

(14) Audit body

This body will perform audit operations as required to ensure the reliability of the study results.

Micron, Inc.: Reliability Assurance
Responsible person: Akiko Shibuya
Address: Mita 43 MT Bldg. 9F, 3-13-16 Mita, Minato-ku, Tokyo, 108-0073, Japan
Tel: 03-6631-3696; Fax: 03-6631-3697

(15) Central measurement bodies

These bodies will receive samples from study sites, perform the measurements described below and prepare a report of the respective results.

Measurement of blood ketone body fraction, blood catecholamine concentration, erythropoietin, and reticulocytes:
SRL, Inc.
Address: 2-1-1 Nishishinjuku, Shinjuku-ku, Tokyo 163-0409, Japan
Tel: 03-6279-0900 (main no.)

Measurement of telomere length and P53, P21, and P16:
Cardiovascular Medicine, Graduate School of Medicine of Juntendo University
Address: 2-1-1 Hongo, Bunkyo-ku, Tokyo 113-8421, Japan
Tel: 03-5802-1054; Fax: 03-5689-0627

Metabolome analysis:
Institute for Advanced Biosciences, Keio University
Address: 246-2, Mizukami, Kakuganji, Tsuruoka, Yamagata 997-0052, Japan
Tel: 0235-29-0528; Fax: 0235-29-0574

(16) People responsible for tests

These people will define the procedures for various tests and ensure that the quality of the tests is maintained.

Holter monitoring:
Kenichi Iijima, Associate Professor, Department of Cardiovascular Biology and Medicine, Juntendo University Graduate School of Medicine
Address: 3-1-3 Hongo, Bunkyo City, Tokyo 113-8421, Japan
Tel: 03-3813-3111; Fax: 03-5689-0627

Echocardiography:
Takeshi Okubo, Department of Cardiovascular Medicine, Niigata University Graduate School of Medical and Dental Sciences
Address: 757 Ichibancho, Asahimachi-dori, Chuo-ku, Niigata city, Niigata 951-8510, Japan
Tel: 025-227-2185; Fax: 025-227-0774

^{123}I-MIBG myocardial scintigraphy:
Yosuke Horii, Assistant Professor, Department of Radiology and Radiation Oncology, Niigata University Graduate School of Medical and Dental Sciences
Address: 757 Ichibancho, Asahimachi-dori, Chuo-ku, Niigata city, Niigata 951-8510, Japan
Tel: 025-227-2185; Fax: 025-227-0774

(17) Study drug management body

Implementation Management division, Clinical and Translational Research Center, Niigata University Medical & Dental Hospital
Responsible person: Hiroyasu Sasahara
Address: 2-5274 Gakkocho-Dori, Chuo-ku, Niigata 951-8514, Japan

Tel: 025-223-6161

(18) Study drug allocation body

Implementation Management division, Clinical and Translational Research Center, Niigata University Medical & Dental Hospital
Responsible person: Hiroyasu Sasahara
Address: 2-5274 Gakkocho-Dori, Chuo-ku, Niigata 951-8514, Japan
Tel: 025-223-6161

Person responsible for study drug allocation
Department of Medical Statistics, Osaka City University Graduate School of Medicine and Faculty of Medicine
Responsible person: Hisako Yoshida
Address: Asahi-machi 1-5-7, Abeno-ku, Osaka-City, Osaka 545-8586, Japan
Tel: 06-6645-3894

(19) Study contact person

EMPA-ICD Assistant research office
Project Management, Micron, Inc.
Noriko Kawaharada
Address: Mita 43 MT Bldg. 9F, 3-13-16 Mita, Minato-ku, Tokyo, 108-0073, Japan
Tel: 03-6631-3696; Fax: 03-6631-3697

(20) Research Funder

Nippon Boehringer Ingelheim Co., Ltd
Address: ThinkPark Tower, 2-1-1 Osaki, Shinagawa-ku, Tokyo 141-6017, Japan
Tel: 03-6417-2958; Fax: 03-5435-2981
(*Some of the research expenses will be borne by Eli Lilly and Company [US].)

2. Research institutions expected to participate (in order of the Japanese syllabary)

Institution	Affiliation	Principal Investigator	Address	Tel no. (main)
Iwate Medical University Hospital	Cardiovascular Medicine	Shingen Owada	2-1-1 Idaidori, Yahaba-cho, Shiwa-gun, Iwate 028-3695	019-613-7111
Tachikawa General Hospital, Tachikawa Medical Center	Cardiovascular Medicine	Masaaki Okabe	Asahioka 1-24, Nagaoka-shi, Niigata, 940-8621	0258-33-3111
Kitasato University Hospital	Cardiovascular Medicine	Shinichi Niwano	1-15-1, Kitazato, Minami, Sagamihara, Kanagawa, 252-0375	042-778-8111
Kyoto University Hospital	Cardiovascular Medicine	Yugo Yamashita	54 Shogoin-Kawahara-cho, Sakyo-ku, Kyoto 606-8507	075-751-3111
Kumamoto University Hospital	Cardiovascular Medicine	Kenichi Tsujita	1-1-1 Honjo, Chuo-ku, Kumamoto City, Kumamoto 860-8556	096-344-2111
Gunma Prefectural Cardiovascular Center	Cardiovascular Medicine	Shigeto Naito	3-12, Kameizumi-town, Maebashi-city, Gunma 371-0004	027-269-7455
Kokura Kinen Hospital	Cardiovascular Medicine	Kenji Ando	3-2-1 Asano Kokurakita-Ku, Kitakyushu, Fukuoka 802-8555	093-511-2000
National Cerebral and Cardiovascular Center	Cardiovascular Department	Kengo Kusano	6-1 Kishibe-Shimmachi, Suita, Osaka, 564-8565	06-6170-1070
Saitama Medical University International Medical Center	Department of Cardiology	Ritsushi Kato	1397-1, Yamane, Hidaka-City, Saitama 350-1298	042-984-4111
Sakakibara Heart Institute	Cardiovascular Medicine	Junichi Nitta	3-16-1 Asahi-cho, Fuchu, Tokyo, 183-0003	042-314-3111
Sapporo Medical University Hospital	Department of Cardiovascular, Renal and Metabolic Medicine	Tetsuji Miura	16-291, Minami-ichijo-nishi, Chuo-ku, Sapporo 060-8543	011-611-2111

Jichi Medical University Saitama Medical Center	Cardiovascular Medicine	Tomio Umemoto	1-847 Amanuma-cho, Omiya-ku, Saitama 330-8503	048-647-2111
Jichi Medical University Hospital	Cardiovascular Medicine	Kazuomi Kario	3311-1 Yakushiji, Shimotsuke-shi, Tochigi 329-0498	0285-58-7344
Juntendo University Urayasu Hospital	Cardiovascular Medicine	Takashi Tokano	2-1-1 Tomioka, Urayasu-shi, Chiba 279-0021	047-353-3111
Juntendo University Hospital	Cardiovascular Medicine	Hidemori Hayashi	3-1-3 Hongo, Bunkyo-ku, Tokyo 113-8431	03-3813-3111
Chiba University Hospital	Cardiovascular Medicine	Yusuke Kondo	1-8-1 Inohana, Chuo-ku, Chiba-shi, Chiba, 260-8677	043-222-7171
University of Tsukuba Hospital	Cardiovascular Medicine	Masaki Ieda	2-1-1 Amakubo, Tsukuba, Ibaraki 305-8576	029-853-3900
Tokyo Women's Medical University Hospital	Cardiovascular Medicine	Morio Shoda	8-1. Kawada-cho, Shinjuku-ku, Tokyo, 162-8666	03-3353-8111
Nagoya University Hospital	Cardiovascular Medicine	Toyoaki Murohara	65 Tsurumai-cho, Showa-ku, Nagoya 466-8560	052-741-2111
Niigata Prefectural Central Hospital	Cardiovascular Medicine	Takashi Saigawa	205 Shinnan-cho, Joetsu-City, Niigata 943-0192	025-522-7711
Niigata City General Hospital	Cardiovascular Medicine	Kazuyoshi Takahashi	463-7 Shumoku, Chuo-ku, Niigata City, Niigata 950-1197	025-281-5151
Niigata University Graduate School of Medical and Dental Sciences	Cardiovascular Medicine	Tohru Minamino	757 Ichibancho, Asahimachi-dori, Chuo-ku, Niigata city, Niigata 951-8510	025-227-2185
Hirosaki University Hospital	Cardiology and Nephrology	Hirofumi Tomita	53 Honcho, Hirosaki, Aomori 036-8563	0172-33-5111
Fukushima Medical University Hospital	Cardiovascular Medicine	Takashi Kaneshiro	1 Hikarigaoka, Fukushima-city, Fukushima 960-1295	024-547-1111
Hokkaido University Hospital	Department of Cardiovascular Medicine	Toshihisa Anzai	5 Kita14jonishi, Kita-Ku, Sapporo, Hokkaido 060-8648	011-716-1161
Nippon Medical School Hospital	Cardiovascular Medicine	Yoshiaki Kubota	1-1-5 Sendagi, Bunkyo-ku, Tokyo 113-8603	03-3822-2131
Kanazawa University Hospital	Cardiovascular Medicine	Takeshi Kato	13-1, Takaramachi, Kanazawa-shi, Ishikawa 920-8641	076-265-2259
Shiga University of Medical Science Hospital	Cardiovascular Medicine	Yoshihisa Nakagawa	Seta-Tsukinowa-cho, Otsu, Shiga 520-2192	077-548-2111
Nagasaki University Hospital	Cardiovascular Medicine	Koji Maemura	1-chōme-7-1 Sakamoto, Nagasaki, 852-8501	095-819-7200

University of Fukui Hospital	Cardiovascular Medicine	Hiroshi Tada	23-3 Matsuoka-shimoaizuki, Eiheiji-cho, Yoshida-gun, Fukui 910-1193	0776-61-8800
Yamagata University Hospital	First Department of Internal Medicine	Masafumi Watanabe	2-2-2 Iida-Nishi, Yamagata-city, Yamagata, 990-9585	023-628-5302
Yamaguchi University Hospital	Department of Medicine and Clinical Science	Masafumi Yano	1-1-1 Minami-Kogushi, Ube, Yamaguchi 755-8505	0836-22-2111

Version No. before revision	Version No. after revision	Page No. before revision	Page No. after revision	Before revision	After revision	Reason for revision
1.3	1.4	52	52	16-2. Conflict of interest (COI)<br>Study-Representing Physician Tohru Minamino, Department of Cardiovascular Medicine, Niigata University Graduate School of Medical and Dental Sciences<br>COI with Nippon Boehringer Ingelheim Co., Ltd: Personal interest relationship over annual 1 million yen	Version 1.4<br>16-2. Conflict of interest (COI)<br>1. Study-Representing Physician Tohru Minamino, Department of Cardiovascular Medicine, Niigata University Graduate School of Medical and Dental Sciences<br>COI with Nippon Boehringer Ingelheim Co., Ltd: Personal interest relationship over annual 1 million yen<br>2. Investigator Kengo Kusano, Cardiovascular Department, National Cerebral and Cardiovascular Center Hospital<br>COI with Nippon Boehringer Ingelheim Co., Ltd:（A member of his family）Assumption of the position of an officer etc.<br>3. Investigator Tetsuji Miura, Department of Cardiovascular, Renal and Metabolic Medicine, Sapporo Medical University Hospital<br>COI with Nippon Boehringer Ingelheim Co., Ltd:（Himself）Other interest relationship<br>4. Investigator Yoshio Kobayashi, Department of Cardiology, Chiba University Hospital<br>COI with Nippon Boehringer Ingelheim Co., Ltd: Personal interest relationship over annual 1 million yen<br>5. Investigator Nobuhisa Hagiwara, Department of Cardiology, Tokyo Women's Medical University Hospital<br>COI with Nippon Boehringer Ingelheim Co., Ltd: Total donation of 2 million yen or more annually<br>6. Investigator Toyoaki Murohara, Department of Cardiology, Nagoya University Hospital<br>COI with Nippon Boehringer Ingelheim Co., Ltd: [1] Total donation of 2 million yen or more annually [2] Personal interest relationship over annual 1 million yen<br>COI with Eli Lilly Japan K.K.: Personal interest relationship over annual 1 million yen<br>7. Subinvestigator Yasuya Inden, Department of Cardiology, Nagoya University Hospital<br>COI with Nippon Boehringer Ingelheim Co., Ltd: Personal interest relationship over annual 1 million yen	To add disclosure of COI situation under the Clinical Trials Act.
1.4	1.5	44	44	[No. of patients at each institution]<br>Kumamoto University Hospital	[No. of patients at each institution]<br>Kumamoto University Hospital	Change in the name of the institution.
1.4	1.5	44	44	[No. of patients at each institution]<br>--	[No. of patients at each institution]<br>Nippon Medical School Hospital: 10	Addition of institutions.
1.4	1.5	52	52	16-2. Conflict of interest (COI)<br>--	16-2. Conflict of interest (COI)<br>8. Study-Representing Physician Wataru Shimizu, Department of Cardiovascular Medicine, Nippon Medical School Hospital<br>COI with Nippon Boehringer Ingelheim Co., Ltd: Personal interest relationship over annual 1 million yen	Addition of institutions.
1.5	1.6	1	1	Issued: May 10, 2019 Ver. 1.5	Issued: Aug. 5, 2019 Ver. 1.6	Ammendment
1.5	1.6	43	43	13: Target sample size and rationales for setting<br>[Rationales for setting]<br>(in total of 20 institutions: 10 to 15 patients/institution)	13: Target sample size and rationales for setting<br>[Rationales for setting]<br>(in total of 20 institutions: 2 to 15 patients/institution)	To adjust descriptions following the addition of institutions.
1.5	1.6	44	44	[No. of patients at each institution]<br>--	[No. of patients at each institution]<br>Kanazawa University Hospital: 10<br>Shiga University of Medical Science Hospital: 5<br>Nagasaki University Hospital: 3<br>University of Fukui Hospital: 5<br>Yamagata University Hospital: 3<br>Yamaguchi University Hospital: 2	Addition of institutions.
1.6	1.7	1	1	Issued: Aug. 5, 2019 Ver. 1.6	Issued: Dec. 13, 2019 Ver. 1.7	Update to preparation date and version.
1.6	1.7	6	6	Abbreviations and definition of terms<br>--	Abbreviations and definition of terms<br>Abbreviation: ALP Spelled-out terms: alkaline phosphatase	Addition of omitted information.
1.6	1.7	7	7	Abbreviations and definition of terms<br>--	Abbreviations and definition of terms<br>Abbreviation: HR Spelled-out terms: heart rate	Addition of omitted information.
1.6	1.7	30	30	*4: The test results obtained within 12 weeks before the tests at Week 0 can be utilized, even before the consent was obtained. The echocardiographic results at the time of eligibility tests are used for allocation to treatment group.	*4: The test results obtained within 12 weeks before the tests at Week 0 can be utilized, even before the consent was obtained. The echocardiographic results at the time of eligibility tests are used for allocation to treatment group. Eligibility test results obtained within 3 months of informed consent are available even if they are obtained before 12 weeks prior to Week 0 tests.	Clarification of operation.
1.6	1.7	31	31	6) Height, body weight, and body temperature: at the time of eligibility test and Week 0 and 24 (or study discontinuation) (*Optional at Week 12).<br>Height, body weight, and body temperature in the examination room are measured at visit to the hospital (at fasting). At Week 0, 12, and 24 (or study discontinuation), only body weight is measured. Height is measured only at the time of eligibility tests.	6) Height, body weight, and body temperature: at the time of eligibility test and Week 0 and 24 (or study discontinuation) (*Optional at Week 12).<br>Height, body weight, and body temperature in the examination room are measured at visit to the hospital. Height is measured only at the time of eligibility tests.	Correction of errors.
1.6	1.7	40	40	10-4. Report of adverse events<br>1) Expedited (alert) report<br>In this study, serious adverse events and specifically noteworthy adverse events are subjected to expedited report.	10-4. Report of adverse events<br>1) Expedited (alert) report<br>In this study, serious adverse events (including serious diseases or the like) and specifically noteworthy adverse events are subjected to expedited report.	Clarification of operation.

1.6	1.7	40	40	10-4. Report of adverse events<br>1) Expedited (alert) report<br>When onset of relevant adverse event was recognized, Investigator or Subinvestigator immediately fills out Adverse Event Report Form as predetermined, transmits to Study Secretariat by FAX at 03-6262-2815, to input through EDC.	10-4. Report of adverse events<br>1) Expedited (alert) report<br>When onset of relevant adverse event was recognized, Investigator or Subinvestigator immediately fills out Adverse Event Report Form as predetermined, reports to Study Secretariat by FAX or email, to input through EDC.	Clarification of operation.
1.6	1.7	40	40	10-4. Report of adverse events<br>4) Report to Independent Data Monitoring Committee (IDMC)<br>Upon receiving an expedited report on adverse event, Study Secretariat promptly reports to IDMC in writing, simultaneously asking to review the appropriateness of measures taken for the relevant adverse event.	10-4. Report of adverse events<br>4) Report to Independent Data Monitoring Committee (IDMC)<br>When considered an adverse event to be serious and unable to be ruled out of the causal relationship to the study (serious disease or the like), Study-Representing Physician promptly reports to IDMC in writing, simultaneously asking to review the appropriateness of measures taken for the relevant adverse event.	To reflect that only possibly related SAEs shall be subjected to IDMC review.
1.6	1.7	44	44	[No. of patients at each institution]<br>—	[No. of patients at each institution]<br>Name of institution: Iwate Medical University Hospital<br>No. of patients prospected to be enrolled: 3	Addition of institutions.
1.6	1.7	44	44	[No. of patients at each institution]<br>—	[No. of patients at each institution]<br>Name of institution: Kyoto University Hospital<br>No. of patients prospected to be enrolled: 10	Addition of institutions.
1.6	1.7	53	53	18: Revision of study protocol<br>If considered necessary, Study-Representing Physician consults with Steering Committee to decide revision of the study protocol. The decision is again reviewed at CRB and communicated to Investigator. Then, administrator of the participating medical institution approves the study based on the revised protocol.	18: Revision of study protocol<br>If considered necessary, Study-Representing Physician consults with Steering Committee to decide revision of the study protocol as needed. The decision is reviewed at CRB and communicated to Investigator. Then, administrator of the participating medical institution approves the study based on the revised protocol.	To adjust descriptions because Steering Committee may not be held.
1.6	1.7	54	54	18: Revision of study protocol<br>Using the updated patient information, patients are again asked about their willing whether to continuously participate in the study, to obtain the consent.	18: Revision of study protocol<br>If any onset of the event possibly affecting patient's will to continue to participate in the study leads to revision of the informed consent form, according to the procedures of the protocol 6-2, using the approved updated informed consent form, written consent to continue to participate in the study should be obtained from patients again.	To adjust descriptions according to the procedures of the protocol 6-2.
1.7	1.8	1	1	Study duration planned: From publication date in jRCT till October 2022	Study duration planned: From publication date in jRCT till October 2023	Extension of study duration.
1.7	1.8	1	1	Issued: Dec. 13, 2019 Ver. 1.7	Issued: March 23, 2020 Ver. 1.8	Update to preparation date and version.
1.7	1.8	15	15	1-7. Target sample size and study duration planned<br>2) Treatment observation: From the disclosure date in jRCT till October 2020 (Deadline of enrollment: April 2020)<br>3) Study duration: From the disclosure date in jRCT till October 2022	1-7. Target sample size and study duration planned<br>2) Treatment observation: From the disclosure date in jRCT till October 2021 (Deadline of enrollment: April 2021)<br>3) Study duration: From the disclosure date in jRCT till October 2023	Extension of study duration.
1.7	1.8	23	23	7-3. Target sample size and study duration planned<br>2) Treatment observation: From the disclosure date in jRCT till October 2020 (Deadline of enrollment: April 2020)<br>3) Study duration: From the disclosure date in jRCT till October 2022	7-3. Target sample size and study duration planned<br>2) Treatment observation: From the disclosure date in jRCT till October 2021 (Deadline of enrollment: April 2021)<br>3) Study duration: From the disclosure date in jRCT till October 2023	Extension of study duration.
1.7	1.8	41	41	Investigator or Subinvestigator appropriately records the investigation items predetermined by CRF in examination records or the likes, and stores. In this study, the data are input in EDC to report to Data Center, including no information allowing to identify patients.	Investigator or Subinvestigator appropriately records the investigation items predetermined by CRF in examination records or the likes, and stores. In this study, the data are input in EDC to report to Data Center, including no information allowing to identify patients. If any source document exists and its objectivity is assurable, and medical judgment is not exercised, trial collaborators may transcribe the data from source documents to EDC. Investigator or Subinvestigator will inspect, confirm and then electronically sign the contents of the prepared EDC. No personal information to identify patients will be included in such data.	To clarify that trial collaborators may transcribe the data from source documents to EDC.
1.7	1.8	52	52	16-2. Conflict of interest (COI)<br>2. Investigator Kengo Kusano, Cardiovascular Department, National Cerebral and Cardiovascular Center Hospital<br>COI with Nippon Boehringer Ingelheim Co., Ltd: (A member of his family) Assumption of the position of an officer etc.	16-2. Conflict of interest (COI)<br>Deleted the underlined portion.<br>Subsequent items were re-numbered.	Correction
1.8	2.0	1	1	Issued: March 23, 2020 Ver. 1.8	Issued: June 12, 2020 Ver. 2.0	Update to preparation date and version.
1.8	2.0	9	9	**1: Overview**<br>**1-3. Patients**<br>Type 2 diabetes-complicated patients（HbA1c ≥ 6.5%）with arrhythmia treatment device (ICD/CRT-D) implanted	**1: Overview**<br>**1-3. Patients**<br>Type 2 diabetes-complicated patients with arrhythmia treatment device (ICD/CRT-D) implanted	Deletion because of the inclusion criteria being relaxed.
1.8	2.0	9	9	**1: Overview**<br>**1-3-1. Inclusion criteria**<br>3) Patients diagnosed with type 2 diabetes whom Investigator or Subinvestigator judged as possible to be administered empagliflozin (HbA1c should be 6.5 to 10% at the time of eligibility tests, regardless whether drugs other than SGLT2 inhibitor are used to treat diabetes).	**1: Overview**<br>**1-3-1. Inclusion criteria**<br>3) Patients diagnosed with type 2 diabetes whom Investigator or Subinvestigator judged as possible to be administered empagliflozin (regardless whether drugs other than SGLT2 inhibitor are used to treat diabetes).	Deletion because of the inclusion criteria being relaxed.
1.8	2.0	18	18	**5: Patients**<br>5-1. Patients<br>Type 2 diabetes-complicated patients (HbA1c ³ 6.5%) with arrhythmia device (ICD/CRT-D) implanted.	**5: Patients**<br>5-1. Patients<br>Type 2 diabetes-complicated patients with arrhythmia treatment device (ICD/CRT-D) implanted.	Deletion because of the inclusion criteria being relaxed.
1.8	2.0	18	18	**5: Patients**<br>5-2. Inclusion criteria<br>3) Patients diagnosed with type 2 diabetes whom Investigator or Subinvestigator judged as possible to be administered empagliflozin (HbA1c should be between 6.5 and 10% at the time of eligibility tests, regardless whether drugs other than SGLT2 inhibitor are used to treat diabetes).	**5: Patients**<br>5-2. Inclusion criteria<br>3) Patients diagnosed with type 2 diabetes whom Investigator or Subinvestigator judged as possible to be administered empagliflozin (regardless whether drugs other than SGLT2 inhibitor are used to treat diabetes).	Deletion because of the inclusion criteria being relaxed.
1.8	2.0	30	30	**8: Observation and test items**<br>8-1. Schedule for observation and tests<br>*5: AST, ALT, eGFR, and HbA1c are included in the eligibility tests.	**8: Observation and test items**<br>8-1. Schedule for observation and tests<br>*5: AST, ALT, and eGFR are included in the eligibility tests.	Deletion because of the inclusion criteria being relaxed.

1.8	2.0	32	32	**8: Observation and test items**<br>8-2. Observation and test items<br>8) Hematological tests: at the time of eligibility tests and Week 0 and 24 (or study discontinuation) (*Optional at Week 12).	**8: Observation and test items**<br>8-2. Observation and test items<br>8) Hematological tests: at the time of eligibility tests (AST, ALT, Cr, and eGRF [estimated values] only) and Week 0 and 24 (or study discontinuation) (*Optional at Week 12).	Addition to adjust descriptions.
1.8	2.0	32	32	**8: Observation and test items**<br>8-2. Observation and test items<br>9) Hematological tests (blood sugar and lipid): at the time of the eligibility tests and Week 0 and 24 (or study discontinuation) (*Optional at Week 12)<br>Blood glucose tests: fasting blood glucose and HbA1c<br>Lipid tests: TG, TC, HDL-C, and LDL-C<br>HbA1c is tested for the eligibility.	**8: Observation and test items**<br>8-2. Observation and test items<br>9) Hematological tests (blood sugar and lipid): at the time of the eligibility tests (only HbA1c) and Week 0 and 24 (or study discontinuation) (*Optional at Week 12)<br>Blood glucose tests: fasting blood glucose and HbA1c<br>Lipid tests: TG, TC, HDL-C, and LDL-C	Correction because of the inclusion criteria being relaxed
2.0	2.1	1	1	Issued: June 12, 2020 Ver. 2.0	Issued: July 10, 2020 Ver. 2.1	Update to preparation date and version.
2.0	2.1	44	44	13: Target sample size and rationales for setting<br>[No. of patients at each institution]<br>--	13: Target sample size and rationales for setting<br>[No. of patients at each institution]<br>Niigata Prefectural Central Hospital: 10<br>Juntendo University Hospital: 10<br>Juntendo University Urayasu Hospital: 10	Addition of institutions.
2.0	2.1	19	19	**17. Contact for the study**<br>Study-Representing Physician<br>Tohru Minamino, Professor, Department of Cardiovascular Medicine, Niigata University Graduate School of Medical and Dental Sciences	**17. Contact for the study**<br>Study-Representing Physician<br>Tohru Minamino, Part-time Instructor, Department of Cardiovascular Medicine, Niigata University Graduate School of Medical and Dental Sciences	Change in the job title.
2.1	2.2	1	1	Issued: July 10, 2020 Ver. 2.1	Issued: July 9, 2021 Ver. 2.2	Update to preparation date and version.
2.1	2.2	47	47	15-4. Monitoring and audit<br>In reality, the appropriate conduct of this study adhering to the protocol is confirmed by on-site inspection, e-mail, and telephone.	15-4. Monitoring and audit<br>In reality, the appropriate conduct of this study adhering to the protocol is confirmed by on-site inspection, e-mail, and telephone. Moreover, instead of on-site source data verification for materials, the confirmation of provided copies of materials and the confirmation of materials using TV conference system will be accepted.	Changes in audit subjects and methods.
2.1	2.2	53	53	16-2. Conflict of interest (COI)<br>7. Study-Representing Physician Wataru Shimizu, Department of Cardiovascular Medicine, Nippon Medical School Hospital<br>COI with Nippon Boehringer Ingelheim Co., Ltd: Personal interest relationship over annual 1 million yen	16-2. Conflict of interest (COI)<br>7. Subinvestigator Wataru Shimizu, Department of Cardiovascular Medicine, Nippon Medical School Hospital<br>COI with Nippon Boehringer Ingelheim Co., Ltd: Personal interest relationship over annual 1 million yen	Change in study administrative structure.
2.2	2.3	1	1	Issued: July 9, 2021 Ver. 2.2	Issued: Oct. 8, 2021 Ver. 2.3	Update to preparation date and version.
2.2	2.3	13	13	1-5-3. Exploratory evaluation endpoints<br>12) DNA tests: Telomere length and G-tail length	1-5-3. Exploratory evaluation endpoints<br>12) DNA tests: Telomere length	Change in an endpoint.
2.2	2.3	30	30	8-1. Schedule for observation and tests<br>*7: Special blood sample volume is as follows: (snip) 10 mL for DNA tests (telomere length and G-tail length); 5 mL for RNA tests (P53, P21, and P16); and 5 mL for metabolome analysis.<br>*8: Some of blood samplings for DNA tests (telomere length and G-tail length), RNA tests (P53, P21, and P16), and metabolome analysis, and 123I-MIBG myocardial scintigraphy are optional.	8-1. Schedule for observation and tests<br>*7: Special blood sample volume is as follows: (snip) 10 mL for DNA tests (telomere length); 5 mL for RNA tests (P53, P21, and P16); and 5 mL for metabolome analysis.<br>*8: Some of blood samplings for DNA tests (telomere length), RNA tests (P53, P21, and P16), and metabolome analysis, and 123I-MIBG myocardial scintigraphy are optional.	Change in an endpoint.
2.2	2.3	32	32	8-2. Observation and test items<br>10) Hematological tests (special): at Week 0 and 24 (or study discontinuation) (*Blood sampling for DNA tests (telomere length and G-tail length), RNA tests (P53, P21, and P16), and metabolome analysis are optional)<br>DNA tests: Telomere length and G-tail length	8-2. Observation and test items<br>10) Hematological tests (special): at Week 0 and 24 (or study discontinuation) (*Blood sampling for DNA tests (telomere length), RNA tests (P53, P21, and P16), and metabolome analysis are optional)<br>DNA tests: Telomere length	Change in an endpoint.
2.2	2.3	37	37	9-3. Exploratory evaluation endpoints<br>12) DNA tests: Telomere length and G-tail length	9-3. Exploratory evaluation endpoints<br>12) DNA tests: Telomere length	Change in an endpoint.
2.2	2.3	47	47	15-2. Handling of data and samples<br>--	15-2. Handling of data and samples<br>As DNA and RNA tests are performed in Cardiovascular Medicine, Graduate School of Medicine of Juntendo University, samples will be sent to the above facility after being collected by the Department of Cardiovascular Medicine, Niigata University Graduate School of Medical and Dental Sciences.	Change in how to handle samples due to the change in central measurement facility.

STATISTICAL ANALYSIS PLAN

Placebo-controlled, double-blind study of empagliflozin and implantable cardioverter-defibrillator in patients with type 2 diabetes

EMPA-ICD

jRCTs031180120

Clinical and Translational Research Center, Niigata University Medical & Dental Hospital

Date: DEC 8, 2021 Ver1.0.0

Revision History

Revision	Date of preparation	Author	Description of change
1.0.0	Dec 8, 2021	Takahiro Tanaka	First edition prepared

Table of Contents

1. INTRODUCTION 1

2. STUDY OBJECTIVE AND DESIGN 1

2.1. Study Objectives 1

2.2. Study Design 1

2.3. Randomization method 1

2.4. Time Points of Evaluation 2

2.5. Determination of Sample Size 3

3. ENDPOINTS 3

3.1. Efficacy Endpoints 3

3.2. Safety Endpoints 4

4. DEFINITION OF DERIVED VARIABLE 5

5. ANALYSIS SETS 5

5.1. Full analysis set (FAS) 5

5.2. Per protocol set (PPS) 6

5.3. Safety analysis set (SAS) 6

6. DATA HANDLING 6

6.2. Handling of calculated values 6

6.4. Handling of Protocol Deviations 7

6.5. Time Windows for Measurement Time Points 7

7. STATISTICAL METHOD 8

7.1. Analysis Sets 8

7.1.1. Sample Size of Each Analysis Set 8

7.2. Demographic Data and Baseline Subject Characteristics 8

7.2.1. The summaries of demographic data and baseline subject characteristics 8

7.2.1.1. Analysis set 8

7.2.1.2. Analytical parameters 8

7.2.1.3. Analysis methods 10

7.2.1.4. Level of significance and confidence coefficient 10

7.3. Treatment Compliance Status 10

7.4. Efficacy Analysis 11

7.4.1. Primary Endpoint 11

7.4.1.1. Analysis set 11

7.4.1.2. Analytical parameter 11

7.4.1.3. Analysis method 11

7.4.1.4. Level of significance and confidence coefficient 11

7.4.2. Secondary Endpoints 11

7.4.2.1. Analysis set 11

7.4.2.2. Analytical parameters 11

7.4.2.3. Analysis method 12

7.4.2.4. Level of significance and confidence coefficient 14

7.5. Safety Analysis 15

7.5.1. Tabulation of Adverse Events 16

7.5.1.1. Analysis set 16

7.5.1.2. Analytical parameters 16

7.5.1.3. Analysis method 16

7.5.1.4. Level of significance and confidence coefficient 16

8. SOFTWARE TO BE USED FOR STATISTICAL ANALYSIS 16

9. CHANGES FROM THE PROTOCOL SPECIFIED FOR STATISTICAL ANALYSIS 16

10. LIST OF TABLES 17

11. LIST OF FIGURES 18

Appendix 19

List of Abbreviations and Definition of Terms

Abbreviations	Meaning
FAS	Full Analysis Set
IRB	Institutional Review Board
PPS	Per Protocol Set
LVEF	Left Ventricular Ejection Fraction

1. INTRODUCTION

This document provides more detailed information on the statistical analysis plan for the efficacy and safety of the "Double-blind, placebo-controlled study to evaluate the change in the number of severe arrhythmias after empagliflozin intervention in patients with an implanted arrhythmia treatment device complicated by type 2 diabetes mellitus" in addition to the information in the study protocol.

2. STUDY OBJECTIVE AND DESIGN

2.1. Study Objectives

The main objective of this study is to determine whether empagliflozin improves the number of severe arrhythmia events in patients with arrhythmia treatment device implantation complicated by type 2 diabetes.

2.2. Study Design

Multicenter, prospective, placebo-controlled, randomized, double-blind, parallel-group, physician-initiated clinical study

2.3. Randomization method

Stratified randomization: stratification factor “LVEF/Age/Sex”

2.4. Time Points of Evaluation

Enrollment and allocation (between Screening and Visit [1]) | Initiation of study drug (between Visit [1] and Visit [2])

	Before treatment		Treatment period		
	Screening	Visit [1]	Visit [2]	Visit [3]	At time of discontinuation*11
	Week -12 to 0	Week 0	Week 12	Week 24*10	
		–	(± 4 weeks)	(± 4 weeks)	
Patient information/ informed consent	○				
Patient background	○				
Interview/physical examination	○	○	○	○	△
Confirmation of drug compliance			○	○	△
Confirmation of compliance with study procedures		○	○	○	△
Confirmation of other drugs being used	○	○	○	○	△
Height	○				△
Body weight/body temperature	○	○	△	○	△
Blood pressure/ pulse rate*1		○	△	○	△
Hematological tests	○*4,5	○*4	△	○	△
Hematological tests (blood glucose/lipids)*2	○*4,5	○*4	△	○	△
Hematological tests (special)*2		○*6,7,8		○*7,8	△*7,8
12-lead ECG		○*4		○	△
Holter monitoring		○*4		○	△
Echocardiography	○*4	○*4		○	△
^{123}I-MIBG myocardial scintigraphy		△*4,8		△*8	△*8
Adverse events*3	○	○	○	○	△
Evaluation of arrhythmia device		○*9		○	△

For details of *1-11, refer to Protocol 8.1.

2.5. Determination of Sample Size

The planned sample size has been determined to be 105 subjects per group, with a total of 210 subjects in both groups.

[Rationale for sample size determination]

On the basis of the previous report[9] and by using a generalized linear model for differences in the number of arrhythmias, the incidence rate ratio (IRR) of severe arrhythmia in the placebo to empagliflozin group is assumed to be 1.44. Thus, at a significance level of 0.05 and power of 80%, 99 patients per group will be necessary to detect an IRR (i.e., effect size) of 1.44. Assuming that 10 patients (5%) will drop out during the study, 200 patients are estimated to be required. The number of patients who can feasibly be enrolled is 210, assuming a total of 20 institutions, each of which can recruit 10 to 15 patients. Thus, the required number of patients is feasible, and the target number of patients has been set at 210.

Number of subjects required per power

power	Sample Size
60%	124 subjects (62+62)
70%	156 subjects (78+78)
80%	198 subjects (99+99)
90%	266 subjects (133+133)

3. ENDPOINTS

3.1. Efficacy Endpoints

3.1.1. Primary Endpoint

The number of severe arrhythmia events (NSVT/VT/VF) recorded in the arrhythmia device will be evaluated at Week 0 and 24 (or at study discontinuation) and used to calculate the difference in the number of severe arrhythmia events between the empagliflozin and placebo groups.

3.1.2 Secondary Endpoints

1) The number of severe arrhythmia events (NSVT/VT/VF) and appropriate device operations (anti-tachycardia pacing/shock operation) recorded in the arrhythmia device will be evaluated at Week 0 and 24 (or at study discontinuation) and used to calculate the following:

(1-1) Change in the number of severe arrhythmia events

(1-2) Incidence of severe arrhythmia events before and after treatment

(1-3) Number of severe arrhythmia events at Week 24
(1-4) Presence or absence of severe arrhythmia events at Week 24
(1-5) Change rate of the number of appropriate device operations
(1-6) Change in the number of appropriate device operations
(1-7) Incidence of appropriate device operations before and after treatment
(1-8) Number of appropriate device operations at Week 24
(1-9) Presence or absence of appropriate device operations at Week 24

These variables will be compared between the empagliflozin and placebo groups.

In addition, the following will be evaluated at Week 0 and 24 (or at study discontinuation) to compare the change rate, change, and values at Week 24 between the empagliflozin and placebo groups:

2) Holter electrocardiographic tests: total recording time, total number of ventricular premature complexes (VPCs), number of single VPCs, number of occurrences of two consecutive VPCs, and number of ventricular tachycardia
3) Blood ketone body fraction (acetoacetic acid, 3-hydroxybutyric acid, and total ketone bodies), i.e., blood concentration of each ketone body
4) Blood catecholamine fraction (adrenalin, noradrenalin, and dopamine), i.e., blood concentration of each catecholamine

The correlation between the difference in the number of severe arrhythmia events and blood ketone body or catecholamine fraction will be compared between the empagliflozin and placebo groups.

3.2. Safety Endpoints

1) Adverse events and adverse reactions that occur in the period from the date of informed consent until Week 24 (or study discontinuation).

3.2.1. Definition of Adverse Events

An adverse event is defined as any untoward medical occurrence (including laboratory abnormalities) in patients administered a study drug; it does not necessarily have to have a causal relationship with the study drug. Among such adverse events, those whose causal relationship to the study drug cannot be ruled out are called "disease or the like." In case of any adverse event, the Investigator or Sub-investigator will promptly take appropriate measures to treat the patient and ensure their safety. In addition, if administration of the study drug is discontinued or treatment is required for adverse events, the Investigator or Sub-investigator will inform patients accordingly. For measures to be taken in the event of adverse events, refer to Appendix G (Procedure for Reporting Adverse Events).

[Adverse reactions to empagliflozin expected from the package insert] (Refer to Appendix A)
Clinically significant adverse reactions: hypoglycemia, dehydration, ketoacidosis, pyelonephritis, and sepsis
Other adverse reactions: infections (cystitis, urinary tract infection, asymptomatic bacteriuria,

vulvovaginal candidiasis, trichomoniasis, bacterial vaginitis, vulvovaginitis); reproductive system disorders (balanitis, genital pruritus, balanoposthitis, vulvovaginal discomfort, and vulvovaginal pruritus); metabolism and nutrition disorders (hyperlipidemia and decreased fluid volume); blood and lymphatic system disorders (hemoconcentration); nervous system disorders (dizziness and dysgeusia); gastrointestinal disorders (constipation and abdominal distention); skin and subcutaneous tissue disorders (rash, pruritus, and urticaria); renal and urinary disorders (pollakiuria, polyuria, increased urine output, and dysuria); general disorders (thirst and feeling hungry), and investigations (weight decreased, urine ketone body present, and blood ketone body present)
According to the guidance of the US Food and Drug Administration (FDA; dated August 29, 2018), attention should be paid also to the onset of necrotizing fasciitis of the perineum (Fournier's gangrene) as a clinically significant adverse reaction.

3.2.2. Definition of SAEs

SAE is any adverse event that results in one of the following outcomes:

(1) Death
(2) Disability
(3) Possibly leading to death
(4) Possibly leading to disability
(5) Requiring inpatient hospitalization or prolongation of existing hospitalization in a hospital or clinic
(6) Serious outcome that is equivalent to the outcomes (1)-(5)
(7) Congenital anomaly or birth defect in next generations

"Hospitalization" in (5) does not include that intended only for therapy or examination that has been planned before the study (such as planned surgery and examination) (events that are newly developed during the hospitalization will be regarded as adverse events).

4. DEFINITION OF DERIVED VARIABLE

The derived variable is defined in the table below.

Derived variable	Calculation
Age	Subtract [date of birth] from [date of measurement]

5. ANALYSIS SETS

5.1. Full analysis set (FAS)

FAS includes all subjects enrolled in this study but excludes subjects with major protocol violation (failure to obtain informed consent and major procedural violation).

5.2. Per protocol set (PPS)

PPS includes all enrolled subjects but excludes subjects with any of the following major violations of the criteria for study treatment and concomitant therapy specified in the study protocol:

・ Violation of the inclusion criteria
・ Violation of the exclusion criteria
・ Violation of the prohibited concomitant drugs and therapies

5.3. Safety analysis set (SAS)

SAS includes all enrolled subjects but excludes subjects who did not take any dose of the study drug.

6. DATA HANDLING

6.1.1. Handling of Missing Data

In principle, missing values will not be imputed; that is, missing data will be rejected.

6.2. Handling of calculated values

Geometric mean value, geometric coefficient of variation

Geometric mean value = exp (mean logarithmically transformed value)

geometric coefficient of variation =

$$100 \times \sqrt{\text{exp}(\text{standard deviationof of logarithmic transformed value}^2) - 1}$$

6.3. Handling of Number of Display Digits

(1) Number of display digits

The number of display digits for summary statistics is increased to the number of digits of the measurement value plus 1 digit. Minimum and maximum values are displayed with the same digits as data obtained. Test statistics are rounded to 2 decimal places in principle. P values are rounded to 4 decimal places.

6.4. Handling of Protocol Deviations

Analytical handling of subjects and data in which protocol deviation is identified is defined in the table below.

Items of protocol deviations		Subject handling				Data handling
		FAS	mITT	PPS	SAS	
Violation of [Ethical Guidelines for Medical and Health Research Involving Human Subjects]		×	×	×	×	
Subjects who never received the study drug		○	×	×	×	
Violation of inclusion/exclusion criteria		○	○	×	○	All data will be acceptable.
Procedural deviation	Non-compliance with the dosage regimen	○	○	×	○	All data will be acceptable.
	Violation of prohibited concomitant drugs	○	○	×	○	All data will be acceptable.
	Missed examinations, etc.	○	○	×	○	All data will be acceptable.
	Violation of discontinuation criteria	○	○	×	○	Data obtained after consent withdrawal will be rejected.
Violation of the time window for measurement time points		○	○	×	○	Data handling is specified in 6.5. Time Windows for Measurement Time Points.

6.5. Time Windows for Measurement Time Points

Time windows for measurement time points for analyses are specified in the table below.

Scheduled evaluation time point	Time window (day)	Remarks
Visit 0 Eligibility check	-84<, ≤-1	If 2 or more values are available within the time window, the value obtained on the day closest to the scheduled time point will be used.<br>(If the 2 dates are different from the scheduled time point by the same number
Visit 1 (day 0)	0	
Visit 2 (day 84)	1<, ≤ 126	
Visit 3 (day 168)	126<, ≤ 196	

7. STATISTICAL METHOD

7.1. Analysis Sets

7.1.1. Sample Size of Each Analysis Set

No. of subjects who gave their consent, No. of enrolled subjects, No. of subjects randomized to each group, No. of subjects and withdrawals of FAS, PPS and SAS of each group, and No. of subjects excluded from each analysis set by the reason will be shown in tables and patient flow diagram. In addition, the list of withdrawals, subjects excluded from each analysis set and deviations will be shown in a table.

7.2. Demographic Data and Baseline Subject Characteristics

7.2.1. The summaries of demographic data and baseline subject characteristics

7.2.1.1. Analysis set

The analysis set will be the FAS.

7.2.1.2. Analytical parameters

The following baseline characteristics information will be used in the analysis.

Characteristic	Data type	Definition/classification
Sex	Binary	Man/woman
Age	measured value	Descriptive statistics
	Category	~19/20~29/30~39/40~49/<br>50~59/60~69/70~
Smoking history	Category	Current/Ex/Never
Alcohol drinking history	Category	~19/20~29/30~39/40~49/<br>50~59/60~69/70~
Life-threatening arrhythmia that resulted in implantation of arrhythmia device	Category	Ventricular fibrillation/monomorphic ventricular tachycardia/polymorphic ventricular tachycardia/nonsustained ventricular tachycardia
Underlying diseases *1	Binary	Present/Absent
Maker of arrhythmia treatment device	Category	BIOTRONIK/Boston Scientific/<br>Japan Lifeline/Medtronic/Abbott
Type of arrhythmia treatment device	Category	See Appendix: List of the types of arrhythmia treatment devices
Comorbidity and past history *2	Binary	Present/Absent

Past history of non-drug treatment [*3]	Binary	Present/Absent
NYHA classification	Category	I/II/III/IV
Height	measured value	Descriptive statistics
Body weight	measured value	Descriptive statistics
Body temperature	measured value	Descriptive statistics
Blood pressure (SBP/DBP)	measured value	Descriptive statistics
Pulse rate	measured value	Descriptive statistics
Hematological [*4]	measured value	Descriptive statistics
Blood biochemical [*5]	measured value	Descriptive statistics
Blood glucose tests<br>Fasting blood glucose, HbA1c	measured value	Descriptive statistics
Lipid tests<br>TG, TC, HDL-C, LDL-C	measured value	Descriptive statistics
Hematological tests (special)<br>Blood ketone body fraction[*6]<br>Blood catecholamine fraction[*7]<br>DNA tests [*8]	measured value	Descriptive statistics
12-lead ECG tests<br>Heart rate and rhythm; PQ, QRS, and QT interval	measured value	Descriptive statistics
Holter monitoring<br>Total recoding time, total number of VPCs, number of single VPCs, number of occurrences of two consecutive VPCs, and number of ventricular tachycardia	measured value	Descriptive statistics

Echocardiographic tests *9	measured value	Descriptive statistics
^{123}I-MIBG myocardial scintigraphy tests<br>H/M ratio and washout rate	measured value	Descriptive statistics
Evaluation of arrhythmia device *10	measured value	Descriptive statistics

*1. Ischemic heart disease, cardiac valve disease, dilated cardiomyopathy, hypertrophic cardiomyopathy, restrictive cardiomyopathy, arrhythmogenic right ventricular cardiomyopathy, left ventricular non-compaction, mitochondrial cardiomyopathy, other cardiomyopathy, Brugada syndrome, long QT syndrome, idiopathic ventricular fibrillation, etc.
*2. Past history of atrial fibrillation, hypertension, dyslipidemia, cerebral infarction, or cerebral hemorrhage
*3. PCI, CABG, cardiac valvulopathy surgery, catheter ablation, etc.
*4. RBC count, WBC count, Hb, Ht, and Plt
*5. Total protein, albumin, AST, ALT, ALP, T-Bil, LDH, BUN, Na, K, Cl, uric acid, Cr, and eGFR
*6. Acetoacetic acid, 3-hydroxybutyric acid, and total ketone bodies
*7. Adrenalin, noradrenalin, dopamine, erythropoietin, and reticulocytes
*8. Telomere length, G-tail length, RNA tests (P53, P21 and P16), and metabolome analysis
*9. Systolic capacity (LVEF) and diastolic capacity (LVEF, E wave, A wave, E/A, sep-e’, lat-e’, E/e’, LAVI, peak TRV)
*10. Severe arrhythmia (NSVT/VT/VF), number of events required to appropriately treat severe arrhythmia (anti-tachycardia pacing/shock operation), biological monitoring indicators, and number of atrial fibrillations

7.2.1.3. Analysis methods

Frequency and proportion will be calculated for discrete data; summary statistics (number of subjects, mean, standard deviation, minimum, median, and maximum) will be calculated for continuous data.

7.2.1.4. Level of significance and confidence coefficient

All tests will be two-sided at a significance level of 5%. The two-sided confidence interval will be used with a confidence coefficient of 95%.

7.3. Treatment Compliance Status

7.3.1. Summary Statistics of Treatment Compliance Data

7.3.1.1. Analysis set

The analysis of treatment compliance will be based on the SAS population.

7.3.1.2. Analytical parameters

Medication compliance at 12 weeks and 24 weeks (or discontinuation)

7.3.1.3. Analysis method

Listing of medication compliance at 12 weeks and 24 weeks (or discontinuation).

7.4. Efficacy Analysis

7.4.1. Primary Endpoint

7.4.1.1. Analysis set

The primary efficacy analysis will be based on the FAS population.

7.4.1.2. Analytical parameter

The number of severe arrhythmia events (NSVT/VT/VF) recorded in the arrhythmia device will be evaluated at Week 0 and 24 (or at study discontinuation) to calculate the difference in the number of severe arrhythmia events between the empagliflozin and placebo groups.

7.4.1.3. Analysis method

The number of severe arrhythmia events (NSVT/VT/VF) recorded in the arrhythmia device will be evaluated at Week 0 and 24 (or at study discontinuation) and subjected to Poisson regression analysis by the generalized linear model, whereby the difference in the number of severe arrhythmia events between the empagliflozin and placebo groups will be used as an objective variable.

7.4.1.4. Level of significance and confidence coefficient

All tests for efficacy evaluation will be two-sided at a significance level of 5%. The two-sided confidence interval will be used with a confidence coefficient of 95%.

7.4.2. Secondary Endpoints

7.4.2.1. Analysis set

The secondary efficacy analysis will be based on the FAS population.

7.4.2.2. Analytical parameters

1) The number of severe arrhythmia events (NSVT/VT/VF) and appropriate device operations (anti-tachycardia pacing/shock operation) recorded in the arrhythmia device will be evaluated at Week 0 and 24 (or at study discontinuation) to calculate the following:

(1-1) Change in the number of severe arrhythmia events

(1-2) Incidence of severe arrhythmia events before and after treatment

(1-3) Number of severe arrhythmia events at Week 24

(1-4) Presence or absence of severe arrhythmia events at Week 24

(1-5) Change rate of number of appropriate device operations

(1-6) Change in number of appropriate device operations

(1-7) Incidence of appropriate device operations before and after treatment

(1-8) Number of appropriate device operations at Week 24

(1-9) Presence or absence of appropriate device operations at Week 24

These variables will be compared between the empagliflozin and placebo groups.

In addition, the following will be evaluated at Week 0 and 24 (or at study discontinuation) to compare the change rate, change, and values at Week 24 between the empagliflozin and placebo groups:

2) Holter electrocardiographic tests: total recording time, total number of VPCs, number of single VPCs, number of occurrences of two consecutive VPCs, and number of ventricular tachycardia

3) Blood ketone body fraction (acetoacetic acid, 3-hydroxybutyric acid, and total ketone bodies), i.e., blood concentration of each ketone body

4) Blood catecholamine fraction (adrenalin, noradrenalin, and dopamine), i.e., blood concentration of each catecholamine

The correlation between the difference in the number of severe arrhythmia events and blood ketone body or catecholamine fractions will be compared between the empagliflozin and placebo groups.

7.4.2.3. Analysis method

[Primary analysis]:

1) The number of severe arrhythmia events (NSVT/VT/VF) and appropriate device operations (anti-tachycardia pacing/shock operation) recorded in the arrhythmia device will be evaluated at Week 0 and 24 (or at study discontinuation).

(1-1) Change in the number of severe arrhythmia events

The difference in the change in the number of severe arrhythmia events until Week 0 and from Week 0 to 24 (or study discontinuation) will be compared between the empagliflozin and placebo groups by a two-sample test.

(1-2) Incidence of severe arrhythmia events before and after treatment

Incidences of severe arrhythmia events until Week 0 and from Week 0 to 24 (or study discontinuation) in the empagliflozin and placebo groups will be summarized.

(1-3) Number of severe arrhythmia events at Week 24

The difference in the number of severe arrhythmia events from Week 0 to 24 (or study discontinuation) will be compared between the empagliflozin and placebo groups by a two-sample test.

(1-4) Presence or absence of severe arrhythmia events at Week 24

The difference in the incidence of severe arrhythmia events from Week 0 to 24 (or study discontinuation) will be compared between the empagliflozin and placebo groups by Fisher's exact test.

(1-5) Change rate of number of appropriate device operations

The change rates of the number of appropriate device operations until Week 0 and from Week 0 to 24 (or study discontinuation) in the empagliflozin and placebo groups will be summarized.

(1-6) Change in number of appropriate device operations

The difference in the change in the number of appropriate device operations until Week 0 and from Week 0 to 24 (or study discontinuation) will be compared between the empagliflozin and placebo groups by a two-sample test.

(1-7) Incidence of appropriate device operations before and after treatment

The incidences of appropriate device operations until Week 0 and from Week 0 to 24 (or study discontinuation) and the 95% confidence interval of the empagliflozin and placebo groups will be calculated.

(1-8) Number of appropriate device operations at Week 24

The difference in the number of appropriate device operations from Week 0 to 24 (or study discontinuation) will be compared between the empagliflozin and placebo groups by a two-sample test.

(1-9) Presence or absence of appropriate device operations at Week 24

The difference in the incidence of appropriate device operations from Week 0 to 24 (or study discontinuation) will be compared between the empagliflozin and placebo groups by Fisher's exact test.

2) Holter electrocardiographic tests: total recording time, total number of VPCs, number of single VPCs, number of occurrences of two consecutive VPCs, and number of ventricular tachycardia.

For the total recording time, total number of VPCs, number of single VPCs, number of occurrences of two consecutive VPCs, and number of ventricular tachycardia at each time point of the Holter electrocardiographic tests in the empagliflozin and placebo groups, the number of participants, mean, median, standard deviation, and interquartile range will be calculated. The change over time at each measurement time in individual participants will be displayed by treatment group in a graph (mean ± standard deviation).

3) Blood ketone body fraction (acetoacetic acid, 3-hydroxybutyric acid, and total ketone bodies), i.e., blood concentration of each ketone body

For acetoacetic acid, 3-hydroxybutyric acid, and total ketone bodies in the empagliflozin and placebo groups, the number of participants, mean, median, standard deviation, and interquartile range will be calculated. The change over time at each measurement time in individual participants will be displayed by treatment group in a graph (mean ± standard deviation).

4) Blood catecholamine fraction (adrenalin, noradrenalin, and dopamine), i.e., blood concentration of each catecholamine

For adrenalin, noradrenalin, and dopamine in the empagliflozin and placebo groups, the number of participants, mean, median, standard deviation, and interquartile range will be calculated. The change over time at each measurement time in individual participants will be displayed by treatment group in a graph (mean ± standard deviation).

7.4.2.4. Level of significance and confidence coefficient

All tests for efficacy evaluation will be two-sided at a significance level of 5%. The two-sided confidence interval will be used with a confidence coefficient of 95%.

7.4.3. Other Endpoints

7.4.3.1. Analysis set

The analysis of other variables will be based on the FAS population.

7.4.3.2. Analytical parameters

1) The number of atrial fibrillation events and the biological monitoring index recorded in the arrhythmia device will be evaluated at Week 0 and 24 to calculate the following, and the relationship with ventricular arrhythmia also will be examined:

(1-1) Change rate of the number of atrial fibrillation events
(1-2) Change in the number of atrial fibrillation events
(1-3) Incidence of atrial fibrillation events before and after treatment
(1-4) Number of atrial fibrillation events at Week 24
(1-5) Presence or absence of atrial fibrillation events at Week 24
(1-6) Change rate of biological monitoring index
(1-7) Change in biological monitoring index
(1-8) Biological monitoring index at Week 24

These variables will be compared between the empagliflozin and placebo groups.

2) Each of the following items will be evaluated at Week 0 and 24 (or at study discontinuation) to compare the change rate, change, and value at Week 24 between the empagliflozin and placebo groups:

(2-2) NYHA classification of cardiac function
(2-3) Body weight
(2-4) Blood pressure, pulse rate, and body temperature
(2-5) Blood glucose, assessed by HbA1c and fasting blood glucose
(2-6) Serum lipid, serum uric acid, and the cardiac failure markers TC, HDL-C, LDL-C, TG, UA, and BNP
(2-7) Renal function, assessed by serum Cr, eGFR, BUN, Na, K, and Cl
(2-8) Hepatic function, assessed by total protein, albumin, AST, ALT, ALP, T-Bil, and LDH
(2-9) Hematological value, assessed by RBC count, WBC count, Hb, Ht, and Plt
(2-10) Erythropoietin
(2-11) Reticulocytes
(2-12) DNA tests (telomere length)
(2-13) RNA tests (P53, P21, and P16)

(2-14) Metabolome analysis
(2-15) 12-lead ECG (pulse rate and PQ, QRS, and QT intervals)
(2-16) Systolic and diastolic capacity measured by echocardiography (LVEF, E wave, A wave, E/A, sep-e’, lat-e’, E/e’, LAVI, peak TRV, and HR)
(2-17) ^{123}I-MIBG myocardial scintigraphy-obtained sympathetic activity index, assessed by heart-to-mediastinum ratio (H/M), and washout rate

3) Subgroup analysis will be performed on the following items, as necessary:
(3-1) Blood glucose control
(3-2) Concentration of ketone bodies
(3-3) Increase in erythropoietin
(3-4) Renal function
(3-5) Hepatic function

7.4.3.3. Analysis method

[Primary analysis]:
For each endpoint, changes in the measured values over time are shown graphically for each time point in each treatment group (as mean ± standard deviation). The number of subjects, means, medians, standard deviations, and interquartile ranges will be calculated for each time period and measurement time point. For the discrete variables, the proportions will be determined at each time point in each treatment group. The number of subjects, proportions, and 95% confidence intervals will also be calculated for each time period and measurement time point.

[Secondary analysis]:
Secondary analysis will not be performed in this study.

7.4.3.4. Level of significance and confidence coefficient

All tests for efficacy evaluation will be two-sided at a significance level of 5%. The two-sided confidence interval will be used with a confidence coefficient of 95%.

7.5. Safety Analysis

An adverse event is any unfavorable and unintended sign (including an abnormal laboratory change), symptom, or disease that develops in a subject, whether or not related to the study drug. Underlying diseases are not considered as adverse events, except where the severity worsens or the frequency increases after study treatment. A laboratory change is determined to be an adverse event if it is considered to be unfavorable for the subject by the investigators. The causal relationship between an adverse event and the study drug will be determined

according to the following criteria. An adverse event considered related to the study drug is regarded as an adverse drug reaction.

Related: There is a reasonable possibility of the causal relationship between the study treatment and an adverse event.

Not related: There is no reasonable possibility of the causal relationship between the study treatment and an adverse event.

7.5.1. Tabulation of Adverse Events

7.5.1.1. Analysis set

The analysis of adverse events will be based on the SAS population.

7.5.1.2. Analytical parameters

SAEs occurring from the initiation of study treatment through after the end of study.

7.5.1.3. Analysis method

A list of adverse events will be prepared showing the number of events and participants. Adverse reactions will be classified as “related” or “not related.”

7.5.1.4. Level of significance and confidence coefficient

All tests for safety evaluation will be two-sided at a significance level of 5%. The two-sided confidence interval will be used with a confidence coefficient of 95%.

8. SOFTWARE TO BE USED FOR STATISTICAL ANALYSIS

SAS for Windows (release 9.4 or later) will be used for statistical analysis.

9. CHANGES FROM THE PROTOCOL SPECIFIED FOR STATISTICAL ANALYSIS

Not applicable.

10. LIST OF TABLES

Table No.	Details
T7.1.1	List of discontinued cases
T7.1.2	List of cases excluded from analysis
T7.2	Subject demographics
T7.3.1	Study drug compliance rate
T7.4.1.1	Primary endpoint: difference in number of serious arrhythmic events
T7.4.2.1.1	Changes in number of serious arrhythmic events
T7.4.2.1.2	Pre- and post-treatment incidence of severe arrhythmic events
T7.4.2.1.3	Number of severe arrhythmic events at 24 weeks
T7.4.2.1.4	Presence of severe arrhythmic events at 24 weeks
T7.4.2.1.5	Percentage change in number of appropriate shocks
T7.4.2.1.6	Changes in number of appropriate shocks
T7.4.2.1.7	Pre- and post-treatment incidence of appropriate shocks
T7.4.2.1.8	Number of appropriate shocks at 24 weeks
T7.4.2.1.9	Presence of appropriate shocks at 24 weeks
T7.4.2.2	Holter ECG examination: total recording time, total number of VPCs, number of single VPCs, number of double VPCs, number of ventricular tachycardia
T7.4.2.3	Blood ketone fractions (acetoacetic acid, 3-hydroxybutyric acid, total ketones): concentration of blood ketone bodies
T7.4.2.4	Blood catecholamine fractions (adrenaline, noradrenaline, dopamine): catecholamine concentration in each blood sample
T7.4.3.1.1	Percent change in number of atrial fibrillation events
T7.4.3.1.2	Changes in number of atrial fibrillation events
T7.4.3.1.3	Pre- and post-treatment incidence of atrial fibrillation events
T7.4.3.1.4	Number of atrial fibrillation events at 24 weeks
T7.4.3.1.5	Presence of atrial fibrillation events at 24 weeks
T7.4.3.1.6	Percentage change in biomonitoring indicators
T7.4.3.1.7	Changes in biomonitoring indicators
T7.4.3.1.8	Biomonitoring indicators at 24 weeks
T7.4.3.2	For each parameter, change and percentage change from week 0 to week 24 (or discontinuation) and value at 24 weeks

11. LIST OF FIGURES

Figure No.	Details
F7.1.1	Details of subjects
F7.4.1.1	Primary endpoint: difference in number of serious arrhythmic events
F7.4.2.1.1	Change in number of serious arrhythmic events
F7.4.2.1.3	Number of severe arrhythmic events at 24 weeks
F7.4.2.1.6	Change in number of appropriate shocks
F7.4.2.1.8	Number of appropriate shocks at 24 weeks
F7.4.3.1.2	Change in number of atrial fibrillation events
F7.4.3.1.4	Number of atrial fibrillation events at 24 weeks
F7.4.3.1.7	Change in biomonitoring indicators
F7.4.3.2	For each parameter, change and percentage change from week 0 to week 24 (or discontinuation) and value at 24 weeks
T7.5.1	Percentage of adverse events

Appendix

**Relevant information that cannot be included in the main body of this document are described and indicated in this section as necessary.*

List of the types of arrhythmia treatment devices

11101	[BIOTRONIK] [ICD] Ilivia 7 DR-T DF-1 ProMRI	22402	[Boston Scientific] [CRT-D] COGNIS 100-D
11102	[BIOTRONIK] [ICD] Ilivia 7 DR-T DF4 ProMRI	29999	[Boston Scientific] [No applicable models] Free comment
11103	[BIOTRONIK] [ICD] Ilivia 7 VR-T DX DF-1 ProMRI	31101	[Japan Lifeline] [ICD] Ovatio
11104	[BIOTRONIK] [ICD] Ilivia 7 VR-T DF-1 ProMRI	31201	[Japan Lifeline] [ICD] PARADYM
11105	[BIOTRONIK] [ICD] Ilivia 7 VR-T DF4 ProMRI	31202	[Japan Lifeline] [ICD] PARADYM 2
11201	[BIOTRONIK] [ICD] Inlexa 7 DR-T DF-1	31203	[Japan Lifeline] [ICD] PARADYM RF
11202	[BIOTRONIK] [ICD] Inlexa 7 DR-T DF4	31301	[Japan Lifeline] [ICD] INTENSIA
11203	[BIOTRONIK] [ICD] Inlexa 7 VR-T DX DF-1	31401	[Japan Lifeline] [ICD] PLATINIUM
11204	[BIOTRONIK] [ICD] Inlexa 7 VR-T DF4	32501	[Japan Lifeline] [CRT-D] PARADYM 2
11301	[BIOTRONIK] [ICD] Iperia 7 DR-T DF4 ProMRI	32502	[Japan Lifeline] [CRT-D] PARADYM RF
11302	[BIOTRONIK] [ICD] Iperia 7 VR-T DX DF-1 ProMRI	32601	[Japan Lifeline] [CRT-D] INTENSIA
11303	[BIOTRONIK] [ICD] Iperia 7 VR-T DF4 ProMRI	32701	[Japan Lifeline] [CRT-D] PLATINIUM
11401	[BIOTRONIK] [ICD] Itrevia 5 DR-T DF-1	39999	[Japan Lifeline] [No applicable models] Free comment
11402	[BIOTRONIK] [ICD] Itrevia 5 DR-T DF4	41101	[Medtronic] [ICD] Secura DR
11403	[BIOTRONIK] [ICD] Itrevia 5 VR-T DX DF-1	41201	[Medtronic] [ICD] Protecta XT DR
11404	[BIOTRONIK] [ICD] Itrevia 5 VR-T DF4	41301	[Medtronic] [ICD] Evera XT DR
12101	[BIOTRONIK] [CRT-D] Intica 7 HF-T QP DF-1 ProMRI	41302	[Medtronic] [ICD] Evera MRI XT DR SureScan
12102	[BIOTRONIK] [CRT-D] Intica 7 HF-T QP DF4 ProMRI	41102	[Medtronic] [ICD] Secura VR
12103	[BIOTRONIK] [CRT-D] Intica 7 HF-T DF-1 ProMRI	41202	[Medtronic] [ICD] Protecta XT VR
12104	[BIOTRONIK] [CRT-D] Intica 7 HF-T DF4 ProMRI	41303	[Medtronic] [ICD] Evera XT VR
12201	[BIOTRONIK] [CRT-D] Inlexa 7 HF-T QP DF-1	41304	[Medtronic] [ICD] Evera MRI XT VR SureScan
12202	[BIOTRONIK] [CRT-D] Inlexa 7 HF-T QP DF4	41401	[Medtronic] [ICD] Visia AF MRI XT VR
12203	[BIOTRONIK] [CRT-D] Inlexa 7 HF-T DF-1	42101	[Medtronic] [CRT-D] Consulta CRT-D
12204	[BIOTRONIK] [CRT-D] Inlexa 7 HF-T DF4	42201	[Medtronic] [CRT-D] Protecta XT CRT-D
12301	[BIOTRONIK] [CRT-D] Itrevia 7 HF-T QP ProMRI	42301	[Medtronic] [CRT-D] Viva
12302	[BIOTRONIK] [CRT-D] Itrevia 7 HF-T DF-1 ProMRI	42401	[Medtronic] [CRT-D] Compia MRI Quad CRT-D
12303	[BIOTRONIK] [CRT-D] Itrevia 7 HF-T DF4 ProMRI	42501	[Medtronic] [CRT-D] Amplia MRI Quad CRT-D
12304	[BIOTRONIK] [CRT-D] Itrevia 5 HF-T QP	42601	[Medtronic] [CRT-D] Claria MRI Quad CRT-D
12305	[BIOTRONIK] [CRT-D] Itrevia 5 HF-T DF-1	49999	[Medtronic] [No applicable models] Free comment
12306	[BIOTRONIK] [CRT-D] Itrevia 5 HF-T DF4	51101	[Abbott] [ICD] Fortify Assura DR DF4

19999	[BIOTRONIK] [No applicable models] Free comment	51102	[Abott] [ICD] Fortify Assura VR DF4
21101	[Boston Scientific] [ICD] RESONATE EL ICD VR	51201	[Abott] [ICD] Ellipse DR DF4
21102	[Boston Scientific] [ICD] RESONATE EL ICD DR	51202	[Abott] [ICD] Ellipse VR DF4
21201	[Boston Scientific] [ICD] MOMENTUM EL ICD VR	51203	[Abott] [ICD] Ellipse Limited DR
21202	[Boston Scientific] [ICD] MOMENTUM EL ICD DR	51204	[Abott] [ICD] Ellipse Limited VR
21301	[Boston Scientific] [ICD] CHARISMA EL ICD VR	51301	[Abott] [ICD] AnalyST DR RF
21302	[Boston Scientific] [ICD] CHARISMA EL ICD DR	51302	[Abott] [ICD] AnalyST VR RF
21401	[Boston Scientific] [ICD] PERCIVA MINI ICD VR	51401	[Abott] [ICD] Current DR RF
21402	[Boston Scientific] [ICD] DYNAGEN? MINI ICD	51402	[Abott] [ICD] Current VR RF
21403	[Boston Scientific] [ICD] DYNAGEN? EL ICD	51501	[Abott] [ICD] FortifyST DR
21404	[Boston Scientific] [ICD] INCEPTA? ICD	51502	[Abott] [ICD] FortifyST VR
21501	[Boston Scientific] [ICD] TELIGEN 100	52101	[Abott] [CRT-D] Unify
22101	[Boston Scientific] [CRT-D] RESONATE X4 CRT-D	52102	[Abott] [CRT-D] Unify Assura
22102	[Boston Scientific] [CRT-D] MOMENTUM X4 CRT-D	52103	[Abott] [CRT-D] Unify Quadra
22201	[Boston Scientific] [CRT-D] MOMENTUM CRT-D	52201	[Abott] [CRT-D] Quadra? Assura
22202	[Boston Scientific] [CRT-D] CHARISMA X4 CRT-D	52301	[Abott] [CRT-D] Promote RF
22203	[Boston Scientific] [CRT-D] DYNAGEN? X4 CRT-D	52401	[Abott] [CRT-D] Quadra Assura MP
22301	[Boston Scientific] [CRT-D] DYNAGEN? CRT-D	59999	[Abott] [No applicable models] Free comment
22401	[Boston Scientific] [CRT-D] INCEPTA? CRT-D		

STATISTICAL ANALYSIS PLAN

Placebo-controlled, double-blind study of empagliflozin and implantable cardioverter-defibrillator in patients with type 2 diabetes

EMPA-ICD

jRCTs031180120

Clinical and Translational Research Center, Niigata University Medical & Dental Hospital

Date: JAN 24, 2022 Ver1.1.0

Revision History

Revision	Date of preparation	Author	Description of change
1.0.0	Dec 8, 2021	Takahiro Tanaka	First edition prepared
1.10	Jan 24, 2022	Takahiro Tanaka	2.5 Determination of Sample Size<br><br>Before revision:<br>differences in the number of arrhythmias<br><br>After revision:<br>the number of arrhythmias as the objective variable

Table of Contents

1. INTRODUCTION 1

2. STUDY OBJECTIVE AND DESIGN 1

2.1. Study Objectives 1

2.2. Study Design 1

2.3. Randomization method 1

2.4. Time Points of Evaluation 2

2.5. Determination of Sample Size 3

3. ENDPOINTS 3

3.1. Efficacy Endpoints 3

3.2. Safety Endpoints 4

4. DEFINITION OF DERIVED VARIABLE 5

5. ANALYSIS SETS 5

5.1. Full analysis set (FAS) 5

5.2. Per protocol set (PPS) 6

5.3. Safety analysis set (SAS) 6

6. DATA HANDLING 6

6.2. Handling of calculated values 6

6.4. Handling of Protocol Deviations 7

6.5. Time Windows for Measurement Time Points **7**

7. STATISTICAL METHOD **8**

7.1. Analysis Sets **8**

7.1.1. Sample Size of Each Analysis Set 8

7.2. Demographic Data and Baseline Subject Characteristics **8**

7.2.1. The summaries of demographic data and baseline subject characteristics 8

7.2.1.1. Analysis set 8

7.2.1.2. Analytical parameters 8

7.2.1.3. Analysis methods 10

7.2.1.4. Level of significance and confidence coefficient 10

7.3. Treatment Compliance Status **10**

7.4. Efficacy Analysis **11**

7.4.1. Primary Endpoint 11

7.4.1.1. Analysis set 11

7.4.1.2. Analytical parameter 11

7.4.1.3. Analysis method 11

7.4.1.4. Level of significance and confidence coefficient 11

7.4.2. Secondary Endpoints 11

7.4.2.1. Analysis set 11

7.4.2.2. Analytical parameters 11

7.4.2.3. Analysis method 12

7.4.2.4. Level of significance and confidence coefficient 14

7.5. Safety Analysis **15**

7.5.1. Tabulation of Adverse Events 16

7.5.1.1. Analysis set 16

7.5.1.2. Analytical parameters 16

7.5.1.3. Analysis method 16

7.5.1.4. Level of significance and confidence coefficient 16

8. SOFTWARE TO BE USED FOR STATISTICAL ANALYSIS **16**

9. CHANGES FROM THE PROTOCOL SPECIFIED FOR STATISTICAL ANALYSIS **16**

10. LIST OF TABLES 17

11. LIST OF FIGURES 18

Appendix 19

List of Abbreviations and Definition of Terms

Abbreviations	Meaning
FAS	Full Analysis Set
IRB	Institutional Review Board
PPS	Per Protocol Set
LVEF	Left Ventricular Ejection Fraction

1. INTRODUCTION

This document provides more detailed information on the statistical analysis plan for the efficacy and safety of the "Double-blind, placebo-controlled study to evaluate the change in the number of severe arrhythmias after empagliflozin intervention in patients with an implanted arrhythmia treatment device complicated by type 2 diabetes mellitus" in addition to the information in the study protocol.

2. STUDY OBJECTIVE AND DESIGN

2.1. Study Objectives

The main objective of this study is to determine whether empagliflozin improves the number of severe arrhythmia events in patients with arrhythmia treatment device implantation complicated by type 2 diabetes.

2.2. Study Design

Multicenter, prospective, placebo-controlled, randomized, double-blind, parallel-group, physician-initiated clinical study

2.3. Randomization method

Stratified randomization: stratification factor “LVEF/Age/Sex”

2.4. Time Points of Evaluation

Enrollment and allocation (between Screening and Visit [1]) | Initiation of study drug (between Visit [1] and Visit [2])

	Before treatment		Treatment period		
	Screening	Visit [1]	Visit [2]	Visit [3]	At time of discontinuation[*11]
	Week -12 to 0	Week 0	Week 12	Week 24[*10]	
		–	(± 4 weeks)	(± 4 weeks)	
Patient information/ informed consent	○				
Patient background	○				
Interview/physical examination	○	○	○	○	△
Confirmation of drug compliance			○	○	△
Confirmation of compliance with study procedures		○	○	○	△
Confirmation of other drugs being used	○	○	○	○	△
Height	○				△
Body weight/body temperature	○	○	△	○	△
Blood pressure/ pulse rate[*1]		○	△	○	△
Hematological tests	○[*4,5]	○[*4]	△	○	△
Hematological tests (blood glucose/lipids)[*2]	○[*4,5]	○[*4]	△	○	△
Hematological tests (special)[*2]		○[*6,7,8]		○[*7,8]	△[*7,8]
12-lead ECG		○[*4]		○	△
Holter monitoring		○[*4]		○	△
Echocardiography	○[*4]	○[*4]		○	△
^{123}I-MIBG myocardial scintigraphy		△[*4,8]		△[*8]	△[*8]
Adverse events[*3]	○	○	○	○	△
Evaluation of arrhythmia device		○[*9]		○	△

For details of *1-11, refer to Protocol 8.1.

2.5. Determination of Sample Size

The planned sample size has been determined to be 105 subjects per group, with a total of 210 subjects in both groups.

[Rationale for sample size determination]

On the basis of the previous report[9] and by using a generalized linear model for the number of arrhythmias as the objective variable, the incidence rate ratio (IRR) of severe arrhythmia in the placebo to empagliflozin group is assumed to be 1.44. Thus, at a significance level of 0.05 and power of 80%, 99 patients per group will be necessary to detect an IRR (i.e., effect size) of 1.44. Assuming that 10 patients (5%) will drop out during the study, 200 patients are estimated to be required. The number of patients who can feasibly be enrolled is 210, assuming a total of 20 institutions, each of which can recruit 10 to 15 patients. Thus, the required number of patients is feasible, and the target number of patients has been set at 210.

Number of subjects required per power

power	Sample Size
60%	124 subjects (62+62)
70%	156 subjects (78+78)
80%	198 subjects (99+99)
90%	266 subjects (133+133)

3. ENDPOINTS

3.1. Efficacy Endpoints

3.1.1. Primary Endpoint

The number of severe arrhythmia events (NSVT/VT/VF) recorded in the arrhythmia device will be evaluated at Week 0 and 24 (or at study discontinuation) and used to calculate the difference in the number of severe arrhythmia events between the empagliflozin and placebo groups.

3.1.2 Secondary Endpoints

1) The number of severe arrhythmia events (NSVT/VT/VF) and appropriate device operations (anti-tachycardia pacing/shock operation) recorded in the arrhythmia device will be evaluated at Week 0 and 24 (or at study discontinuation) and used to calculate the following:

(1-1) Change in the number of severe arrhythmia events

(1-2) Incidence of severe arrhythmia events before and after treatment

(1-3) Number of severe arrhythmia events at Week 24
(1-4) Presence or absence of severe arrhythmia events at Week 24
(1-5) Change rate of the number of appropriate device operations
(1-6) Change in the number of appropriate device operations
(1-7) Incidence of appropriate device operations before and after treatment
(1-8) Number of appropriate device operations at Week 24
(1-9) Presence or absence of appropriate device operations at Week 24

These variables will be compared between the empagliflozin and placebo groups.

In addition, the following will be evaluated at Week 0 and 24 (or at study discontinuation) to compare the change rate, change, and values at Week 24 between the empagliflozin and placebo groups:

2) Holter electrocardiographic tests: total recording time, total number of ventricular premature complexes (VPCs), number of single VPCs, number of occurrences of two consecutive VPCs, and number of ventricular tachycardia
3) Blood ketone body fraction (acetoacetic acid, 3-hydroxybutyric acid, and total ketone bodies), i.e., blood concentration of each ketone body
4) Blood catecholamine fraction (adrenalin, noradrenalin, and dopamine), i.e., blood concentration of each catecholamine

The correlation between the difference in the number of severe arrhythmia events and blood ketone body or catecholamine fraction will be compared between the empagliflozin and placebo groups.

3.2. Safety Endpoints

1) Adverse events and adverse reactions that occur in the period from the date of informed consent until Week 24 (or study discontinuation).

3.2.1. Definition of Adverse Events

An adverse event is defined as any untoward medical occurrence (including laboratory abnormalities) in patients administered a study drug; it does not necessarily have to have a causal relationship with the study drug. Among such adverse events, those whose causal relationship to the study drug cannot be ruled out are called "disease or the like." In case of any adverse event, the Investigator or Sub-investigator will promptly take appropriate measures to treat the patient and ensure their safety. In addition, if administration of the study drug is discontinued or treatment is required for adverse events, the Investigator or Sub-investigator will inform patients accordingly. For measures to be taken in the event of adverse events, refer to Appendix G (Procedure for Reporting Adverse Events).

[Adverse reactions to empagliflozin expected from the package insert] (Refer to Appendix A)
Clinically significant adverse reactions: hypoglycemia, dehydration, ketoacidosis, pyelonephritis, and sepsis
Other adverse reactions: infections (cystitis, urinary tract infection, asymptomatic bacteriuria,

vulvovaginal candidiasis, trichomoniasis, bacterial vaginitis, vulvovaginitis); reproductive system disorders (balanitis, genital pruritus, balanoposthitis, vulvovaginal discomfort, and vulvovaginal pruritus); metabolism and nutrition disorders (hyperlipidemia and decreased fluid volume); blood and lymphatic system disorders (hemoconcentration); nervous system disorders (dizziness and dysgeusia); gastrointestinal disorders (constipation and abdominal distention); skin and subcutaneous tissue disorders (rash, pruritus, and urticaria); renal and urinary disorders (pollakiuria, polyuria, increased urine output, and dysuria); general disorders (thirst and feeling hungry), and investigations (weight decreased, urine ketone body present, and blood ketone body present)
According to the guidance of the US Food and Drug Administration (FDA; dated August 29, 2018), attention should be paid also to the onset of necrotizing fasciitis of the perineum (Fournier’s gangrene) as a clinically significant adverse reaction.

3.2.2. Definition of SAEs

SAE is any adverse event that results in one of the following outcomes:
(1) Death
(2) Disability
(3) Possibly leading to death
(4) Possibly leading to disability
(5) Requiring inpatient hospitalization or prolongation of existing hospitalization in a hospital or clinic
(6) Serious outcome that is equivalent to the outcomes (1)-(5)
(7) Congenital anomaly or birth defect in next generations

“Hospitalization” in (5) does not include that intended only for therapy or examination that has been planned before the study (such as planned surgery and examination) (events that are newly developed during the hospitalization will be regarded as adverse events).

4. DEFINITION OF DERIVED VARIABLE

The derived variable is defined in the table below.

Derived variable	Calculation
Age	Subtract [date of birth] from [date of measurement]

5. ANALYSIS SETS

5.1. Full analysis set (FAS)

FAS includes all subjects enrolled in this study but excludes subjects with major protocol violation (failure to obtain informed consent and major procedural violation).

5.2. Per protocol set (PPS)

PPS includes all enrolled subjects but excludes subjects with any of the following major violations of the criteria for study treatment and concomitant therapy specified in the study protocol:

- Violation of the inclusion criteria
- Violation of the exclusion criteria
- Violation of the prohibited concomitant drugs and therapies

5.3. Safety analysis set (SAS)

SAS includes all enrolled subjects but excludes subjects who did not take any dose of the study drug.

6. DATA HANDLING

6.1.1. Handling of Missing Data

In principle, missing values will not be imputed; that is, missing data will be rejected.

6.2. Handling of calculated values

Geometric mean value, geometric coefficient of variation

Geometric mean value = exp (mean logarithmically transformed value)

geometric coefficient of variation =

$$100 \times \sqrt{\text{exp(standard deviationof of logarithmic transformed value}^2) - 1}$$

6.3. Handling of Number of Display Digits

(1) Number of display digits

The number of display digits for summary statistics is increased to the number of digits of the measurement value plus 1 digit. Minimum and maximum values are displayed with the same digits as data obtained. Test statistics are rounded to 2 decimal places in principle. P values are rounded to 4 decimal places.

6.4. Handling of Protocol Deviations

Analytical handling of subjects and data in which protocol deviation is identified is defined in the table below.

Items of protocol deviations		Subject handling				Data handling
		FAS	mITT	PPS	SAS	
Violation of [Ethical Guidelines for Medical and Health Research Involving Human Subjects]		×	×	×	×	
Subjects who never received the study drug		○	×	×	×	
Violation of inclusion/exclusion criteria		○	○	×	○	All data will be acceptable.
Procedural deviation	Non-compliance with the dosage regimen	○	○	×	○	All data will be acceptable.
	Violation of prohibited concomitant drugs	○	○	×	○	All data will be acceptable.
	Missed examinations, etc.	○	○	×	○	All data will be acceptable.
	Violation of discontinuation criteria	○	○	×	○	Data obtained after consent withdrawal will be rejected.
Violation of the time window for measurement time points		○	○	×	○	Data handling is specified in 6.5. Time Windows for Measurement Time Points.

6.5. Time Windows for Measurement Time Points

Time windows for measurement time points for analyses are specified in the table below.

Scheduled evaluation time point	Time window (day)	Remarks
Visit 0 Eligibility check	-84<, ≤-1	If 2 or more values are available within the time window, the value obtained on the day closest to the scheduled time point will be used. (If the 2 dates are different from the scheduled time point by the same number
Visit 1 (day 0)	0	
Visit 2 (day 84)	1<, ≤ 126	
Visit 3 (day 168)	126<, ≤ 196	

7. STATISTICAL METHOD

7.1. Analysis Sets

7.1.1. Sample Size of Each Analysis Set

No. of subjects who gave their consent, No. of enrolled subjects, No. of subjects randomized to each group, No. of subjects and withdrawals of FAS, PPS and SAS of each group, and No. of subjects excluded from each analysis set by the reason will be shown in tables and patient flow diagram. In addition, the list of withdrawals, subjects excluded from each analysis set and deviations will be shown in a table.

7.2. Demographic Data and Baseline Subject Characteristics

7.2.1. The summaries of demographic data and baseline subject characteristics

7.2.1.1. Analysis set

The analysis set will be the FAS.

7.2.1.2. Analytical parameters

The following baseline characteristics information will be used in the analysis.

Characteristic	Data type	Definition/classification
Sex	Binary	Man/woman
Age	measured value	Descriptive statistics
	Category	~19/20~29/30~39/40~49/<br>50~59/60~69/70~
Smoking history	Category	Current/Ex/Never
Alcohol drinking history	Category	~19/20~29/30~39/40~49/<br>50~59/60~69/70~
Life-threatening arrhythmia that resulted in implantation of arrhythmia device	Category	Ventricular fibrillation/monomorphic ventricular tachycardia/polymorphic ventricular tachycardia/nonsustained ventricular tachycardia
Underlying diseases [*1]	Binary	Present/Absent
Maker of arrhythmia treatment device	Category	BIOTRONIK/Boston Scientific/<br>Japan Lifeline/Medtronic/Abbott
Type of arrhythmia treatment device	Category	See Appendix: List of the types of arrhythmia treatment devices
Comorbidity and past history [*2]	Binary	Present/Absent

Past history of non-drug treatment [*3]	Binary	Present/Absent
NYHA classification	Category	I/II/III/IV
Height	measured value	Descriptive statistics
Body weight	measured value	Descriptive statistics
Body temperature	measured value	Descriptive statistics
Blood pressure (SBP/DBP)	measured value	Descriptive statistics
Pulse rate	measured value	Descriptive statistics
Hematological [*4]	measured value	Descriptive statistics
Blood biochemical [*5]	measured value	Descriptive statistics
Blood glucose tests<br>Fasting blood glucose, HbA1c	measured value	Descriptive statistics
Lipid tests<br>TG, TC, HDL-C, LDL-C	measured value	Descriptive statistics
Hematological tests (special)<br>Blood ketone body fraction[*6]<br>Blood catecholamine fraction[*7]<br>DNA tests [*8]	measured value	Descriptive statistics
12-lead ECG tests<br>Heart rate and rhythm; PQ, QRS, and QT interval	measured value	Descriptive statistics
Holter monitoring<br>Total recoding time, total number of VPCs, number of single VPCs, number of occurrences of two consecutive VPCs, and number of ventricular tachycardia	measured value	Descriptive statistics

Echocardiographic tests *9	measured value	Descriptive statistics
^{123}I-MIBG myocardial scintigraphy tests<br>H/M ratio and washout rate	measured value	Descriptive statistics
Evaluation of arrhythmia device *10	measured value	Descriptive statistics

*1. Ischemic heart disease, cardiac valve disease, dilated cardiomyopathy, hypertrophic cardiomyopathy, restrictive cardiomyopathy, arrhythmogenic right ventricular cardiomyopathy, left ventricular non-compaction, mitochondrial cardiomyopathy, other cardiomyopathy, Brugada syndrome, long QT syndrome, idiopathic ventricular fibrillation, etc.
*2. Past history of atrial fibrillation, hypertension, dyslipidemia, cerebral infarction, or cerebral hemorrhage
*3. PCI, CABG, cardiac valvulopathy surgery, catheter ablation, etc.
*4. RBC count, WBC count, Hb, Ht, and Plt
*5. Total protein, albumin, AST, ALT, ALP, T-Bil, LDH, BUN, Na, K, Cl, uric acid, Cr, and eGFR
*6. Acetoacetic acid, 3-hydroxybutyric acid, and total ketone bodies
*7. Adrenalin, noradrenalin, dopamine, erythropoietin, and reticulocytes
*8. Telomere length, G-tail length, RNA tests (P53, P21 and P16), and metabolome analysis
*9. Systolic capacity (LVEF) and diastolic capacity (LVEF, E wave, A wave, E/A, sep-e', lat-e', E/e', LAVI, peak TRV)
*10. Severe arrhythmia (NSVT/VT/VF), number of events required to appropriately treat severe arrhythmia (anti-tachycardia pacing/shock operation), biological monitoring indicators, and number of atrial fibrillations

7.2.1.3. Analysis methods

Frequency and proportion will be calculated for discrete data; summary statistics (number of subjects, mean, standard deviation, minimum, median, and maximum) will be calculated for continuous data.

7.2.1.4. Level of significance and confidence coefficient

All tests will be two-sided at a significance level of 5%. The two-sided confidence interval will be used with a confidence coefficient of 95%.

7.3. Treatment Compliance Status

7.3.1. Summary Statistics of Treatment Compliance Data

7.3.1.1. Analysis set

The analysis of treatment compliance will be based on the SAS population.

7.3.1.2. Analytical parameters

Medication compliance at 12 weeks and 24 weeks (or discontinuation)

7.3.1.3. Analysis method

Listing of medication compliance at 12 weeks and 24 weeks (or discontinuation).

7.4. Efficacy Analysis

7.4.1. Primary Endpoint

7.4.1.1. Analysis set

The primary efficacy analysis will be based on the FAS population.

7.4.1.2. Analytical parameter

The number of severe arrhythmia events (NSVT/VT/VF) recorded in the arrhythmia device will be evaluated at Week 0 and 24 (or at study discontinuation) to calculate the difference in the number of severe arrhythmia events between the empagliflozin and placebo groups.

7.4.1.3. Analysis method

The number of severe arrhythmia events (NSVT/VT/VF) recorded in the arrhythmia device will be evaluated at Week 0 and 24 (or at study discontinuation) and subjected to Poisson regression analysis by the generalized linear model, whereby the difference in the number of severe arrhythmia events between the empagliflozin and placebo groups will be used as an objective variable.

7.4.1.4. Level of significance and confidence coefficient

All tests for efficacy evaluation will be two-sided at a significance level of 5%. The two-sided confidence interval will be used with a confidence coefficient of 95%.

7.4.2. Secondary Endpoints

7.4.2.1. Analysis set

The secondary efficacy analysis will be based on the FAS population.

7.4.2.2. Analytical parameters

1) The number of severe arrhythmia events (NSVT/VT/VF) and appropriate device operations (anti-tachycardia pacing/shock operation) recorded in the arrhythmia device will be evaluated at Week 0 and 24 (or at study discontinuation) to calculate the following:

(1-1) Change in the number of severe arrhythmia events
(1-2) Incidence of severe arrhythmia events before and after treatment
(1-3) Number of severe arrhythmia events at Week 24
(1-4) Presence or absence of severe arrhythmia events at Week 24
(1-5) Change rate of number of appropriate device operations
(1-6) Change in number of appropriate device operations
(1-7) Incidence of appropriate device operations before and after treatment
(1-8) Number of appropriate device operations at Week 24

(1-9) Presence or absence of appropriate device operations at Week 24

These variables will be compared between the empagliflozin and placebo groups.

In addition, the following will be evaluated at Week 0 and 24 (or at study discontinuation) to compare the change rate, change, and values at Week 24 between the empagliflozin and placebo groups:

2) Holter electrocardiographic tests: total recording time, total number of VPCs, number of single VPCs, number of occurrences of two consecutive VPCs, and number of ventricular tachycardia

3) Blood ketone body fraction (acetoacetic acid, 3-hydroxybutyric acid, and total ketone bodies), i.e., blood concentration of each ketone body

4) Blood catecholamine fraction (adrenalin, noradrenalin, and dopamine), i.e., blood concentration of each catecholamine

The correlation between the difference in the number of severe arrhythmia events and blood ketone body or catecholamine fractions will be compared between the empagliflozin and placebo groups.

7.4.2.3. Analysis method

[Primary analysis]:

1) The number of severe arrhythmia events (NSVT/VT/VF) and appropriate device operations (anti-tachycardia pacing/shock operation) recorded in the arrhythmia device will be evaluated at Week 0 and 24 (or at study discontinuation).

(1-1) Change in the number of severe arrhythmia events

The difference in the change in the number of severe arrhythmia events until Week 0 and from Week 0 to 24 (or study discontinuation) will be compared between the empagliflozin and placebo groups by a two-sample test.

(1-2) Incidence of severe arrhythmia events before and after treatment

Incidences of severe arrhythmia events until Week 0 and from Week 0 to 24 (or study discontinuation) in the empagliflozin and placebo groups will be summarized.

(1-3) Number of severe arrhythmia events at Week 24

The difference in the number of severe arrhythmia events from Week 0 to 24 (or study discontinuation) will be compared between the empagliflozin and placebo groups by a two-sample test.

(1-4) Presence or absence of severe arrhythmia events at Week 24

The difference in the incidence of severe arrhythmia events from Week 0 to 24 (or study discontinuation) will be compared between the empagliflozin and placebo groups by Fisher's exact test.

(1-5) Change rate of number of appropriate device operations

The change rates of the number of appropriate device operations until Week 0 and from Week 0 to 24 (or study discontinuation) in the empagliflozin and placebo groups will be summarized.

(1-6) Change in number of appropriate device operations

The difference in the change in the number of appropriate device operations until Week 0 and from Week 0 to 24 (or study discontinuation) will be compared between the empagliflozin and placebo groups by a two-sample test.

(1-7) Incidence of appropriate device operations before and after treatment

The incidences of appropriate device operations until Week 0 and from Week 0 to 24 (or study discontinuation) and the 95% confidence interval of the empagliflozin and placebo groups will be calculated.

(1-8) Number of appropriate device operations at Week 24

The difference in the number of appropriate device operations from Week 0 to 24 (or study discontinuation) will be compared between the empagliflozin and placebo groups by a two-sample test.

(1-9) Presence or absence of appropriate device operations at Week 24

The difference in the incidence of appropriate device operations from Week 0 to 24 (or study discontinuation) will be compared between the empagliflozin and placebo groups by Fisher's exact test.

2) Holter electrocardiographic tests: total recording time, total number of VPCs, number of single VPCs, number of occurrences of two consecutive VPCs, and number of ventricular tachycardia.

For the total recording time, total number of VPCs, number of single VPCs, number of occurrences of two consecutive VPCs, and number of ventricular tachycardia at each time point of the Holter electrocardiographic tests in the empagliflozin and placebo groups, the number of participants, mean, median, standard deviation, and interquartile range will be calculated. The change over time at each measurement time in individual participants will be displayed by treatment group in a graph (mean ± standard deviation).

3) Blood ketone body fraction (acetoacetic acid, 3-hydroxybutyric acid, and total ketone bodies), i.e., blood concentration of each ketone body

For acetoacetic acid, 3-hydroxybutyric acid, and total ketone bodies in the empagliflozin and placebo groups, the number of participants, mean, median, standard deviation, and interquartile range will be calculated. The change over time at each measurement time in individual participants will be displayed by treatment group in a graph (mean ± standard deviation).

4) Blood catecholamine fraction (adrenalin, noradrenalin, and dopamine), i.e., blood concentration of each catecholamine

For adrenalin, noradrenalin, and dopamine in the empagliflozin and placebo groups, the number of participants, mean, median, standard deviation, and interquartile range will be calculated. The change over time at each measurement time in individual participants will be displayed by treatment group in a graph (mean ± standard deviation).

7.4.2.4. Level of significance and confidence coefficient

All tests for efficacy evaluation will be two-sided at a significance level of 5%. The two-sided confidence interval will be used with a confidence coefficient of 95%.

7.4.3. Other Endpoints

7.4.3.1. Analysis set

The analysis of other variables will be based on the FAS population.

7.4.3.2. Analytical parameters

1) The number of atrial fibrillation events and the biological monitoring index recorded in the arrhythmia device will be evaluated at Week 0 and 24 to calculate the following, and the relationship with ventricular arrhythmia also will be examined:

(1-1) Change rate of the number of atrial fibrillation events
(1-2) Change in the number of atrial fibrillation events
(1-3) Incidence of atrial fibrillation events before and after treatment
(1-4) Number of atrial fibrillation events at Week 24
(1-5) Presence or absence of atrial fibrillation events at Week 24
(1-6) Change rate of biological monitoring index
(1-7) Change in biological monitoring index
(1-8) Biological monitoring index at Week 24

These variables will be compared between the empagliflozin and placebo groups.

2) Each of the following items will be evaluated at Week 0 and 24 (or at study discontinuation) to compare the change rate, change, and value at Week 24 between the empagliflozin and placebo groups:

(2-2) NYHA classification of cardiac function
(2-3) Body weight
(2-4) Blood pressure, pulse rate, and body temperature
(2-5) Blood glucose, assessed by HbA1c and fasting blood glucose
(2-6) Serum lipid, serum uric acid, and the cardiac failure markers TC, HDL-C, LDL-C, TG, UA, and BNP
(2-7) Renal function, assessed by serum Cr, eGFR, BUN, Na, K, and Cl
(2-8) Hepatic function, assessed by total protein, albumin, AST, ALT, ALP, T-Bil, and LDH
(2-9) Hematological value, assessed by RBC count, WBC count, Hb, Ht, and Plt
(2-10) Erythropoietin
(2-11) Reticulocytes
(2-12) DNA tests (telomere length)
(2-13) RNA tests (P53, P21, and P16)

(2-14) Metabolome analysis
(2-15) 12-lead ECG (pulse rate and PQ, QRS, and QT intervals)
(2-16) Systolic and diastolic capacity measured by echocardiography (LVEF, E wave, A wave, E/A, sep-e', lat-e', E/e', LAVI, peak TRV, and HR)
(2-17) ^{123}I-MIBG myocardial scintigraphy-obtained sympathetic activity index, assessed by heart-to-mediastinum ratio (H/M), and washout rate

3) Subgroup analysis will be performed on the following items, as necessary:
(3-1) Blood glucose control
(3-2) Concentration of ketone bodies
(3-3) Increase in erythropoietin
(3-4) Renal function
(3-5) Hepatic function

7.4.3.3. Analysis method

[Primary analysis]:
For each endpoint, changes in the measured values over time are shown graphically for each time point in each treatment group (as mean ± standard deviation). The number of subjects, means, medians, standard deviations, and interquartile ranges will be calculated for each time period and measurement time point. For the discrete variables, the proportions will be determined at each time point in each treatment group. The number of subjects, proportions, and 95% confidence intervals will also be calculated for each time period and measurement time point.

[Secondary analysis]:
Secondary analysis will not be performed in this study.

7.4.3.4. Level of significance and confidence coefficient

All tests for efficacy evaluation will be two-sided at a significance level of 5%. The two-sided confidence interval will be used with a confidence coefficient of 95%.

7.5. Safety Analysis

An adverse event is any unfavorable and unintended sign (including an abnormal laboratory change), symptom, or disease that develops in a subject, whether or not related to the study drug. Underlying diseases are not considered as adverse events, except where the severity worsens or the frequency increases after study treatment. A laboratory change is determined to be an adverse event if it is considered to be unfavorable for the subject by the investigators. The causal relationship between an adverse event and the study drug will be determined

according to the following criteria. An adverse event considered related to the study drug is regarded as an adverse drug reaction.

Related: There is a reasonable possibility of the causal relationship between the study treatment and an adverse event.

Not related: There is no reasonable possibility of the causal relationship between the study treatment and an adverse event.

7.5.1. Tabulation of Adverse Events

7.5.1.1. Analysis set

The analysis of adverse events will be based on the SAS population.

7.5.1.2. Analytical parameters

SAEs occurring from the initiation of study treatment through after the end of study.

7.5.1.3. Analysis method

A list of adverse events will be prepared showing the number of events and participants. Adverse reactions will be classified as “related” or “not related.”

7.5.1.4. Level of significance and confidence coefficient

All tests for safety evaluation will be two-sided at a significance level of 5%. The two-sided confidence interval will be used with a confidence coefficient of 95%.

8. SOFTWARE TO BE USED FOR STATISTICAL ANALYSIS

SAS for Windows (release 9.4 or later) will be used for statistical analysis.

9. CHANGES FROM THE PROTOCOL SPECIFIED FOR STATISTICAL ANALYSIS

Not applicable.

10. LIST OF TABLES

Table No.	Details
T7.1.1	List of discontinued cases
T7.1.2	List of cases excluded from analysis
T7.2	Subject demographics
T7.3.1	Study drug compliance rate
T7.4.1.1	Primary endpoint: difference in number of serious arrhythmic events
T7.4.2.1.1	Changes in number of serious arrhythmic events
T7.4.2.1.2	Pre- and post-treatment incidence of severe arrhythmic events
T7.4.2.1.3	Number of severe arrhythmic events at 24 weeks
T7.4.2.1.4	Presence of severe arrhythmic events at 24 weeks
T7.4.2.1.5	Percentage change in number of appropriate shocks
T7.4.2.1.6	Changes in number of appropriate shocks
T7.4.2.1.7	Pre- and post-treatment incidence of appropriate shocks
T7.4.2.1.8	Number of appropriate shocks at 24 weeks
T7.4.2.1.9	Presence of appropriate shocks at 24 weeks
T7.4.2.2	Holter ECG examination: total recording time, total number of VPCs, number of single VPCs, number of double VPCs, number of ventricular tachycardia
T7.4.2.3	Blood ketone fractions (acetoacetic acid, 3-hydroxybutyric acid, total ketones): concentration of blood ketone bodies
T7.4.2.4	Blood catecholamine fractions (adrenaline, noradrenaline, dopamine): catecholamine concentration in each blood sample
T7.4.3.1.1	Percent change in number of atrial fibrillation events
T7.4.3.1.2	Changes in number of atrial fibrillation events
T7.4.3.1.3	Pre- and post-treatment incidence of atrial fibrillation events
T7.4.3.1.4	Number of atrial fibrillation events at 24 weeks
T7.4.3.1.5	Presence of atrial fibrillation events at 24 weeks
T7.4.3.1.6	Percentage change in biomonitoring indicators
T7.4.3.1.7	Changes in biomonitoring indicators
T7.4.3.1.8	Biomonitoring indicators at 24 weeks
T7.4.3.2	For each parameter, change and percentage change from week 0 to week 24 (or discontinuation) and value at 24 weeks

11. LIST OF FIGURES

Figure No.	Details
F7.1.1	Details of subjects
F7.4.1.1	Primary endpoint: difference in number of serious arrhythmic events
F7.4.2.1.1	Change in number of serious arrhythmic events
F7.4.2.1.3	Number of severe arrhythmic events at 24 weeks
F7.4.2.1.6	Change in number of appropriate shocks
F7.4.2.1.8	Number of appropriate shocks at 24 weeks
F7.4.3.1.2	Change in number of atrial fibrillation events
F7.4.3.1.4	Number of atrial fibrillation events at 24 weeks
F7.4.3.1.7	Change in biomonitoring indicators
F7.4.3.2	For each parameter, change and percentage change from week 0 to week 24 (or discontinuation) and value at 24 weeks
T7.5.1	Percentage of adverse events

Appendix

**Relevant information that cannot be included in the main body of this document are described and indicated in this section as necessary.*

List of the types of arrhythmia treatment devices

11101	[BIOTRONIK] [ICD] Ilivia 7 DR-T DF-1 ProMRI	22402	[Boston Scientific] [CRT-D] COGNIS 100-D
11102	[BIOTRONIK] [ICD] Ilivia 7 DR-T DF4 ProMRI	29999	[Boston Scientific] [No applicable models] Free comment
11103	[BIOTRONIK] [ICD] Ilivia 7 VR-T DX DF-1 ProMRI	31101	[Japan Lifeline] [ICD] Ovatio
11104	[BIOTRONIK] [ICD] Ilivia 7 VR-T DF-1 ProMRI	31201	[Japan Lifeline] [ICD] PARADYM
11105	[BIOTRONIK] [ICD] Ilivia 7 VR-T DF4 ProMRI	31202	[Japan Lifeline] [ICD] PARADYM 2
11201	[BIOTRONIK] [ICD] Inlexa 7 DR-T DF-1	31203	[Japan Lifeline] [ICD] PARADYM RF
11202	[BIOTRONIK] [ICD] Inlexa 7 DR-T DF4	31301	[Japan Lifeline] [ICD] INTENSIA
11203	[BIOTRONIK] [ICD] Inlexa 7 VR-T DX DF-1	31401	[Japan Lifeline] [ICD] PLATINIUM
11204	[BIOTRONIK] [ICD] Inlexa 7 VR-T DF4	32501	[Japan Lifeline] [CRT-D] PARADYM 2
11301	[BIOTRONIK] [ICD] Iperia 7 DR-T DF4 ProMRI	32502	[Japan Lifeline] [CRT-D] PARADYM RF
11302	[BIOTRONIK] [ICD] Iperia 7 VR-T DX DF-1 ProMRI	32601	[Japan Lifeline] [CRT-D] INTENSIA
11303	[BIOTRONIK] [ICD] Iperia 7 VR-T DF4 ProMRI	32701	[Japan Lifeline] [CRT-D] PLATINIUM
11401	[BIOTRONIK] [ICD] Itrevia 5 DR-T DF-1	39999	[Japan Lifeline] [No applicable models] Free comment
11402	[BIOTRONIK] [ICD] Itrevia 5 DR-T DF4	41101	[Medtronic] [ICD] Secura DR
11403	[BIOTRONIK] [ICD] Itrevia 5 VR-T DX DF-1	41201	[Medtronic] [ICD] Protecta XT DR
11404	[BIOTRONIK] [ICD] Itrevia 5 VR-T DF4	41301	[Medtronic] [ICD] Evera XT DR
12101	[BIOTRONIK] [CRT-D] Intica 7 HF-T QP DF-1 ProMRI	41302	[Medtronic] [ICD] Evera MRI XT DR SureScan
12102	[BIOTRONIK] [CRT-D] Intica 7 HF-T QP DF4 ProMRI	41102	[Medtronic] [ICD] Secura VR
12103	[BIOTRONIK] [CRT-D] Intica 7 HF-T DF-1 ProMRI	41202	[Medtronic] [ICD] Protecta XT VR
12104	[BIOTRONIK] [CRT-D] Intica 7 HF-T DF4 ProMRI	41303	[Medtronic] [ICD] Evera XT VR
12201	[BIOTRONIK] [CRT-D] Inlexa 7 HF-T QP DF-1	41304	[Medtronic] [ICD] Evera MRI XT VR SureScan
12202	[BIOTRONIK] [CRT-D] Inlexa 7 HF-T QP DF4	41401	[Medtronic] [ICD] Visia AF MRI XT VR
12203	[BIOTRONIK] [CRT-D] Inlexa 7 HF-T DF-1	42101	[Medtronic] [CRT-D] Consulta CRT-D
12204	[BIOTRONIK] [CRT-D] Inlexa 7 HF-T DF4	42201	[Medtronic] [CRT-D] Protecta XT CRT-D
12301	[BIOTRONIK] [CRT-D] Itrevia 7 HF-T QP ProMRI	42301	[Medtronic] [CRT-D] Viva
12302	[BIOTRONIK] [CRT-D] Itrevia 7 HF-T DF-1 ProMRI	42401	[Medtronic] [CRT-D] Compia MRI Quad CRT-D
12303	[BIOTRONIK] [CRT-D] Itrevia 7 HF-T DF4 ProMRI	42501	[Medtronic] [CRT-D] Amplia MRI Quad CRT-D
12304	[BIOTRONIK] [CRT-D] Itrevia 5 HF-T QP	42601	[Medtronic] [CRT-D] Claria MRI Quad CRT-D
12305	[BIOTRONIK] [CRT-D] Itrevia 5 HF-T DF-1	49999	[Medtronic] [No applicable models] Free comment
12306	[BIOTRONIK] [CRT-D] Itrevia 5 HF-T DF4	51101	[Abbott] [ICD] Fortify Assura DR DF4

19999	[BIOTRONIK] [No applicable models] Free comment	51102	[Abott] [ICD] Fortify Assura VR DF4
21101	[Boston Scientific] [ICD] RESONATE EL ICD VR	51201	[Abott] [ICD] Ellipse DR DF4
21102	[Boston Scientific] [ICD] RESONATE EL ICD DR	51202	[Abott] [ICD] Ellipse VR DF4
21201	[Boston Scientific] [ICD] MOMENTUM EL ICD VR	51203	[Abott] [ICD] Ellipse Limited DR
21202	[Boston Scientific] [ICD] MOMENTUM EL ICD DR	51204	[Abott] [ICD] Ellipse Limited VR
21301	[Boston Scientific] [ICD] CHARISMA EL ICD VR	51301	[Abott] [ICD] AnalyST DR RF
21302	[Boston Scientific] [ICD] CHARISMA EL ICD DR	51302	[Abott] [ICD] AnalyST VR RF
21401	[Boston Scientific] [ICD] PERCIVA MINI ICD VR	51401	[Abott] [ICD] Current DR RF
21402	[Boston Scientific] [ICD] DYNAGEN? MINI ICD	51402	[Abott] [ICD] Current VR RF
21403	[Boston Scientific] [ICD] DYNAGEN? EL ICD	51501	[Abott] [ICD] FortifyST DR
21404	[Boston Scientific] [ICD] INCEPTA? ICD	51502	[Abott] [ICD] FortifyST VR
21501	[Boston Scientific] [ICD] TELIGEN 100	52101	[Abott] [CRT-D] Unify
22101	[Boston Scientific] [CRT-D] RESONATE X4 CRT-D	52102	[Abott] [CRT-D] Unify Assura
22102	[Boston Scientific] [CRT-D] MOMENTUM X4 CRT-D	52103	[Abott] [CRT-D] Unify Quadra
22201	[Boston Scientific] [CRT-D] MOMENTUM CRT-D	52201	[Abott] [CRT-D] Quadra? Assura
22202	[Boston Scientific] [CRT-D] CHARISMA X4 CRT-D	52301	[Abott] [CRT-D] Promote RF
22203	[Boston Scientific] [CRT-D] DYNAGEN? X4 CRT-D	52401	[Abott] [CRT-D] Quadra Assura MP
22301	[Boston Scientific] [CRT-D] DYNAGEN? CRT-D	59999	[Abott] [No applicable models] Free comment
22401	[Boston Scientific] [CRT-D] INCEPTA? CRT-D		
