# Supplementary material for: Effect of empagliflozin on ventricular arrhythmias in patients with type 2 diabetes treated with an implantable cardioverter-defibrillator: the EMPA-ICD trial
Source: Cardiovasc Diabetol. 2024 Jun 28;23:224. doi: 10.1186/s12933-024-02309-9 (PMC11214255; doi:10.1186/s12933-024-02309-9)
Supplement: Supplementary file 4 — Supplementary Material 4. [file 12933_2024_2309_MOESM4_ESM.docx]

| **Supplementary Table 1. Details of underlying cardiac diseases** | | |
| --- | --- | --- |
| **Underlying cardiac diseases^a^** | **Empagliflozin**  **(n=75)** | **Placebo**  **(n=75)** |
| IHD, n (%) | 33 (44.0) | 33 (44.0) |
| DCM, n (%) | 12 (16.0) | 11 (14.7) |
| HCM, n (%) | 8 (10.7) | 10 (13.3) |
| Cardiac sarcoidosis, n (%) | 11 (14.7) | 7 (9.3) |
| Valvular heart disease, n (%) | 3 (4.9) | 4 (5.3) |
| ARVC, n (%) | 0 (0) | 2 (2.7) |
| Brugada syndrome, n (%) | 5 (6.7) | 7 (9.3) |
| LQTS, n (%) | 2 (2.7) | 1 (1.3) |
| IVF/IVT, n (%) | 5 (6.7) | 2 (2.7) |
| Others, n (%) | 8 (10.7) | 12 (16.0) |

^a^The total number of cases and percentages exceeded 75 cases and 100% in each group because some patients had multiple underlying cardiac diseases. No variable was significantly different between the two groups.

ARVC, arrhythmogenic right ventricular cardiomyopathy; DCM, dilated cardiomyopathy; HCM, hypertrophic cardiomyopathy; IHD, ischemic heart disease; IVF/IVT, idiopathic ventricular fibrillation/tachycardia; LQTS, long QT syndrome
